# Bound Water at Protein-Protein Interfaces: Partners, Roles and Hydrophobic Bubbles as a Conserved Motif

**DOI:** 10.1371/journal.pone.0024712

**Published:** 2011-09-22

**Authors:** Mostafa H. Ahmed, Francesca Spyrakis, Pietro Cozzini, Parijat K. Tripathi, Andrea Mozzarelli, J. Neel Scarsdale, Martin A. Safo, Glen E. Kellogg

**Affiliations:** 1 Department of Medicinal Chemistry, Virginia Commonwealth University, Richmond, Virginia, United States of America; 2 Center for the Study of Biological Complexity, Virginia Commonwealth University, Richmond, Virginia, United States of America; 3 Institute for Structural Biology and Drug Discovery, Virginia Commonwealth University, Richmond, Virginia, United States of America; 4 Department of General and Inorganic Chemistry, University of Parma, Parma, Italy; 5 Department of Biochemistry and Molecular Biology, University of Parma, Parma, Italy; 6 Institute of Biostructures and Biosystems, Rome, Italy; University Of Oxford, United Kingdom

## Abstract

**Background:**

There is a great interest in understanding and exploiting protein-protein associations as new routes for treating human disease. However, these associations are difficult to structurally characterize or model although the number of X-ray structures for protein-protein complexes is expanding. One feature of these complexes that has received little attention is the role of water molecules in the interfacial region.

**Methodology:**

A data set of 4741 water molecules abstracted from 179 high-resolution (≤ 2.30 Å) X-ray crystal structures of protein-protein complexes was analyzed with a suite of modeling tools based on the HINT forcefield and hydrogen-bonding geometry. A metric termed Relevance was used to classify the general roles of the water molecules.

**Results:**

The water molecules were found to be involved in: a) (bridging) interactions with both proteins (21%), b) favorable interactions with only one protein (53%), and c) no interactions with either protein (26%). This trend is shown to be independent of the crystallographic resolution. Interactions with residue backbones are consistent for all classes and account for 21.5% of all interactions. Interactions with polar residues are significantly more common for the first group and interactions with non-polar residues dominate the last group. Waters interacting with both proteins stabilize on average the proteins' interaction (−0.46 kcal mol^−1^), but the overall average contribution of a single water to the protein-protein interaction energy is unfavorable (+0.03 kcal mol^−1^). Analysis of the waters without favorable interactions with either protein suggests that this is a conserved phenomenon: 42% of these waters have SASA ≤ 10 Å^2^ and are thus largely buried, and 69% of these are within predominantly hydrophobic environments or “hydrophobic bubbles”. Such water molecules may have an important biological purpose in mediating protein-protein interactions.

## Introduction

Over the last decade there has been a growing interest in understanding and exploiting protein-protein interactions as potential new routes to disease therapeutics [1]–[8]. It is believed that if one or more critical, but often transient, protein-protein interactions could be inhibited by a peptidic or small molecule agent, this could lead to a novel and specific approach for treatment of a wide variety of human diseases. The understanding of numerous cell cycle pathways that we have developed has been nothing short of revolutionary, and these pathways repeatedly invoke protein-protein interactions, but to date few therapeutics have resulted from this knowledge [2]. One reason is that our structural knowledge of protein-protein complexes is lagging, largely because experimental X-ray crystallographic structure determinations of these complexes are demanding [9], [10]. The principal difficulty is the challenge of growing high-quality crystals for protein complexes [11], which *in vivo* are often transiently associated and disordered [12].

Nonetheless, the RCSB Protein Data Bank [13] does contain several hundred protein-protein complexes [9], although the collection is somewhat biased towards a few classes, e.g., antigen-antibody complexes. Whether the interactions at these interfaces differ from the interactions between ligands and proteins, between polynucleotides and proteins, or within a protein is a widely explored issue. To this aim an in-depth assessment of the role of water molecules located at protein-protein interfaces is particularly relevant. A small number of research groups have worked in this area over the last several years. Notably, Baker and colleagues have demonstrated that incorporating discrete water molecules in design of protein-protein interfaces [14]–[16] is critical. Janin and colleagues have analyzed hydration [17]–[19] as part of their campaign to model protein-protein structure and interactions [17]–[21]. Papoian, Ulander and Wolynes applied energy landscape theory to evaluate water-mediated recognition [22]. Keskin and Nussinov [23], [24] have described water inclusion as an alternative strategy for proteins to achieve optimum association. Pisabarro and colleagues have examined solvent at protein-protein interfaces [25]–[27] and have shown that considering water may improve protein contacts predictions [25] that are based on the principle of correlated mutations [28]-[31]. Several authors have discussed approaches for including water explicitly and implicitly in docking protocols, e.g., Jiang *et al.* [32], van Dijk and Bonvin [33], Li and Lazaridis [34], de Graaf *et al.* [35], Jackson, Gabb and Sternberg [36], and Zuo, Gandhi and Mancera [37]. Commercially available tools such as Freisner and Berne's WaterMap [38], [39] and Goodford's GRID [40] for predicting positions of waters, while quite successful in protein-ligand systems, have only begun to be used in conjunction with protein-protein docking [41]. Thus, expanding our understanding of the roles of water molecules in guiding protein-protein associations [42] and at the resulting interfaces is a significant goal with wide-ranging implications.

### Water Relevance

We have previously undertaken several studies on the roles of water in various environments. Our analyses employ the HINT model for biomolecular interaction, which is based on experimental logP for 1-octanol/water partitioning. HINT simultaneously accounts for enthalpic, entropic and solvation contributions to biological association [43]–[49]. Water molecules found at protein-DNA interfaces influence both the energetics [43] and specificity [44] of the amino acid-base interactions between the two molecules. In an examination of the free energy of dimer-dimer association for native and mutant β37 hemoglobins, the inclusion of crystallographic waters at the dimer-dimer interface improved the HINT score-based binding predictions [45]. Likewise, we showed that superior correlations of predicted binding free energies with experimental binding free energies for HIV-1 protease complexes were obtained when the contribution of water molecules bridging between the protein and inhibitors were incorporated [46]. Finally, an analysis of the waters in unliganded and ligand-bound proteins rationalized the roles of water [47] in terms of HINT score for the water with respect to its environment and Rank, a simple metric representing the number and quality of hydrogen bonds that the water can make [49].

Together, HINT and Rank comprise what we have termed “Relevance”, a global metric for describing the conservation of water between unliganded and ligand-bound states [50]. Water molecules with low Relevance are easily displaced upon ligand binding, while those with high Relevance are not. However, ligands specifically designed with functional groups that mimic the role of high Relevance waters are expected to have particularly potent binding free energy as they would gain the entropic benefit of releasing one or more of these ordered water molecules to bulk. This concept can also be applied to the study of protein-protein complexes, where many water molecules can be trapped at the protein-protein interface, again likely to be playing distinct roles – some passive and some active, e.g., in allosteric proteins like hemoglobin [51], [52].

### Roles of Waters at Protein-Protein Interfaces

The highest-level view is that there are three distinct roles for waters at these interfaces: bridging, i.e., having significant interactions with both proteins; non-bridging, i.e., having significant interactions with only one of the two proteins; or simply trapped without significant interactions with either protein. More detailed analyses may reveal additional details such as whether these classifications are dependent on the resolution of the underlying X-ray crystallographic experiment, e.g., are trapped waters more or less likely to be detected at high-resolution? We can also evaluate whether residue types have differences in interaction preferences for waters in these three categories, e.g., what residue types are most often involved in interactions with bridging waters? Water is unique in its ability to simultaneously provide two hydrogen-bond acceptor sites and two donor sites. Thus, it can effectively bridge in every way possible: donor-to-donor, donor-to-acceptor and acceptor-to-acceptor.

In this report we describe a detailed analysis of protein-protein interfaces in 179 high-resolution (better than 2.30 Å) X-ray crystal structures of protein-protein complexes extracted from the RCSB Protein Data Bank [13]. All water molecules within 4.0 Å of both proteins, 4741 unique waters, comprised the data set. We will show that only about 21% of these waters are truly bridging while 26% are seemingly only trapped at the interface. While it is probably not surprising that Asp and Glu residues appear most frequently in interactions with bridging waters, it is somewhat surprising that bridging is dominated by Asp-H_2_O-Arg and Glu-H_2_O-Arg interactions but Asp-H_2_O-Asp or Glu-H_2_O-Glu interactions are relatively infrequent, even compared to Asp-H_2_O-Glu. Also of note is that certain unfavorable interaction motifs are conserved. The results from this work have implications for the design of compounds that can break protein-protein interactions.

## Materials and Methods

### Data set

The protein-protein complexes data set was obtained from the RSCB Protein Data Bank [13] by applying search filters for several structural criteria. First, the structures were required to have at least two separate protein entities where each was at least 100 amino acids in length. Structures with either DNA or RNA were excluded as were structures with sequence identity similarity > 50% to another protein complex in the data set. The data set was restricted to structures with resolutions 2.3 Å and better. This set (1331) of PDB structures consisted of both homo and hetero protein complexes. Further screening of the structure description isolated protein-protein complexes (861) for individual inspection where only structures comprised of completely different proteins, i.e., not subunits or chains of the same protein, were retained. Finally, 179 structures (Table S1) were randomly selected from this set for analysis.

The downloaded coordinate files were prepared by first removing ligands or cofactors other than water. About one-third of the interfaces examined in this study had cofactors or ligands at or near the interface. Smaller ionic cofactors, e.g., Mg^2+^, SO_4_^2–^, etc., may affect the orientation of nearby water molecules, but should not appreciably impact whether and how those waters interact with the protein pair. While larger cofactors and ligands leave inclusion volumes at the interface when removed, only waters meeting the distance restraint are included in the data set, so the deletion of larger cofactors unlikely causes significant changes to water orientations or roles.

Then, using Sybyl 8.1 [53], hydrogen atoms were added and minimized (Tripos forcefield, with Gasteiger-Hückel charges and distance-dependent dielectric) to a gradient of 0.01 kcal mol^−1^ Å^−1^ while the non-hydrogen atoms were treated as an aggregate. Water molecules that were within 4.0 Å from atoms on both of the interacting proteins were retained with each protein-protein complex. Together, the water data set is comprised of 4741 unique water molecules, which is 5.4% of all waters in these complexes (ranging from 0.5% to 17.9%).

### Hydropathic Analysis

Each model contains two proteins and an array of solvents, and was analyzed with HINT [48], [49] by computing intermolecular scores between the proteins and the interfacial solvent arrays. The HINT score (H_TOTAL_) is a double sum over all atom-atom pairs of the product (b_ij_) of the hydrophobic atom constants (a_i_, partial log P_octanol/water_) and atom solvent accessible surface areas (S_i_) for the interacting atoms, mediated by a function of the distance between the atoms:
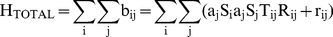
(1)where R_ij_ is a simple exponential function, e^-r^ [48], r_ij_ is an adaptation of the Lennard-Jones function [54], [55], and T_ij_ is a logic function assuming +1 or −1 values, depending on the polar (Lewis acid or base) nature of interacting atoms. HINT parameters and controls were as in previous studies [43], [44], [47]: partition calculations were performed with the “dictionary” method for the proteins with ‘essential hydrogens’, where polar hydrogens are treated explicitly and non-polar hydrogens are ‘united’ with their parent non-polar heavy atom; the HINT option that corrects the S_i_ terms for backbone amide nitrogens by adding 30 Å^2^ was used in this study to improve the relative energetics of inter- and intramolecular hydrogen bonds involving these nitrogens. Water molecules are partitioned as a “solvent set” with analogous HINT parameters. Previous work [56], [57] has suggested that approximately 500 HINT score units correspond to −1.0 kcal mol^−1^ of free energy.

Each crystallographically observed water's orientation was optimized by an exhaustive protocol [58] that maximizes the HINT score with respect to its surrounding environment by evaluating its interactions with a “receptor” created from atoms within 6.0 Å. For water molecules, this optimization rewards hydrogen bond and acid/base interactions while penalizing acid/acid and base/base interactions and those with hydrophobic entities on either of the two protein surfaces. Hydropathic interaction analysis was then performed with HINT for each of the optimized water molecules with respect to the two proteins with which it interacts. The resulting data were tabulated by frequency and strength of interactions with each amino acid residue type. In cases where a water molecule had significant interactions (> |10| HINT score units, approximately |0.02| kcal mol^−1^) with more than one residue on a protein, that water's count was fractionally distributed to interacting residues based on the absolute values of the relative HINT scores for those residues that interact with it, i.e., 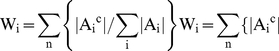
(2)where A_i_^c^ are the interaction HINT scores by residue type (i) interacting with water n. Similarly, the fractions of interactions with interfacial water molecules arising from backbone and sidechain atoms were calculated by weighted counts with A_i_^c^ representing the interaction HINT scores by i, separated into c  =  sidechain or c  =  backbone subsets. Heat maps for frequency and interaction scores and map clustering were calculated and drawn with R [59].

### Rank Algorithm

Rank represents the weighted number of potential hydrogen bonds for each water molecule with respect to a pseudo-receptor of atoms from the target molecule(s) surrounding the water. Rank is calculated as:
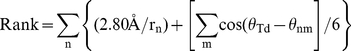
(3)where r_n_ is the distance between the water's oxygen and the target's heavy atom n (n is the number of interaction hydrogen bond donor/acceptor (doneptor) targets up to a maximum of 4). This is scaled relative to 2.8 Å, the presumed ideal hydrogen bond length. θ_Td_ is the optimum tetrahedral angle (109.5°) and θ_nm_ is the angle between targets n and m (m  =  n to number of valid targets). The algorithm thus allows a maximum number of 4 doneptor targets (≤ 2 donors and ≤ 2 acceptors). To properly weight the geometrical quality of hydrogen bonds, targets that have an angle less than 60° with respect to other (higher quality) targets are rejected [58].

### Relevance

Relevance is a synthesis of HINT score and Rank [50]. Specifically, 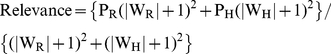
(4)where P_R_ is the percent probability for water conservation based on Rank and P_H_ the probability based on HINT score. W_R_ and W_H_ are the weights for these probabilities, respectively. The values for P_R_, P_H_, W_R_ and W_H_ are as shown in Figure 2 of reference [50]. This relationship was derived with the expectation that water molecules with Relevance ≥ 0.5 would be conserved and those with Relevance < 0.5 would be non-conserved because the waters analyzed in developing the training set were, by their nature, binary – either conserved and present in the ligand-bound complex or non-conserved and absent in the complex.

### Solvent Accessible Surface Area

A new algorithm for calculating solvent accessible surface area (SASA) was developed for this work based on the concept of Lee and Richards [60]. The results are similar to those obtained with NACCESS (S. Hubbard and J.M. Thornton[61]). The volume surrounding each atom (water) of interest was set within a cube centered at that atom and extending 2.0 Å plus twice the assumed solvent radius (1.4 Å), i.e., 4.8 Å, in ±x, ±y and ±z directions. Each grid box within the cube has a size of 0.5 Å × 0.5 Å × 0.5 Å. All grid points within the van der Waals radii of atoms in either protein or the atom of interest are marked as unavailable. For each grid point on a spherical shell of radius  =  van der Waals radius of the atom of interest plus 1.4 Å, a solid test sphere (radius 1.4 Å) is constructed. If all grid points within that test sphere are available, then the grid point at its center (on the spherical shell) is solvent accessible with surface area of 0.25 Å^2^. All such grid points are summed to obtain the atom's SASA. (See Figure S1).

## Results

Protein-protein complexes are under intense scrutiny as possible targets for new therapies, particularly in cancer [2], [62], [63] and amyloidogenic diseases [64], [65]. It has proven difficult to design molecules that can inhibit specific protein-protein associations due to the relative paucity of structural data on relevant complexes, although the number of such structures is growing [9]. Computational approaches to building reliable models of protein-protein complexes are also stymied by the sheer magnitude of the problem: in the absence of specific knowledge, there are nearly an infinite number of ways to dock two irregularly shaped objects with a relatively small surface contact area. This contrasts to the better-defined and easier problem of small molecule docking in pockets of proteins. Even there, however, no universal scoring function has emerged that can confidently predict either the docked conformation or the free energy of binding [66]-[68]. Despite these major issues, computational algorithms and protocols are being developed for macromolecular docking [69]-[73].

We [43]-[47], [50], [74], and others [14,15,17,25–27,32-35,37,39,40,75–80], have shown in numerous studies that water molecules are much more than an inconvenience to modeling in the biological environment. In fact, water plays many roles in structure: on the bulk scale by dominating the dielectric on and near protein surfaces and directing diffusion and on the individual scale by influencing both the surface shape and interaction energetics. With a few notable exceptions [32]-[37], [41], [81], [82], the effects of water on this latter (atomistic) scale have been ignored in protein-protein docking methods. Here we establish a basis for including the effects of individual waters in macromolecular docking algorithms by reporting a detailed analysis of water molecules found at protein-protein interfaces in high-resolution X-ray crystal structures.

### The Water Relevance Metric

As described above, water Relevance [50] is a descriptor combining two metrics of structure: Rank [58] and HINT score [48], where each orientation-optimized water is scored against its environment. Others [78], suggested the crystallographic B-factor as a predictor of water conservation, but we did not find it useful for our data set [50]. While Relevance was initially trained on and for protein-ligand complexes, *the role(s) that water molecules can play are independent of the stage*: water will interact favorably with up to two hydrogen bond donors and up to two hydrogen bond acceptors, and will generally avoid interaction with hydrophobic functional groups, regardless of whether these groups are in small organic molecules or in proteins.

**Figure 1:**
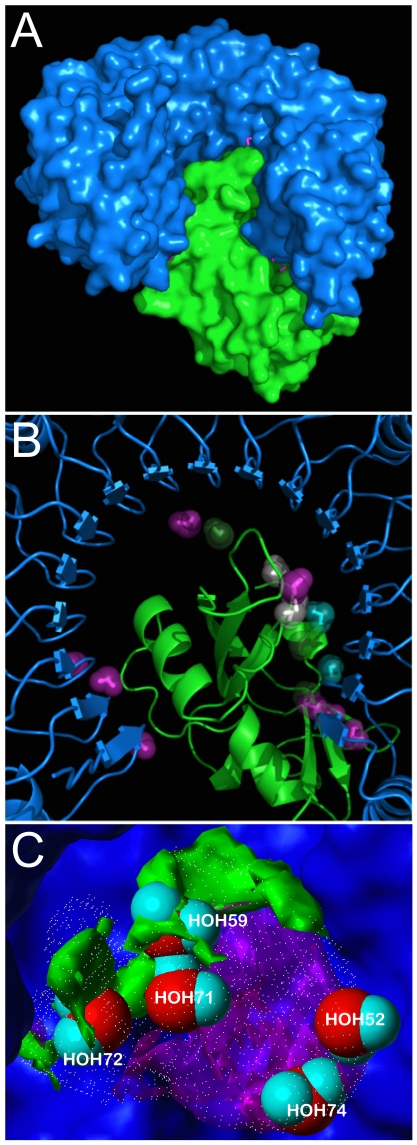
Molecular model of human placental RNase inhibitor (hRI)- human angiogenin (hAng) complex (1a4y). (A) Connolly-type surface representation with blue for hRI and green for hAng; (B) Interface region; water molecules colored blue are Relevant (≥ 0.25) with respect to hRI, green with respect to hAng, magenta with respect to both hRI and hAng, and white with respect to neither (see Table 2); (C) Of particular interest is the “hydrophobic bubble” enclosing the non-Relevant waters HOH59, HOH71 and HOH72. Note that these three waters are encompassed within a region of the cavity (rendered with white dots by VICE [84]) that is of hydrophobic character (green contours) as indicated by focused HINT complement maps [85]. HOH52 and HOH74 are also in the cavity but in a polar region (magenta contours). The pocket map is set on the surface of hRI; the structure and surface for hAng has been deleted for clarity.

**Figure 2:**
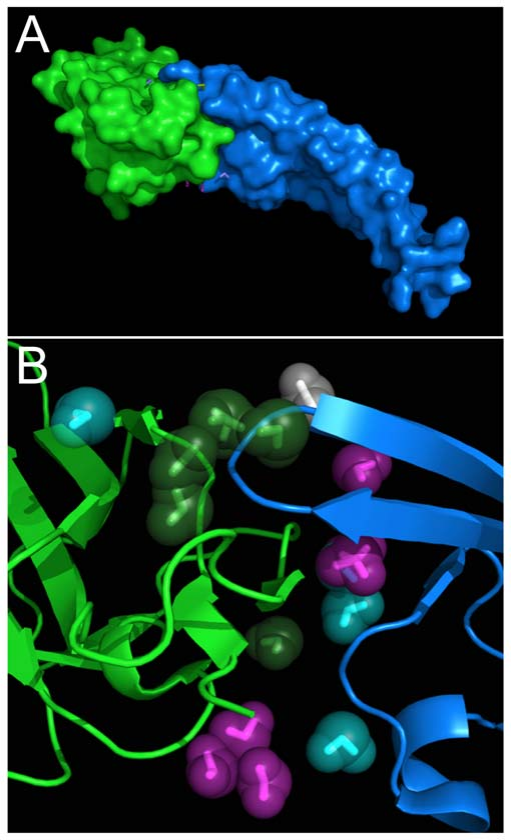
Molecular model of human TGFβ Type II receptor extracellular domain (hβIIR)-TGF β3 (hβ3) complex (1ktz). (A) Connolly-type surface representation with blue for hβIIR and green for hβ3. (B) Interface region; water molecules colored blue are Relevant (≥ 0.25) with respect to hβIIR, green with respect to hβ3, magenta with respect to both hβIIR and hβ3, and white with respect to neither (see Table 2).

We applied the Relevance algorithm to the set of water molecules at protein-protein interfaces to understand their roles in these complexes. The water set for each complex was comprised of all water molecules that were within 4.0 Å of atoms in both proteins. This set, from 179 proteins, was comprised of 4741 unique water molecules, with between 1 and 69 waters (average 27) at the protein-protein interfaces. The orientation of each water molecule was exhaustively optimized [58]. Rodier *et al.* [19] reported 20 per interface in their study of 46 protein-protein complexes. Figure 1 illustrates the set of 16 unique water molecules for the human placental RNase inhibitor (hRI)- human angiogenin (hAng) complex (PDB 1a4y, 2.00 Å) [83], while Figure 2 displays the same (17 unique waters) for the human TGFβ Type II receptor extracellular domain (hβIIR)-TGF β3 complex (PDB 1ktz, 2.15 Å) [86]. The training and derivation of the Relevance metric specified that Relevance ≥ 0.5 corresponds to a water molecule that is conserved and largely static within a ligand binding pocket [50]. We believe that this same Relevance score would also identify a water conserved at a protein-protein interface, and of the 4741 waters in this study, 37% (1741) have total Relevance ≥ 0.5.

More interesting are the evaluations of Relevance with respect to the partner proteins of the complexes. Applying this original definition of Relevance (≥ 0.5 for each protein) identifies only 43 waters (<1%) as bridging. Rodier *et al*. reported that 30% of waters at protein-protein interfaces are bridging, and while their definition of interaction is loose – the water must only be within 3.5 Å of a polar (N, O, S) protein atom to be counted as bridging [19] – we propose that using an intermediate value of Relevance, by halving it to 0.25, to flag association (or Relevance) with respect to a single protein, makes pragmatic sense. With this definition, the Rank, HINT score and Relevance for each were calculated with respect to each protein and in total. These data for 1a4y are listed in Table 1 and listed for 1ktz in Table 2. (Table S2 lists these data for all proteins in the study.)

**Table 1:** Water metrics for human placental RNase inhibitor (hRI)- human angiogenin (hAng) complex (PDB 1a4y, 2.00 Å).

	With hRI:			With hAng:							
Water name	Rank	HINT score	Relevance	Rank	HINT score	Relevance	Total Rank	Total HINT score	Total Relevance	Relevance (≥0.25) w/respect to:	SASA (Å^2^)
HOH1	1.29	409	0.566	2.13	−96	0.205	3.41	313	0.778	hRI	2
HOH2	3.67	−64	0.481	1.18	70	0.333	4.85	6	0.640	Both	8
HOH19	3.51	−26	0.495	1.24	92	0.360	4.74	66	0.687	Both	7
HOH25	3.72	−25	0.529	1.31	68	0.347	5.03	44	0.682	Both	4
HOH52	2.34	358	0.687	1.09	−174	−0.137	3.43	184	0.727	hRI	3
HOH54	3.62	111	0.639	1.25	21	0.295	4.87	132	0.772	Both	10
HOH56	1.05	335	0.419	0.95	30	0.264	2.00	365	0.678	Both	54
HOH59	0.00	−35	−0.039	2.21	−236	−0.280	2.21	−271	−0.362	Neither	2
HOH60	3.78	316	0.822	1.46	−40	0.230	5.24	275	0.924	hRI	18
HOH61	2.30	271	0.627	2.60	141	0.563	4.90	412	0.948	Both	13
HOH68	0.98	80	0.305	1.03	24	0.273	2.01	105	0.441	Both	41
HOH70	1.05	−90	0.186	2.24	134	0.508	3.29	44	0.534	hAng	13
HOH71	0.72	−7	0.196	0.00	−255	−0.299	0.72	−262	−0.342	Neither	11
HOH72	0.89	−39	0.201	1.05	−321	−0.487	1.94	−360	−0.586	Neither	6
HOH73	0.91	22	0.251	1.12	62	0.315	2.03	84	0.418	Both	17
HOH74	1.32	−197	−0.191	2.38	105	0.490	3.70	−92	0.459	hAng	1

**Table 2:** Water metrics for human TGFβ Type II receptor extracellular domain (hβIIR)-TGF β3 (hβ3) complex (PDB 1ktz, 2.15 Å).

	With hβIIR:			With hβ3:							
Water name	Rank	HINT score	Relevance	Rank	HINT score	Relevance	Total Rank	Total HINT score	Total Relevance	Relevance (≥0.25) w/respect to:	SASA (Å^2^)
HOH3	2.47	22	0.413	2.51	−128	−0.036	4.98	−107	0.540	hβIIR	0
HOH4	3.50	14	0.533	0.00	−213	−0.191	3.50	−199	−0.193	hβIIR	21
HOH8	3.66	232	0.784	1.05	−123	−0.024	4.70	110	0.725	hβIIR	8
HOH9	1.10	150	0.357	1.06	−176	−0.142	2.16	−26	0.306	hβIIR	33
HOH11	1.37	106	0.392	2.52	283	0.660	3.89	388	0.861	Both	19
HOH17	0.97	2	0.246	0.00	−200	−0.162	0.97	−198	−0.193	Neither	44
HOH23	2.24	−126	−0.031	1.17	232	0.399	3.41	106	0.607	hβ3	12
HOH24	1.18	205	0.383	1.14	31	0.296	2.32	236	0.607	Both	20
HOH34	2.52	−147	-0.078	1.10	176	0.359	3.63	29	0.564	hβ3	18
HOH54	1.10	131	0.354	0.98	18	0.260	2.08	149	0.506	Both	58
HOH72	0.00	−75	−0.040	3.39	335	0.786	3.39	261	0.756	hβ3	14
HOH74	0.00	−105	−0.040	3.59	362	0.819	3.59	257	0.780	hβ3	1
HOH79	4.06	−16	0.571	1.57	33	0.325	5.62	17	0.683	Both	1
HOH114	0.00	−7	−0.039	1.96	307	0.629	1.96	300	0.623	hβ3	40
HOH115	0.00	−100	−0.040	1.92	366	0.671	1.92	266	0.589	hβ3	15
HOH153	1.16	138	0.372	1.18	296	0.450	2.34	434	0.731	Both	34
HOH161	1.00	47	0.285	1.12	35	0.296	2.12	82	0.429	Both	36

**Figure 3:**
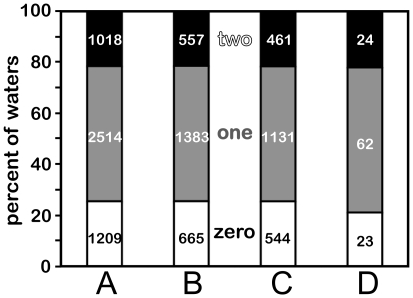
Relative fractions of waters with Relevance to neither (white), one (gray) and both (black) proteins for. (A) full data set of 4741 waters from 179 protein X-ray structures of resolutions ≤ 2.3 Å; (B) reduced data set of 2605 waters from 87 structures of resolutions ≤ 1.90 Å; (C) reduced data set of 2136 waters from 92 protein structures of resolutions between 1.91 Å and 2.3 Å; and (D) 109 waters from 16 structures with resolutions between 2.4 Å and 3.5 Å.

**Figure 4:**
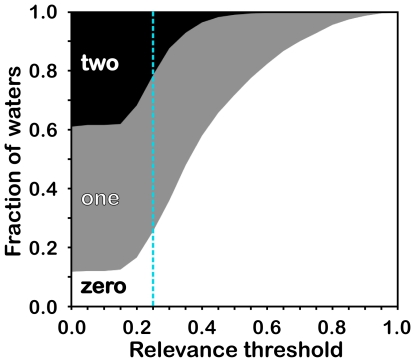
Selection of Relevance threshold. Fraction of waters Relevant to neither (white), one (gray) and both (black) proteins. Relevance was previously trained [50] so that waters having total values 0.50 or greater with respect to all other molecules are conserved; 0.25 (blue line) is the corresponding Relevance with respect to one molecule (protein). Values less than 0.25 are thus not statistically meaningful, while the rapid decrease in the number of bridging waters for thresholds between 0.15 and 0.40 argues for a low threshold. Thus, the selected 0.25 threshold meets both criteria.

Only 21% (1018) of the interface waters have Relevance ≥ 0.25 with respect to *both* proteins, 53% (2514) have Relevance ≥ 0.25 with one member of the protein pair and 26% (1209) are not Relevant with respect to either (see Figure 3A). This suggests that one-fifth of the waters found at a protein-protein interface are truly bridging, while one-fourth are merely trapped at the interface. More than half of the waters are strongly associated with one protein, and while they provide steric constraints for the protein-protein association, they do not provide significant favorable energetic contributions to the association. This is an important distinction, as these waters still likely influence the association in more subtle ways (*vide infra*). While the choice of 0.25 as a threshold to determine the Relevance/non-Relevance of a water molecule with respect to a single protein in a protein-protein complex is somewhat arbitrary (see Figure 4), values smaller than 0.25 both indicate a paucity of potential favorable interactions arguing against the water's conservation and are not consistent with the training of the Relevance metric, while values larger than 0.25 would suggest even fewer bridging waters than reported by Rodier *et al.* [19].

We used a data set comprised of protein X-ray crystal structures with resolutions better than 2.30 Å to construct a representative set of high-quality water molecules. The number of water molecules located and placed by crystallographers during refinement has been shown to be dependent on the resolution of the reflection data [87], [88]. We thus investigated whether, given the categories of waters we define here, there is a resolution-dependence in the relative ratios of water molecules Relevant to zero, one or two proteins. The hypothesis is that at poorer resolutions fewer non-Relevant water molecules would be located and placed in the electron density – presumably because they would be less ordered or conserved – and that the fraction of non-Relevant waters would decrease. However, the relative ratios of water molecules above and below the average resolution for this data set (1.90 Å) are precisely the same (Figure 3B and 3C, respectively). Calculations performed for waters in a second small data set of 16 poorer resolution complexes (2.4–3.5 Å, see Figure 3D and Table S3), where 109 water molecules were located at the interfaces, revealed essentially the same fractions: 23 waters relevant to zero (21%), 62 waters relevant to one (57%) and 24 waters Relevant to two (22%). Crystallographic waters are seldom located in X-ray structures with resolutions poorer than 3.5 Å, and water placements from structures with resolutions between 2.5 and 3.5 Å may be considered somewhat unreliable. Assuming that all of these low-resolution waters are not crystallographic mistakes or artifacts [10], these data pose an interesting question: can water molecules without a stabilizing role at an interface be “conserved”?

### Residue Preferences for Interface H_2_O

Given the three general categories of interface waters we have described, the preferences these water molecules show for the types of amino acid residues within the interfaces were examined. First, for all interface waters, the preferences are tabulated by interaction counts (Table 3). As expected, the more polar residues, in particular Asp (11.9%) and Glu (11.3%), appear most often in interactions involving water at protein-protein interfaces. Cys (0.7%) is most rarely found. However, the aliphatic hydrophobic residues (Ala, Gly, Ile, Leu, Pro and Val) are surprisingly prevalent with 4.5 – 7.8% frequency, notably more so than His, Met, Phe or Trp (<2.3%). Glaser *et al.* [89] reported contact counts (within certain C_ß_- C_ß_ cutoffs) at protein-protein interfaces that are generally similar except that Asp and Glu appear more than twice as frequently and Cys and Phe appear less than half as frequently in our water-mediated observations. Likewise, our results are in qualitative agreement with the report of Teyra and Pisabarro for “dual” and “wet” interactions between residues at protein-protein interfaces [27]. In their nomenclature, dual refers to an interaction that has both direct residue-residue interaction and water-mediated interaction, while wet refers to an interaction that is only water-mediated. When examining these preferences for waters having productive and Relevant interactions with both proteins, the fraction arising from residue sidechains carrying hydrogen bond donors or acceptors is enhanced (Arg, 9.6%; Asp, 18.4%; Glu, 17.0%) relative to those arising from hydrophobic sidechains. For the cases where the waters are Relevant with respect to neither protein, the opposite is true – as expected (Ala, 11.0%; Ile, 6.9%; Leu, 13.0%; Pro, 9.9%; Thr, 8.8%; Val, 9.1%). However, as described by Teyra and Pisabarro [27], water interactions with non-polar residues may in some cases be energetically favorable from interactions involving backbone atoms (*vide infra*).

**Table 3:** Frequencies and HINT scores of water molecules at protein-protein interfaces with respect to interacting amino acid residues.

	All Waters			Waters Relevant to 0			Waters Relevant to 1			Waters Relevant to 2		
Residue Type	Wtd. Count^a^	Average HINT score^b^		Wtd. Count^a^	Average HINT score^b^		Wtd. Count^a^	Average HINT score^b^		Wtd. Count^a^	Average HINT score^b^	
		For All	For Type		For All	For Type		For All	For Type		For All	For Type
Ala	320	−28.51	−422.3	133	−48.90	−444.7	158	−27.42	−436.8	29	−6.95	−242.5
Arg	279	15.04	255.9	42	4.32	124.3	139	12.99	235.6	98	32.83	341.3
Asn	229	9.96	205.9	37	2.20	71.6	125	12.07	242.0	67	13.97	213.3
Asp	564	63.32	532.7	49	7.12	176.6	328	72.88	558.2	187	106.60	580.7
Cys	32	0.29	42.7	7	0.36	65.4	17	0.11	16.2	8	0.64	79.1
Gln	201	6.66	156.9	34	0.57	20.2	120	8.14	171.0	48	10.26	219.8
Glu	535	54.91	486.8	50	4.73	114.2	312	64.03	515.6	173	92.11	542.9
Gly	212	−9.89	−221.1	53	−11.15	−254.3	113	−10.87	−241.3	46	−5.98	−132.7
His	75	2.71	170.8	13	1.36	130.6	42	2.38	142.6	21	5.15	251.9
Ile	212	−21.49	−481.7	84	−36.71	−529.1	107	−20.31	−478.3	21	−6.31	−308.1
Leu	369	−35.54	−456.8	157	−62.71	−484.2	179	−33.34	−467.7	33	−8.65	−267.3
Lys	220	0.86	18.5	57	−5.16	−110.4	110	0.67	15.3	54	8.49	160.6
Met	107	−9.63	−425.2	41	−16.30	−483.4	54	−9.63	−448.8	13	−1.68	−135.7
Phe	75	1.25	79.7	16	0.16	12.2	40	1.83	113.9	18	1.14	63.3
Pro	278	−22.61	−385.3	120	−36.76	−369.9	137	−22.18	−408.0	21	−6.86	−327.3
Ser	260	−5.69	−103.8	69	−9.28	−162.8	137	−6.12	−112.3	54	−0.37	−7.0
Thr	307	−19.10	−294.5	106	−34.25	−390.5	158	−18.75	−298.6	44	−1.93	−45.2
Trp	52	1.62	148.1	10	0.55	67.2	26	1.48	143.9	16	3.21	205.0
Tyr	147	6.13	198.0	23	1.64	87.3	81	6.92	214.3	43	9.52	225.6
Val	267	−27.61	−489.7	110	−45.96	−503.8	131	−26.26	−503.2	26	−9.12	−360.7

Notes:

a Weighted count is calculated as ∑_n_ { |A_i_|/∑_i_ |A_i_| }, where A_i_ are the interaction HINT scores by residue type (i) interacting with water n;

b HINT scores are averaged two ways: first, over all waters in set or Relevance subset, second, by frequency (weighted count) of that residue type in set or Relevance subset.

While optimizing and scoring, each water molecule in the present report was treated as a small ligand in a site defined by neighboring residues. The average HINT score for the waters in the entire data set is -17 (ΔG ∼ +0.03 kcal mol^−1^); thus, the average interaction of a water with only one of its neighboring proteins would be half of that value, i.e., essentially negligible. Table 3 lists the HINT score values for each of the twenty amino acid types, first by averaging over all waters in the data set, and second by averaging over all waters interacting (by weighted count) with that residue type. The first average, over all waters, reveals the reason for the near zero value for the average interaction energy of an interfacial water with its environment: there is a complex mix of favorable and unfavorable interactions with water, depending on the residue type. The latter average, weighted instead by the frequency of that particular water-residue interaction, represents the score that would be expected if a water interacted with only that residue and thus reveals the specific benefits of interacting with some residue types, e.g., Asp (−1.03 kcal mol^−1^), Glu (−0.95 kcal mol^−1^), or Arg (−0.50 kcal mol^−1^), vs. the cost of interacting with others, e.g., Pro (+0.75 kcal mol^−1^), Ala (+0.82 kcal mol^−1^), Met (+0.83 kcal mol^−1^), Leu (+0.89 kcal mol^−1^), Ile (+0.94 kcal mol^−1^) or Val (+0.95 kcal mol^−1^). The biggest surprise here is that Lys, while responsible for 4.6% of interactions with interface waters has, on average, a minimal contribution to the water score. This is partly because Lys, if NZ is protonated as expected, is only a hydrogen bond donor and is unable to accept from water, but also, the long hydrophobic polymethylene sidechain of Lys may be interacting unfavorably with some water molecules compared with the other “basic” residue Arg that has multiple polar atoms and can act as an acceptor through its sidechain π system. Also, Lys with its flexible sidechain is more likely to be disordered and its atomic coordinates are thus less certain. Furthermore, Jones and Thornton [90] noted that Lys frequency is depleted at protein-protein interfaces relative to protein surfaces.

**Figure 5:**
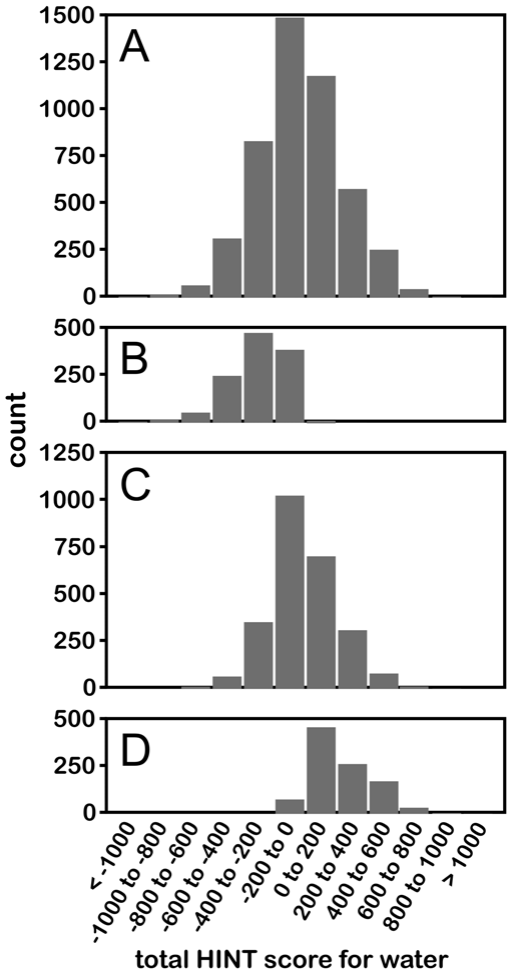
Histograms illustrating distribution of HINT scores for water molecules. (A) All waters in data set; (B) water molecules with Relevance to neither protein; (C) waters with Relevance to one protein; (D) waters with Relevance to both proteins. Note that 500 HINT score units is approximately −1.0 kcal mol^−1^.

The differences in interactions between water molecules Relevant to zero, one and two proteins are instructive. First, these waters have average HINT scores of -284 (+0.55 kcal mol^−1^), 9 (−0.02 kcal mol^−1^) and 236 (−0.46 kcal mol^−1^), respectively (see Figure 5). Also, as calculated with the averages over all waters that are Relevant to zero, one or two proteins (Table 3), the interactions are dominated by Ala, Ile, Leu, Pro, Thr and Val (generally unfavorable, with negative HINT scores) for the waters Relevant to neither protein, and dominated by favorable interactions with Arg, Asp and Glu for the waters Relevant to both proteins. The fact that Thr “acts” more hydrophobic is probably because its methyl group partially shields the hydroxyl's ability to engage in hydrogen bonding.

### Sidechain and Backbone Preferences for Interface water

Teyra and Pisabarro [27] showed that a significant fraction of interface water molecules appear to be interacting with backbone atoms on one of both of the proteins. Rodier *et al*. calculate that 12% of water interactions at protein-protein interfaces are with backbone NH and 33% with CO [19]. Our analysis of backbone and sidechain interactions reveals interesting details: the average interaction score for a water with a backbone atom [C, O, (OXT), CA, HA, N, HN, (HN2, HN3)] is favorable (57, −0.11 kcal mol^−1^), while on average the interaction with sidechain atoms is unfavorable (−74, +0.14 kcal mol^−1^). Obviously, this can be explained by the ability, although usually shielded by the sidechain, of the backbone to be both a hydrogen bond donor (via NH) and acceptor (via O). Table 4 lists the weighted counts and average scores for backbone and sidechain interactions with water by residue type. Our calculations of weighted interaction counts, which are based on HINT scores of H-bond optimized structures and not simple distance metrics, suggest (Table 4) that only 21.5% of the water-protein interactions involve backbone atoms, and that the remaining 78.5% arise from sidechain atoms. Thus, while the backbone interactions are mostly favorable, they play a lesser role in describing the protein-protein interface than do the sidechain interactions. The average scores, when weighted by the frequency of interactions for the residue types for either the backbone or sidechain (Table 4), clearly show that the backbone interactions are remarkably consistent and independent of residue identity. These scores represent how strongly a single water would interact with a residue backbone (or sidechain) isolated from all other interactions.

**Table 4:** Frequencies and HINT scores of water molecules at protein-protein interfaces with respect to backbones and sidechains of interacting amino acid residues.

	All	Interacting with backbone			Interacting with sidechain		
Residue Type	Wtd. Count^a^	Wtd. Count^b^	Average HINT score^c^		Wtd. Count^b^	Average HINT score^c^	
			For All	For Type		For All	For Type
Ala	320	63	5.39	403.2	257	−33.90	−625.9
Arg	279	54	4.27	376.2	225	10.76	227.1
Asn	229	54	4.44	388.6	175	5.52	149.4
Asp	564	65	5.29	388.4	499	58.03	551.3
Cys	32	18	1.47	390.3	14	−1.18	−393.1
Gln	201	39	3.27	395.7	162	3.39	99.2
Glu	535	51	4.13	384.7	484	50.79	497.5
Gly^d^	212	212	−9.89	−221.1	0	0.00	−
His	75	20	1.71	410.0	55	1.00	85.5
Ile	212	29	2.60	430.5	183	−24.09	−624.3
Leu	369	53	4.78	429.2	316	−40.32	−604.9
Lys	220	45	3.80	398.7	175	−2.94	−79.7
Met	107	20	1.82	440.2	88	−11.44	−618.2
Phe	75	33	2.81	409.7	42	−1.56	−175.3
Pro	278	45	2.81	298.7	234	−25.43	−516.0
Ser	260	71	5.39	358.2	189	−11.09	−278.5
Thr	307	59	5.03	407.0	249	−24.13	−459.7
Trp	52	20	1.84	443.6	32	−0.22	−32.8
Tyr	147	36	2.90	380.2	111	3.23	138.4
Val	267	36	3.33	439.1	231	−30.95	−634.3

Notes:

a Same as Table 3;

b Weighted count is calculated as ∑_n_ { |A_i_|/∑_i_ |A_i_| }, where A_i_ are the interaction HINT scores for the backbone or sidechain by residue type (i) interacting with water n;

c HINT scores are averaged two ways: first, over all waters in set or Relevance subset, second, by frequency of the backbone or sidechain contribution (weighted count) of that residue type in set or Relevance subset;

d For Gly (and all other residues) the CA atom is considered part of the backbone, thus Gly has no sidechain.

**Figure 6:**
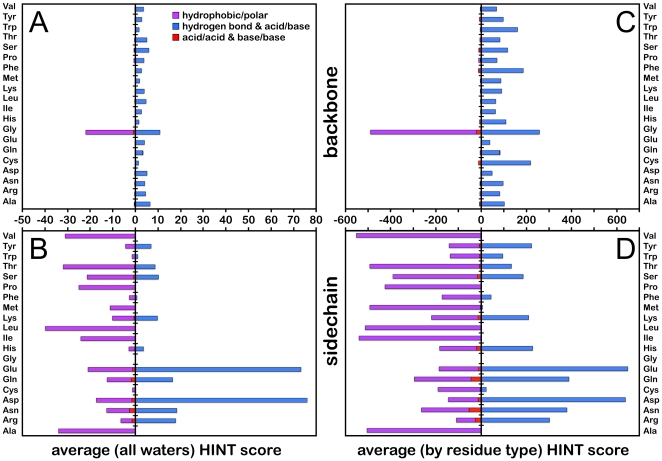
Average HINT interaction scores for waters at protein-protein interfaces. (A) Scores averaged over all water molecules for interactions with protein backbone atoms; (B) scores averaged over all water molecules for interactions with protein sidechain atoms; (C) scores normalized by weighted count of residue types (Table 4) with protein backbone atoms; and (D) scores normalized by weighted count of residue types with protein sidechain atoms.

However, the total score only tells part of the story and obscures the operational details on how the waters actually interact with the proteins. Figure 6 displays (A) backbone and (B) sidechain interactions by residue type and interaction class, averaged over all water molecules in the data set. In particular, favorable polar (hydrogen bonds and acid/base) interactions are plotted as positive contributions, while unfavorable polar (acid/acid and base/base) and unfavorable hydrophobic (i.e., interacting with polar) interactions are plotted as negative contributions. This analysis indicates (Figure 6A) that other than for the more exposed Gly, which does not have a side chain, the backbone interactions with water are dominated by favorable polar interactions and expectedly largely independent of residue type. In contrast, the interactions with sidechains are quite obviously dependent on residue type and vary from highly unfavorable (for hydrophobic residues like Ala and Leu) to highly favorable (for the carboxylate residues Asp and Glu). Figure 6C (backbone) and 6D (sidechain) illustrates the average scores for each residue type, i.e., weighted by the number of water interactions of those types in the data set. These charts emphasize the similar role of backbone interactions for nearly all residue types, excluding Gly. This contribution is largely independent of the Relevance of the water involved, increasing only modestly from 49 (−0.10 kcal mol^−1^) to 57 (−0.11 kcal mol^−1^) and 67 (−0.13 kcal mol^−1^) for waters Relevant to zero, one and both proteins, respectively. At the same time, the average sidechain interaction scores respond dramatically, increasing from −333 (+0.65 kcal mol^−1^) to −48 (−0.09 kcal mol^−1^) and 169 (−0.33 kcal mol^−1^).

### Residue-Pair Preferences for Interface H_2_O

**Figure 7:**
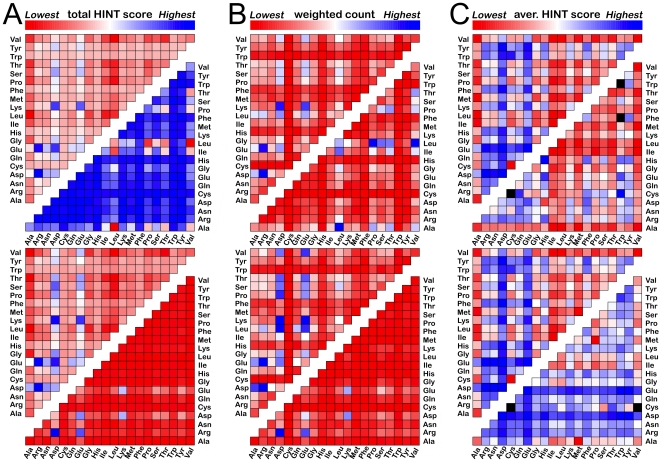
Color heat maps depicting Res_1_-H_2_O-Res_2_ interactions for water molecules found at protein-protein interfaces. All maps are linearly scaled over the maximum range of values for that data set. (A) Total HINT score between waters and Res_1_/Res_2_: upper left – all waters in data set (minimum score -71,358, maximum score 114,632); upper right – waters in set with Relevance to neither protein (minimum -41,868, maximum 3,685); lower left – waters in set with Relevance to one protein (minimum -26,470, maximum 50,220); lower right – waters in set with Relevance to both proteins (minimum -3,534, maximum 60,727). (B) Weighted count of Res_1_/Res_2_ with water interactions: upper left – all waters in data set (minimum count 0.1, maximum count 242.7); upper right – waters in set with Relevance to neither protein (minimum 0.0, maximum 74.0); lower left – waters in set with Relevance to one protein (minimum 0.1, maximum 113.3); lower right – waters in set with Relevance to both proteins (minimum 0.0, maximum 114.5). (C) Average HINT score (normalized by weighted count) between waters and Res_1_/Res_2_: upper left – all waters in data set (minimum average score -601.6, maximum average score 540.5); upper right – waters in set with Relevance to neither protein (minimum -624.3, maximum 483.0); lower left – waters in set with Relevance to one protein (minimum -633.7, maximum 499.7); lower right – waters in set with Relevance to both proteins (minimum -875.1, maximum 680.9). Cells colored black represent cases where the weighted count was zero, and the HINT score normalization yields an undefined value.

By definition, waters found at the interface should interact with residues on both proteins. Our floor value for interactions of |10| HINT score units, or about |0.02| kcal mol^−1^, excludes a small number of waters (<1.5%) from having any recorded interaction with one (or in rare cases both) of the proteins. As shown above, in Table 3, there is a residue identity preference for water-mediated interactions at protein-protein interfaces and this differs depending on the role the water plays at the interface. More specifically, we show here that there are distinct residue identity preferences for mediated residue pairs. Consider first the total gross sum of HINT scores for each pair of amino acid residue types as graphically illustrated with color heat maps in Figure 7A for all waters, and those Relevant to neither, one and both proteins. This depiction combines both the strength of interaction and frequency of interaction for the residue pairs. Overall, in the upper left of Figure 7A, the most energetically favorable pairs for interface water involve one of the polar residues, especially the hydrogen bond acceptors Asp and Glu. These can partner with each other – intriguingly Asp-H_2_O-Glu scores higher than Asp-H_2_O-Asp or Glu-H_2_O-Glu – or partner extensively with the hydrogen bond donor or amphiprotic residues (Arg, Asn, Gln, Lys, Ser, Thr, Tyr), but not significantly with His or Trp. The most unfavorable pairings involve the most hydrophobic and aliphatic residues Ala, Ile, Leu, Pro and Val. The intermediate effect of Phe may be due to its aromatic ring being a potential hydrogen bond acceptor. The scores for waters with Relevance to neither protein (Figure 7A, upper right) are dominated by strongly unfavorable interactions with hydrophobic residues, especially Leu and Ile, while the scores for waters with Relevance to both proteins (Figure 7A, lower right) are most favorable for interactions involving Asp and Glu, particularly when partnered with Arg.

Frequencies weighted as described in Materials and Methods are set out in Figure 7B. Overall (upper left), water-mediated interactions involving Asp, Glu, Lys, Arg and surprisingly Leu are clearly dominant while those involving Cys, His, Phe and Trp are most infrequent. Waters not relevant to either protein (Figure 7B, upper right) generally interact with hydrophobic residues. For waters relevant to both proteins (Figure 7B, lower right), the most frequent pairs are Asp and Glu with Arg and Lys. Also, Asp and Glu are found fairly frequently in water-bridged interactions with Asn, Gln, Ser and Tyr. Note that the color pattern here is strikingly similar to that of the overall score for the doubly relevant case (Figure 7A, lower right), which indicates that frequency of pair interactions is a key factor. Finally (Figure 7C), the score normalized by weighted frequency reveals the relative average energetic importance of each interaction pair ranging between -602 score units (+1.17 kcal mol^−1^) and 541 score units (−1.05 kcal mol^−1^).

### Residue-Pair Roles in Water Interactions

**Figure 8:**
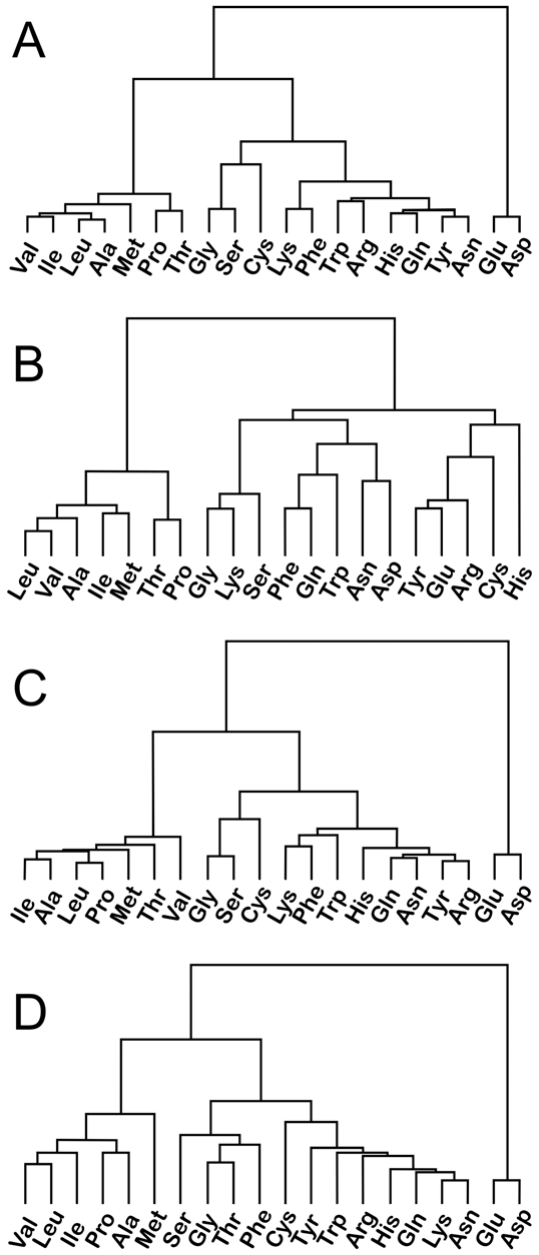
Dendograms indicating clustering of residues with respect to average HINT score (normalized by weighted count) in Res_1_-H_2_O-Res_2_ interactions. (A) for all waters; (B) for waters with Relevance to neither protein; (C) for waters with Relevance to one protein; and (D) for waters with Relevance to both proteins.

Cluster analysis of the matrices behind the heat maps of Figure 7D provide additional insight into the roles that residues play in interacting with waters. Figure 8 sets out dendograms of average HINT score for: (A) all waters, (B) waters not Relevant to either protein, (C) waters Relevant to one protein and (D) waters Relevant to both proteins. The Relevant to zero case is most different from the others. Generally, the most hydrophobic aliphatic residues (Ala, Ile, Leu, Met, Pro, Thr and Val) are clustered together with Thr (except for the case of Relevant to both, Figure 8D). At the opposite extreme, Asp and Glu are clustered, save the Relevant to zero case, far from all other clusters. The ability of water to be equally proficient as both a hydrogen bond donor and an acceptor somewhat blurs the distinction between residues that are formally acids or bases when they interact with it. The remaining residue types divide into two clusters with somewhat variable membership. Because the aromatic ring of Phe can act as a hydrogen bond acceptor, it clusters with an eclectic group of residues: Ser, Gly, Gln, Lys, Trp and/or Thr, but surprisingly not Tyr. For waters Relevant to neither protein, there are typically few favorable interactions, regardless of the character of the residues interacting with the water. The patterns in the associated dendogram (Figure 8B), other than the large distance separating the hydrophobic residues from the polar residues, are difficult to discern; here, Asp and Glu are not clustered together. A likely determinant defining these clusters may involve residue size.

## Discussion

This analysis of 4741 water molecules at 179 protein-protein interfaces has revealed new information about the various roles that water can play at interfaces. Our analysis was anchored by the HINT free energy forcefield and the Relevance metric. The former characterizes the types and qualities of interactions between the interface waters and proteins, while the latter is a simple parameter that was previously shown to identify water molecules conserved/non-conserved in ligand binding sites [50]. Relevance was shown in the present report to be a useful classifier for identifying the roles and partner proteins and residues for interfacial waters.

Previous studies of water in the interface between interacting proteins have generally relied solely on interatomic distances in non-protonated crystallographic models to mark interactions between waters and proteins. This approach, however, often poorly represents the complex and subtle energetics and geometric preferences of hydrogen bonding. Thus, we performed this study with all atoms after exhaustive optimization of all water orientations [58] to surmount local minima in our models. The hydropathic minimization procedure rewards favorable polar interactions, i.e., hydrogen bonds and acid/base, and penalizes unfavorable polar, i.e., acid/acid and base/base, and hydrophobic-polar interactions, by maximizing the HINT score for the water in its environment.

### Waters Relevant to Multiple Proteins: How important is the energetic contribution of water to protein-protein associations?

This is an important question since most protein-protein docking utilities ignore the actual (and potential) presence of water at putative interfaces. Unfortunately, it is difficult to determine *de novo* which water molecules are or will be energetically important. Only 59 (33%) of the protein-protein complexes have an overall favorable water contribution considering all interface waters, but 145 (81%) have a favorable contribution from waters Relevant to one/both proteins and nearly all, 173 (97%), have a favorable contribution from waters that are Relevant to both (the other 6 protein pairs have no waters of this class). The average scores are: -2072 (+4.02 kcal mol^−1^), -84 (+0.16 kcal mol^−1^) and 1297 (−2.52 kcal mol^−1^) for the water sets at these interfaces Relevant to 0, 1 and 2 proteins, respectively. While each water at each protein-protein interface should be evaluated for its own specific environment and role, the overall analysis shows that the total water contribution can be quite important: ranging up to 5845 (−11.35 kcal mol^−1^) per protein pair for the water sets Relevant to both proteins and presumably “bridging”. Also, the Relevance-based classification scheme we have proposed certainly has merit for facilely selecting waters that should be considered in modeling protein-protein complexes.

The energetic role of bridging water molecules at interfaces is clear and well understood, although difficult to experimentally quantify [22], [91]-[93]. Reichmann *et al*. [91] performed double mutant cycle analysis on eight residue pairs (all with SASA < 10 Å^2^) that appeared to be bridged by waters at the TEM1/BLIP (1jtg) interface; only six of the eight pairs are truly bridged by water (residue-residue distance > 3.8 Å), yielding an average ΔΔG_KA_ [91] for these water-mediated hydrogen bonds of -0.003 kcal mol^−1^, i.e., essentially having an energetically neutral effect on interface stability much as shown above (+0.03 kcal mol^−1^) for an average interface water in our analysis. Only four waters support these six pairs because two of the waters interact with more than one residue on one of the partner proteins (one highly Relevant to both proteins and the other Relevant to only BLIP), and it is thus impossible to isolate the specific energetic contribution from experimental double mutant data for these two waters. Of the remaining two waters, our analysis shows that one (HOH72) is Relevant to only TEM1 and the other (HOH111) is not Relevant to either protein, supporting the view that the former is strongly associated with TEM1's Glu104 and weakly associated with BLIP's Ser146, while the latter is only weakly associated with Gln99 and repulsive with respect to Ser128. Even here, interpretation is not straightforward: mutating these residues to Ala may or may not excise the putative bridging waters, just change their environment. In fact, there may even be space for more than one water in some of the double mutant complexes.

**Figure 9:**
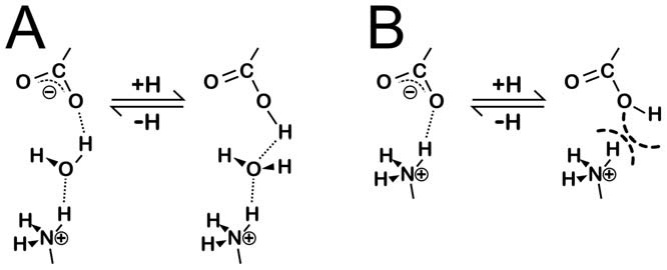
Water as a nano-scale buffer. (A) increasing the pH of the system is compensated by a reorientation of the bridging water molecule; (B) direct unmediated interactions are less able to compensate for changes in pH.

Another, more subtle, role is that bridging waters also serve as nano-scale pH buffers (see Figure 9). By simply re-orienting, individual water molecules can swap between acting as donors and acceptors as necessary to maintain a mediated (wet) interaction and the integrity of the entire interface. In contrast, direct hydrogen-bonded (dry) interactions between proteins may be weakened by changes in pH. Of course, hydrophobic interactions between protein surfaces are largely unaffected by changes in pH. Evidence for this role of waters was given in the cluster dendogram of Figure 8D. Other than the distinct clustering of Asp with Glu and the aliphatic hydrophobic residues with Met, the remaining twelve residues cluster together regardless of their hydrogen bond donor or acceptor character.

### Waters Relevant to One Protein: Is their role purely steric?

The majority of water molecules in this study appeared to be largely associated with one or the other of the interacting proteins. Rodier *et al.* [19] described the interface waters in terms of ordered rings surrounding the joints/interface patches between the two proteins and internal waters at “wet” interface patches. A few other waters may be near, and stabilized by, ligands or other cofactors at the interface. The ring waters are potentially accessible to bulk solvent, which may stabilize them, whereas internal waters are isolated from the solvent. Using a threshold solvent accessible surface area of 5 Å^2^ to define an internal or buried water, Rodier *et al.* estimated that 71% of interface waters in complexes are in these rings [19]. We wanted to see if there is a correlation between Relevance class and SASA. Our algorithm (see Materials and Methods) for calculating SASA found a similar result to that of Rodier *et al*. for all waters in the data set – 64% of the waters have SASA > 5 Å^2^. However, this definition is somewhat arbitrary: 50% have SASA > 10 Å^2^ and only 42% have SASA > 15 Å^2^. Note that, because all water molecules in the interface set are used in defining the non-available volume, our algorithm calculates low accessibility for water molecules clustered together in isolated non-accessible pockets. Also, the presence of void spaces, including those from (deleted) ligands or cofactors, near waters in these pockets would overestimate their solvent accessibility. The average SASAs for Relevance 0, 1 and 2 waters are 16.7, 14.2 and 13.9 Å^2^, respectively.

**Figure 10:**
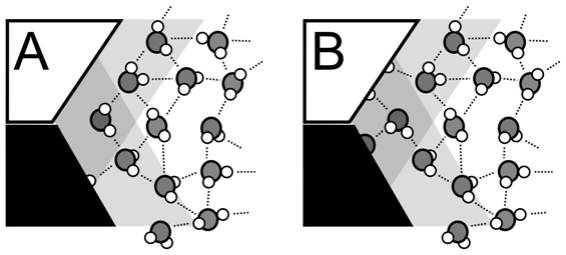
Motifs for water molecules in ring region (overlap of shaded zones). (A) water without interactions with either protein may be stabilized *in situ* by other water molecules; (B) under favorable conditions water may bridge between proteins and be Relevant with respect to both.

Table 5 sets out the counts of buried water molecules for the Relevance 0, 1 and 2 sets. In addition to the lower number of buried waters found for Relevance 0 cases (discussed below) there are, for buried definitions of SASA ≤ 5 Å^2^ and SASA ≤ 10 Å^2^, somewhat *higher* fractions of buried waters with Relevance 1 than with Relevance 2. As stated above, the ring waters may be considered part of the bulk water network that just happens to reach into an interface neck, which would stabilize waters that have little or no viable interaction with protein (see Figure 10A). At the opposite extreme, ring waters definitively bridging the two proteins (Figure 10B), and Relevant to both, are likely to be a common motif. Although it is difficult to ascertain the role of ring waters Relevant to only one protein, they *are* somewhat less common. Overall, these waters likely have mostly a steric effect of shaping the surfaces of the individual proteins.

**Table 5:** Summary of solvent accessible surface area calculations for waters at protein-protein interfaces.

	Number (Fraction) of Waters Buried at Interface			
Buried H_2_O Definition	All Waters	Waters Relevant to 0	Waters Relevant to 1	Waters Relevant to 2
SASA ≤ 5 Å^2^	1716 (0.362)	345 (0.285)	1006 (0.400)	365 (0.359)
SASA ≤ 10 Å^2^	2350 (0.496)	503 (0.416)	1340 (0.533)	507 (0.498)
SASA ≤ 15 Å^2^	2753 (0.581)	635 (0.525)	1499 (0.596)	619 (0.608)

### Waters not Relevant to either protein: Why are there so many waters that are seemingly non-Relevant?

There are a large number of water molecules that do not appear to have a role in structure. A brief survey of moderate-resolution complex structures revealed essentially the same fraction of waters that lacked favorable interactions with their protein pairs as did the much more extensive high-resolution set. These results suggest that this type of water is a conserved phenomenon as only the most ordered water molecules will have interpretable experimental electron density for resolutions poorer than 2.5 Å.

**Figure 11:**
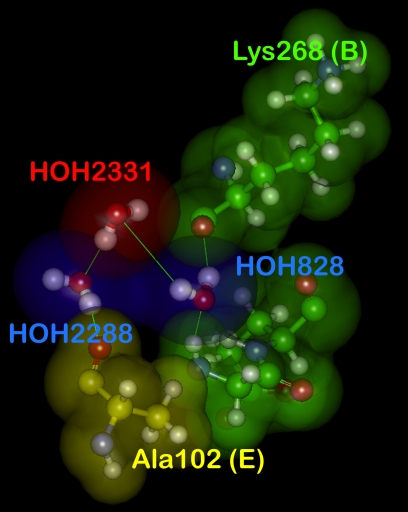
Water in chain of three water molecules. HOH2331 (red) from protein complex 1kxq is Relevant with respect to waters HOH828 and HOH2288 (blue), which are each, in turn, Relevant to the proteins in the complex.

The analysis described above did not attempt to detect water molecules that are involved in water network chains, i.e., waters that are strongly and favorably interacting with two or more other waters that are themselves Relevant to a protein. To investigate this possibility (for an example, see Figure 11), we added the water molecules that were Relevant to one or both proteins to their partners of highest Relevance and examined the remaining (i.e., initially Relevance zero) waters with respect to these “hydrated” protein entities. Only 326 (27%) of the remaining waters were found to have Relevance (≥ 0.25) with one and 30 (2.5%) were found to have Relevance to both hydrated proteins. The latter represent water molecules networked in three-water chains. It is a surprisingly low number, but the Relevance-based definition of networking is fairly stringent, and these waters are already constrained to be within the confines of the interface region while not already interacting favorably with other protein residues. It is therefore unlikely that significant numbers of these water molecules would turn up to be involved in higher order chains.

**Figure 12:**
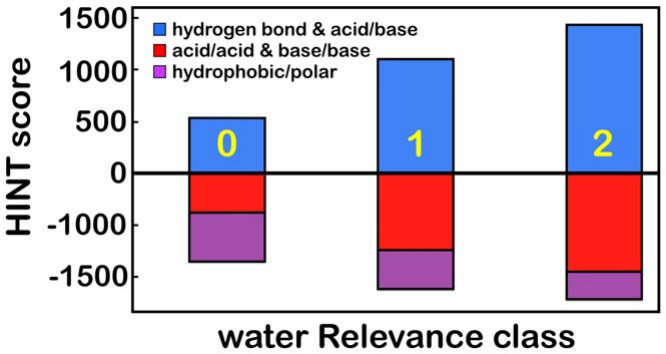
Average interaction type scores for waters with Relevance to zero, one and two proteins.

Table 5 indicates that, while there are fewer buried waters in the set with Relevance to neither protein as compared to the other sets, the difference is not that dramatic. More than one-quarter of water molecules that do not have a favorable interaction with either of the proteins are *well buried* within the interface. As discussed above, Relevance zero waters have overall unfavorable interactions with their partner proteins, which largely arises from interactions with the protein's sidechains. It can be seen in Figure 12 that the dominant unfavorable interaction type for these waters is hydrophobic-polar; the favorable polar interactions shown in Figure 12 are due to interactions with the backbone (see Table 4). In fact, 69% of the Relevance zero water molecules within (i.e., with SASA ≤ 10 Å^2^) the interface are trapped in hydrophobic environments or what we term “hydrophobic bubbles”. This is 7.4% of all waters in the data set.

It would appear that these hydrophobic bubbles represent a conserved motif. One intriguing possibility is that a certain amount of instability is required in protein-protein interfaces to ensure that the associations are dynamic. Meenan *et al.* described the role of some waters found at the 1.77 Å structure of the colicin E9 endonuclease-immunity protein 2 interface as “aggravating” the binding between the two proteins [15]. Sundaralingham and Sekharudu [94] proposed that water may be considered a “lubricant” in dynamic protein folding and interaction. Teyra and Pisabarro [27] classified the complexes in their analysis as “obligate” meaning that the association is permanent as these interfaces were formed concurrent with chain folding and “transient” where the component proteins fold independent of their association [90], [95]. The latter of course includes proteins involved in regulation of biochemical pathways and signal transduction. Similar concentrations (10 vs. 11 water-bridged residues/1000 Å^2^ surface contact area) of waters were found in the two groups [27]. Our primary data set is composed entirely of transient proteins. However, for comparison, we examined a set of 12 homo-dimer, predominantly obligate [96], interfaces containing 546 water molecules (see Table S4) selected as described above. In the obligate set, there were 113 waters (21%) Relevant to neither protein, 302 waters (55%) Relevant to one protein, and 131 (24%) Relevant to both. As would be expected, there are somewhat higher fractions of waters with Relevance to both one and two proteins, and a smaller fraction that are non-Relevant. Perhaps more significantly, 55% of the waters in the homo-dimer set have SASA ≤ 10 Å^2^ and this is independent of Relevance class. It appears that protein-protein interfaces, independent of the longevity of their association, commonly include water molecules that do not have favorable interactions with either protein, although the possibility that some or maybe even many of these waters are incorrectly assigned electron density or other crystallographic artifacts cannot be completely discounted [10].

### Predictions of water roles

The principle of correlated mutations is that interface contacts co-evolve to maintain or enhance biologically important associations [28]-[31]. Using this principle, Samsonov *et al*. recently reported [25] that including solvent matrices in contact predictions [97], [98] of protein-protein interfaces improve these predictions by 20-30%. However, no residue level information was reported. We noted above (see Figure 7B) that the observed frequency of Asp-H_2_O-Glu interactions, in waters Relevant to one or both proteins, is notably higher than Asp-H_2_O-Asp or Glu-H_2_O-Glu interactions. This suggests that water molecules may act as spacers to effectively lengthen Asp sidechains to mimic Glu sidechains. We observed a similar role for Asp+H_2_O in protein/DNA interactions [43], [44]. Whether this is a consequence of correlated mutations is difficult to say, but it is an intriguing possibility.

Water Relevance may be used as a metric to predict the locations of water molecules computationally. We previously described [99] an algorithm for generating water solvent arrays around proteins or in binding pockets that is superficially similar to the GRID algorithm proposed by Goodford [40]. This protocol can easily be adapted to use Relevance-based criteria for water placement; for this purpose it is especially significant that Relevance is calculated independent of (experimentally-determined) crystallographic data like B-factors. However, this present study indicates that the presence of as many as one-in-four energetically unfavorable water molecules is an apparently conserved motif. Their positions and orientations will almost certainly be difficult to predict! Nevertheless, we believe that there are common structural features such as hydrophobic bubbles that may aid in this understanding and in developing algorithms for computationally orienting and locating these waters. At the same time, we propose that these “unfavorable” water molecules may actually have an important biological purpose [56], [94]. It is fair to say that we won't be able to completely model or exploit protein-protein interfaces until we can properly deal with all of the water molecules that are present.

This work represents one small piece of our overarching goal of estimating the energetics of protein-protein associations and being able to *de novo* predict their structure. One additional consideration is adjusting the ionization states of residues involved in dry and wet interactions. For this, we are currently adapting our Computational Titration procedure [100] with a genetic algorithm front end to effectively sample the many millions of potential ionization state ensembles at a large interface. Also, the effects of ligands and cofactors, particularly ions, should be recorded. Finally, these species are not rigid and sidechain flexibility is another important energetic and structural factor.

## Supporting Information

Figure S1 **Algorithm for calculating solvent accessible surface area (SASA).** (A) grid is constructed around water (blue) of interest including atoms from two proteins (green and red); (B) grid boxes fully or partially occupied by atoms (Van der Waals volume) are set as unavailable; (C) a set of spheres (yellow dashed lines) centered (black circles) at a distance r_VdW_ + r_solvent_ from the water of interest are constructed (r_VdW_  =  r_solvent_  =  1.4 Å in this case); (D) if entire volume, i.e., all grid boxes, of one of these spheres is available, then the surface area represented by the center of that sphere (black cube) is solvent accessible. All such areas are summed to obtain the SASA for the water molecule of interest.
(TIFF)

Table S1 **Protein complexes examined in study with interface parameters and water roles.**
(PDF)

Table S2 **Water Rank, HINT score, Relevance and solvent accessible surface area for full data set.**
(PDF)

Table S3 **Water Rank, HINT score, Relevance and solvent accessible surface area for low-resolution data set.**
(PDF)

Table S4 **Water Rank, HINT score, Relevance and solvent accessible surface area for homo-dimer data set.**
(PDF)
