# Supplementary figures and images for: Bound Water at Protein-Protein Interfaces: Partners, Roles and Hydrophobic Bubbles as a Conserved Motif
Source: PLoS One. 2011 Sep 22;6(9):e24712. doi: 10.1371/journal.pone.0024712 (PMC3178540; doi:10.1371/journal.pone.0024712)

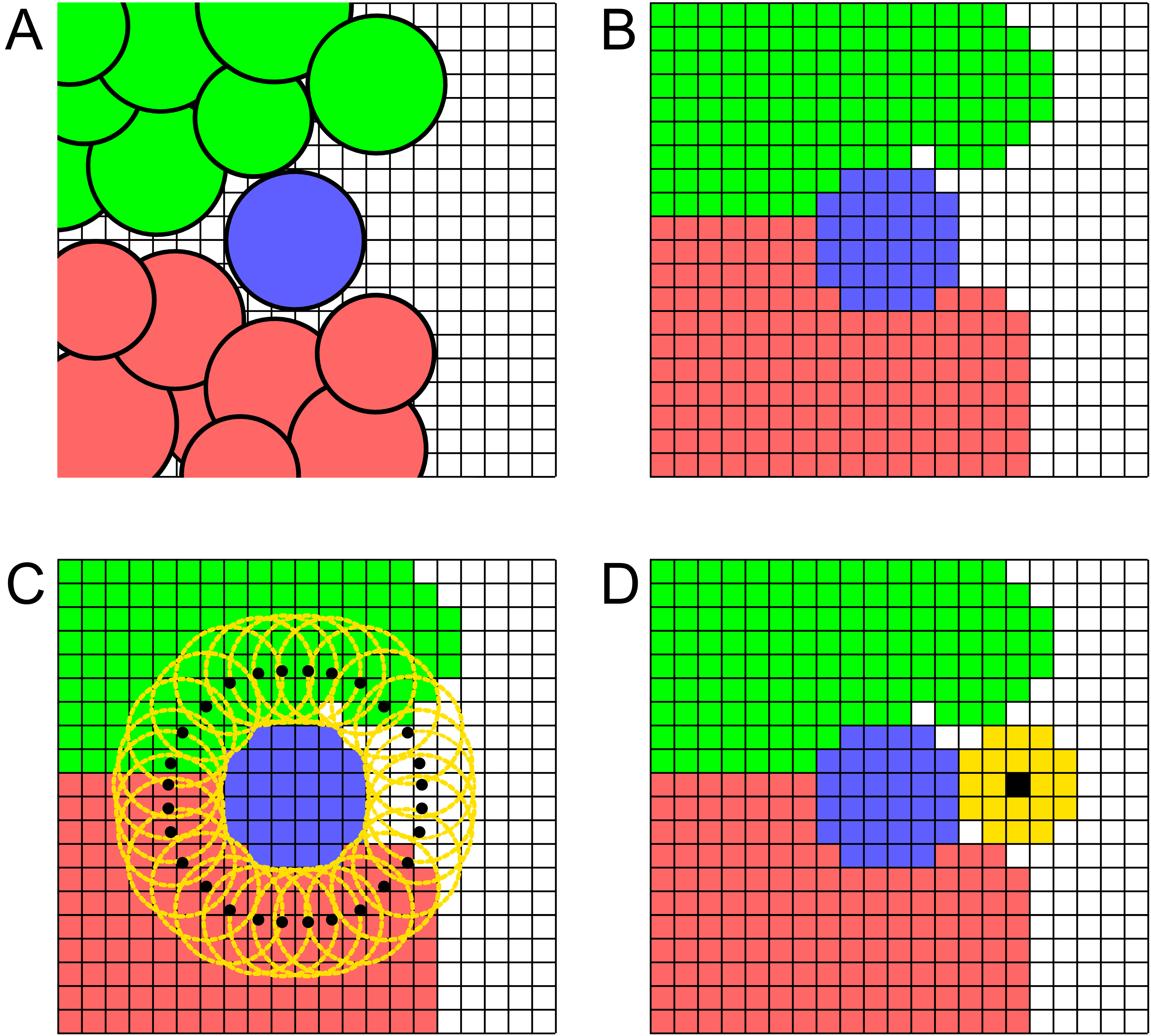

Supplement: Figure S1 — Algorithm for calculating solvent accessible surface area (SASA). (A) grid is constructed around water (blue) of interest including atoms from two proteins (green and red); (B) grid boxes fully or partially occupied by atoms (Van der Waals volume) are set as unavailable; (C) a set of spheres (yellow dashed lines) centered (black circles) at a distance rVdW + rsolvent from the water of interest are constructed (rVdW = rsolvent = 1.4 Å in this case); (D) if entire volume, i.e., all grid boxes, of one of these spheres is available, then the surface area represented by the center of that sphere (black cube) is solvent accessible. All such areas are summed to obtain the SASA for the water molecule of interest. (TIFF) [file pone.0024712.s001.tiff]
