# Supplementary material for: Bound Water at Protein-Protein Interfaces: Partners, Roles and Hydrophobic Bubbles as a Conserved Motif
Source: PLoS One. 2011 Sep 22;6(9):e24712. doi: 10.1371/journal.pone.0024712 (PMC3178540; doi:10.1371/journal.pone.0024712)
Supplement: Table S1 — Protein complexes examined in study with interface parameters and water roles. (PDF) [file pone.0024712.s002.pdf]

Table S1: Protein complexes examined in study with interface parameters and water roles.

					Relevant to:		
**PDB ID**	**Resolution (Å)**	**All H_2O**	**Interface**	**Interface H_2O**	**0**	**1**	**2**
1a4y[a]	2.00	133	A / B	16	3	3 / 3	7
1ava[a]	1.90	748	A / C	38	8	9 / 9	12
1avw	1.75	142	A / B	11	1	2 / 6	2
1blx	1.90	294	A / B	33	8	7 / 10	8
1d2z[a]	2.00	266	AC / B	30	9	5 / 9	7
1eer	1.90	298	A / BC	33	8	11 / 9	5
1ev2[a]	2.20	263	C / FGH	14	2	2 / 4	6
1f3v	2.00	208	A / B	12	3	4 / 3	2
1fns	2.00	636	A / LH	21	5	1 / 5	10
1fyh[a]	2.04	481	AD / B	24	6	8 / 4	6
1g4y	1.60	199	B / R	19	4	4 / 5	6
1ghq	2.04	666	A / BC	20	4	7 / 2	7
1gpq[a]	1.60	635	B / CD	38	9	12 / 6	11
1he1[a]	2.00	712	A / C	28	10	2 / 15	1
1hx1	1.90	359	A / B	28	6	6 / 10	6
1i2m[a]	1.76	475	A / B	28	2	5 / 11	10
1i7w[a]	2.00	669	A / B	24	7	8 / 2	7
1iqd	2.00	477	AB / C	25	8	5 / 9	3
1jiw	1.74	592	I / P	29	7	4 / 14	4
1jyo[a]	1.90	629	AC / F	37	10	15 / 5	7
1ksh	1.80	124	A / B	13	2	4 / 3	4
1ktz	2.15	163	A / B	17	1	3 / 5	8
1kxp	2.10	388	A / D	42	11	13 / 9	9
1kxq[a]	1.60	2807	BD / E	45	4	11 / 14	16
1lk3[a]	1.91	1203	LHM / B	23	3	6 / 6	8
1nf3[a]	2.10	423	A / C	16	2	6 / 6	2
1nmb	2.20	83	LH / N	8	2	2 / 1	3
1o94[a]	2.00	2149	A / CD	20	2	9 / 4	5
1okk	2.05	568	A / B	43	7	16 / 14	6
1ors	1.90	403	A / C	6	1	3 / 0	2
1osp	1.95	328	HL / O	23	2	8 / 9	4
1ow3	1.80	374	A / B	39	9	7 / 9	14
1oy3	2.05	248	BC / D	24	8	5 / 4	7
1pxv[a]	1.80	458	A / C	27	7	8 / 5	7
1q40[a]	1.95	331	A / BD	29	7	10 / 7	5
1r8s	1.46	350	A / E	31	11	9 / 5	6
1rew[a]	1.86	185	ABD / C	16	1	4 / 7	4
1slu	1.80	137	A / B	5	4	0 / 1	0
1sq2	1.45	189	L / N	20	1	11 / 1	7
1t6g[a]	1.80	1074	AB / C	55	11	15 / 18	11
1ta3	1.70	785	A / B	52	12	7 / 12	21
1tue[a]	2.10	969	M / LQ	16	4	4 / 3	5
1tx4	1.65	497	A / B	44	12	7 / 17	8
1tx6[a]	2.20	492	ABC / I	20	5	3 / 6	6
1unn[a]	1.90	947	AB / C	41	11	15 / 7	8
1usu	2.15	227	A / B	13	2	2 / 6	3

1v7p	1.90	463	AB / C	37	8	10 / 8	11
1vg0	2.20	438	A / B	32	8	11 / 7	6
1wa5	2.00	472	AC / B	28	8	10 / 7	3
1www[a]	2.20	262	VW / X	21	7	4 / 6	4
1wxc[b]	1.20	393	A / B	25	7	2 / 8	8
1xg2	1.90	453	A / B	41	13	3 / 14	11
1xkp	1.70	179	A / BC	26	9	9 / 1	7
1xx9[a]	2.20	260	AB / C	15	2	6 / 4	3
1yar[a]	1.90	3470	DEF / O	19	6	5 / 5	3
1ycs	2.20	275	A / B	7	0	2 / 2	3
1yro	1.90	830	BD / C	46	10	10 / 13	13
1yu6	1.55	329	B / D	17	6	4 / 3	4
1z5y	1.94	254	D / E	17	5	2 / 8	2
1zc3[a]	2.00	413	A / D	23	9	7 / 4	3
1ze3	1.84	510	CH / D	27	5	4 / 12	6
1zhh	1.94	333	A / B	32	4	9 / 9	10
2a2q	1.80	722	HL / T	57	10	14 / 15	18
2a9k	1.73	210	A / B	21	4	8 / 7	2
2aq2	1.80	231	A / B	21	4	5 / 6	6
2arp	2.00	171	A / F	19	5	5 / 6	3
2b2x[a]	2.20	226	A / HL	8	0	3 / 1	4
2bcg	1.48	923	G / Y	48	12	14 / 13	9
2bex[a]	1.99	616	AB / C	36	4	14 / 4	14
2bkk[a]	2.15	347	AC / B	16	7	3 / 3	3
2bo9[a]	1.60	1153	AC / D	56	14	13 / 17	12
2cio	1.50	161	A / B	4	1	1 / 1	1
2co7	1.80	274	A / B	29	10	7 / 5	7
2dfk[a]	2.15	581	AC / B	47	14	11 / 17	5
2e2d	2.00	323	A / C	34	8	14 / 9	3
2eke[a]	1.90	375	A / B	21	3	4 / 7	7
2es4[a]	1.85	843	AB / D	55	18	14 / 13	10
2f2l	2.10	195	A / X	9	5	0 / 3	1
2f93	2.00	68	A / B	3	1	0 / 1	1
2f95	2.20	30	A / B	3	1	0 / 2	0
2fd6[a]	1.90	336	ALH / U	13	4	6 / 0	3
2fdb[a]	2.28	125	MN / P	19	4	6 / 4	5
2fm8	2.20	612	AB / C	53	15	16 / 12	10
2fu5[a]	2.00	301	A / C	12	2	2 / 3	5
2g2u	1.60	331	A / B	39	11	4 / 13	11
2gc7[a]	1.90	1234	ABDE / C	17	7	4 / 3	3
2gh0[a]	1.92	236	A / C	8	3	1 / 2	2
2goo[a]	2.20	327	A / BC	36	8	7 / 18	3
2hqs[a]	1.50	2812	DF / G	53	13	19 / 12	9
2iaa[a]	1.95	989	ABD / C	13	8	1 / 4	0
2j12	1.50	287	A / B	37	17	9 / 9	2
2j59[a]	2.10	1656	ABCDF / N	45	11	4 / 19	11
2jjs[a]	1.85	540	AB / D	39	8	10 / 18	3
2npt[a]	1.75	320	A / D	15	1	8 / 4	2
2nqd	1.75	564	A / B	36	11	7 / 14	4
2ns1	1.96	498	A / B	20	7	6 / 3	4
2nxy[a]	2.00	806	BCD / A	44	9	14 / 11	10
2nz8	2.00	242	A / B	42	6	19 / 7	10

2ode[a]	1.90	655	A / B	44	11	8 / 13	12
2omz	1.60	800	A / B	61	9	25 / 12	15
2ot3	2.10	463	A / B	25	9	5 / 6	5
2oul	2.20	171	A / B	18	9	6 / 1	2
2p45	1.10	319	A / B	21	5	8 / 3	5
2q0o[a]	2.00	484	AB / C	43	12	12 / 12	7
2q4g[a]	1.95	854	WY / X	40	7	16 / 6	11
2r25	1.70	238	A / B	21	3	7 / 9	2
2sic	1.80	258	E / I	17	4	5 / 6	2
2v9t	1.70	385	A / B	34	11	2 / 17	4
2vol	1.95	241	A / B	21	2	4 / 10	5
2vsm	1.80	705	A / B	54	8	18 / 19	9
2vxt	1.49	593	HL / I	33	5	11 / 4	13
2wel	1.90	405	A / D	14	2	4 / 7	1
2wwx	1.50	117	A / B	21	2	6 / 8	5
2wy3[a]	1.80	639	A / B	32	9	18 / 4	1
2xg5	2.00	202	A / B	32	12	6 / 8	6
2xgy	1.80	367	A / B	30	15	2 / 10	3
2xna	2.10	249	AB / C	10	1	3 / 3	3
2xqy[a]	2.05	952	E / JK	27	6	3 / 11	7
2yvj	1.90	78	A / B	1	1	0 / 0	0
2z0d	1.90	314	A / B	46	12	17 / 10	7
2z3q[a]	1.85	228	ACD / B	24	5	12 / 5	2
2zd1	1.80	626	A / B	69	28	21 / 15	5
2zfd	1.20	236	A / B	24	6	7 / 5	6
3a4u	1.84	189	A / B	16	5	3 / 3	5
3a8k[a]	1.95	1495	AB / E	33	9	4 / 14	6
3a98[a]	2.10	133	AC / D	18	2	2 / 6	8
3bh7	1.90	219	A / B	26	11	6 / 5	4
3bn3	2.10	226	A / B	22	6	4 / 9	3
3bn9[a]	2.17	824	A / EF	32	5	9 / 10	8
3bwu	1.76	641	CD / F	46	17	6 / 17	6
3bx1[a]	1.85	574	AB / C	28	8	5 / 11	4
3bx7	2.10	186	A / C	31	16	9 / 5	1
3cbj	1.80	243	A / B	27	11	7 / 5	4
3cip	1.60	461	A / G	28	9	13 / 2	4
3cx8	2.00	298	A / B	33	10	10 / 7	6
3d85[a]	1.90	906	ABD / C	30	6	11 / 3	10
3d9a	1.20	683	LH / C	33	4	8 / 4	17
3ddc	1.80	115	A / B	18	7	7 / 1	3
3dlq	1.90	309	I / R	26	6	8 / 8	4
3egg[a]	1.85	521	AB / C	39	9	17 / 5	8
3egv	1.75	415	A / B	42	13	13 / 9	7
3evs	2.10	51	B / C	4	1	0 / 1	2
3f62	2.00	120	A / B	13	5	5 / 2	1
3f75	1.99	167	A / P	28	4	8 / 8	8
3ffd	2.00	204	AB / P	11	2	4 / 1	4
3fhi	2.00	159	A / B	18	4	11 / 3	0
3g5o[a]	2.00	173	AD / C	17	5	6 / 5	1
3gew[a]	2.00	299	AD / C	24	8	6 / 4	6
3gmw[a]	2.10	372	A / B	14	2	4 / 4	4
3grw	2.10	274	A / LH	29	10	8 / 3	8

3hct	2.10	195	A / B	12	3	4 / 3	2
3hei[a]	2.00	2756	CGIO / D	37	10	13 / 11	3
3hg0[a]	2.10	446	ABC / D	15	3	3 / 6	3
3hh2[a]	2.15	349	AB / C	23	6	5 / 7	5
3hy2[a]	2.10	321	AB / X	30	14	5 / 8	3
3hzh	1.96	158	A / B	20	9	4 / 5	2
3jza	1.80	246	A / B	37	8	9 / 12	8
3k2m[a]	1.75	286	A / CD	23	4	6 / 5	8
3kdf[a]	1.98	260	BD / C	15	4	3 / 6	2
3kdj	1.88	170	A / B	9	3	1 / 3	2
3kf6	1.65	191	A / B	22	7	5 / 4	6
3kld	2.00	415	A / B	21	4	7 / 5	5
3kmu	1.80	298	A / B	15	3	4 / 5	3
3kyj	1.40	273	A / B	16	8	6 / 0	2
3l9j	2.10	246	C / T	16	0	2 / 5	9
3liz[c]	1.80	870	A / HL	43	13	11 / 14	5
3lxr	1.68	484	A / F	48	16	11 / 17	4
3m18	1.95	261	A / B	34	3	12 / 9	10
3m7f	2.00	141	A / B	11	5	3 / 1	2
3ma2[a]	2.05	145	AD / B	12	4	6 / 0	2
3ma9	2.05	442	A / LH	23	5	4 / 10	4
3mc0[a]	2.00	438	A / BD	22	7	8 / 3	4
3mdy[a]	2.05	687	AC / B	21	4	3 / 7	7
3n3a[a]	1.99	280	A / D	15	4	5 / 3	3
3nce	2.00	452	A / B	33	11	8 / 8	6
3og6	2.10	266	A / B	23	7	1 / 8	7
3oky	2.19	389	A / B	26	9	6 / 1	10
3orv[a]	1.91	1301	A / CDF	31	11	11 / 5	4
3q3j	1.97	38	A / B	3	0	1 / 1	1

Notes: [a]These complexes have multiple protein-protein interfaces – only one (as indicated) was selected for this study; [b]Three waters (HOH254, HOH281 and HOH282) were deleted because of steric clashes; [c]One water (HOH412) was deleted because of steric clashes.
