# Supplementary material for: Bound Water at Protein-Protein Interfaces: Partners, Roles and Hydrophobic Bubbles as a Conserved Motif
Source: PLoS One. 2011 Sep 22;6(9):e24712. doi: 10.1371/journal.pone.0024712 (PMC3178540; doi:10.1371/journal.pone.0024712)
Supplement: Table S2 — Water Rank, HINT score, Relevance and solvent accessible surface area for full data set. (PDF) [file pone.0024712.s003.pdf]

Table S2: Water Rank, HINT score, Relevance and solvent accessible surface area for full data set.

PDB ID	Water Name	Rank A	Score A	Relev A	Rank B	Score B	Relev B	Total Rank	Total Score	Overall Relev	Water Relev to:	SASA (A^2)
1A4Y	HOH1	1.29	408	0.565	2.13	-96	0.205	3.41	312	0.778	Protein A	2
	HOH2	3.67	35	0.575	1.18	70	0.333	4.85	105	0.727	Both	7
	HOH19	3.51	-40	0.481	1.24	92	0.360	4.75	52	0.674	Both	8
	HOH25	3.72	-32	0.522	1.31	68	0.347	5.03	36	0.676	Both	4
	HOH52	2.34	354	0.685	1.09	-174	-0.137	3.43	181	0.722	Protein A	3
	HOH54	3.62	124	0.657	1.25	21	0.295	4.87	144	0.796	Both	10
	HOH56	1.05	335	0.419	0.95	30	0.264	2.00	365	0.678	Both	54
	HOH59	0.00	-35	-0.039	2.21	-236	-0.280	2.21	-271	-0.362	Neither	2
	HOH60	3.78	314	0.822	1.46	-40	0.230	5.24	274	0.923	Protein A	18
	HOH61	2.30	266	0.624	2.60	141	0.563	4.91	407	0.946	Both	13
	HOH68	0.98	72	0.299	1.03	24	0.273	2.01	96	0.431	Both	41
	HOH70	1.05	-89	0.187	2.24	134	0.508	3.29	45	0.535	Protein B	13
	HOH71	0.72	-7	0.196	0.00	-255	-0.299	0.72	-262	-0.341	Neither	11
	HOH72	0.89	-39	0.201	1.05	-321	-0.487	1.94	-360	-0.586	Neither	6
	HOH73	0.91	10	0.242	1.12	62	0.315	2.03	72	0.404	Protein B	17
	HOH74	1.32	-179	-0.150	2.38	105	0.490	3.70	-74	0.477	Protein B	1
1AVA	HOH20	3.45	68	0.575	0.00	-67	-0.039	3.45	1	0.513	Protein A	0
	HOH22	2.32	265	0.625	0.00	-97	-0.040	2.32	168	0.558	Protein A	3
	HOH28	2.19	-14	0.326	1.18	86	0.347	3.37	72	0.570	Both	17
	HOH39	2.45	-93	0.277	1.12	16	0.280	3.56	-77	0.452	Both	17
	HOH54	0.91	-188	-0.170	1.03	65	0.302	1.94	-123	-0.028	Protein B	1
	HOH87	1.84	-163	-0.113	0.00	13	-0.039	1.84	-150	-0.085	Neither	2
	HOH88	0.81	-90	0.144	0.00	-40	-0.039	0.81	-130	-0.040	Neither	22
	HOH94	2.39	800	0.853	0.00	-90	-0.040	2.39	710	0.836	Protein A	7
	HOH113	1.25	-54	0.219	2.08	-110	0.001	3.33	-164	-0.115	Neither	0
	HOH115	3.10	59	0.527	0.00	-129	-0.041	3.10	-70	0.394	Protein A	44
	HOH148	1.98	-163	-0.114	1.01	39	0.280	2.99	-124	-0.024	Protein B	12
	HOH177	0.00	-170	-0.098	0.70	-87	0.128	0.70	-257	-0.330	Neither	19
	HOH196	2.41	95	0.483	1.07	-119	-0.017	3.49	-24	0.493	Protein A	20
	HOH197	2.23	91	0.454	1.06	155	0.350	3.29	247	0.738	Both	47
	HOH198	1.17	98	0.354	1.10	24	0.284	2.27	122	0.496	Both	4
	HOH200	1.19	-43	0.232	3.91	-81	0.500	5.10	-123	-0.018	Protein B	0
	HOH201	1.07	-121	-0.019	0.88	-8	0.223	1.95	-129	-0.040	Neither	4
	HOH241	1.89	270	0.589	0.00	-213	-0.192	1.89	57	0.375	Protein A	8
	HOH292	0.91	45	0.267	0.85	5	0.228	1.76	50	0.358	Protein A	18
	HOH537	1.09	240	0.374	1.26	429	0.565	2.36	669	0.825	Both	8
	HOH538	2.54	-32	0.364	2.68	70	0.490	5.22	38	0.686	Both	4
	HOH539	1.02	-30	0.230	1.11	-39	0.237	2.13	-69	0.245	Neither	0
	HOH540	0.96	29	0.264	2.62	462	0.768	3.58	491	0.870	Both	2
	HOH541	1.18	26	0.295	2.76	369	0.734	3.93	395	0.867	Both	23
	HOH542	1.19	-15	0.259	2.44	-13	0.369	3.63	-28	0.511	Both	16
	HOH543	0.00	-30	-0.039	0.90	-149	-0.082	0.90	-179	-0.149	Neither	8
	HOH547	0.00	-254	-0.296	1.07	203	0.347	1.07	-52	0.221	Protein B	6
	HOH550	1.14	-110	0.003	2.41	34	0.418	3.55	-76	0.451	Protein B	0
	HOH551	2.07	9	0.332	1.24	238	0.431	3.31	247	0.740	Both	12
	HOH553	1.14	81	0.334	2.60	141	0.562	3.74	221	0.794	Both	3
	HOH571	0.00	-112	-0.030	2.00	-16	0.286	2.00	-128	-0.036	Protein B	54
	HOH574	2.36	-300	-0.433	2.59	327	0.693	4.95	27	0.664	Protein B	40
	HOH579	2.20	-106	0.209	1.35	261	0.489	3.55	154	0.699	Protein B	27
	HOH588	0.85	45	0.258	1.07	-82	0.197	1.93	-37	0.251	Protein A	46
	HOH596	0.97	4	0.248	2.18	68	0.422	3.15	72	0.544	Protein B	28
	HOH613	1.13	142	0.364	1.20	-205	-0.208	2.32	-63	0.291	Protein A	19
	HOH622	0.00	-259	-0.308	0.79	-89	0.142	0.79	-348	-0.555	Neither	15
	HOH626	0.97	14	0.254	1.18	21	0.291	2.15	35	0.378	Both	10
1AVW	HOH5	3.94	-43	0.538	1.22	107	0.371	5.16	64	0.705	Both	16
	HOH11	3.87	-209	-0.215	1.25	103	0.373	5.12	-106	0.548	Protein B	0

	HOH26	0.90	88	0.296	0.77	-116	-0.010	1.66	-28	0.252	Protein A	15
	HOH50	1.09	-171	-0.130	0.86	37	0.253	1.94	-134	-0.050	Protein B	13
	HOH52	2.10	145	0.503	0.00	-473	-0.890	2.10	-329	-0.507	Protein A	15
	HOH109	0.93	-27	0.218	1.20	310	0.464	2.13	283	0.619	Protein B	26
	HOH111	0.00	-235	-0.248	0.99	202	0.330	0.99	-34	0.222	Protein B	4
	HOH124	1.00	51	0.287	1.19	192	0.388	2.19	242	0.595	Both	33
	HOH127	0.99	-62	0.200	2.03	0	0.312	3.02	-62	0.393	Protein B	0
	HOH134	0.00	10	-0.039	1.85	-6	0.291	1.85	4	0.304	Protein B	22
	HOH136	0.00	-102	-0.040	1.95	-275	-0.374	1.95	-377	-0.631	Neither	3
1BLX	HOH330	1.27	-125	-0.030	2.11	-96	0.202	3.38	-221	-0.245	Neither	0
	HOH337	2.10	73	0.416	0.00	-291	-0.391	2.10	-218	-0.238	Protein A	2
	HOH346	2.27	113	0.485	0.99	-12	0.239	3.26	101	0.584	Protein A	10
	HOH347	1.15	-134	-0.048	2.15	-29	0.300	3.30	-163	-0.112	Protein B	3
	HOH352	1.23	184	0.401	1.12	-210	-0.220	2.35	-26	0.340	Protein A	3
	HOH358	3.53	434	0.844	1.17	6	0.276	4.70	439	0.944	Both	1
	HOH410	2.35	-150	-0.083	1.11	65	0.315	3.45	-85	0.427	Protein B	21
	HOH428	1.13	73	0.325	0.86	-47	0.189	1.98	26	0.339	Protein A	19
	HOH450	1.20	-55	0.220	2.08	-136	-0.055	3.28	-191	-0.176	Neither	0
	HOH505	0.82	-158	-0.102	0.00	-93	-0.040	0.82	-250	-0.314	Neither	0
	HOH167	1.09	-23	0.247	2.34	239	0.611	3.43	216	0.751	Protein B	25
	HOH171	0.98	-423	-0.754	2.56	-198	-0.191	3.54	-621	-1.330	Neither	0
	HOH173	1.23	-100	0.170	2.27	108	0.478	3.50	8	0.527	Protein B	8
	HOH176	2.33	58	0.433	2.42	149	0.549	4.75	207	0.896	Both	20
	HOH177	1.08	38	0.292	2.61	-26	0.381	3.69	12	0.559	Both	32
	HOH179	1.09	59	0.309	1.28	-173	-0.136	2.38	-114	-0.005	Protein A	12
	HOH181	0.00	-33	-0.039	0.81	154	0.300	0.81	121	0.298	Protein B	35
	HOH183	0.96	-93	0.167	1.21	13	0.285	2.16	-80	0.236	Protein B	13
	HOH184	2.46	193	0.603	1.33	-377	-0.630	3.79	-184	-0.158	Protein A	31
	HOH186	1.02	-21	0.237	2.65	9	0.424	3.67	-12	0.533	Protein B	24
	HOH197	0.00	-35	-0.039	0.99	436	0.449	0.99	401	0.436	Protein B	0
	HOH220	0.92	-65	0.185	2.28	310	0.651	3.21	245	0.728	Protein B	20
	HOH227	0.92	-274	-0.371	1.10	-50	0.228	2.02	-324	-0.495	Neither	24
	HOH229	0.70	-16	0.185	0.00	-266	-0.327	0.70	-282	-0.391	Neither	10
	HOH233	0.00	-118	-0.033	1.01	250	0.362	1.01	132	0.337	Protein B	4
	HOH239	1.12	-10	0.261	2.73	668	0.848	3.85	658	0.941	Both	27
	HOH248	0.74	-21	0.189	0.00	-114	-0.031	0.74	-135	-0.052	Neither	0
	HOH252	1.11	31	0.291	2.40	-3	0.375	3.51	28	0.547	Both	15
	HOH258	0.00	-43	-0.039	0.74	-297	-0.426	0.74	-340	-0.535	Neither	3
	HOH261	1.29	487	0.608	2.46	-118	-0.012	3.76	370	0.840	Protein A	20
	HOH262	2.80	305	0.705	2.77	308	0.702	5.56	613	1.045	Both	35
	HOH264	1.12	15	0.280	2.23	106	0.471	3.34	121	0.619	Both	1
	HOH265	2.28	-88	0.251	2.47	-17	0.370	4.75	-105	0.530	Both	36
1D2Z	HOH1	3.91	-339	-0.533	0.00	-271	-0.340	3.91	-610	-1.298	Neither	0
	HOH4	1.13	34	0.296	2.04	-185	-0.163	3.17	-151	-0.086	Protein A	9
	HOH9	1.20	184	0.393	1.15	372	0.477	2.36	556	0.787	Both	1
	HOH13	2.53	235	0.635	1.35	217	0.450	3.89	452	0.885	Both	12
	HOH32	0.87	-77	0.166	0.95	203	0.320	1.82	126	0.454	Protein B	10
	HOH40	0.79	-13	0.204	0.00	-229	-0.231	0.79	-241	-0.293	Neither	12
	HOH41	0.00	-153	-0.069	0.00	-213	-0.192	0.00	-366	-0.593	Neither	8
	HOH42	0.87	25	0.245	0.92	-218	-0.239	1.79	-193	-0.182	Neither	4
	HOH52	2.33	-96	0.252	0.99	-124	-0.027	3.32	-220	-0.242	Protein A	17
	HOH57	1.02	-192	-0.179	2.13	-92	0.212	3.15	-285	-0.397	Neither	26
	HOH58	1.29	-66	0.203	2.54	-81	0.306	3.83	-146	-0.073	Protein B	6
	HOH62	1.20	-141	-0.066	2.60	-56	0.344	3.79	-197	-0.189	Protein B	24
	HOH63	1.05	48	0.295	1.08	-602	-1.271	2.14	-554	-1.125	Protein A	18
	HOH68	1.27	18	0.294	4.29	-6	0.596	5.56	12	0.677	Both	11
	HOH77	1.29	88	0.365	2.38	-25	0.346	3.67	63	0.600	Both	39
	HOH80	0.92	-10	0.227	1.15	159	0.376	2.07	148	0.503	Protein B	30
	HOH83	0.90	-9	0.225	1.01	169	0.340	1.91	160	0.499	Protein B	16
	HOH88	1.12	-182	-0.157	1.18	-303	-0.441	2.31	-485	-0.926	Neither	4

	HOH90	1.23	-102	0.169	3.73	39	0.588	4.95	-63	0.582	Protein B	3
	HOH92	0.00	-53	-0.039	1.90	289	0.605	1.90	236	0.559	Protein B	16
	HOH100	0.88	-83	0.164	0.94	-214	-0.228	1.83	-296	-0.425	Neither	0
	HOH103	0.00	-60	-0.039	3.63	214	0.779	3.63	154	0.708	Protein B	1
	HOH104	0.86	-76	0.165	1.10	-49	0.229	1.95	-125	-0.030	Neither	0
	HOH109	2.02	-239	-0.287	1.15	88	0.341	3.16	-150	-0.084	Protein B	18
	HOH128	1.02	44	0.286	2.29	64	0.433	3.32	108	0.598	Both	20
	HOH148	1.33	76	0.358	2.46	8	0.396	3.79	84	0.635	Both	33
	HOH157	0.91	17	0.246	0.00	-131	-0.042	0.91	-113	-0.004	Neither	15
	HOH164	1.25	98	0.368	2.03	38	0.362	3.28	136	0.634	Both	22
	HOH169	2.06	93	0.433	0.00	-187	-0.131	2.06	-94	0.191	Protein A	2
	HOH170	1.01	2	0.253	0.97	-30	0.222	1.98	-27	0.266	Protein A	24
1EER	HOH168	2.32	-130	-0.040	0.00	-157	-0.074	2.32	-287	-0.401	Neither	1
	HOH171	5.04	-3	0.642	0.00	-28	-0.039	5.04	-30	0.617	Protein A	0
	HOH178	1.12	-192	-0.178	1.00	-232	-0.271	2.11	-424	-0.756	Neither	0
	HOH182	1.10	1	0.267	0.89	-139	-0.060	1.99	-138	-0.059	Protein A	3
	HOH187	2.59	-278	-0.380	1.03	-111	0.003	3.61	-388	-0.661	Neither	2
	HOH190	0.96	131	0.328	0.85	122	0.305	1.81	253	0.563	Both	39
	HOH191	3.90	221	0.814	1.39	-42	0.228	5.28	179	0.890	Protein A	2
	HOH197	3.03	74	0.533	0.00	-76	-0.040	3.03	-2	0.457	Protein A	2
	HOH200	2.31	-186	-0.166	1.18	409	0.509	3.49	223	0.760	Protein B	20
	HOH202	1.36	35	0.317	1.97	93	0.422	3.33	128	0.628	Both	42
	HOH205	0.99	45	0.281	0.82	-68	0.164	1.81	-24	0.266	Protein A	10
	HOH212	1.22	323	0.485	1.15	146	0.374	2.37	469	0.751	Both	30
	HOH222	2.65	202	0.638	2.33	-407	-0.711	4.98	-206	-0.209	Protein A	0
	HOH224	1.05	-56	0.215	0.00	-57	-0.039	1.05	-113	-0.002	Neither	2
	HOH227	1.10	5	0.270	0.80	-106	0.131	1.90	-101	0.160	Protein A	13
	HOH229	1.06	36	0.286	0.00	41	-0.038	1.06	76	0.315	Protein A	50
	HOH235	0.97	8	0.250	0.98	-46	0.210	1.95	-39	0.249	Protein A	30
	HOH237	1.09	14	0.275	2.01	166	0.516	3.10	180	0.677	Both	1
	HOH242	2.49	-24	0.365	2.43	-74	0.297	4.92	-97	0.547	Both	0
	HOH244	2.27	429	0.724	0.00	-177	-0.111	2.27	252	0.611	Protein A	23
	HOH257	1.43	149	0.437	2.35	-146	-0.075	3.78	4	0.564	Protein A	34
	HOH228	0.00	-22	-0.039	0.86	57	0.267	0.86	35	0.251	Protein B	23
	HOH232	0.95	-120	-0.019	0.99	-39	0.218	1.94	-159	-0.106	Neither	0
	HOH233	1.27	-85	0.183	4.59	222	0.879	5.86	137	0.826	Protein B	0
	HOH238	1.16	-244	-0.300	1.19	316	0.464	2.35	71	0.449	Protein B	23
	HOH255	0.00	-237	-0.253	1.00	-180	-0.151	1.00	-418	-0.739	Neither	3
	HOH273	0.93	14	0.247	2.37	131	0.521	3.30	145	0.651	Protein B	19
	HOH234	0.86	-385	-0.652	1.15	423	0.499	2.02	38	0.359	Protein B	6
	HOH247	1.13	-77	0.204	3.78	533	0.902	4.90	457	0.964	Protein B	1
	HOH248	0.00	-112	-0.030	1.79	-55	0.222	1.79	-167	-0.123	Neither	17
	HOH276	0.00	-181	-0.120	0.94	127	0.323	0.94	-55	0.196	Protein B	30
	HOH290	0.88	-29	0.207	0.86	-297	-0.427	1.74	-326	-0.501	Neither	37
	HOH329	0.00	-201	-0.164	2.04	238	0.576	2.04	37	0.362	Protein B	22
1EV2	HOH33	1.39	138	0.424	2.38	48	0.429	3.78	186	0.778	Both	8
	HOH35	1.34	481	0.628	2.29	-80	0.262	3.63	401	0.839	Both	0
	HOH36	1.36	-144	-0.072	2.57	93	0.500	3.92	-51	0.529	Protein B	0
	HOH37	2.36	1	0.374	2.53	106	0.508	4.89	107	0.732	Both	19
	HOH39	5.26	334	0.939	0.00	-301	-0.418	5.26	34	0.684	Protein A	38
	HOH170	0.82	22	0.235	1.00	-72	0.194	1.82	-49	0.231	Neither	6
	HOH187	1.11	37	0.297	2.38	-124	-0.026	3.49	-86	0.431	Protein A	12
	HOH188	2.54	-148	-0.079	2.97	398	0.771	5.50	250	0.935	Protein B	13
	HOH198	0.98	4	0.250	1.03	-4	0.251	2.01	-1	0.309	Both	2
	HOH201	0.97	14	0.255	1.01	62	0.297	1.98	76	0.402	Both	1
	HOH211	1.19	25	0.296	4.29	565	0.956	5.48	591	1.035	Both	21
	HOH215	1.00	-38	0.220	1.24	-229	-0.265	2.24	-268	-0.355	Neither	0
	HOH216	0.96	-60	0.196	2.32	254	0.618	3.28	194	0.718	Protein B	2
	HOH217	2.15	-70	0.246	1.29	109	0.385	3.44	39	0.548	Protein B	0
1F3V	HOH12	2.22	-429	-0.771	1.35	385	0.578	3.56	-44	0.485	Protein B	13

	HOH15	1.21	-13	0.261	2.31	193	0.581	3.52	180	0.735	Both	5
	HOH34	1.07	85	0.324	0.00	-144	-0.057	1.07	-59	0.215	Protein A	0
	HOH35	1.36	-235	-0.278	2.24	29	0.387	3.60	-206	-0.211	Protein B	31
	HOH40	1.01	-114	-0.005	0.85	-13	0.214	1.87	-127	-0.036	Neither	1
	HOH99	0.00	-174	-0.106	2.44	82	0.473	2.44	-93	0.276	Protein B	25
	HOH107	0.00	-81	-0.040	0.90	-314	-0.470	0.90	-395	-0.679	Neither	3
	HOH144	1.12	225	0.374	2.40	-211	-0.222	3.52	14	0.536	Protein A	29
	HOH178	3.94	560	0.925	1.43	10	0.291	5.37	570	1.025	Both	27
	HOH182	1.07	109	0.339	2.29	-197	-0.190	3.36	-88	0.410	Protein A	1
	HOH194	0.00	18	-0.039	3.33	-208	-0.213	3.33	-189	-0.171	Neither	23
	HOH203	0.98	160	0.335	1.21	-135	-0.053	2.19	25	0.374	Protein A	18
1FNS	HOH761	1.02	31	0.276	1.11	63	0.315	2.13	93	0.444	Both	6
	HOH797	0.85	28	0.245	1.12	122	0.356	1.97	150	0.494	Protein B	24
	HOH1244	0.00	-8	-0.039	1.86	207	0.531	1.86	200	0.526	Protein B	26
	HOH1304	0.87	40	0.256	0.89	80	0.289	1.76	120	0.442	Both	1
	HOH727	2.52	32	0.431	1.34	-6	0.270	3.86	25	0.596	Both	27
	HOH735	1.19	109	0.366	2.25	7	0.362	3.44	116	0.623	Both	60
	HOH738	2.17	18	0.362	1.26	-16	0.258	3.43	2	0.512	Both	18
	HOH740	1.21	124	0.380	2.30	-334	-0.520	3.51	-211	-0.221	Protein A	44
	HOH823	1.15	17	0.284	2.35	210	0.598	3.50	227	0.762	Both	11
	HOH851	1.26	65	0.339	2.63	359	0.715	3.89	423	0.874	Both	0
	HOH891	0.00	-41	-0.039	0.92	-13	0.225	0.92	-54	0.193	Neither	5
	HOH962	0.00	-168	-0.095	0.00	-96	-0.040	0.00	-264	-0.322	Neither	5
	HOH1237	0.94	-3	0.237	2.37	407	0.718	3.31	404	0.809	Protein B	9
	HOH726	2.57	92	0.499	1.30	195	0.424	3.87	287	0.823	Both	25
	HOH813	3.92	122	0.690	1.04	9	0.263	4.95	131	0.774	Both	29
	HOH919	2.35	-244	-0.300	1.13	75	0.327	3.48	-169	-0.126	Protein B	0
	HOH1001	1.19	-189	-0.171	2.26	226	0.593	3.45	37	0.547	Protein B	30
	HOH1010	0.00	-298	-0.412	0.00	-21	-0.039	0.00	-319	-0.467	Neither	8
	HOH1017	1.00	-46	0.214	0.91	18	0.248	1.91	-28	0.264	Neither	31
	HOH1173	1.20	242	0.420	2.24	-24	0.323	3.44	218	0.753	Both	19
	HOH1280	3.34	-135	-0.050	0.00	172	-0.039	3.34	37	0.533	Neither	40
1FYH	HOH8	2.26	35	0.396	0.99	-29	0.225	3.24	6	0.492	Protein A	0
	HOH21	2.48	-58	0.324	1.02	-392	-0.671	3.49	-450	-0.827	Protein A	0
	HOH25	3.98	525	0.918	1.50	100	0.399	5.48	625	1.044	Both	17
	HOH26	2.43	73	0.462	2.28	-28	0.326	4.71	45	0.667	Both	6
	HOH38	2.57	35	0.440	2.72	9	0.433	5.29	44	0.694	Both	3
	HOH55	1.01	83	0.311	0.00	-108	-0.040	1.01	-26	0.231	Protein A	26
	HOH61	2.45	734	0.844	1.09	-59	0.218	3.54	675	0.920	Protein A	36
	HOH117	2.84	201	0.664	2.69	-14	0.405	5.53	188	0.917	Both	28
	HOH119	1.16	59	0.321	3.93	-89	0.492	5.09	-30	0.619	Both	0
	HOH126	1.21	-194	-0.184	2.23	49	0.408	3.44	-145	-0.072	Protein B	13
	HOH145	1.12	-77	0.205	2.45	-251	-0.314	3.57	-327	-0.502	Neither	2
	HOH157	1.12	-122	-0.021	1.23	-119	-0.017	2.34	-240	-0.291	Neither	32
	HOH158	1.06	55	0.300	1.08	-6	0.259	2.14	49	0.394	Both	2
	HOH160	0.00	-97	-0.040	1.12	355	0.450	1.12	258	0.396	Protein B	23
	HOH162	0.96	23	0.260	1.03	-304	-0.445	1.99	-282	-0.390	Protein A	32
	HOH166	1.08	-58	0.218	4.22	32	0.625	5.30	-26	0.632	Protein B	47
	HOH171	1.05	-177	-0.144	2.44	420	0.731	3.48	243	0.763	Protein B	9
	HOH186	1.17	-11	0.262	2.26	-210	-0.219	3.44	-220	-0.243	Protein A	12
	HOH200	0.95	70	0.293	1.12	-234	-0.275	2.07	-164	-0.116	Protein A	19
	HOH212	0.00	-270	-0.336	0.99	-111	0.002	0.99	-380	-0.640	Neither	50
	HOH213	0.00	-406	-0.700	0.00	-178	-0.112	0.00	-583	-1.213	Neither	0
	HOH224	0.00	-173	-0.103	0.00	-74	-0.040	0.00	-247	-0.277	Neither	45
	HOH255	2.60	-289	-0.407	1.01	-52	0.211	3.61	-341	-0.537	Neither	34
	HOH374	1.03	14	0.266	0.00	-195	-0.149	1.03	-181	-0.154	Protein A	1
1G4Y	HOH28	0.99	64	0.295	0.94	49	0.276	1.93	113	0.445	Both	9
	HOH29	1.09	64	0.312	0.00	-165	-0.088	1.09	-101	0.184	Protein A	15
	HOH33	1.01	373	0.427	0.90	107	0.308	1.90	480	0.744	Both	23
	HOH36	1.27	361	0.530	1.18	-33	0.242	2.44	328	0.678	Protein A	17

	HOH37	0.91	95	0.303	0.00	-66	-0.039	0.91	30	0.256	Protein A	29
	HOH52	1.15	-25	0.249	0.92	-35	0.209	2.07	-60	0.243	Neither	22
	HOH56	1.24	-129	-0.039	1.19	-517	-1.017	2.42	-646	-1.408	Neither	16
	HOH61	1.00	-339	-0.533	0.00	-330	-0.495	1.00	-669	-1.482	Neither	14
	HOH70	1.20	-42	0.233	4.01	-68	0.519	5.21	-110	0.015	Protein B	14
	HOH83	2.09	7	0.334	1.17	-260	-0.336	3.26	-253	-0.320	Protein A	44
	HOH101	0.95	-35	0.214	0.99	282	0.378	1.94	248	0.575	Protein B	35
	HOH108	1.30	47	0.325	2.46	54	0.447	3.76	101	0.646	Both	41
	HOH115	0.00	-209	-0.183	2.02	374	0.684	2.02	165	0.515	Protein B	24
	HOH118	0.00	107	-0.038	2.01	-199	-0.195	2.01	-92	0.181	Neither	13
	HOH121	1.13	90	0.338	1.20	44	0.313	2.33	134	0.519	Both	13
	HOH151	0.00	-82	-0.040	2.03	55	0.384	2.03	-27	0.277	Protein B	0
	HOH165	0.00	-149	-0.063	0.94	414	0.430	0.94	265	0.357	Protein B	9
	HOH167	1.16	132	0.372	2.09	427	0.719	3.25	559	0.863	Both	7
	HOH182	0.96	28	0.264	0.99	177	0.335	1.95	205	0.541	Both	4
1GHQ	HOH29	1.16	285	0.433	1.13	-337	-0.527	2.29	-51	0.300	Protein A	1
	HOH38	2.25	-30	0.318	0.00	-372	-0.608	2.25	-401	-0.695	Protein A	24
	HOH60	4.05	-45	0.544	1.12	-9	0.261	5.17	-54	0.601	Both	0
	HOH78	1.92	-227	-0.260	0.00	-176	-0.109	1.92	-403	-0.700	Neither	15
	HOH208	3.84	36	0.602	1.39	120	0.409	5.23	156	0.840	Both	22
	HOH233	1.02	110	0.330	0.00	-560	-1.142	1.02	-450	-0.828	Protein A	40
	HOH306	1.09	461	0.477	1.02	-160	-0.105	2.11	302	0.632	Protein A	41
	HOH330	2.28	51	0.418	0.00	-174	-0.105	2.28	-123	-0.025	Protein A	0
	HOH336	0.00	-135	-0.046	0.00	-122	-0.036	0.00	-257	-0.304	Neither	11
	HOH350	0.00	-123	-0.036	0.00	-144	-0.056	0.00	-267	-0.329	Neither	7
	HOH383	2.49	-62	0.321	2.58	37	0.445	5.08	-25	0.623	Both	50
	HOH391	2.57	-198	-0.191	2.44	40	0.428	5.01	-158	-0.098	Protein B	11
	HOH427	1.03	126	0.339	1.22	142	0.392	2.25	268	0.619	Both	17
	HOH462	0.88	-305	-0.448	1.07	-36	0.234	1.95	-341	-0.538	Neither	13
	HOH466	2.36	259	0.625	1.39	134	0.421	3.75	393	0.848	Both	9
	HOH470	2.47	114	0.512	2.36	-11	0.360	4.82	103	0.724	Both	60
	HOH479	1.30	-108	0.152	2.40	124	0.516	3.70	16	0.563	Protein B	14
	HOH482	0.89	31	0.254	1.17	350	0.473	2.05	381	0.689	Both	25
	HOH493	0.89	155	0.315	1.19	-207	-0.212	2.08	-52	0.255	Protein A	19
	HOH554	1.15	354	0.463	0.99	-62	0.199	2.13	292	0.626	Protein A	19
1GPQ	HOH169	0.00	-180	-0.118	0.00	-172	-0.102	0.00	-353	-0.557	Neither	38
	HOH231	0.91	112	0.312	0.00	-187	-0.132	0.91	-75	0.174	Protein A	56
	HOH232	1.08	131	0.350	0.94	-61	0.190	2.02	70	0.399	Protein A	24
	HOH235	1.42	-59	0.206	2.87	101	0.542	4.29	43	0.639	Protein B	0
	HOH236	1.03	-1	0.255	0.95	21	0.257	1.99	21	0.332	Both	20
	HOH239	2.58	137	0.555	2.61	79	0.491	5.19	216	0.923	Both	5
	HOH241	0.92	-64	0.185	0.00	-24	-0.039	0.92	-88	0.165	Neither	21
	HOH242	2.74	405	0.751	2.35	-295	-0.423	5.08	110	0.745	Protein A	2
	HOH247	2.40	23	0.404	1.00	-2	0.248	3.39	21	0.525	Protein A	14
	HOH262	0.83	96	0.289	0.00	-128	-0.040	0.83	-32	0.195	Protein A	26
	HOH263	0.86	101	0.298	0.00	-197	-0.155	0.86	-96	0.148	Protein A	45
	HOH264	0.95	288	0.374	0.89	-121	-0.021	1.84	167	0.500	Protein A	25
	HOH265	2.39	533	0.780	0.00	-78	-0.040	2.39	455	0.745	Protein A	30
	HOH266	2.58	59	0.466	1.27	69	0.344	3.85	127	0.695	Both	7
	HOH267	1.32	-292	-0.415	2.35	73	0.452	3.67	-219	-0.239	Protein B	9
	HOH268	0.90	-203	-0.203	0.00	12	-0.039	0.90	-191	-0.175	Neither	24
	HOH273	2.58	224	0.636	1.09	21	0.281	3.67	245	0.787	Both	15
	HOH294	1.81	106	0.429	0.00	-120	-0.034	1.81	-14	0.279	Protein A	22
	HOH295	2.45	356	0.696	1.19	67	0.331	3.64	422	0.850	Both	18
	HOH301	1.20	-92	0.183	1.19	-228	-0.260	2.39	-319	-0.483	Neither	11
	HOH310	2.00	54	0.377	0.00	-312	-0.448	2.00	-258	-0.332	Protein A	3
	HOH311	1.13	120	0.359	0.00	-204	-0.170	1.13	-84	0.198	Protein A	32
	HOH314	0.77	38	0.238	0.00	-40	-0.039	0.77	-2	0.208	Neither	41
	HOH327	1.24	85	0.355	4.37	274	0.864	5.61	359	0.964	Both	2
	HOH331	0.00	-1	-0.039	2.38	388	0.708	2.38	387	0.707	Protein B	23

	HOH360	0.72	21	0.216	0.00	-175	-0.107	0.72	-154	-0.093	Neither	21
	HOH381	1.12	16	0.281	3.75	297	0.811	4.87	312	0.910	Both	6
	HOH382	2.84	51	0.489	2.60	131	0.549	5.44	181	0.902	Both	0
	HOH383	0.91	-98	0.156	1.12	-55	0.223	2.03	-153	-0.093	Neither	12
	HOH387	0.00	148	-0.038	0.99	-255	-0.324	0.99	-107	0.163	Neither	10
	HOH389	0.00	-217	-0.201	2.01	-325	-0.498	2.01	-542	-1.091	Neither	11
	HOH392	0.87	189	0.306	1.02	104	0.327	1.89	293	0.608	Both	21
	HOH408	1.20	96	0.357	2.72	575	0.820	3.92	670	0.950	Both	15
	HOH409	2.36	-41	0.325	2.84	232	0.675	5.20	191	0.905	Both	0
	HOH457	1.14	134	0.368	1.17	-70	0.208	2.31	64	0.437	Protein A	10
	HOH483	0.00	-273	-0.346	2.09	-36	0.279	2.09	-309	-0.457	Protein B	5
	HOH489	0.00	-191	-0.140	3.23	424	0.810	3.23	234	0.728	Protein B	30
	HOH532	0.85	10	0.231	1.02	70	0.305	1.87	80	0.402	Protein B	69
1HE1	HOH2003	2.04	-180	-0.151	0.00	43	-0.038	2.04	-137	-0.057	Neither	1
	HOH2005	2.26	-745	-1.731	0.00	-224	-0.218	2.26	-968	-2.532	Neither	0
	HOH2009	1.11	-79	0.204	0.84	147	0.307	1.94	68	0.391	Protein B	24
	HOH2021	0.92	-85	0.169	0.93	49	0.274	1.85	-36	0.251	Protein B	28
	HOH2023	0.92	351	0.400	0.00	-10	-0.039	0.92	341	0.395	Protein A	24
	HOH2034	0.91	-99	0.155	0.86	-52	0.185	1.77	-150	-0.086	Neither	9
	HOH2088	0.96	-280	-0.384	0.85	127	0.307	1.81	-152	-0.091	Protein B	24
	HOH2091	1.17	-60	0.217	1.07	-58	0.217	2.24	-118	-0.014	Neither	18
	HOH2092	1.06	-72	0.202	0.96	-212	-0.224	2.01	-284	-0.396	Neither	42
	HOH2026	2.59	-136	-0.052	2.75	21	0.449	5.34	-114	0.004	Protein B	50
	HOH2027	0.00	-265	-0.324	1.03	-133	-0.047	1.03	-398	-0.687	Neither	16
	HOH2030	0.00	-404	-0.695	0.98	170	0.332	0.98	-234	-0.275	Protein B	7
	HOH2033	1.01	-10	0.244	2.03	509	0.768	3.04	499	0.822	Protein B	37
	HOH2036	1.40	33	0.317	3.97	257	0.825	5.38	290	0.933	Both	28
	HOH2039	0.99	-313	-0.466	1.11	70	0.320	2.10	-243	-0.297	Protein B	0
	HOH2040	0.00	-292	-0.396	0.94	165	0.326	0.94	-127	-0.034	Protein B	11
	HOH2051	0.97	148	0.333	1.15	-57	0.221	2.13	91	0.440	Protein A	0
	HOH2053	1.09	-288	-0.404	1.24	336	0.502	2.34	48	0.423	Protein B	7
	HOH2054	0.00	-231	-0.237	2.22	264	0.613	2.22	33	0.387	Protein B	44
	HOH2055	1.14	-435	-0.786	1.19	-16	0.258	2.32	-451	-0.829	Protein B	1
	HOH2059	1.17	-154	-0.094	4.25	241	0.849	5.41	86	0.734	Protein B	16
	HOH2061	0.00	-125	-0.038	0.82	84	0.278	0.82	-42	0.185	Protein B	27
	HOH2062	0.00	23	-0.039	3.59	35	0.563	3.59	57	0.584	Protein B	23
	HOH2063	0.92	18	0.249	1.15	-188	-0.169	2.07	-170	-0.129	Neither	16
	HOH2085	0.86	25	0.244	0.98	47	0.281	1.84	72	0.390	Protein B	18
	HOH2124	0.73	30	0.225	0.00	-140	-0.052	0.73	-110	0.002	Neither	30
	HOH2165	0.76	-140	-0.062	0.00	-202	-0.166	0.76	-342	-0.539	Neither	40
	HOH2227	0.98	-21	0.230	0.92	-7	0.230	1.90	-28	0.263	Neither	46
1HX1	HOH402	1.23	-319	-0.482	1.15	162	0.376	2.39	-158	-0.101	Protein B	3
	HOH403	2.55	-46	0.350	1.02	-43	0.220	3.57	-88	0.442	Protein A	1
	HOH405	2.72	474	0.782	2.79	3	0.434	5.51	477	1.002	Both	0
	HOH407	1.51	11	0.295	3.71	38	0.585	5.23	49	0.695	Both	1
	HOH413	3.88	74	0.636	1.13	-47	0.230	5.01	28	0.667	Protein A	6
	HOH417	0.92	99	0.308	0.00	-302	-0.421	0.92	-203	-0.202	Protein A	3
	HOH435	2.50	175	0.591	0.89	-314	-0.469	3.39	-139	-0.057	Protein A	32
	HOH446	3.92	53	0.621	1.13	80	0.332	5.05	133	0.785	Both	46
	HOH474	1.37	466	0.631	2.20	-214	-0.229	3.57	251	0.775	Protein A	10
	HOH480	1.08	128	0.348	0.91	-141	-0.064	1.98	-13	0.285	Protein A	33
	HOH497	1.23	28	0.301	1.04	-9	0.251	2.27	20	0.381	Both	59
	HOH509	2.62	-227	-0.259	1.03	134	0.342	3.65	-93	0.449	Protein B	5
	HOH562	1.23	-465	-0.869	1.00	57	0.292	2.23	-408	-0.714	Protein B	26
	HOH628	0.82	-15	0.206	0.00	-184	-0.126	0.82	-200	-0.196	Neither	18
	HOH264	0.00	-269	-0.334	1.07	-160	-0.105	1.07	-428	-0.768	Neither	0
	HOH265	1.29	-25	0.249	2.71	-119	-0.014	4.00	-144	-0.067	Neither	14
	HOH266	0.00	-165	-0.088	1.05	-155	-0.094	1.05	-320	-0.483	Neither	0
	HOH268	2.34	-84	0.269	1.37	132	0.416	3.71	48	0.594	Both	1
	HOH274	0.00	-220	-0.208	0.96	67	0.292	0.96	-152	-0.090	Protein B	22

	HOH279	0.97	-242	-0.295	1.16	-65	0.213	2.13	-308	-0.453	Neither	19
	HOH286	0.00	-71	-0.040	2.18	66	0.420	2.18	-6	0.336	Protein B	12
	HOH287	1.94	-170	-0.130	1.06	462	0.470	3.00	292	0.721	Protein B	10
	HOH291	3.60	417	0.843	1.33	26	0.304	4.92	442	0.960	Both	24
	HOH299	0.94	-39	0.210	1.11	157	0.362	2.06	118	0.464	Protein B	33
	HOH300	0.80	-35	0.187	1.09	439	0.469	1.88	404	0.693	Protein B	8
	HOH346	0.00	-506	-0.984	1.12	-90	0.194	1.12	-597	-1.255	Neither	0
	HOH355	0.91	2	0.236	1.27	68	0.343	2.18	70	0.423	Protein B	11
	HOH356	0.81	-307	-0.451	1.02	316	0.403	1.83	9	0.311	Protein B	14
1I2M	HOH1251	2.38	-256	-0.329	1.05	-110	0.005	3.43	-366	-0.602	Neither	0
	HOH1257	2.40	193	0.593	0.00	32	-0.038	2.40	225	0.611	Protein A	6
	HOH1259	1.12	114	0.353	0.90	-276	-0.376	2.02	-162	-0.112	Protein A	18
	HOH1261	2.66	444	0.762	1.06	79	0.318	3.71	523	0.893	Both	5
	HOH1263	1.15	129	0.367	0.89	-90	0.159	2.04	39	0.365	Protein A	25
	HOH1271	2.04	-53	0.244	0.98	288	0.379	3.02	235	0.701	Protein B	8
	HOH1275	2.35	130	0.517	1.33	211	0.439	3.68	342	0.820	Both	8
	HOH1276	2.19	-204	-0.205	1.06	214	0.349	3.25	10	0.496	Protein B	22
	HOH1279	1.16	130	0.372	1.10	180	0.355	2.26	310	0.649	Both	15
	HOH1282	4.00	97	0.665	1.25	8	0.282	5.25	105	0.746	Both	1
	HOH1285	3.72	-176	-0.140	1.29	183	0.422	5.02	7	0.650	Protein B	10
	HOH1303	2.74	80	0.506	1.17	117	0.366	3.91	196	0.806	Both	12
	HOH1310	3.81	121	0.679	1.28	-14	0.261	5.08	108	0.742	Both	20
	HOH1311	1.14	69	0.325	1.12	-180	-0.151	2.26	-111	0.002	Protein A	24
	HOH424	0.00	-276	-0.353	1.99	-278	-0.380	1.99	-554	-1.125	Neither	3
	HOH452	1.28	15	0.292	2.59	-276	-0.376	3.88	-261	-0.339	Protein A	2
	HOH453	0.00	-201	-0.164	2.08	37	0.369	2.08	-165	-0.118	Protein B	19
	HOH477	1.18	-85	0.193	2.25	47	0.408	3.43	-38	0.471	Protein B	7
	HOH488	1.09	75	0.320	2.34	-85	0.267	3.43	-9	0.500	Both	0
	HOH499	0.00	43	-0.038	2.15	240	0.589	2.15	283	0.621	Protein B	28
	HOH527	1.20	116	0.373	2.31	240	0.609	3.51	356	0.808	Both	15
	HOH548	1.18	169	0.387	1.19	343	0.480	2.37	512	0.770	Both	32
	HOH556	0.00	-61	-0.039	0.90	109	0.309	0.90	47	0.268	Protein B	5
	HOH578	0.88	22	0.246	1.06	225	0.356	1.94	247	0.574	Protein B	15
	HOH590	1.08	-102	0.181	2.26	224	0.591	3.34	121	0.619	Protein B	7
	HOH592	1.10	141	0.354	2.24	30	0.387	3.33	171	0.695	Both	9
	HOH595	0.00	193	-0.039	5.11	63	0.702	5.11	255	0.914	Protein B	2
	HOH597	0.00	-46	-0.039	5.21	248	0.919	5.21	202	0.922	Protein B	8
1I7W	HOH3	3.85	425	0.871	0.00	-89	-0.040	3.85	335	0.837	Protein A	8
	HOH9	1.07	-328	-0.504	0.00	-191	-0.140	1.07	-519	-1.023	Neither	20
	HOH22	2.52	-267	-0.353	1.09	-45	0.230	3.60	-311	-0.462	Neither	39
	HOH25	1.05	-164	-0.116	0.84	-61	0.175	1.89	-225	-0.255	Neither	12
	HOH35	4.40	501	0.945	1.27	-49	0.224	5.67	452	1.000	Protein A	54
	HOH68	4.17	311	0.857	1.47	-198	-0.193	5.64	112	0.771	Protein A	19
	HOH75	0.00	-260	-0.310	0.00	-191	-0.142	0.00	-451	-0.826	Neither	0
	HOH79	0.87	26	0.246	0.81	-247	-0.306	1.68	-221	-0.246	Neither	33
	HOH102	2.66	164	0.600	2.60	3	0.410	5.26	167	0.865	Both	9
	HOH103	1.17	111	0.362	1.02	-36	0.226	2.19	75	0.431	Protein A	25
	HOH115	1.26	468	0.579	0.94	152	0.327	2.20	620	0.808	Both	37
	HOH171	2.16	-180	-0.152	1.01	83	0.312	3.17	-97	0.373	Protein B	50
	HOH188	1.00	115	0.329	0.86	-13	0.215	1.86	102	0.427	Protein A	11
	HOH206	0.77	-392	-0.670	0.00	-100	-0.040	0.77	-492	-0.945	Neither	3
	HOH242	3.46	-193	-0.180	1.09	177	0.355	4.55	-16	0.603	Protein B	27
	HOH255	1.98	-48	0.236	1.04	-18	0.243	3.02	-66	0.388	Neither	34
	HOH265	1.08	58	0.306	0.98	-29	0.223	2.06	28	0.355	Protein A	0
	HOH281	1.07	60	0.306	1.01	-20	0.237	2.08	41	0.374	Protein A	1
	HOH284	4.06	318	0.850	1.15	-87	0.193	5.22	231	0.921	Protein A	0
	HOH292	1.16	203	0.377	1.18	312	0.457	2.34	515	0.770	Both	1
	HOH293	1.01	41	0.283	2.18	8	0.352	3.19	50	0.528	Both	0
	HOH295	1.16	20	0.288	1.21	102	0.364	2.37	121	0.508	Both	8
	HOH296	2.25	-18	0.333	1.33	362	0.556	3.58	345	0.810	Both	20

	HOH297	1.17	-6	0.266	2.61	515	0.789	3.78	509	0.894	Both	9
1IQD	HOH3034	3.88	155	0.742	1.13	19	0.284	5.01	174	0.864	Both	0
	HOH3070	1.17	-215	-0.231	1.15	10	0.278	2.32	-205	-0.209	Protein B	0
	HOH3086	2.61	-128	-0.036	2.44	88	0.479	5.05	-40	0.608	Protein B	1
	HOH3199	1.07	-105	0.177	0.00	-180	-0.118	1.07	-285	-0.398	Neither	0
	HOH3413	1.19	-10	0.263	0.96	58	0.285	2.15	47	0.393	Both	1
	HOH3014	4.00	303	0.839	0.00	24	-0.038	4.00	327	0.847	Protein A	0
	HOH3019	1.30	339	0.529	2.37	-7	0.366	3.67	332	0.815	Both	6
	HOH3024	2.50	5	0.398	0.98	-43	0.212	3.48	-39	0.478	Protein A	2
	HOH3036	0.99	-121	-0.021	0.00	-141	-0.053	0.99	-262	-0.343	Neither	2
	HOH3088	4.05	542	0.929	0.00	-8	-0.039	4.05	534	0.927	Protein A	17
	HOH3161	1.02	-306	-0.450	0.00	-63	-0.039	1.02	-369	-0.611	Neither	0
	HOH3169	0.91	-335	-0.522	0.00	-114	-0.031	0.91	-449	-0.824	Neither	18
	HOH3215	1.16	290	0.435	0.96	3	0.246	2.12	293	0.626	Protein A	24
	HOH3239	1.01	-226	-0.256	0.00	-262	-0.317	1.01	-488	-0.934	Neither	25
	HOH3252	2.04	1	0.316	0.00	-187	-0.131	2.04	-186	-0.165	Protein A	8
	HOH3297	1.09	-68	0.211	0.98	-37	0.217	2.07	-106	0.178	Neither	19
	HOH3383	1.14	-485	-0.925	1.10	-251	-0.315	2.24	-735	-1.700	Neither	45
	HOH3022	1.01	-58	0.205	1.04	-220	-0.242	2.04	-278	-0.381	Neither	21
	HOH3030	0.00	-60	-0.039	1.88	75	0.397	1.88	15	0.321	Protein B	29
	HOH3031	0.00	-218	-0.204	2.12	-58	0.256	2.12	-276	-0.376	Protein B	25
	HOH3064	0.00	-102	-0.040	3.42	104	0.606	3.42	2	0.510	Protein B	8
	HOH3114	1.08	-68	0.210	2.42	14	0.397	3.50	-54	0.465	Protein B	19
	HOH3194	0.99	-1	0.247	1.15	36	0.301	2.14	36	0.378	Protein B	21
	HOH3203	1.01	-176	-0.143	2.58	94	0.502	3.59	-83	0.451	Protein B	3
	HOH3368	1.14	-105	0.177	2.48	130	0.533	3.62	25	0.559	Protein B	33
1JIW	HOH503	4.48	100	0.699	1.54	474	0.687	6.02	573	1.050	Both	0
	HOH522	1.35	-273	-0.369	3.59	187	0.752	4.94	-86	0.558	Protein B	15
	HOH540	0.00	-679	-1.514	5.53	517	1.016	5.53	-162	-0.108	Protein B	4
	HOH545	1.28	-202	-0.201	2.54	447	0.753	3.82	246	0.807	Protein B	2
	HOH551	0.00	-340	-0.523	0.99	197	0.329	0.99	-143	-0.069	Protein B	20
	HOH557	1.11	-427	-0.766	1.22	470	0.560	2.34	42	0.416	Protein B	16
	HOH561	0.00	-21	-0.039	5.64	679	1.062	5.64	658	1.058	Protein B	36
	HOH573	0.00	-267	-0.330	0.96	100	0.314	0.96	-167	-0.122	Protein B	33
	HOH605	1.11	-68	0.214	1.14	72	0.328	2.25	5	0.360	Protein B	15
	HOH646	0.97	-228	-0.260	2.47	229	0.623	3.43	1	0.511	Protein B	56
	HOH666	0.00	-173	-0.103	0.92	-135	-0.052	0.92	-308	-0.454	Neither	0
	HOH675	0.00	-72	-0.040	3.33	-170	-0.128	3.33	-242	-0.293	Neither	2
	HOH686	0.85	-113	-0.003	1.11	-92	0.192	1.96	-205	-0.209	Neither	0
	HOH694	0.00	-436	-0.785	0.72	-15	0.188	0.72	-452	-0.832	Neither	21
	HOH715	1.07	32	0.286	1.16	-201	-0.198	2.23	-168	-0.125	Protein A	23
	HOH722	0.00	-331	-0.498	2.12	85	0.433	2.12	-246	-0.303	Protein B	3
	HOH768	0.84	-32	0.197	1.13	403	0.475	1.96	372	0.681	Protein B	0
	HOH773	0.00	-83	-0.040	3.20	0	0.480	3.20	-84	0.392	Protein B	1
	HOH979	0.00	-201	-0.164	1.06	168	0.350	1.06	-33	0.234	Protein B	28
	HOH107	1.22	-255	-0.326	2.19	37	0.388	3.41	-218	-0.238	Protein B	20
	HOH112	1.12	-215	-0.230	1.11	-186	-0.165	2.23	-401	-0.693	Neither	10
	HOH115	1.36	111	0.396	2.34	-184	-0.160	3.70	-73	0.479	Protein A	10
	HOH116	0.94	-137	-0.055	2.12	-170	-0.129	3.05	-306	-0.450	Neither	0
	HOH117	1.17	25	0.293	1.06	61	0.305	2.23	86	0.449	Both	3
	HOH128	3.67	272	0.793	0.00	-74	-0.040	3.67	198	0.777	Protein A	34
	HOH140	1.22	493	0.571	1.16	28	0.294	2.38	521	0.774	Both	24
	HOH157	1.12	-12	0.259	1.02	353	0.421	2.14	342	0.663	Both	53
	HOH165	2.34	52	0.427	1.04	-30	0.234	3.38	22	0.524	Protein A	26
	HOH182	0.84	-62	0.173	0.00	54	-0.038	0.84	-8	0.216	Neither	56
1JYO	HOH135	2.64	-235	-0.277	2.94	101	0.549	5.57	-134	-0.043	Protein B	13
	HOH136	1.11	57	0.311	1.04	-3	0.253	2.15	54	0.401	Both	2
	HOH153	2.41	-150	-0.085	1.09	-246	-0.303	3.50	-396	-0.681	Neither	13
	HOH156	2.61	67	0.478	1.21	-54	0.220	3.82	13	0.580	Protein A	7
	HOH159	0.92	432	0.433	0.00	-113	-0.030	0.92	319	0.385	Protein A	4

	HOH178	1.00	-385	-0.652	0.00	-52	-0.039	1.00	-437	-0.792	Neither	0
	HOH190	1.18	437	0.519	1.04	-57	0.211	2.21	380	0.692	Protein A	13
	HOH193	0.00	-266	-0.326	0.00	-136	-0.048	0.00	-402	-0.690	Neither	8
	HOH202	0.00	-182	-0.121	0.00	-262	-0.317	0.00	-444	-0.806	Neither	2
	HOH217	1.22	228	0.414	1.11	197	0.358	2.33	425	0.725	Both	2
	HOH224	1.19	46	0.313	0.87	-60	0.181	2.06	-14	0.301	Protein A	0
	HOH134	2.07	-459	-0.852	1.13	217	0.374	3.20	-242	-0.293	Protein B	7
	HOH138	2.59	247	0.648	1.35	20	0.300	3.94	267	0.823	Both	0
	HOH149	2.58	227	0.637	1.25	-26	0.248	3.82	200	0.802	Protein A	0
	HOH154	3.51	-55	0.466	0.00	-163	-0.085	3.51	-218	-0.238	Protein A	19
	HOH161	1.89	-271	-0.364	0.00	-241	-0.263	1.89	-512	-1.004	Neither	29
	HOH162	1.21	173	0.396	0.84	-269	-0.360	2.05	-97	0.186	Protein A	8
	HOH166	0.00	-265	-0.324	0.00	-227	-0.226	0.00	-492	-0.942	Neither	36
	HOH186	3.16	-51	0.422	0.00	-319	-0.465	3.16	-370	-0.612	Protein A	8
	HOH191	1.25	-138	-0.058	1.22	-45	0.230	2.46	-183	-0.158	Neither	9
	HOH200	2.37	-153	-0.091	1.13	25	0.288	3.49	-128	-0.033	Protein B	30
	HOH211	0.00	-243	-0.268	0.73	-69	0.147	0.73	-312	-0.465	Neither	29
	HOH232	1.24	206	0.406	0.89	47	0.265	2.13	253	0.596	Both	4
	HOH238	2.17	60	0.412	0.00	-71	-0.040	2.17	-11	0.327	Protein A	25
	HOH141	2.70	-79	0.332	2.81	-337	-0.528	5.51	-416	-0.735	Protein A	5
	HOH142	1.32	-123	-0.025	3.99	45	0.621	5.31	-77	0.584	Protein B	35
	HOH144	0.81	-192	-0.179	1.14	-54	0.224	1.95	-246	-0.304	Neither	4
	HOH145	1.07	-6	0.257	1.15	-412	-0.724	2.22	-418	-0.740	Protein A	32
	HOH146	2.29	41	0.408	1.39	-263	-0.343	3.67	-221	-0.245	Protein A	38
	HOH155	2.27	15	0.374	1.35	-645	-1.406	3.62	-630	-1.359	Protein A	40
	HOH158	2.20	-34	0.305	1.21	139	0.387	3.41	105	0.606	Both	31
	HOH169	0.98	-5	0.243	1.21	-338	-0.531	2.20	-343	-0.544	Neither	47
	HOH170	0.00	-165	-0.088	1.05	7	0.263	1.05	-158	-0.101	Protein B	39
	HOH175	1.10	59	0.309	1.10	-284	-0.396	2.20	-225	-0.256	Protein A	25
	HOH182	2.17	193	0.560	1.27	98	0.371	3.44	291	0.772	Both	3
	HOH201	1.06	87	0.322	2.20	54	0.410	3.26	141	0.640	Both	28
	HOH210	1.00	41	0.280	2.00	-56	0.229	2.99	-15	0.440	Protein A	29
1KSH	HOH204	1.33	147	0.419	2.16	-45	0.281	3.49	102	0.612	Both	0
	HOH224	1.24	44	0.317	1.24	335	0.499	2.48	379	0.711	Both	6
	HOH227	1.38	-48	0.220	3.81	-27	0.542	5.20	-75	0.582	Protein B	0
	HOH229	1.02	42	0.285	0.00	-420	-0.740	1.02	-378	-0.634	Protein A	31
	HOH152	2.36	-69	0.291	2.74	-164	-0.115	5.10	-233	-0.272	Protein A	14
	HOH153	2.50	50	0.447	2.47	-156	-0.099	4.97	-106	0.540	Protein A	6
	HOH154	2.32	289	0.641	1.33	-213	-0.227	3.65	76	0.609	Protein A	29
	HOH158	0.00	2	-0.039	2.08	149	0.506	2.08	152	0.509	Protein B	23
	HOH170	2.38	20	0.397	1.21	79	0.346	3.59	99	0.621	Both	11
	HOH173	1.08	-183	-0.158	1.30	-60	0.209	2.38	-244	-0.298	Neither	28
	HOH176	0.00	-181	-0.120	3.30	119	0.611	3.30	-63	0.428	Protein B	7
	HOH181	1.15	25	0.291	3.80	446	0.874	4.95	471	0.971	Both	1
	HOH199	0.00	-157	-0.075	1.86	-80	0.190	1.86	-237	-0.282	Neither	20
1KTZ	HOH3	2.47	14	0.404	2.51	-34	0.357	4.98	-20	0.624	Both	19
	HOH4	3.50	153	0.690	0.00	-213	-0.191	3.50	-59	0.461	Protein A	40
	HOH8	3.66	219	0.783	1.05	-102	0.176	4.70	117	0.737	Protein A	15
	HOH9	1.10	150	0.357	1.06	-174	-0.137	2.16	-24	0.309	Protein A	34
	HOH11	1.37	106	0.392	2.52	278	0.657	3.89	384	0.859	Both	36
	HOH17	0.97	5	0.248	0.00	-200	-0.161	0.97	-195	-0.186	Neither	45
	HOH23	2.24	-24	0.323	1.17	230	0.398	3.41	206	0.748	Both	12
	HOH24	1.18	205	0.383	1.14	26	0.291	2.32	231	0.604	Both	21
	HOH34	2.52	-157	-0.100	1.11	172	0.359	3.63	16	0.552	Protein B	0
	HOH54	1.10	131	0.354	0.98	22	0.263	2.08	153	0.510	Both	18
	HOH72	0.00	-74	-0.040	3.39	325	0.781	3.39	251	0.753	Protein B	21
	HOH74	0.00	-88	-0.040	3.59	451	0.856	3.59	363	0.820	Protein B	59
	HOH79	4.06	97	0.668	1.57	28	0.319	5.62	125	0.793	Both	14
	HOH114	0.00	-7	-0.039	1.96	308	0.629	1.96	301	0.624	Protein B	1
	HOH115	0.00	-76	-0.040	1.93	364	0.670	1.93	288	0.608	Protein B	1

	HOH153	1.16	139	0.373	1.18	292	0.447	2.34	431	0.729	Both	8
	HOH161	1.00	47	0.285	1.12	9	0.276	2.12	57	0.400	Both	33
1KXP	HOH1	1.17	-157	-0.100	1.13	25	0.289	2.30	-132	-0.046	Protein B	0
	HOH7	3.33	-5	0.492	1.16	91	0.346	4.49	86	0.688	Both	12
	HOH15	3.36	-289	-0.408	0.00	-206	-0.176	3.36	-496	-0.956	Neither	18
	HOH19	1.32	73	0.354	2.07	-51	0.253	3.39	22	0.525	Both	3
	HOH24	3.67	129	0.672	1.25	-297	-0.427	4.92	-168	-0.121	Protein A	24
	HOH25	3.19	-122	-0.020	0.00	-196	-0.152	3.19	-318	-0.480	Neither	12
	HOH26	1.13	-173	-0.136	1.04	-121	-0.019	2.17	-294	-0.419	Neither	0
	HOH27	2.31	86	0.459	1.30	8	0.285	3.60	94	0.619	Both	11
	HOH28	2.61	668	0.839	1.19	112	0.368	3.81	780	0.957	Both	2
	HOH38	1.31	512	0.627	2.44	-57	0.319	3.75	455	0.873	Both	3
	HOH40	1.19	-267	-0.354	1.01	4	0.255	2.20	-263	-0.344	Protein B	34
	HOH46	2.72	157	0.600	1.13	-186	-0.166	3.85	-29	0.546	Protein A	3
	HOH49	2.08	-432	-0.779	0.00	-139	-0.050	2.08	-571	-1.176	Neither	0
	HOH51	1.21	313	0.473	2.12	-164	-0.117	3.32	149	0.660	Protein A	0
	HOH53	2.66	446	0.763	2.66	5	0.419	5.31	451	0.984	Both	9
	HOH57	3.44	-40	0.471	0.00	-65	-0.039	3.44	-105	0.402	Protein A	15
	HOH59	1.06	-251	-0.315	0.00	-174	-0.105	1.06	-425	-0.758	Neither	26
	HOH66	1.34	118	0.400	2.19	96	0.455	3.53	213	0.766	Both	4
	HOH72	2.02	294	0.622	0.00	-58	-0.039	2.02	236	0.573	Protein A	0
	HOH75	2.13	-53	0.265	0.00	-205	-0.174	2.13	-258	-0.333	Protein A	4
	HOH90	2.55	-18	0.380	2.44	8	0.393	4.99	-10	0.633	Both	14
	HOH106	2.46	302	0.664	2.42	-192	-0.178	4.87	110	0.736	Protein A	10
	HOH109	1.02	-27	0.233	0.00	-91	-0.040	1.02	-118	-0.013	Neither	0
	HOH118	1.42	-101	0.152	2.35	-192	-0.178	3.77	-292	-0.415	Neither	5
	HOH119	1.10	83	0.326	0.94	-9	0.233	2.04	75	0.409	Protein A	27
	HOH134	2.44	143	0.545	1.03	-69	0.200	3.47	74	0.583	Protein A	24
	HOH173	2.01	-16	0.289	1.21	227	0.412	3.23	211	0.723	Both	39
	HOH175	0.99	-96	0.171	1.96	23	0.334	2.96	-73	0.372	Protein B	2
	HOH176	2.35	-106	0.241	1.35	-3	0.274	3.69	-109	0.441	Protein B	32
	HOH193	1.01	-61	0.204	2.22	-92	0.233	3.23	-153	-0.089	Neither	0
	HOH196	1.10	326	0.422	1.15	-213	-0.226	2.25	113	0.483	Protein A	0
	HOH205	2.80	-35	0.395	2.66	-184	-0.159	5.46	-219	-0.239	Protein A	1
	HOH206	0.86	130	0.309	1.13	-117	-0.012	1.99	13	0.322	Protein A	10
	HOH212	0.00	-60	-0.039	2.17	64	0.415	2.17	4	0.344	Protein B	3
	HOH225	0.92	-175	-0.140	1.14	78	0.332	2.06	-97	0.187	Protein B	10
	HOH233	1.13	299	0.426	1.23	-92	0.179	2.36	207	0.597	Protein A	1
	HOH247	0.87	-128	-0.037	1.14	-104	0.178	2.01	-232	-0.272	Neither	31
	HOH321	1.16	-124	-0.028	2.32	84	0.459	3.47	-40	0.476	Protein B	10
	HOH325	0.00	-210	-0.184	0.85	-31	0.199	0.85	-241	-0.291	Neither	3
	HOH341	0.00	-257	-0.303	0.72	-15	0.189	0.72	-272	-0.365	Neither	14
	HOH360	0.00	-80	-0.040	5.31	93	0.736	5.31	13	0.668	Protein B	3
	HOH375	0.94	-141	-0.064	0.99	403	0.437	1.94	262	0.587	Protein B	3
1KXQ	HOH802	3.69	197	0.779	0.00	106	-0.038	3.69	303	0.807	Protein A	15
	HOH828	2.68	152	0.588	1.03	-61	0.208	3.72	92	0.631	Protein A	0
	HOH929	2.53	416	0.737	1.03	83	0.316	3.57	499	0.872	Both	5
	HOH1104	1.07	-54	0.220	0.92	244	0.340	2.00	191	0.536	Protein B	18
	HOH1648	2.77	394	0.748	2.54	372	0.713	5.30	766	1.062	Both	19
	HOH1666	1.14	-289	-0.408	1.04	16	0.268	2.18	-274	-0.370	Protein B	2
	HOH1700	2.60	-83	0.314	2.61	136	0.558	5.21	53	0.699	Both	1
	HOH1720	2.39	43	0.424	1.14	1	0.271	3.52	44	0.563	Both	32
	HOH1775	2.64	456	0.766	0.99	-99	0.169	3.63	357	0.821	Protein A	22
	HOH1776	2.59	61	0.470	2.63	189	0.623	5.22	250	0.920	Both	1
	HOH1783	2.16	451	0.733	1.25	155	0.404	3.40	607	0.892	Both	22
	HOH1802	3.87	39	0.609	1.15	-4	0.266	5.01	34	0.673	Both	0
	HOH1811	1.35	538	0.654	1.20	131	0.383	2.55	669	0.835	Both	5
	HOH1880	2.02	-144	-0.072	1.00	46	0.284	3.03	-98	0.354	Protein B	16
	HOH1989	2.37	319	0.665	1.10	54	0.306	3.47	372	0.811	Both	14
	HOH2029	1.11	132	0.357	1.07	28	0.282	2.18	160	0.531	Both	10

	HOH2046	1.31	113	0.390	2.18	91	0.447	3.48	204	0.756	Both	1
	HOH2048	2.32	161	0.551	1.02	-5	0.249	3.34	156	0.674	Protein A	9
	HOH2049	2.31	133	0.516	0.99	74	0.302	3.31	207	0.735	Both	19
	HOH2051	2.32	63	0.437	1.05	-272	-0.366	3.37	-209	-0.216	Protein A	2
	HOH2095	1.17	222	0.390	1.12	92	0.338	2.29	313	0.654	Both	0
	HOH2168	0.75	-68	0.152	0.00	-124	-0.037	0.75	-192	-0.178	Neither	18
	HOH2253	1.29	-7	0.269	2.25	-106	0.221	3.54	-112	0.005	Protein A	14
	HOH2272	2.76	44	0.473	2.97	131	0.592	5.74	175	0.902	Both	22
	HOH2273	0.00	-164	-0.087	1.01	124	0.336	1.01	-40	0.221	Protein B	10
	HOH2278	1.05	-103	0.175	2.06	10	0.333	3.11	-93	0.371	Protein B	8
	HOH2283	0.94	79	0.298	1.11	-129	-0.038	2.05	-50	0.250	Protein A	8
	HOH2284	0.94	-238	-0.286	1.00	77	0.306	1.93	-161	-0.110	Protein B	11
	HOH2286	1.14	-234	-0.274	1.15	105	0.353	2.29	-129	-0.038	Protein B	10
	HOH2288	1.21	30	0.301	2.26	136	0.514	3.47	166	0.707	Both	6
	HOH2289	0.00	-27	-0.039	3.54	10	0.533	3.54	-17	0.508	Protein B	18
	HOH2296	1.19	-36	0.239	3.73	114	0.658	4.92	78	0.706	Protein B	11
	HOH2298	0.00	-57	-0.039	3.06	64	0.527	3.06	7	0.471	Protein B	0
	HOH2306	0.00	-113	-0.030	1.93	8	0.312	1.93	-105	0.154	Protein B	9
	HOH2310	0.00	-282	-0.369	1.99	-101	0.163	1.99	-383	-0.646	Neither	31
	HOH2311	2.21	89	0.450	1.25	-209	-0.218	3.46	-120	-0.015	Protein A	40
	HOH2319	2.14	384	0.692	1.27	119	0.389	3.41	503	0.859	Both	15
	HOH2325	0.88	48	0.265	0.92	-104	0.152	1.80	-56	0.222	Protein A	21
	HOH2331	0.86	25	0.244	0.94	11	0.248	1.80	36	0.343	Neither	23
	HOH2337	1.12	183	0.364	2.15	-102	0.205	3.27	82	0.567	Protein A	45
	HOH2343	0.00	-115	-0.031	0.84	42	0.253	0.84	-73	0.165	Protein B	29
	HOH2346	2.56	112	0.520	1.44	-170	-0.129	4.00	-58	0.528	Protein A	16
	HOH2359	0.82	-33	0.192	0.82	8	0.224	1.63	-25	0.254	Neither	2
	HOH2371	1.05	-20	0.244	1.94	135	0.473	2.99	115	0.572	Protein B	13
	HOH2391	0.84	-59	0.175	1.15	163	0.376	1.99	105	0.438	Protein B	9
1LK3	HOH302	1.17	-118	-0.014	0.87	0	0.227	2.04	-118	-0.016	Neither	0
	HOH305	1.06	-176	-0.142	0.79	-147	-0.077	1.85	-323	-0.491	Neither	1
	HOH360	2.09	8	0.336	0.00	-80	-0.040	2.09	-71	0.232	Protein A	15
	HOH657	2.77	122	0.557	2.63	412	0.744	5.40	534	1.016	Both	7
	HOH659	1.03	-105	0.171	2.41	143	0.542	3.45	38	0.548	Protein B	26
	HOH660	0.00	-163	-0.085	3.20	94	0.570	3.20	-69	0.408	Protein B	15
	HOH661	1.12	-31	0.243	1.16	89	0.344	2.28	58	0.425	Protein B	14
	HOH662	0.98	148	0.334	1.20	97	0.359	2.18	245	0.596	Both	0
	HOH663	0.91	47	0.269	0.95	158	0.329	1.86	205	0.529	Both	6
	HOH665	1.14	120	0.362	2.36	176	0.572	3.50	297	0.782	Both	9
	HOH666	2.84	38	0.476	2.73	34	0.460	5.57	72	0.728	Both	46
	HOH667	1.23	6	0.280	2.34	-52	0.308	3.57	-45	0.485	Both	14
	HOH668	1.09	-26	0.245	3.49	-28	0.491	4.58	-54	0.570	Protein B	24
	HOH671	0.00	-177	-0.111	2.38	557	0.789	2.38	380	0.704	Protein B	10
	HOH677	0.00	-265	-0.324	0.91	124	0.316	0.91	-141	-0.064	Protein B	1
	HOH768	1.10	-17	0.254	0.99	-10	0.240	2.09	-27	0.291	Protein A	1
	HOH797	1.20	219	0.402	1.12	-218	-0.239	2.32	0	0.366	Protein A	6
	HOH931	2.23	219	0.586	0.97	-8	0.238	3.20	212	0.720	Protein A	11
	HOH999	2.17	46	0.396	1.04	62	0.302	3.21	108	0.587	Both	44
	HOH1010	2.60	75	0.486	2.55	-141	-0.064	5.15	-66	0.589	Protein A	18
	HOH1013	3.85	66	0.629	1.36	42	0.325	5.21	109	0.750	Both	8
	HOH1026	0.72	-55	0.156	0.00	-134	-0.046	0.72	-190	-0.173	Neither	30
	HOH1169	2.45	86	0.478	1.21	-27	0.247	3.65	59	0.594	Protein A	24
1NF3	HOH6	1.23	-233	-0.274	1.17	3	0.274	2.40	-230	-0.267	Protein B	3
	HOH11	3.63	62	0.594	1.46	24	0.308	5.09	86	0.720	Both	19
	HOH20	3.69	478	0.876	0.00	-70	-0.039	3.69	408	0.849	Protein A	9
	HOH37	2.74	350	0.722	1.37	-180	-0.152	4.10	170	0.788	Protein A	16
	HOH40	2.59	9	0.415	1.24	-106	0.163	3.83	-96	0.477	Protein A	14
	HOH64	0.81	48	0.252	0.00	-70	-0.039	0.81	-22	0.200	Protein A	36
	HOH223	2.51	127	0.534	1.41	126	0.417	3.93	253	0.819	Both	5
	HOH232	1.26	151	0.404	2.33	-507	-0.989	3.58	-356	-0.576	Protein A	14

	HOH237	3.53	108	0.624	1.11	-71	0.211	4.64	37	0.656	Protein A	34
	HOH239	0.00	-182	-0.122	1.08	118	0.345	1.08	-64	0.213	Protein B	56
	HOH241	0.00	-503	-0.975	1.04	2	0.258	1.04	-501	-0.972	Protein B	31
	HOH264	0.00	-340	-0.522	0.86	-90	0.154	0.86	-430	-0.773	Neither	23
	HOH278	0.00	-172	-0.102	0.77	-184	-0.160	0.77	-356	-0.577	Neither	0
	HOH300	0.00	0	-0.039	1.05	113	0.337	1.05	113	0.337	Protein B	11
	HOH305	0.00	-145	-0.058	1.06	106	0.335	1.06	-39	0.229	Protein B	17
	HOH313	0.00	-531	-1.058	0.86	67	0.275	0.86	-465	-0.869	Protein B	36
1NMB	HOH51	0.00	-501	-0.968	0.92	3	0.239	0.92	-497	-0.961	Neither	20
	HOH54	1.01	11	0.259	2.21	-87	0.238	3.22	-77	0.402	Protein A	5
	HOH55	0.99	-245	-0.301	1.04	-471	-0.885	2.03	-715	-1.633	Neither	3
	HOH62	1.13	-250	-0.313	1.30	134	0.405	2.43	-116	-0.009	Protein B	23
	HOH63	1.05	4	0.260	2.29	48	0.415	3.33	52	0.546	Both	26
	HOH73	2.29	212	0.590	1.27	97	0.371	3.56	309	0.793	Both	9
	HOH76	3.46	42	0.553	0.00	-259	-0.308	3.46	-217	-0.235	Protein A	0
	HOH78	2.89	29	0.473	2.61	44	0.455	5.50	73	0.726	Both	10
1O94	HOH401	2.02	-52	0.239	0.00	-234	-0.244	2.02	-286	-0.400	Neither	3
	HOH452	1.01	-99	0.172	0.88	153	0.314	1.89	54	0.371	Protein B	41
	HOH454	1.08	106	0.338	0.00	-169	-0.096	1.08	-63	0.213	Protein A	9
	HOH458	2.18	-9	0.331	0.00	-447	-0.813	2.18	-455	-0.842	Protein A	14
	HOH459	1.69	63	0.370	0.00	-301	-0.418	1.69	-238	-0.284	Protein A	29
	HOH461	0.98	81	0.305	0.00	4	-0.039	0.98	85	0.307	Protein A	25
	HOH462	1.39	213	0.456	2.51	-172	-0.133	3.90	41	0.610	Protein A	27
	HOH467	1.13	131	0.363	1.09	19	0.279	2.22	151	0.525	Both	16
	HOH468	1.27	77	0.352	2.29	74	0.444	3.56	151	0.695	Both	23
	HOH479	4.22	-20	0.579	1.28	14	0.290	5.50	-6	0.658	Both	31
	HOH481	1.15	61	0.320	0.88	-113	-0.003	2.03	-52	0.243	Protein A	16
	HOH1573	0.87	84	0.288	0.84	-336	-0.525	1.71	-252	-0.318	Protein A	15
	HOH1619	0.00	-30	-0.039	0.74	29	0.226	0.74	-1	0.203	Neither	5
	HOH1627	0.82	79	0.276	0.93	-17	0.225	1.75	62	0.373	Protein A	17
	HOH1628	1.16	93	0.348	1.17	61	0.324	2.33	154	0.544	Both	27
	HOH1699	1.11	-151	-0.086	1.16	47	0.311	2.28	-104	0.230	Protein B	0
	HOH1700	1.05	-76	0.197	2.06	37	0.366	3.11	-40	0.428	Protein B	27
	HOH1703	1.16	102	0.353	1.19	-88	0.188	2.35	14	0.386	Protein A	19
	HOH1704	0.00	-82	-0.040	1.01	136	0.339	1.01	53	0.291	Protein B	1
	HOH1705	2.11	-30	0.290	1.35	192	0.437	3.46	162	0.698	Both	17
1OKK	HOH67	0.90	-16	0.221	0.78	-9	0.204	1.68	-25	0.257	Neither	46
	HOH70	2.55	215	0.629	1.02	-153	-0.090	3.57	62	0.586	Protein A	7
	HOH72	1.40	461	0.640	2.36	-152	-0.088	3.75	310	0.816	Protein A	5
	HOH80	2.68	462	0.773	1.24	-250	-0.313	3.91	212	0.816	Protein A	0
	HOH81	1.02	-214	-0.229	0.00	-188	-0.134	1.02	-402	-0.697	Neither	60
	HOH137	0.75	-112	-0.003	0.00	-159	-0.079	0.75	-272	-0.365	Neither	33
	HOH179	0.83	-554	-1.127	0.00	-130	-0.041	0.83	-684	-1.530	Neither	8
	HOH200	2.53	2	0.400	1.15	210	0.377	3.69	212	0.787	Both	3
	HOH203	4.20	16	0.609	1.18	-196	-0.187	5.37	-180	-0.150	Protein A	31
	HOH204	1.15	520	0.529	0.80	-168	-0.124	1.95	352	0.665	Protein A	9
	HOH205	1.09	227	0.366	0.00	-309	-0.441	1.09	-82	0.200	Protein A	4
	HOH206	1.24	-220	-0.243	1.90	-322	-0.490	3.13	-542	-1.091	Neither	28
	HOH284	2.43	-70	0.303	1.10	20	0.283	3.54	-49	0.476	Both	10
	HOH288	2.06	464	0.742	0.96	-158	-0.101	3.01	307	0.730	Protein A	11
	HOH289	2.45	310	0.668	1.31	-52	0.218	3.77	258	0.803	Protein A	32
	HOH321	1.38	572	0.680	3.56	-240	-0.290	4.94	331	0.920	Protein A	0
	HOH323	1.02	232	0.354	0.90	-2	0.232	1.93	230	0.558	Protein A	1
	HOH325	1.13	407	0.479	1.02	-325	-0.496	2.16	82	0.434	Protein A	0
	HOH327	0.98	289	0.379	0.00	-209	-0.183	0.98	80	0.304	Protein A	7
	HOH342	1.06	174	0.350	0.99	-15	0.236	2.05	159	0.513	Protein A	34
	HOH375	1.31	160	0.420	2.70	265	0.672	4.01	425	0.886	Both	4
	HOH376	0.00	-39	-0.039	3.62	196	0.768	3.62	157	0.712	Protein B	1
	HOH378	1.25	-246	-0.304	2.67	285	0.678	3.92	39	0.609	Protein B	44
	HOH380	1.11	129	0.356	1.21	-412	-0.724	2.32	-283	-0.393	Protein A	17

	HOH381	0.87	138	0.312	1.10	-122	-0.023	1.97	16	0.324	Protein A	2
	HOH422	1.19	154	0.387	2.46	-76	0.300	3.65	79	0.611	Both	0
	HOH423	0.00	-190	-0.139	3.90	66	0.631	3.90	-124	-0.021	Protein B	29
	HOH449	0.00	-130	-0.042	0.98	247	0.353	0.98	117	0.326	Protein B	7
	HOH486	1.03	67	0.303	1.19	-5	0.268	2.22	61	0.420	Both	15
	HOH507	1.15	-145	-0.072	1.16	111	0.361	2.31	-33	0.325	Protein B	38
	HOH509	1.08	-232	-0.271	4.06	-684	-1.531	5.14	-916	-2.337	Neither	43
	HOH512	0.97	-30	0.222	2.19	-10	0.332	3.16	-40	0.434	Protein B	46
	HOH513	2.16	-179	-0.149	1.28	-23	0.251	3.43	-202	-0.201	Protein B	15
	HOH515	1.23	446	0.553	1.27	128	0.396	2.50	574	0.802	Both	7
	HOH531	0.00	-401	-0.688	1.05	119	0.341	1.05	-282	-0.390	Protein B	8
	HOH532	0.00	-216	-0.200	1.09	404	0.457	1.09	188	0.354	Protein B	23
	HOH534	0.00	-217	-0.203	1.98	151	0.495	1.98	-67	0.209	Protein B	8
	HOH536	1.15	393	0.487	1.22	-50	0.224	2.37	343	0.680	Protein A	31
	HOH546	3.91	-278	-0.381	1.51	494	0.690	5.42	216	0.936	Protein B	22
	HOH549	2.22	-271	-0.363	1.46	593	0.719	3.67	322	0.812	Protein B	5
	HOH564	0.00	16	-0.039	3.81	96	0.647	3.81	112	0.665	Protein B	2
	HOH565	0.00	-143	-0.056	0.72	65	0.248	0.72	-79	0.138	Neither	0
	HOH568	0.97	-42	0.212	1.94	57	0.376	2.90	15	0.461	Protein B	18
1ORS	HOH329	1.03	186	0.341	0.00	-493	-0.945	1.03	-306	-0.450	Protein A	1
	HOH226	2.28	35	0.399	1.23	62	0.333	3.51	97	0.609	Both	18
	HOH232	2.16	-285	-0.398	0.00	-254	-0.296	2.16	-539	-1.082	Neither	20
	HOH303	0.89	52	0.270	0.88	-52	0.189	1.78	1	0.296	Protein A	20
	HOH319	2.68	767	0.865	0.99	-79	0.186	3.67	688	0.933	Protein A	5
	HOH152	2.86	-54	0.381	2.72	-37	0.382	5.58	-91	0.580	Both	38
1OSP	HOH215	1.23	-88	0.183	1.19	157	0.388	2.42	68	0.456	Protein B	0
	HOH220	3.63	214	0.780	0.00	-344	-0.534	3.63	-131	-0.038	Protein A	0
	HOH221	0.99	-106	0.162	0.00	-351	-0.552	0.99	-457	-0.848	Neither	8
	HOH224	0.95	283	0.370	0.00	-100	-0.040	0.95	183	0.325	Protein A	1
	HOH234	2.31	-92	0.251	0.00	-59	-0.039	2.31	-151	-0.087	Protein A	17
	HOH249	1.15	205	0.374	0.88	-79	0.167	2.04	125	0.471	Protein A	24
	HOH257	3.52	19	0.540	0.00	-56	-0.039	3.52	-37	0.486	Protein A	6
	HOH259	2.13	144	0.505	0.00	-159	-0.078	2.13	-15	0.313	Protein A	3
	HOH279	1.97	129	0.469	0.97	101	0.317	2.94	230	0.689	Both	21
	HOH277	1.11	-60	0.220	2.24	-45	0.297	3.35	-105	0.390	Protein B	37
	HOH289	1.00	-161	-0.109	2.12	95	0.445	3.13	-66	0.402	Protein B	1
	HOH292	1.13	-144	-0.072	2.20	15	0.363	3.32	-129	-0.036	Protein B	1
	HOH298	2.07	55	0.389	1.34	339	0.546	3.41	394	0.814	Both	29
	HOH299	0.00	-66	-0.039	0.99	-195	-0.185	0.99	-261	-0.339	Neither	21
	HOH307	1.08	2	0.264	2.30	161	0.548	3.37	163	0.689	Both	21
	HOH313	1.00	116	0.331	1.18	-406	-0.706	2.18	-289	-0.408	Protein A	3
	HOH345	0.00	21	-0.039	0.99	427	0.445	0.99	448	0.452	Protein B	35
	HOH347	0.00	2	-0.039	3.46	-92	0.421	3.46	-90	0.423	Protein B	5
	HOH352	2.04	-48	0.252	1.16	48	0.312	3.21	0	0.481	Both	11
	HOH359	4.01	164	0.769	1.40	-60	0.205	5.41	104	0.751	Protein A	1
	HOH361	0.93	0	0.239	1.90	76	0.399	2.84	76	0.514	Protein B	19
	HOH372	1.07	-112	-0.001	3.83	297	0.821	4.90	184	0.875	Protein B	12
	HOH382	0.97	-14	0.234	1.19	354	0.487	2.16	340	0.663	Protein B	35
1OW3	HOH380	1.17	-95	0.184	0.86	-19	0.212	2.03	-114	-0.006	Neither	26
	HOH381	1.34	80	0.364	2.22	85	0.446	3.56	165	0.717	Both	0
	HOH386	1.84	-370	-0.613	0.00	1	-0.039	1.84	-369	-0.609	Neither	0
	HOH396	3.58	56	0.582	0.00	-180	-0.117	3.58	-124	-0.022	Protein A	0
	HOH407	3.52	143	0.676	1.27	-125	-0.030	4.79	18	0.648	Protein A	0
	HOH408	0.98	-224	-0.253	0.00	-252	-0.290	0.98	-476	-0.901	Neither	0
	HOH436	2.53	15	0.413	1.02	-85	0.186	3.55	-70	0.456	Protein A	0
	HOH439	0.94	-95	0.163	0.00	-164	-0.086	0.94	-259	-0.334	Neither	31
	HOH448	0.00	-206	-0.176	0.00	-171	-0.099	0.00	-377	-0.622	Neither	2
	HOH453	2.39	95	0.480	1.15	12	0.281	3.54	107	0.625	Both	4
	HOH467	2.56	79	0.485	1.01	-255	-0.326	3.57	-176	-0.141	Protein A	20
	HOH477	1.25	201	0.410	1.19	25	0.295	2.44	227	0.618	Both	2

	HOH510	1.06	-41	0.228	1.04	-188	-0.168	2.10	-228	-0.262	Neither	20
	HOH518	3.01	-92	0.358	1.44	314	0.558	4.45	222	0.867	Both	0
	HOH536	0.86	-37	0.197	0.00	-14	-0.039	0.86	-52	0.186	Neither	31
	HOH684	1.24	26	0.300	3.74	-68	0.490	4.99	-42	0.603	Both	19
	HOH687	2.48	-1	0.390	2.66	22	0.438	5.14	22	0.668	Both	0
	HOH691	2.53	65	0.467	1.35	9	0.287	3.89	74	0.637	Both	1
	HOH698	1.20	193	0.391	2.38	-12	0.361	3.58	180	0.742	Both	22
	HOH702	2.08	-221	-0.246	1.17	219	0.392	3.25	-2	0.484	Protein B	0
	HOH707	1.18	-121	-0.021	1.31	540	0.634	2.48	419	0.734	Protein B	19
	HOH711	0.96	-50	0.203	2.27	269	0.622	3.23	219	0.724	Protein B	0
	HOH713	2.30	101	0.475	1.41	223	0.469	3.71	323	0.816	Both	1
	HOH722	1.12	76	0.327	1.17	25	0.294	2.29	101	0.474	Both	33
	HOH726	1.05	-34	0.231	3.67	96	0.628	4.71	62	0.681	Protein B	0
	HOH740	1.10	-47	0.230	1.16	108	0.358	2.26	61	0.426	Protein B	0
	HOH745	1.01	-25	0.232	2.31	298	0.645	3.32	273	0.751	Protein B	2
	HOH747	0.98	-61	0.198	2.45	463	0.753	3.43	402	0.819	Protein B	16
	HOH751	0.00	-226	-0.225	1.02	-56	0.209	1.02	-282	-0.391	Neither	38
	HOH757	0.84	-518	-1.020	1.01	350	0.418	1.85	-168	-0.124	Protein B	0
	HOH772	0.95	44	0.274	0.95	157	0.328	1.90	201	0.532	Both	31
	HOH773	0.84	43	0.253	1.02	214	0.341	1.86	257	0.573	Both	39
	HOH775	1.21	303	0.468	1.25	86	0.358	2.47	389	0.716	Both	6
	HOH778	0.92	65	0.283	0.94	-182	-0.157	1.86	-118	-0.016	Protein A	6
	HOH782	0.00	-22	-0.039	0.00	15	-0.039	0.00	-7	-0.039	Neither	29
	HOH783	1.04	146	0.347	1.11	-253	-0.321	2.15	-107	0.196	Protein A	5
	HOH787	1.07	57	0.303	2.24	63	0.425	3.31	120	0.614	Both	8
	HOH796	0.95	15	0.253	2.02	-167	-0.123	2.97	-152	-0.088	Protein A	2
	HOH834	1.06	-318	-0.480	1.32	538	0.640	2.38	219	0.607	Protein B	17
1OY3	HOH9	3.76	445	0.870	1.34	100	0.383	5.10	545	1.004	Both	36
	HOH13	3.89	89	0.650	1.21	-23	0.251	5.10	66	0.704	Both	31
	HOH14	2.05	482	0.753	1.37	180	0.439	3.41	662	0.906	Both	7
	HOH15	0.74	-198	-0.192	0.00	-186	-0.130	0.74	-384	-0.648	Neither	21
	HOH34	1.19	-45	0.231	1.17	64	0.326	2.36	19	0.394	Protein B	0
	HOH55	1.18	164	0.387	0.87	-227	-0.258	2.05	-62	0.233	Protein A	22
	HOH77	1.13	-70	0.210	0.86	-23	0.208	1.99	-93	0.174	Neither	0
	HOH79	2.15	-242	-0.294	0.00	-28	-0.039	2.15	-270	-0.361	Neither	2
	HOH84	1.15	17	0.284	1.13	169	0.368	2.28	186	0.570	Both	8
	HOH89	1.05	-38	0.228	0.94	4	0.242	1.98	-34	0.257	Neither	42
	HOH112	2.24	397	0.704	0.99	-73	0.191	3.23	325	0.763	Protein A	13
	HOH121	1.19	530	0.562	0.97	158	0.332	2.16	688	0.830	Both	23
	HOH125	2.35	263	0.628	1.01	172	0.340	3.37	435	0.828	Both	17
	HOH134	0.00	-357	-0.568	3.78	135	0.697	3.78	-222	-0.247	Protein B	1
	HOH145	0.98	-47	0.210	0.99	-260	-0.337	1.98	-307	-0.451	Neither	0
	HOH147	0.00	202	-0.039	3.60	-370	-0.613	3.60	-168	-0.124	Neither	40
	HOH148	1.29	83	0.360	3.86	-324	-0.494	5.15	-241	-0.291	Protein A	4
	HOH153	1.25	82	0.354	2.37	-349	-0.558	3.62	-267	-0.353	Protein A	27
	HOH154	1.32	167	0.424	2.37	-404	-0.703	3.69	-238	-0.284	Protein A	15
	HOH156	3.63	436	0.855	1.43	92	0.384	5.06	529	0.997	Both	9
	HOH165	1.24	-29	0.245	1.96	-422	-0.751	3.20	-451	-0.831	Neither	30
	HOH182	1.33	-578	-1.199	2.36	-291	-0.412	3.69	-869	-2.163	Neither	1
	HOH204	0.00	140	-0.038	1.02	438	0.455	1.02	578	0.493	Protein B	17
	HOH211	1.06	-146	-0.076	2.39	-64	0.302	3.45	-210	-0.219	Protein B	5
1PXV	HOH27	2.32	-196	-0.189	1.33	-41	0.231	3.64	-237	-0.282	Neither	39
	HOH35	1.95	-28	0.264	0.00	-84	-0.040	1.95	-112	-0.003	Protein A	5
	HOH36	1.20	-224	-0.252	1.20	81	0.345	2.39	-143	-0.070	Protein B	28
	HOH41	2.22	158	0.534	0.00	-211	-0.186	2.22	-52	0.284	Protein A	15
	HOH76	1.45	-118	-0.017	4.10	159	0.767	5.55	40	0.701	Protein B	10
	HOH85	2.62	180	0.614	1.16	-216	-0.234	3.78	-36	0.528	Protein A	16
	HOH91	0.96	331	0.399	0.00	-266	-0.326	0.96	65	0.291	Protein A	29
	HOH94	2.67	511	0.792	1.04	24	0.275	3.71	535	0.897	Both	15
	HOH106	0.71	31	0.223	0.00	-232	-0.239	0.71	-201	-0.198	Neither	0

	HOH136	2.35	-113	-0.003	0.00	-361	-0.578	2.35	-474	-0.895	Neither	0
	HOH161	3.29	276	0.748	0.00	-172	-0.102	3.29	103	0.590	Protein A	8
	HOH269	2.10	-45	0.269	0.94	18	0.253	3.04	-27	0.433	Both	0
	HOH317	2.30	122	0.501	1.23	98	0.366	3.54	220	0.766	Both	0
	HOH329	2.13	168	0.533	0.00	-84	-0.040	2.13	85	0.434	Protein A	10
	HOH352	4.37	-427	-0.765	1.51	-11	0.268	5.88	-438	-0.794	Protein B	13
	HOH353	1.28	142	0.406	3.67	15	0.557	4.95	157	0.826	Both	12
	HOH354	0.00	-27	-0.039	1.99	-363	-0.594	1.99	-390	-0.666	Neither	25
	HOH356	0.00	-280	-0.364	5.55	208	0.945	5.55	-72	0.598	Protein B	9
	HOH358	4.16	133	0.727	1.52	95	0.395	5.67	228	0.945	Both	10
	HOH361	1.03	-27	0.234	2.54	-219	-0.240	3.57	-246	-0.303	Neither	15
	HOH363	2.26	216	0.587	1.30	228	0.443	3.55	443	0.849	Both	26
	HOH367	0.91	-151	-0.086	1.20	-21	0.254	2.10	-171	-0.132	Protein B	3
	HOH368	0.00	-6	-0.039	1.01	-422	-0.750	1.01	-428	-0.766	Neither	25
	HOH375	0.89	-100	0.152	0.99	-133	-0.047	1.88	-233	-0.273	Neither	5
	HOH384	1.05	38	0.286	1.06	-211	-0.222	2.11	-174	-0.138	Protein A	0
	HOH401	1.03	57	0.297	1.04	-114	-0.004	2.07	-57	0.247	Protein A	36
	HOH402	1.17	236	0.403	3.54	251	0.771	4.71	487	0.962	Both	13
1Q40	HOH3	3.87	357	0.848	0.00	-34	-0.039	3.87	323	0.835	Protein A	3
	HOH5	3.52	100	0.614	0.00	82	-0.038	3.52	182	0.736	Protein A	13
	HOH7	1.31	57	0.336	2.28	-163	-0.114	3.59	-106	0.426	Protein A	0
	HOH14	2.35	-1	0.370	0.00	-319	-0.468	2.35	-321	-0.486	Protein A	23
	HOH17	1.40	-35	0.236	3.68	122	0.663	5.08	86	0.720	Protein B	6
	HOH18	1.12	-267	-0.354	1.06	-109	0.172	2.18	-376	-0.628	Neither	25
	HOH19	2.07	187	0.542	0.00	-245	-0.273	2.07	-58	0.244	Protein A	16
	HOH20	3.62	210	0.778	0.00	-240	-0.260	3.62	-30	0.507	Protein A	3
	HOH23	2.19	53	0.407	1.04	-139	-0.059	3.24	-85	0.395	Protein A	4
	HOH31	2.85	398	0.759	1.25	94	0.365	4.10	492	0.918	Both	6
	HOH32	1.05	-65	0.206	0.00	-235	-0.246	1.05	-300	-0.434	Neither	3
	HOH48	0.83	47	0.255	0.00	-188	-0.133	0.83	-141	-0.064	Protein A	24
	HOH57	0.78	-110	0.003	0.00	-178	-0.114	0.78	-288	-0.406	Neither	1
	HOH75	2.66	-506	-0.985	2.52	-325	-0.498	5.19	-831	-2.029	Neither	29
	HOH76	1.23	-336	-0.525	2.18	-51	0.279	3.42	-387	-0.657	Protein B	38
	HOH81	0.00	-288	-0.384	3.89	89	0.650	3.89	-199	-0.193	Protein B	4
	HOH85	3.94	-152	-0.085	1.42	45	0.331	5.36	-107	0.556	Protein B	0
	HOH90	1.33	40	0.320	2.79	186	0.643	4.12	225	0.837	Both	4
	HOH97	0.00	-237	-0.252	5.12	-401	-0.695	5.12	-638	-1.384	Neither	14
	HOH102	1.16	-36	0.239	2.19	263	0.609	3.34	227	0.741	Protein B	53
	HOH103	1.12	-48	0.229	1.23	-8	0.266	2.34	-56	0.304	Protein B	0
	HOH111	1.02	-163	-0.113	2.13	-21	0.306	3.15	-184	-0.159	Protein B	30
	HOH115	0.00	-149	-0.063	1.93	-176	-0.144	1.93	-325	-0.497	Neither	4
	HOH121	1.27	99	0.372	2.60	26	0.434	3.87	124	0.692	Both	1
	HOH135	0.98	90	0.311	2.04	-53	0.245	3.02	38	0.497	Protein A	5
	HOH138	2.20	74	0.433	1.32	218	0.441	3.52	292	0.782	Both	4
	HOH158	2.58	125	0.539	3.09	-164	-0.114	5.67	-39	0.633	Protein A	3
	HOH265	0.00	-90	-0.040	1.02	-80	0.190	1.02	-170	-0.130	Neither	2
	HOH276	1.24	207	0.404	3.82	163	0.749	5.06	369	0.941	Both	0
1R8S	HOH511	1.97	95	0.424	0.00	-99	-0.040	1.97	-4	0.298	Protein A	1
	HOH514	3.99	270	0.828	0.00	-109	-0.040	3.99	161	0.762	Protein A	5
	HOH516	2.34	490	0.759	0.00	-333	-0.505	2.34	156	0.548	Protein A	4
	HOH522	3.48	-168	-0.123	0.00	-265	-0.323	3.48	-433	-0.780	Neither	0
	HOH534	2.51	-96	0.283	2.55	-172	-0.134	5.06	-268	-0.356	Protein A	0
	HOH538	0.00	-149	-0.064	0.00	-49	-0.039	0.00	-198	-0.156	Neither	6
	HOH539	2.37	6	0.381	2.52	99	0.500	4.89	105	0.729	Both	0
	HOH542	0.95	-188	-0.170	1.09	118	0.347	2.04	-71	0.219	Protein B	0
	HOH570	2.55	121	0.530	1.10	22	0.283	3.65	143	0.693	Both	14
	HOH574	2.65	-202	-0.202	0.97	-178	-0.146	3.61	-380	-0.639	Neither	11
	HOH578	1.25	-127	-0.034	2.10	-181	-0.154	3.35	-308	-0.453	Neither	30
	HOH579	4.06	476	0.909	1.30	114	0.389	5.36	590	1.030	Both	34
	HOH604	3.64	37	0.573	0.00	69	-0.038	3.64	106	0.636	Protein A	0

	HOH613	2.50	-114	-0.004	1.12	-190	-0.174	3.62	-304	-0.443	Neither	25
	HOH615	3.45	227	0.755	0.00	-158	-0.077	3.45	69	0.577	Protein A	5
	HOH620	1.11	-20	0.252	0.83	-23	0.202	1.94	-44	0.243	Protein A	7
	HOH621	2.31	71	0.444	0.96	-35	0.217	3.27	36	0.525	Protein A	9
	HOH637	4.03	228	0.827	1.39	95	0.383	5.41	323	0.943	Both	7
	HOH645	0.85	-184	-0.161	0.00	-156	-0.073	0.85	-340	-0.535	Neither	22
	HOH507	0.00	-311	-0.446	2.23	-196	-0.188	2.23	-507	-0.990	Neither	24
	HOH510	1.26	-223	-0.250	2.60	26	0.434	3.85	-197	-0.189	Protein B	16
	HOH515	1.29	-428	-0.768	4.28	430	0.910	5.57	2	0.668	Protein B	28
	HOH544	0.00	-122	-0.036	0.88	-59	0.183	0.88	-182	-0.155	Neither	13
	HOH555	2.87	95	0.535	2.69	-205	-0.209	5.56	-111	0.013	Protein A	13
	HOH564	2.15	112	0.468	1.03	34	0.279	3.17	145	0.637	Both	7
	HOH631	0.00	-22	-0.039	3.77	362	0.838	3.77	341	0.829	Protein B	34
	HOH670	0.81	-42	0.184	0.95	-186	-0.165	1.76	-228	-0.261	Neither	12
	HOH683	1.10	-144	-0.070	2.39	413	0.723	3.49	270	0.771	Protein B	16
	HOH685	1.01	12	0.260	1.21	557	0.580	2.21	568	0.788	Both	56
	HOH695	0.00	-107	-0.040	0.00	-57	-0.039	0.00	-163	-0.086	Neither	27
	HOH704	0.71	-53	0.157	0.00	-209	-0.183	0.71	-262	-0.343	Neither	13
1REW	HOH118	2.54	-622	-1.333	1.18	1	0.273	3.72	-621	-1.329	Protein B	0
	HOH119	2.38	28	0.406	1.24	-78	0.194	3.61	-50	0.486	Protein A	1
	HOH120	2.38	-125	-0.029	1.02	-259	-0.335	3.40	-384	-0.650	Neither	0
	HOH127	1.36	-127	-0.034	2.52	89	0.490	3.88	-38	0.538	Protein B	27
	HOH131	1.08	563	0.501	2.06	-153	-0.093	3.13	410	0.794	Protein A	15
	HOH143	4.01	6	0.588	1.21	-4	0.269	5.22	2	0.654	Both	2
	HOH162	1.07	-358	-0.582	1.01	20	0.266	2.08	-339	-0.532	Protein B	0
	HOH161	3.58	-47	0.484	1.17	171	0.381	4.74	124	0.751	Both	0
	HOH132	3.95	181	0.791	1.46	281	0.534	5.41	462	0.992	Both	9
	HOH135	0.00	-189	-0.136	3.50	-28	0.492	3.50	-217	-0.235	Protein B	0
	HOH140	1.07	-831	-2.030	1.14	120	0.361	2.21	-711	-1.620	Protein B	23
	HOH142	0.00	-24	-0.039	2.38	-52	0.316	2.38	-76	0.286	Protein B	36
	HOH145	1.03	350	0.422	1.05	-209	-0.218	2.08	141	0.496	Protein A	11
	HOH149	0.00	-196	-0.152	0.91	107	0.311	0.91	-89	0.164	Protein B	16
	HOH158	0.85	79	0.281	1.09	-145	-0.073	1.94	-66	0.211	Protein A	19
	HOH159	1.14	36	0.299	3.48	140	0.665	4.62	175	0.840	Both	5
1SLU	HOH144	1.09	-172	-0.132	0.90	-6	0.228	1.99	-177	-0.146	Neither	12
	HOH151	0.81	-59	0.170	0.00	-18	-0.039	0.81	-78	0.155	Neither	28
	HOH158	2.05	-172	-0.133	0.00	-31	-0.039	2.05	-203	-0.204	Neither	15
	HOH188	1.19	-255	-0.325	1.04	-374	-0.622	2.23	-629	-1.355	Neither	1
	HOH314	0.91	-306	-0.450	1.05	73	0.311	1.96	-233	-0.274	Protein B	13
1SQ2	HOH207	3.69	-94	0.456	1.37	-153	-0.093	5.07	-247	-0.306	Protein A	0
	HOH238	2.31	138	0.523	1.27	-54	0.217	3.59	84	0.608	Protein A	3
	HOH246	1.09	-68	0.212	0.86	-7	0.220	1.95	-75	0.197	Neither	0
	HOH252	2.48	163	0.575	2.65	113	0.531	5.13	276	0.917	Both	6
	HOH254	0.98	131	0.332	0.00	-103	-0.040	0.98	28	0.267	Protein A	0
	HOH257	1.15	87	0.340	1.14	-45	0.231	2.29	42	0.409	Protein A	11
	HOH267	2.45	53	0.444	1.07	-157	-0.099	3.52	-103	0.417	Protein A	34
	HOH270	2.40	348	0.686	0.00	-201	-0.164	2.40	147	0.544	Protein A	41
	HOH274	3.84	232	0.808	1.03	-4	0.252	4.86	228	0.899	Both	18
	HOH275	2.02	400	0.703	0.00	-193	-0.145	2.02	207	0.551	Protein A	17
	HOH310	0.90	119	0.313	0.81	29	0.238	1.71	148	0.468	Protein A	12
	HOH311	2.43	237	0.622	1.16	81	0.339	3.59	318	0.800	Both	4
	HOH205	1.16	95	0.349	1.16	-595	-1.250	2.32	-500	-0.968	Protein A	43
	HOH208	1.24	109	0.376	3.86	-377	-0.632	5.10	-269	-0.358	Protein A	15
	HOH224	2.24	3	0.356	1.35	236	0.468	3.59	239	0.776	Both	30
	HOH226	3.88	-71	0.507	1.46	7	0.288	5.34	-64	0.598	Both	12
	HOH248	0.95	11	0.250	1.23	335	0.495	2.18	346	0.668	Both	42
	HOH255	0.99	20	0.262	1.04	-126	-0.031	2.03	-107	0.165	Protein A	23
	HOH261	0.00	-26	-0.039	0.99	86	0.310	0.99	60	0.292	Protein B	31
	HOH282	1.99	10	0.318	1.15	2	0.272	3.14	12	0.484	Both	2
1T6G	HOH1	3.87	-41	0.538	1.16	-28	0.246	5.03	-69	0.579	Protein A	0

	HOH5	1.05	-32	0.235	0.00	-200	-0.160	1.05	-231	-0.268	Neither	6
	HOH9	1.23	88	0.356	1.10	7	0.273	2.34	95	0.473	Both	9
	HOH12	1.19	-227	-0.260	2.07	4	0.327	3.26	-223	-0.249	Protein B	2
	HOH18	2.42	525	0.778	1.15	20	0.287	3.57	546	0.887	Both	8
	HOH24	2.60	-124	-0.026	2.46	145	0.550	5.06	21	0.664	Protein B	14
	HOH28	4.31	216	0.856	1.29	251	0.461	5.61	467	1.002	Both	45
	HOH32	2.30	-8	0.354	1.27	-145	-0.074	3.58	-153	-0.090	Protein A	11
	HOH43	2.91	191	0.665	2.70	-164	-0.116	5.61	27	0.691	Protein A	2
	HOH51	2.32	-50	0.306	0.00	-36	-0.039	2.32	-86	0.262	Protein A	19
	HOH72	2.32	176	0.567	0.00	180	-0.039	2.32	356	0.684	Protein A	0
	HOH102	3.60	92	0.616	0.00	-46	-0.039	3.60	47	0.576	Protein A	0
	HOH135	2.35	-56	0.305	1.00	-48	0.212	3.35	-104	0.390	Protein A	31
	HOH137	2.10	-21	0.299	1.05	127	0.343	3.15	105	0.577	Both	0
	HOH138	1.13	93	0.342	1.02	-4	0.250	2.15	89	0.442	Both	20
	HOH139	1.19	156	0.388	1.11	60	0.314	2.31	216	0.594	Both	15
	HOH158	2.13	73	0.420	0.98	-109	0.005	3.11	-36	0.432	Protein A	0
	HOH198	2.06	-154	-0.094	0.00	2	-0.039	2.06	-152	-0.090	Neither	38
	HOH199	2.14	40	0.383	1.01	-57	0.208	3.15	-17	0.457	Protein A	15
	HOH243	1.20	-176	-0.143	0.88	-95	0.153	2.08	-271	-0.365	Neither	7
	HOH245	1.13	-223	-0.249	1.02	-169	-0.127	2.15	-392	-0.670	Neither	0
	HOH252	0.73	-280	-0.384	0.00	-22	-0.039	0.73	-302	-0.439	Neither	6
	HOH274	1.02	18	0.266	0.97	3	0.246	1.99	21	0.332	Protein A	0
	HOH287	1.11	330	0.433	0.81	-166	-0.121	1.93	163	0.504	Protein A	2
	HOH289	3.68	89	0.625	1.26	-11	0.263	4.94	78	0.707	Both	3
	HOH296	1.09	-52	0.225	0.93	-58	0.192	2.02	-110	0.001	Neither	0
	HOH304	3.27	-245	-0.302	0.00	-85	-0.040	3.27	-331	-0.511	Neither	0
	HOH659	2.72	-313	-0.467	2.37	229	0.610	5.09	-84	0.568	Protein B	0
	HOH664	1.31	26	0.304	3.86	566	0.919	5.17	592	1.021	Both	0
	HOH665	0.00	-99	-0.040	3.30	38	0.530	3.30	-61	0.431	Protein B	0
	HOH668	1.98	29	0.342	1.09	-67	0.213	3.07	-38	0.426	Protein A	2
	HOH676	0.00	-150	-0.064	2.18	-137	-0.056	2.18	-287	-0.401	Neither	12
	HOH684	0.88	-125	-0.030	1.19	262	0.430	2.07	137	0.490	Protein B	0
	HOH687	0.00	-222	-0.214	0.99	98	0.319	0.99	-124	-0.026	Protein B	1
	HOH691	3.62	-88	0.449	1.25	-64	0.207	4.87	-152	-0.086	Protein A	6
	HOH695	1.04	8	0.262	2.34	183	0.575	3.38	191	0.727	Both	0
	HOH702	0.00	-283	-0.371	1.91	-59	0.221	1.91	-342	-0.540	Neither	4
	HOH710	1.19	-245	-0.302	3.49	-87	0.431	4.68	-332	-0.514	Protein B	8
	HOH729	1.05	-42	0.226	1.12	317	0.427	2.17	275	0.616	Protein B	20
	HOH734	0.89	131	0.316	1.15	-125	-0.030	2.05	6	0.325	Protein A	7
	HOH738	2.06	-7	0.311	1.29	-22	0.252	3.35	-29	0.470	Both	1
	HOH739	2.27	-229	-0.264	1.35	190	0.438	3.62	-39	0.499	Protein B	0
	HOH740	0.87	1	0.228	0.93	-125	-0.030	1.80	-125	-0.030	Neither	4
	HOH743	0.99	-108	0.162	2.25	125	0.498	3.25	18	0.503	Protein B	6
	HOH744	1.02	-120	-0.019	2.29	133	0.514	3.31	13	0.506	Protein B	10
	HOH753	0.00	-21	-0.039	5.12	260	0.915	5.12	239	0.915	Protein B	22
	HOH755	0.98	-117	-0.011	2.20	266	0.612	3.18	150	0.644	Protein B	22
	HOH765	1.08	-198	-0.191	1.13	676	0.553	2.21	479	0.749	Protein B	17
	HOH769	2.05	-151	-0.087	1.11	16	0.280	3.16	-135	-0.048	Protein B	2
	HOH775	0.00	-6	-0.039	3.73	585	0.913	3.73	579	0.911	Protein B	7
	HOH783	0.00	-306	-0.431	2.16	139	0.504	2.16	-167	-0.122	Protein B	41
	HOH794	0.94	87	0.303	0.86	-69	0.171	1.81	18	0.321	Protein A	24
	HOH814	1.12	-19	0.253	2.17	162	0.531	3.28	142	0.645	Both	31
	HOH818	0.00	-69	-0.039	2.02	121	0.463	2.02	52	0.378	Protein B	0
	HOH826	0.00	-121	-0.035	1.90	-276	-0.377	1.90	-397	-0.684	Neither	0
1TA3	HOH6	1.20	43	0.312	1.12	-325	-0.496	2.32	-282	-0.391	Protein A	12
	HOH12	1.13	285	0.417	1.02	9	0.259	2.15	294	0.628	Both	2
	HOH14	2.33	75	0.451	0.00	-12	-0.039	2.33	63	0.438	Protein A	33
	HOH24	1.14	69	0.326	0.93	109	0.314	2.07	178	0.535	Both	23
	HOH25	3.42	174	0.711	0.00	-117	-0.032	3.42	56	0.562	Protein A	15
	HOH29	2.27	-33	0.318	0.00	-39	-0.039	2.27	-72	0.269	Protein A	0

	HOH37	1.35	94	0.378	2.45	168	0.577	3.80	262	0.808	Both	24
	HOH40	1.20	236	0.416	1.17	16	0.285	2.37	252	0.623	Both	2
	HOH49	3.74	-21	0.534	1.04	-10	0.250	4.78	-31	0.603	Both	24
	HOH54	1.34	144	0.420	2.36	175	0.572	3.71	319	0.814	Both	19
	HOH59	1.19	460	0.537	1.12	-22	0.250	2.31	438	0.731	Both	0
	HOH60	2.18	-259	-0.335	1.15	-63	0.215	3.33	-322	-0.490	Neither	0
	HOH78	1.44	-36	0.235	3.77	290	0.812	5.21	255	0.920	Protein B	52
	HOH86	2.27	4	0.362	1.03	29	0.276	3.29	33	0.524	Both	41
	HOH147	1.17	335	0.464	0.82	-33	0.194	1.99	303	0.628	Protein A	0
	HOH153	0.00	-29	-0.039	0.00	-30	-0.039	0.00	-60	-0.039	Neither	0
	HOH157	0.80	-39	0.185	0.00	-55	-0.039	0.80	-95	0.139	Neither	0
	HOH158	2.70	-174	-0.136	2.61	65	0.476	5.31	-109	0.553	Protein B	0
	HOH161	2.72	702	0.856	1.35	128	0.409	4.07	831	0.985	Both	0
	HOH165	2.50	51	0.449	1.03	27	0.275	3.53	78	0.596	Both	0
	HOH233	1.10	-138	-0.056	0.86	-52	0.185	1.96	-189	-0.173	Neither	6
	HOH308	0.80	40	0.244	0.00	-218	-0.203	0.80	-178	-0.146	Neither	4
	HOH349	1.15	27	0.293	0.88	-53	0.187	2.03	-25	0.279	Protein A	6
	HOH372	2.58	-25	0.376	1.25	-6	0.268	3.83	-32	0.540	Both	19
	HOH375	1.33	-29	0.244	4.13	608	0.954	5.45	579	1.031	Protein B	0
	HOH382	0.86	92	0.292	1.00	-39	0.220	1.87	52	0.368	Protein A	1
	HOH385	0.00	-188	-0.134	1.93	101	0.431	1.93	-87	0.181	Protein B	0
	HOH394	2.65	-71	0.335	2.98	294	0.719	5.63	223	0.944	Both	34
	HOH401	0.00	-86	-0.040	2.34	279	0.636	2.34	193	0.584	Protein B	29
	HOH407	0.00	-401	-0.688	0.99	-22	0.230	0.99	-424	-0.756	Neither	36
	HOH408	0.00	-197	-0.154	1.00	280	0.379	1.00	83	0.311	Protein B	7
	HOH410	1.14	10	0.278	2.81	572	0.827	3.95	583	0.932	Both	14
	HOH417	1.20	-46	0.229	4.12	143	0.742	5.32	98	0.740	Protein B	0
	HOH423	1.07	46	0.295	2.35	203	0.593	3.41	249	0.755	Both	26
	HOH448	1.91	29	0.339	1.18	73	0.335	3.08	102	0.565	Both	23
	HOH460	0.94	100	0.311	1.09	12	0.273	2.03	111	0.451	Both	3
	HOH463	1.09	29	0.287	3.52	93	0.606	4.61	122	0.739	Both	0
	HOH490	1.30	-17	0.257	2.76	538	0.811	4.07	521	0.924	Both	21
	HOH584	0.98	-17	0.233	2.06	12	0.334	3.03	-5	0.455	Protein B	18
	HOH604	0.00	0	-0.039	1.03	16	0.268	1.03	16	0.267	Protein B	10
	HOH609	0.00	-55	-0.039	0.73	-37	0.174	0.73	-92	0.129	Neither	0
	HOH616	0.00	-243	-0.268	3.29	125	0.620	3.29	-118	-0.010	Protein B	3
	HOH623	0.00	-276	-0.354	1.78	-120	-0.021	1.78	-397	-0.682	Neither	15
	HOH637	0.96	36	0.269	1.89	40	0.353	2.85	75	0.514	Both	9
	HOH643	0.00	-153	-0.069	1.86	0	0.300	1.86	-153	-0.092	Protein B	33
	HOH667	0.00	-203	-0.168	2.42	-113	-0.003	2.42	-316	-0.475	Neither	25
	HOH683	0.00	-165	-0.089	0.00	-94	-0.040	0.00	-260	-0.311	Neither	3
	HOH726	1.03	80	0.313	2.39	110	0.497	3.42	190	0.732	Both	24
	HOH728	1.12	18	0.282	1.22	596	0.601	2.34	614	0.807	Both	3
	HOH733	1.15	-252	-0.318	2.19	214	0.577	3.34	-38	0.459	Protein B	21
	HOH780	0.00	-89	-0.040	0.98	-5	0.242	0.98	-94	0.171	Neither	32
	HOH785	1.47	-615	-1.313	1.67	-1823	-6.517	3.15	-2438	-10.290	Neither	24
1TUE	HOH888	2.46	119	0.517	2.45	-78	0.295	4.91	41	0.674	Both	0
	HOH892	2.48	124	0.525	1.09	-23	0.247	3.57	101	0.620	Protein A	1
	HOH895	1.15	123	0.366	0.85	-19	0.210	2.01	105	0.441	Protein A	15
	HOH907	1.16	-75	0.204	1.03	81	0.315	2.19	6	0.351	Protein B	33
	HOH912	0.98	-5	0.243	0.82	-191	-0.177	1.81	-196	-0.189	Neither	2
	HOH917	1.07	-415	-0.732	0.87	-118	-0.013	1.94	-532	-1.063	Neither	2
	HOH922	1.18	-317	-0.477	1.13	414	0.483	2.31	97	0.472	Protein B	19
	HOH928	1.33	101	0.382	2.28	-98	0.237	3.60	3	0.536	Protein A	6
	HOH941	4.05	270	0.835	1.60	-107	0.140	5.65	163	0.874	Protein A	1
	HOH947	2.30	36	0.404	1.31	70	0.350	3.61	106	0.632	Both	23
	HOH949	0.93	88	0.301	1.10	170	0.357	2.03	258	0.592	Both	10
	HOH958	1.15	-74	0.205	2.32	-146	-0.076	3.47	-221	-0.244	Neither	3
	HOH959	2.18	14	0.359	1.30	136	0.406	3.48	151	0.682	Both	6
	HOH963	1.39	506	0.658	2.44	-83	0.288	3.83	423	0.869	Both	3

	HOH965	0.97	-182	-0.155	1.13	272	0.408	2.10	90	0.436	Protein B	20
	HOH968	0.00	-85	-0.040	0.89	-83	0.164	0.89	-168	-0.124	Neither	2
1TX4	HOH21	2.17	-309	-0.456	1.21	244	0.425	3.38	-65	0.437	Protein B	0
	HOH25	1.18	-88	0.190	0.87	-15	0.215	2.05	-102	0.176	Neither	19
	HOH41	3.59	40	0.569	0.00	-194	-0.148	3.59	-154	-0.092	Protein A	16
	HOH48	2.24	-102	0.225	1.17	-31	0.244	3.41	-133	-0.044	Neither	21
	HOH59	0.00	-224	-0.220	0.00	-179	-0.115	0.00	-403	-0.693	Neither	40
	HOH71	1.51	152	0.450	3.77	7	0.566	5.28	159	0.849	Both	0
	HOH72	2.52	87	0.488	1.01	-252	-0.319	3.54	-166	-0.118	Protein A	20
	HOH74	0.90	-109	0.144	0.00	-139	-0.051	0.90	-248	-0.310	Neither	0
	HOH233	1.27	26	0.302	2.30	15	0.379	3.57	41	0.566	Both	0
	HOH261	0.74	-403	-0.700	0.81	-196	-0.188	1.55	-599	-1.264	Neither	30
	HOH268	0.00	-232	-0.240	0.00	-187	-0.132	0.00	-420	-0.739	Neither	36
	HOH272	2.20	-445	-0.813	1.13	82	0.334	3.34	-362	-0.592	Protein B	5
	HOH274	1.12	24	0.287	2.37	-88	0.269	3.49	-65	0.453	Both	2
	HOH281	2.53	55	0.456	1.34	6	0.284	3.87	61	0.628	Both	2
	HOH284	1.30	7	0.283	2.41	16	0.397	3.70	22	0.569	Both	13
	HOH302	0.00	-116	-0.032	0.86	-44	0.191	0.86	-160	-0.107	Neither	0
	HOH305	1.07	225	0.359	0.95	-75	0.182	2.02	150	0.499	Protein A	4
	HOH310	1.12	-54	0.224	1.22	118	0.379	2.34	64	0.441	Protein B	0
	HOH315	0.99	-29	0.225	2.34	287	0.642	3.33	259	0.747	Protein B	2
	HOH317	1.16	159	0.377	1.21	-141	-0.064	2.36	19	0.394	Protein A	19
	HOH319	0.97	-40	0.213	2.48	458	0.753	3.45	419	0.829	Protein B	0
	HOH322	0.00	-21	-0.039	3.51	131	0.655	3.51	109	0.624	Protein B	11
	HOH324	1.11	228	0.370	1.26	-111	0.000	2.36	117	0.502	Protein A	9
	HOH326	1.06	-570	-1.174	1.32	562	0.649	2.38	-8	0.366	Protein B	0
	HOH327	0.98	9	0.253	2.00	-163	-0.114	2.97	-154	-0.092	Protein A	1
	HOH335	1.09	-344	-0.544	1.13	406	0.477	2.22	63	0.421	Protein B	18
	HOH337	1.12	-56	0.223	3.58	231	0.773	4.70	176	0.846	Protein B	11
	HOH358	0.00	-217	-0.201	1.85	-26	0.265	1.85	-243	-0.296	Protein B	4
	HOH361	1.08	62	0.309	2.20	-37	0.300	3.28	25	0.515	Both	18
	HOH362	2.66	16	0.432	1.40	250	0.493	4.06	266	0.835	Both	0
	HOH372	1.53	-71	0.189	4.29	291	0.861	5.82	219	0.953	Protein B	20
	HOH387	0.00	71	-0.038	0.89	-179	-0.149	0.89	-108	0.143	Neither	29
	HOH392	0.88	-216	-0.233	1.09	277	0.395	1.96	62	0.383	Protein B	24
	HOH393	1.05	92	0.325	1.11	-119	-0.016	2.16	-27	0.305	Protein A	42
	HOH401	0.00	-21	-0.039	0.81	59	0.260	0.81	38	0.245	Protein B	62
	HOH416	0.94	97	0.309	1.16	446	0.514	2.10	543	0.780	Both	1
	HOH423	0.00	-198	-0.155	1.01	-70	0.197	1.01	-267	-0.354	Neither	27
	HOH437	0.00	-228	-0.230	1.94	51	0.369	1.94	-177	-0.146	Protein B	0
	HOH452	0.00	-207	-0.178	0.75	-28	0.185	0.75	-235	-0.279	Neither	8
	HOH469	0.00	-201	-0.162	0.00	-187	-0.133	0.00	-388	-0.651	Neither	11
	HOH484	0.99	-6	0.243	2.20	487	0.753	3.19	481	0.830	Protein B	10
	HOH495	0.00	-351	-0.552	1.04	-117	-0.012	1.04	-468	-0.879	Neither	0
	HOH496	2.46	-127	-0.034	1.41	482	0.657	3.87	355	0.847	Protein B	13
	HOH497	0.00	-423	-0.749	1.27	613	0.639	1.27	190	0.416	Protein B	21
1TX6	HOH1	3.64	-215	-0.231	1.23	43	0.315	4.87	-172	-0.131	Protein B	0
	HOH42	2.59	83	0.492	2.60	-53	0.349	5.19	30	0.678	Both	19
	HOH52	1.59	-502	-0.975	0.00	-231	-0.238	1.59	-734	-1.694	Neither	3
	HOH105	0.93	5	0.242	0.85	-83	0.157	1.78	-78	0.189	Neither	0
	HOH113	3.87	-43	0.537	1.23	75	0.345	5.10	32	0.676	Both	15
	HOH126	4.98	-173	-0.133	0.00	21	-0.039	4.98	-152	-0.085	Neither	39
	HOH128	1.18	-123	-0.024	2.24	-203	-0.203	3.42	-325	-0.497	Neither	12
	HOH174	1.25	92	0.362	0.84	-50	0.182	2.08	42	0.375	Protein A	4
	HOH181	1.18	106	0.362	1.15	61	0.321	2.33	167	0.559	Both	2
	HOH182	1.22	58	0.328	1.14	32	0.295	2.36	90	0.470	Both	2
	HOH420	1.14	9	0.277	4.25	-104	0.500	5.38	-95	0.570	Both	28
	HOH425	0.85	-50	0.184	0.88	95	0.298	1.73	44	0.350	Protein B	39
	HOH428	0.00	-82	-0.040	1.82	105	0.429	1.82	23	0.327	Protein B	6
	HOH430	2.02	-3	0.308	1.17	-75	0.202	3.19	-79	0.397	Protein A	35

	HOH435	0.00	-35	-0.039	1.08	-105	0.178	1.08	-141	-0.063	Neither	12
	HOH436	1.27	11	0.286	2.46	-191	-0.176	3.73	-180	-0.150	Protein A	8
	HOH437	0.00	-44	-0.039	1.87	-6	0.293	1.87	-49	0.233	Protein B	36
	HOH438	0.94	-131	-0.041	1.12	339	0.443	2.06	208	0.556	Protein B	16
	HOH441	1.04	-25	0.238	2.40	268	0.636	3.44	244	0.757	Protein B	12
	HOH445	1.00	3	0.252	2.36	-55	0.308	3.36	-52	0.449	Both	19
1UNN	HOH77	4.11	604	0.951	0.00	-27	-0.039	4.11	577	0.944	Protein A	35
	HOH78	2.24	30	0.388	0.94	-511	-1.000	3.18	-481	-0.915	Protein A	7
	HOH79	2.35	-79	0.276	1.05	48	0.294	3.40	-31	0.474	Both	24
	HOH80	0.99	-131	-0.043	0.00	-291	-0.393	0.99	-423	-0.753	Neither	17
	HOH83	2.02	109	0.449	0.00	-142	-0.054	2.02	-33	0.268	Protein A	18
	HOH88	1.18	220	0.393	0.82	61	0.264	2.00	281	0.610	Both	10
	HOH101	0.89	149	0.317	0.84	-47	0.184	1.73	101	0.418	Protein A	19
	HOH497	1.00	56	0.291	1.00	-557	-1.137	2.00	-501	-0.972	Protein A	4
	HOH498	2.09	166	0.526	0.00	-52	-0.039	2.09	114	0.464	Protein A	2
	HOH501	1.08	39	0.292	0.00	-103	-0.040	1.08	-64	0.213	Protein A	30
	HOH591	2.49	-94	0.283	0.00	-184	-0.125	2.49	-277	-0.379	Protein A	10
	HOH617	2.93	-65	0.378	1.26	349	0.520	4.19	283	0.850	Both	6
	HOH623	0.97	-107	0.159	0.93	7	0.243	1.90	-99	0.162	Neither	31
	HOH625	2.57	263	0.655	1.24	-81	0.191	3.81	182	0.778	Protein A	28
	HOH626	3.17	111	0.585	0.00	-293	-0.397	3.17	-182	-0.156	Protein A	6
	HOH632	0.92	102	0.308	0.00	-251	-0.287	0.92	-149	-0.081	Protein A	27
	HOH633	1.34	451	0.613	2.36	-165	-0.118	3.70	286	0.802	Protein A	47
	HOH634	1.44	-131	-0.044	1.06	-416	-0.734	2.50	-547	-1.105	Neither	51
	HOH645	2.02	138	0.486	0.00	-90	-0.040	2.02	49	0.375	Protein A	31
	HOH665	1.10	-311	-0.463	0.00	-227	-0.227	1.10	-538	-1.080	Neither	13
	HOH678	1.33	37	0.317	2.35	20	0.394	3.69	57	0.598	Both	0
	HOH687	0.00	-84	-0.040	0.00	-241	-0.261	0.00	-325	-0.482	Neither	38
	HOH699	0.00	-187	-0.132	0.00	-8	-0.039	0.00	-195	-0.149	Neither	24
	HOH700	0.00	-13	-0.039	0.00	-60	-0.039	0.00	-73	-0.040	Neither	15
	HOH702	0.00	-198	-0.156	0.00	-269	-0.334	0.00	-467	-0.871	Neither	42
	HOH730	0.00	-100	-0.040	0.95	-227	-0.259	0.95	-327	-0.502	Neither	20
	HOH748	2.42	26	0.411	2.76	5	0.433	5.18	31	0.678	Both	10
	HOH750	0.00	-97	-0.040	1.00	122	0.332	1.00	25	0.268	Protein B	0
	HOH753	1.15	496	0.523	1.19	-57	0.219	2.34	439	0.733	Protein A	0
	HOH777	0.90	30	0.256	1.14	479	0.509	2.04	509	0.767	Both	1
	HOH778	0.00	-45	-0.039	3.60	155	0.706	3.60	110	0.636	Protein B	19
	HOH779	1.11	-40	0.236	2.22	-115	-0.007	3.32	-155	-0.094	Neither	15
	HOH781	1.13	-325	-0.497	1.20	98	0.361	2.34	-227	-0.259	Protein B	5
	HOH782	0.97	-40	0.213	2.32	77	0.453	3.29	37	0.528	Protein B	0
	HOH784	1.04	-133	-0.046	2.36	88	0.469	3.40	-45	0.461	Protein B	39
	HOH785	2.32	59	0.433	1.35	346	0.554	3.67	406	0.846	Both	33
	HOH788	2.30	-88	0.255	1.35	31	0.312	3.65	-57	0.486	Both	40
	HOH789	3.90	-115	0.000	1.43	132	0.424	5.33	17	0.672	Protein B	38
	HOH791	0.00	-262	-0.317	2.12	43	0.384	2.12	-219	-0.242	Protein B	1
	HOH793	0.87	51	0.266	0.98	-91	0.175	1.86	-39	0.247	Protein A	43
	HOH794	0.00	-196	-0.152	0.00	-118	-0.033	0.00	-314	-0.453	Neither	47
1USU	HOH73	2.20	53	0.408	0.00	80	-0.038	2.20	134	0.502	Protein A	23
	HOH116	1.07	-140	-0.062	0.00	-180	-0.117	1.07	-320	-0.484	Neither	6
	HOH118	2.45	-214	-0.229	1.14	18	0.284	3.59	-196	-0.188	Protein B	0
	HOH119	1.51	210	0.480	4.01	-339	-0.532	5.53	-129	-0.032	Protein A	6
	HOH207	1.09	-270	-0.362	1.10	200	0.353	2.19	-70	0.254	Protein B	10
	HOH209	0.99	10	0.255	2.60	287	0.672	3.59	296	0.792	Both	14
	HOH211	0.96	-250	-0.313	1.33	434	0.600	2.29	184	0.570	Protein B	1
	HOH212	1.11	9	0.274	4.24	86	0.672	5.35	95	0.739	Both	49
	HOH219	0.00	-225	-0.221	1.00	97	0.320	1.00	-127	-0.034	Protein B	39
	HOH220	0.88	-285	-0.396	1.01	131	0.337	1.89	-154	-0.093	Protein B	8
	HOH221	4.17	-10	0.585	1.60	65	0.366	5.77	55	0.720	Both	22
	HOH222	1.06	-18	0.246	1.11	133	0.356	2.17	115	0.475	Protein B	39
	HOH223	0.84	-57	0.177	0.79	39	0.242	1.62	-18	0.264	Neither	40

1V7P	HOH1511	4.15	505	0.926	1.17	28	0.295	5.32	533	1.012	Both	9
	HOH1512	1.12	-151	-0.086	0.84	67	0.272	1.96	-84	0.185	Protein B	2
	HOH1534	3.73	177	0.759	1.38	272	0.507	5.11	450	0.973	Both	4
	HOH1579	1.00	-65	0.198	0.97	93	0.311	1.96	28	0.340	Protein B	16
	HOH1631	2.67	143	0.575	2.26	178	0.561	4.94	322	0.917	Both	1
	HOH1635	1.00	-73	0.192	0.90	-23	0.215	1.90	-96	0.168	Neither	1
	HOH1639	1.23	125	0.386	1.00	3	0.252	2.23	128	0.499	Both	8
	HOH1023	2.03	37	0.362	0.00	-252	-0.290	2.03	-215	-0.230	Protein A	13
	HOH1032	1.07	-346	-0.551	1.05	-241	-0.293	2.11	-588	-1.228	Neither	1
	HOH1037	2.69	187	0.630	2.57	-205	-0.209	5.26	-19	0.637	Protein A	31
	HOH1069	0.94	-114	-0.006	0.00	-205	-0.174	0.94	-320	-0.483	Neither	8
	HOH1073	2.74	-260	-0.338	2.62	241	0.649	5.36	-19	0.641	Protein B	3
	HOH1090	2.36	325	0.668	1.18	-105	0.173	3.54	220	0.766	Protein A	8
	HOH1104	2.13	70	0.417	0.00	21	-0.039	2.13	91	0.441	Protein A	22
	HOH1110	2.50	101	0.500	1.26	287	0.477	3.76	387	0.848	Both	1
	HOH1124	2.37	56	0.435	0.00	-248	-0.280	2.37	-192	-0.178	Protein A	6
	HOH1143	0.81	52	0.254	0.81	-91	0.145	1.62	-39	0.236	Protein A	0
	HOH1146	1.20	165	0.391	1.14	-182	-0.156	2.33	-17	0.348	Protein A	1
	HOH1508	0.00	-62	-0.039	2.27	28	0.389	2.27	-34	0.316	Protein B	0
	HOH1516	1.01	-24	0.233	2.23	-65	0.270	3.24	-89	0.392	Protein B	1
	HOH1526	1.10	5	0.271	1.12	458	0.491	2.22	463	0.741	Both	16
	HOH1529	0.98	57	0.288	1.04	-178	-0.146	2.02	-121	-0.022	Protein A	21
	HOH1539	2.59	-43	0.358	1.35	63	0.346	3.94	20	0.594	Both	29
	HOH1540	0.00	-43	-0.039	2.05	-103	0.176	2.05	-146	-0.076	Neither	21
	HOH1546	0.78	1	0.212	0.00	-88	-0.040	0.78	-87	0.142	Neither	51
	HOH1559	1.03	16	0.266	1.18	82	0.342	2.21	97	0.459	Both	11
	HOH1564	0.00	0	-0.039	1.02	58	0.296	1.02	58	0.296	Protein B	7
	HOH1568	1.21	-2	0.271	3.59	46	0.574	4.80	44	0.670	Both	15
	HOH1589	0.92	16	0.247	1.09	35	0.292	2.01	51	0.375	Protein B	34
	HOH1595	3.78	180	0.770	1.48	-32	0.241	5.26	148	0.826	Protein A	7
	HOH1616	2.16	328	0.654	1.21	207	0.395	3.37	535	0.866	Both	8
	HOH1619	0.70	35	0.224	0.00	-56	-0.039	0.70	-20	0.182	Neither	1
	HOH1629	2.02	21	0.339	1.25	-47	0.226	3.26	-25	0.462	Protein A	27
	HOH1633	3.71	125	0.671	1.17	127	0.371	4.88	252	0.899	Both	28
	HOH1652	1.10	-91	0.195	1.19	103	0.361	2.28	12	0.374	Protein B	10
	HOH1661	0.84	8	0.227	1.01	-45	0.217	1.85	-37	0.250	Neither	42
	HOH1674	0.00	-59	-0.039	0.00	5	-0.039	0.00	-54	-0.039	Neither	31
1VG0	HOH13	1.39	27	0.309	2.67	11	0.428	4.06	38	0.620	Both	0
	HOH16	2.46	155	0.563	1.21	-75	0.199	3.67	79	0.614	Protein A	16
	HOH22	1.03	-98	0.177	1.00	34	0.275	2.03	-64	0.227	Protein B	25
	HOH29	2.24	143	0.519	0.00	-44	-0.039	2.24	99	0.465	Protein A	0
	HOH36	1.38	133	0.418	2.24	-444	-0.812	3.62	-311	-0.461	Protein A	39
	HOH44	2.36	-12	0.358	1.05	-16	0.247	3.41	-28	0.479	Protein A	34
	HOH53	2.29	-208	-0.214	1.04	-110	0.004	3.33	-318	-0.478	Neither	22
	HOH72	1.16	-116	-0.009	1.12	-103	0.182	2.28	-219	-0.240	Neither	49
	HOH83	2.53	148	0.563	1.03	25	0.275	3.56	173	0.730	Both	28
	HOH88	1.99	-61	0.220	0.00	-262	-0.317	1.99	-324	-0.493	Neither	4
	HOH134	2.35	-41	0.323	1.17	-50	0.226	3.53	-90	0.432	Protein A	9
	HOH145	1.02	155	0.342	0.96	-106	0.158	1.98	50	0.368	Protein A	5
	HOH163	1.09	71	0.317	0.00	-248	-0.280	1.09	-177	-0.144	Protein A	9
	HOH165	1.08	107	0.340	1.00	16	0.262	2.08	123	0.473	Both	9
	HOH171	1.12	-102	0.183	0.86	-27	0.205	1.99	-129	-0.039	Neither	2
	HOH191	0.00	-29	-0.039	0.76	8	0.214	0.76	-21	0.191	Neither	8
	HOH217	1.06	94	0.328	0.86	-6	0.222	1.92	89	0.415	Protein A	9
	HOH226	2.12	-101	0.197	1.03	-288	-0.404	3.15	-389	-0.663	Neither	2
	HOH228	5.23	372	0.951	0.00	-322	-0.474	5.23	51	0.697	Protein A	20
	HOH232	3.57	62	0.586	0.00	-44	-0.039	3.57	18	0.546	Protein A	25
	HOH248	1.11	37	0.296	1.93	-21	0.274	3.04	16	0.477	Both	0
	HOH252	0.00	-71	-0.040	0.00	-158	-0.076	0.00	-229	-0.231	Neither	0
	HOH321	1.10	29	0.288	0.93	-242	-0.294	2.03	-213	-0.228	Protein A	30

	HOH349	1.18	59	0.323	2.29	7	0.368	3.46	65	0.575	Both	7
	HOH351	1.07	-122	-0.022	1.14	-234	-0.275	2.21	-356	-0.575	Neither	0
	HOH352	0.00	-347	-0.541	1.00	103	0.324	1.00	-244	-0.298	Protein B	42
	HOH355	2.06	-105	0.174	1.17	31	0.299	3.23	-74	0.406	Protein B	35
	HOH384	2.15	372	0.684	1.16	131	0.370	3.30	504	0.849	Both	16
	HOH400	1.04	-364	-0.595	1.22	429	0.540	2.25	65	0.430	Protein B	10
	HOH401	1.00	-51	0.209	2.72	80	0.505	3.72	29	0.578	Protein B	6
	HOH402	0.00	-503	-0.974	0.99	172	0.335	0.99	-331	-0.513	Protein B	22
	HOH426	0.90	-252	-0.317	1.03	101	0.327	1.93	-151	-0.087	Protein B	28
1WA5	HOH41	3.65	-135	-0.048	1.21	98	0.361	4.86	-37	0.601	Protein B	8
	HOH54	3.71	-190	-0.174	1.13	-30	0.245	4.84	-220	-0.242	Neither	6
	HOH74	4.14	116	0.699	1.60	-77	0.183	5.74	39	0.706	Protein A	10
	HOH152	0.87	-21	0.211	1.05	-181	-0.153	1.91	-202	-0.201	Neither	4
	HOH153	0.00	-280	-0.365	0.96	-305	-0.447	0.96	-586	-1.221	Neither	32
	HOH155	2.29	-212	-0.224	1.21	186	0.396	3.50	-26	0.493	Protein B	15
	HOH161	3.55	-474	-0.895	1.45	525	0.688	5.00	51	0.687	Protein B	18
	HOH166	1.11	-75	0.208	2.32	37	0.408	3.43	-38	0.472	Protein B	29
	HOH167	0.84	-17	0.209	1.08	-131	-0.042	1.92	-148	-0.080	Neither	14
	HOH172	2.26	3	0.360	1.17	208	0.381	3.43	211	0.751	Both	7
	HOH174	2.11	-8	0.318	1.32	419	0.585	3.42	411	0.823	Both	25
	HOH187	0.00	-174	-0.104	0.00	-261	-0.314	0.00	-435	-0.780	Neither	0
	HOH215	1.98	350	0.667	0.96	-446	-0.816	2.95	-95	0.346	Protein A	10
	HOH232	1.20	-89	0.186	1.02	210	0.337	2.21	120	0.487	Protein B	7
	HOH234	2.40	-338	-0.529	1.13	-102	0.182	3.53	-440	-0.800	Neither	21
	HOH236	2.69	-274	-0.372	1.33	61	0.342	4.01	-214	-0.227	Protein B	0
	HOH237	1.15	74	0.330	0.94	-18	0.226	2.09	57	0.395	Protein A	15
	HOH238	1.03	37	0.281	1.91	-21	0.274	2.93	16	0.465	Both	0
	HOH240	3.86	490	0.896	1.37	-284	-0.396	5.23	206	0.929	Protein A	35
	HOH241	0.00	-229	-0.232	0.00	-247	-0.278	0.00	-476	-0.897	Neither	4
	HOH243	2.37	104	0.488	1.00	-5	0.246	3.37	99	0.594	Protein A	1
	HOH246	0.87	-142	-0.067	0.86	-358	-0.582	1.73	-500	-0.970	Neither	18
	HOH256	3.70	-22	0.527	1.20	-44	0.231	4.90	-67	0.576	Protein A	18
	HOH273	3.50	-9	0.510	0.00	-75	-0.040	3.50	-84	0.434	Protein A	10
	HOH276	1.99	28	0.343	0.00	-217	-0.202	1.99	-189	-0.173	Protein A	4
	HOH307	0.96	155	0.330	0.00	-401	-0.687	0.96	-246	-0.304	Protein A	4
	HOH310	2.21	87	0.448	1.08	-244	-0.299	3.29	-157	-0.098	Protein A	6
	HOH451	3.86	-186	-0.163	1.24	11	0.285	5.10	-175	-0.137	Protein B	0
1WWW	HOH1	2.17	82	0.436	1.05	-226	-0.256	3.21	-144	-0.068	Protein A	8
	HOH19	1.27	49	0.325	0.97	22	0.261	2.24	71	0.433	Both	27
	HOH26	3.54	-480	-0.910	0.00	-20	-0.039	3.54	-500	-0.968	Neither	15
	HOH29	1.22	-72	0.202	2.20	-51	0.281	3.41	-123	-0.022	Protein B	2
	HOH51	2.24	214	0.583	0.99	-117	-0.011	3.22	98	0.576	Protein A	7
	HOH66	1.25	135	0.395	1.24	190	0.404	2.48	324	0.680	Both	2
	HOH72	0.90	-301	-0.438	0.00	-530	-1.053	0.90	-831	-2.029	Neither	7
	HOH76	1.05	-79	0.195	1.04	356	0.427	2.09	277	0.611	Protein B	0
	HOH78	1.22	-45	0.229	2.17	-579	-1.203	3.39	-625	-1.342	Neither	17
	HOH83	3.72	126	0.673	1.10	-48	0.230	4.81	78	0.700	Protein A	30
	HOH86	1.19	10	0.281	1.00	223	0.343	2.19	233	0.588	Both	36
	HOH96	3.95	-241	-0.292	1.26	164	0.408	5.21	-77	0.580	Protein B	15
	HOH129	0.99	-122	-0.023	1.04	-34	0.229	2.02	-157	-0.101	Neither	9
	HOH133	1.21	-457	-0.847	1.23	-3	0.271	2.44	-460	-0.855	Protein B	2
	HOH143	2.03	-3	0.310	1.26	-51	0.221	3.29	-54	0.436	Protein A	8
	HOH145	0.00	-51	-0.039	1.03	-259	-0.334	1.03	-310	-0.460	Neither	7
	HOH146	0.00	-72	-0.040	0.00	-311	-0.445	0.00	-383	-0.638	Neither	5
	HOH148	1.05	-284	-0.394	2.29	248	0.611	3.34	-36	0.462	Protein B	0
	HOH151	1.33	-108	0.149	2.80	-86	0.337	4.13	-194	-0.182	Protein B	10
	HOH166	1.01	-56	0.207	1.13	-103	0.181	2.14	-160	-0.106	Neither	2
	HOH194	1.11	88	0.332	2.15	267	0.609	3.26	355	0.780	Both	11
1WXC	HOH8	2.86	62	0.502	2.45	-89	0.282	5.30	-27	0.632	Both	3
	HOH20	3.62	262	0.784	1.13	-18	0.255	4.75	244	0.890	Both	0

	HOH40	0.93	-192	-0.177	0.00	-200	-0.161	0.93	-391	-0.669	Neither	43
	HOH52	1.16	-377	-0.630	1.14	214	0.374	2.30	-162	-0.112	Protein B	8
	HOH53	3.90	134	0.708	1.10	-158	-0.102	5.00	-24	0.621	Protein A	0
	HOH63	2.11	89	0.436	0.00	-89	-0.040	2.11	0	0.329	Protein A	25
	HOH99	1.34	-220	-0.244	2.45	-172	-0.134	3.79	-393	-0.672	Neither	0
	HOH100	2.72	-3	0.420	2.71	-56	0.359	5.43	-59	0.606	Both	4
	HOH105	2.34	188	0.580	1.23	167	0.402	3.57	355	0.814	Both	29
	HOH120	0.96	-99	0.163	0.81	-121	-0.022	1.77	-221	-0.245	Neither	45
	HOH146	1.19	-151	-0.087	1.11	30	0.290	2.30	-121	-0.021	Protein B	0
	HOH242	1.19	217	0.397	2.25	2	0.356	3.44	219	0.753	Both	9
	HOH252	2.28	-71	0.273	1.08	42	0.294	3.36	-29	0.471	Both	13
	HOH297	0.00	-528	-1.047	1.82	-10	0.285	1.82	-538	-1.079	Protein B	1
	HOH300	0.78	-22	0.194	1.04	32	0.280	1.82	10	0.311	Protein B	10
	HOH309	0.93	18	0.251	1.28	437	0.575	2.20	456	0.736	Both	0
	HOH323	4.09	143	0.739	1.41	106	0.397	5.50	249	0.935	Both	33
	HOH334	0.96	-367	-0.605	2.31	-93	0.249	3.26	-461	-0.857	Neither	52
	HOH337	1.08	-223	-0.250	1.26	161	0.409	2.34	-62	0.295	Protein B	27
	HOH340	1.06	-35	0.233	2.27	233	0.599	3.33	198	0.730	Protein B	40
	HOH342	1.17	-48	0.228	1.37	-463	-0.864	2.54	-511	-0.999	Neither	8
	HOH347	3.89	-521	-1.028	1.65	-80	0.181	5.54	-600	-1.267	Neither	6
	HOH359	1.04	-128	-0.036	2.35	296	0.648	3.39	168	0.699	Protein B	17
	HOH371	0.70	-97	0.121	0.00	-227	-0.226	0.70	-324	-0.493	Neither	0
	HOH386	0.00	-116	-0.032	0.00	-114	-0.031	0.00	-229	-0.232	Neither	0
1XG2	HOH345	3.43	-396	-0.680	0.00	-254	-0.297	3.43	-650	-1.421	Neither	1
	HOH347	1.20	-199	-0.196	2.26	197	0.575	3.46	-3	0.511	Protein B	0
	HOH350	0.95	-48	0.204	0.00	-34	-0.039	0.95	-81	0.177	Neither	0
	HOH362	2.27	-146	-0.076	0.00	18	-0.039	2.27	-129	-0.037	Neither	2
	HOH396	3.75	182	0.769	1.33	6	0.283	5.08	189	0.894	Both	3
	HOH401	1.24	5	0.279	1.15	-205	-0.208	2.39	-200	-0.197	Protein A	4
	HOH402	1.30	490	0.614	1.18	286	0.443	2.49	777	0.853	Both	3
	HOH420	1.19	63	0.328	1.15	17	0.284	2.33	80	0.457	Both	11
	HOH425	1.11	-378	-0.632	1.01	244	0.359	2.12	-133	-0.048	Protein B	11
	HOH437	2.30	-48	0.304	1.23	238	0.428	3.53	189	0.747	Both	1
	HOH511	1.90	62	0.381	0.00	-31	-0.039	1.90	31	0.342	Protein A	4
	HOH525	2.20	-39	0.297	1.11	42	0.300	3.31	3	0.497	Both	11
	HOH536	2.26	4	0.361	0.97	15	0.256	3.23	19	0.503	Both	20
	HOH543	0.00	-54	-0.039	0.00	-70	-0.040	0.00	-124	-0.037	Neither	14
	HOH559	1.10	-23	0.249	0.96	-130	-0.039	2.05	-153	-0.091	Neither	1
	HOH569	0.92	30	0.259	0.89	-58	0.186	1.82	-28	0.261	Protein A	25
	HOH592	0.80	-65	0.164	0.00	34	-0.038	0.80	-30	0.192	Neither	52
	HOH153	0.95	-6	0.236	1.17	5	0.275	2.11	-1	0.327	Protein B	12
	HOH154	2.12	-140	-0.062	1.23	-446	-0.818	3.35	-586	-1.223	Neither	23
	HOH155	0.00	-132	-0.044	0.98	77	0.302	0.98	-55	0.202	Protein B	31
	HOH156	0.00	-202	-0.167	1.01	335	0.410	1.01	133	0.337	Protein B	21
	HOH157	1.08	-46	0.229	1.21	-55	0.220	2.30	-100	0.239	Neither	5
	HOH162	1.07	-64	0.212	1.10	-196	-0.186	2.17	-259	-0.336	Neither	4
	HOH175	0.00	-261	-0.315	1.98	93	0.423	1.98	-169	-0.126	Protein B	6
	HOH181	0.00	-260	-0.311	0.97	126	0.328	0.97	-134	-0.048	Protein B	7
	HOH182	1.13	-58	0.221	1.97	356	0.671	3.10	298	0.736	Protein B	0
	HOH185	1.00	-240	-0.290	2.28	-150	-0.085	3.27	-390	-0.665	Neither	11
	HOH188	0.00	-3	-0.039	2.02	-155	-0.095	2.02	-157	-0.102	Neither	1
	HOH202	1.13	273	0.411	2.02	382	0.690	3.15	656	0.883	Both	4
	HOH204	1.25	53	0.326	2.65	749	0.859	3.89	802	0.967	Both	22
	HOH208	2.15	66	0.415	1.33	146	0.417	3.47	212	0.757	Both	37
	HOH220	3.88	23	0.593	1.51	541	0.711	5.39	563	1.024	Both	30
	HOH223	0.00	-95	-0.040	2.02	186	0.535	2.02	92	0.426	Protein B	16
	HOH231	0.00	-68	-0.039	0.73	30	0.225	0.73	-38	0.174	Neither	5
	HOH240	1.16	-111	0.001	2.63	513	0.789	3.79	401	0.856	Protein B	39
	HOH243	0.91	-8	0.227	0.93	-45	0.203	1.84	-53	0.227	Neither	22
	HOH245	0.00	-182	-0.121	1.03	121	0.337	1.03	-61	0.206	Protein B	20

	HOH246	0.00	4	-0.039	2.04	300	0.627	2.04	303	0.630	Protein B	37
	HOH274	0.90	-185	-0.163	1.20	416	0.527	2.11	231	0.578	Protein B	6
	HOH283	2.29	64	0.434	1.43	314	0.552	3.72	377	0.839	Both	43
	HOH284	0.91	5	0.238	1.12	173	0.364	2.03	178	0.529	Protein B	42
1XKP	HOH291	1.33	181	0.431	2.51	-101	0.278	3.84	80	0.640	Both	22
	HOH301	2.17	32	0.379	1.18	-141	-0.065	3.35	-109	0.385	Protein A	9
	HOH307	1.34	-427	-0.764	2.23	-230	-0.267	3.57	-657	-1.445	Neither	3
	HOH312	1.32	708	0.692	1.03	-80	0.192	2.35	628	0.813	Protein A	0
	HOH322	1.50	67	0.361	4.23	-2	0.597	5.74	65	0.727	Both	0
	HOH323	1.17	186	0.382	1.12	-101	0.184	2.29	86	0.457	Protein A	14
	HOH328	1.07	-124	-0.027	0.00	-83	-0.040	1.07	-207	-0.212	Neither	14
	HOH329	4.19	20	0.613	1.31	-86	0.179	5.50	-65	0.603	Protein A	17
	HOH337	0.82	-301	-0.436	0.00	-246	-0.275	0.82	-547	-1.105	Neither	33
	HOH344	2.27	45	0.409	2.79	149	0.596	5.06	193	0.900	Both	6
	HOH357	2.60	74	0.485	1.11	-39	0.237	3.71	35	0.582	Protein A	27
	HOH360	1.07	-214	-0.229	0.00	-197	-0.155	1.07	-412	-0.723	Neither	53
	HOH371	1.01	-327	-0.501	0.00	-250	-0.284	1.01	-576	-1.193	Neither	0
	HOH381	2.19	169	0.541	0.00	-199	-0.159	2.19	-30	0.306	Protein A	28
	HOH129	1.12	-78	0.204	1.16	-358	-0.582	2.29	-436	-0.790	Neither	0
	HOH136	0.00	-22	-0.039	2.41	302	0.659	2.41	280	0.645	Protein B	20
	HOH144	2.52	144	0.556	1.38	-196	-0.188	3.90	-52	0.526	Protein A	0
	HOH147	1.21	230	0.413	4.11	191	0.821	5.32	421	0.973	Both	31
	HOH150	1.29	304	0.503	2.61	-88	0.308	3.90	216	0.815	Both	17
	HOH153	1.10	36	0.294	2.66	469	0.774	3.76	505	0.892	Both	0
	HOH158	3.98	309	0.839	1.52	-57	0.208	5.49	252	0.935	Protein A	15
	HOH160	0.93	164	0.323	0.00	-146	-0.060	0.93	18	0.251	Protein A	5
	HOH169	0.00	-50	-0.039	0.00	-160	-0.080	0.00	-210	-0.184	Neither	3
	HOH170	0.00	-300	-0.417	1.03	-51	0.215	1.03	-351	-0.564	Neither	15
	HOH176	0.88	-99	0.149	1.15	-70	0.209	2.02	-170	-0.129	Neither	7
	HOH177	1.03	29	0.276	1.07	132	0.349	2.10	161	0.521	Both	33
1XX9	HOH2	2.68	97	0.516	1.35	57	0.339	4.03	153	0.752	Both	1
	HOH87	1.03	-17	0.241	0.86	20	0.241	1.89	3	0.305	Neither	12
	HOH91	0.90	89	0.297	0.00	-5	-0.039	0.90	84	0.294	Protein A	30
	HOH136	1.34	145	0.420	2.37	-308	-0.454	3.71	-163	-0.111	Protein A	0
	HOH158	0.96	47	0.278	0.89	-31	0.206	1.85	16	0.320	Protein A	3
	HOH171	0.89	-54	0.189	0.99	13	0.257	1.88	-42	0.244	Protein B	14
	HOH173	3.65	-333	-0.518	1.48	174	0.459	5.13	-160	-0.102	Protein B	0
	HOH181	1.12	100	0.343	2.61	-245	-0.302	3.72	-145	-0.071	Protein A	3
	HOH182	0.92	32	0.260	1.18	-20	0.254	2.10	12	0.342	Both	2
	HOH197	0.00	17	-0.039	5.13	-403	-0.699	5.13	-386	-0.655	Neither	42
	HOH198	1.30	239	0.453	2.67	-293	-0.417	3.97	-54	0.529	Protein A	17
	HOH201	0.86	143	0.311	0.97	-150	-0.084	1.83	-7	0.289	Protein A	18
	HOH209	1.05	-233	-0.274	1.99	37	0.354	3.04	-196	-0.187	Protein B	2
	HOH211	0.96	-159	-0.105	1.00	86	0.312	1.96	-73	0.201	Protein B	20
	HOH212	3.92	368	0.856	1.60	47	0.344	5.52	415	0.980	Both	50
1YAR	HOH470	2.09	118	0.469	0.00	-151	-0.065	2.09	-32	0.283	Protein A	2
	HOH561	2.57	127	0.541	1.19	-210	-0.220	3.77	-83	0.480	Protein A	14
	HOH587	2.40	480	0.758	1.10	42	0.297	3.50	522	0.873	Both	21
	HOH601	2.01	16	0.331	0.00	-236	-0.250	2.01	-220	-0.244	Protein A	19
	HOH611	3.60	97	0.620	1.18	-160	-0.107	4.78	-64	0.572	Protein A	10
	HOH623	1.69	9	0.303	0.00	-141	-0.052	1.69	-131	-0.045	Protein A	22
	HOH633	2.25	-338	-0.529	0.00	-197	-0.154	2.25	-534	-1.068	Neither	11
	HOH748	3.62	-524	-1.038	1.27	20	0.296	4.90	-504	-0.980	Protein B	28
	HOH2152	2.52	-741	-1.719	1.30	-28	0.245	3.83	-769	-1.814	Neither	34
	HOH2169	0.00	-37	-0.039	1.99	291	0.618	1.99	254	0.586	Protein B	16
	HOH2187	0.91	55	0.275	1.10	-6	0.262	2.01	49	0.372	Both	16
	HOH2209	1.09	1	0.265	2.43	422	0.732	3.52	423	0.838	Both	22
	HOH2214	0.98	-129	-0.038	0.81	-138	-0.058	1.79	-267	-0.354	Neither	2
	HOH2227	0.92	-413	-0.725	1.19	93	0.353	2.11	-320	-0.485	Protein B	10
	HOH2237	0.93	-26	0.218	2.41	294	0.654	3.35	268	0.752	Protein B	5

	HOH2247	0.00	-198	-0.156	0.97	56	0.286	0.97	-142	-0.066	Protein B	1
	HOH2270	0.00	-188	-0.133	0.00	11	-0.039	0.00	-177	-0.110	Neither	15
	HOH2293	0.91	-372	-0.617	1.04	-26	0.237	1.95	-398	-0.686	Neither	0
	HOH2305	0.89	-261	-0.339	0.88	21	0.244	1.76	-240	-0.291	Neither	0
1YCS	HOH137	1.89	41	0.354	0.00	-68	-0.039	1.89	-28	0.263	Protein A	38
	HOH139	2.37	197	0.592	1.07	-8	0.256	3.44	190	0.735	Both	20
	HOH146	3.82	117	0.675	1.09	6	0.269	4.91	123	0.758	Both	18
	HOH222	1.07	79	0.320	1.13	-252	-0.319	2.20	-173	-0.137	Protein A	27
	HOH244	0.84	-79	0.159	0.96	19	0.257	1.80	-61	0.215	Protein B	39
	HOH264	0.00	-171	-0.099	4.88	23	0.657	4.88	-147	-0.074	Protein B	0
	HOH270	2.30	21	0.388	1.44	117	0.411	3.74	138	0.697	Both	12
1YRO	HOH1025	1.30	145	0.412	2.37	196	0.592	3.67	341	0.819	Both	14
	HOH1099	0.91	-123	-0.025	0.00	-266	-0.327	0.91	-389	-0.662	Neither	10
	HOH1162	1.04	-293	-0.417	0.00	-37	-0.039	1.04	-330	-0.509	Neither	25
	HOH1189	1.20	42	0.311	1.01	-69	0.196	2.21	-27	0.314	Protein A	8
	HOH1221	1.11	148	0.359	0.95	166	0.327	2.05	315	0.639	Both	26
	HOH1342	1.09	48	0.301	1.03	-244	-0.299	2.12	-196	-0.188	Protein A	5
	HOH1355	2.39	26	0.406	0.92	-80	0.172	3.31	-55	0.438	Protein A	17
	HOH1395	2.34	50	0.426	1.11	30	0.291	3.46	80	0.588	Both	35
	HOH1453	1.19	68	0.332	1.14	334	0.449	2.33	401	0.712	Both	5
	HOH1479	1.03	-820	-1.988	0.88	80	0.287	1.90	-740	-1.714	Protein B	18
	HOH1660	2.49	83	0.480	1.16	32	0.298	3.65	115	0.648	Both	0
	HOH920	1.35	22	0.301	4.11	149	0.751	5.46	171	0.881	Both	26
	HOH937	1.17	-58	0.219	3.66	29	0.570	4.83	-28	0.608	Protein B	0
	HOH967	0.85	-326	-0.499	1.16	98	0.351	2.01	-228	-0.261	Protein B	29
	HOH974	1.15	150	0.375	1.18	-70	0.206	2.33	79	0.456	Protein A	10
	HOH1069	0.00	-67	-0.039	3.32	-1	0.494	3.32	-68	0.425	Protein B	19
	HOH1078	0.00	-241	-0.263	1.09	-44	0.231	1.09	-285	-0.397	Neither	21
	HOH1080	1.12	-117	-0.011	1.29	501	0.609	2.41	385	0.709	Protein B	19
	HOH1092	0.89	19	0.245	1.03	27	0.275	1.92	45	0.361	Protein B	15
	HOH1108	0.79	-37	0.184	1.15	65	0.323	1.94	28	0.339	Protein B	13
	HOH1157	0.00	-235	-0.246	0.85	-351	-0.562	0.85	-585	-1.220	Neither	12
	HOH1195	1.30	105	0.382	2.47	90	0.484	3.76	195	0.787	Both	8
	HOH1225	1.13	-189	-0.172	1.26	-58	0.213	2.39	-248	-0.308	Neither	9
	HOH1257	0.97	-89	0.174	1.10	-182	-0.156	2.07	-271	-0.364	Neither	24
	HOH1261	1.09	66	0.314	1.13	172	0.367	2.22	237	0.595	Both	19
	HOH1287	0.95	-122	-0.022	1.20	473	0.549	2.15	352	0.670	Protein B	10
	HOH1325	0.00	-196	-0.152	0.98	-194	-0.183	0.98	-390	-0.665	Neither	22
	HOH1428	1.17	0	0.271	3.60	26	0.557	4.77	26	0.653	Both	8
	HOH1436	0.00	-188	-0.134	1.81	-162	-0.111	1.81	-350	-0.560	Neither	20
	HOH1452	0.00	-349	-0.547	2.31	446	0.736	2.31	97	0.471	Protein B	4
	HOH1470	0.00	-253	-0.293	0.88	133	0.313	0.88	-121	-0.020	Protein B	3
	HOH1471	1.01	-68	0.197	1.11	153	0.362	2.12	85	0.433	Protein B	31
	HOH1550	0.92	-91	0.164	1.14	81	0.335	2.07	-10	0.308	Protein B	10
	HOH1614	0.99	-92	0.174	1.08	206	0.349	2.07	113	0.460	Protein B	14
	HOH1002	1.04	-48	0.219	0.00	-241	-0.262	1.04	-289	-0.407	Neither	36
	HOH1021	0.98	71	0.298	0.00	-52	-0.039	0.98	19	0.261	Protein A	8
	HOH1237	1.90	472	0.739	0.00	-149	-0.063	1.90	323	0.634	Protein A	1
	HOH1288	2.05	-278	-0.381	0.00	71	-0.038	2.05	-207	-0.214	Neither	30
	HOH1319	2.27	-80	0.260	1.13	53	0.311	3.40	-27	0.480	Both	30
	HOH1338	3.30	154	0.666	0.00	5	-0.039	3.30	159	0.673	Protein A	33
	HOH1394	1.13	251	0.396	1.12	-111	0.001	2.24	140	0.516	Protein A	35
	HOH1407	2.03	-47	0.250	0.00	-532	-1.059	2.03	-578	-1.200	Protein A	0
	HOH1424	1.71	14	0.310	0.00	-118	-0.033	1.71	-104	0.149	Protein A	0
	HOH1450	1.09	12	0.274	0.98	186	0.329	2.07	198	0.550	Both	0
	HOH1667	2.32	375	0.696	1.21	92	0.356	3.52	467	0.856	Both	3
	HOH1709	1.21	-4	0.270	1.05	148	0.348	2.26	144	0.523	Both	13
1YU6	HOH161	2.32	-35	0.325	0.00	-214	-0.194	2.32	-249	-0.311	Protein A	12
	HOH162	1.43	40	0.326	3.47	-110	0.008	4.91	-71	0.572	Protein A	1
	HOH168	0.89	-33	0.205	0.00	-203	-0.169	0.89	-236	-0.280	Neither	36

	HOH186	1.07	-37	0.233	0.93	-40	0.207	2.00	-77	0.200	Neither	20
	HOH189	1.08	70	0.315	0.00	-108	-0.040	1.08	-39	0.234	Protein A	15
	HOH214	3.61	110	0.638	1.14	119	0.360	4.76	229	0.891	Both	13
	HOH224	0.00	-230	-0.234	0.00	-242	-0.264	0.00	-471	-0.884	Neither	16
	HOH253	1.10	61	0.311	0.89	-120	-0.019	1.99	-60	0.222	Protein A	21
	HOH256	4.84	-439	-0.797	0.00	-227	-0.226	4.84	-665	-1.471	Neither	0
	HOH295	1.23	-96	0.174	2.62	367	0.719	3.86	271	0.817	Protein B	12
	HOH298	1.32	57	0.337	2.29	14	0.378	3.61	71	0.599	Both	17
	HOH302	0.89	19	0.245	0.99	-152	-0.087	1.88	-133	-0.048	Neither	16
	HOH309	2.12	-109	0.186	1.34	-13	0.262	3.46	-122	-0.018	Protein B	21
	HOH314	0.87	-93	0.153	1.18	165	0.385	2.05	72	0.407	Protein B	14
	HOH315	1.33	-9	0.267	3.69	-9	0.538	5.02	-18	0.627	Both	2
	HOH319	0.87	-1	0.226	0.93	-77	0.178	1.80	-78	0.191	Neither	44
	HOH320	1.19	-5	0.268	2.23	29	0.385	3.42	25	0.532	Both	18
1Z5Y	HOH2010	3.58	-61	0.471	0.00	-11	-0.039	3.58	-72	0.459	Protein A	18
	HOH2013	1.15	-184	-0.160	0.90	-33	0.206	2.04	-217	-0.236	Neither	20
	HOH2039	1.22	-154	-0.094	1.09	79	0.322	2.30	-76	0.271	Protein B	6
	HOH2060	2.42	-136	-0.053	1.18	-65	0.211	3.60	-201	-0.198	Neither	5
	HOH2097	0.76	28	0.228	0.00	13	-0.039	0.76	41	0.237	Neither	5
	HOH2099	3.44	103	0.607	1.10	-242	-0.295	4.53	-140	-0.057	Protein A	38
	HOH2119	2.29	40	0.407	0.97	14	0.256	3.26	54	0.540	Both	43
	HOH2120	1.02	-6	0.248	0.99	14	0.258	2.00	9	0.320	Protein B	5
	HOH2147	1.98	-157	-0.101	0.00	26	-0.038	1.98	-131	-0.044	Neither	25
	HOH2187	0.97	-53	0.202	0.97	-48	0.207	1.94	-102	0.159	Neither	30
	HOH1007	0.00	-310	-0.442	1.02	62	0.299	1.02	-247	-0.306	Protein B	0
	HOH1012	0.00	-191	-0.140	1.96	80	0.407	1.96	-110	0.000	Protein B	56
	HOH1017	2.32	30	0.400	1.31	47	0.326	3.63	77	0.607	Both	13
	HOH1050	0.00	-52	-0.039	3.56	240	0.772	3.56	189	0.750	Protein B	16
	HOH1051	1.13	-111	0.002	3.64	-29	0.513	4.77	-140	-0.057	Protein B	33
	HOH1057	0.91	-21	0.218	1.22	98	0.362	2.13	77	0.424	Protein B	31
	HOH1060	0.00	-151	-0.066	2.16	189	0.555	2.16	38	0.383	Protein B	24
1ZC3	HOH4	0.99	-41	0.216	0.87	-120	-0.019	1.85	-161	-0.109	Neither	26
	HOH5	0.98	97	0.317	0.00	-105	-0.040	0.98	-8	0.241	Protein A	1
	HOH22	1.38	-492	-0.946	1.17	-91	0.187	2.55	-583	-1.214	Neither	0
	HOH26	1.23	144	0.394	1.19	-384	-0.648	2.42	-239	-0.288	Protein A	32
	HOH38	1.31	184	0.425	1.06	84	0.321	2.37	268	0.632	Both	1
	HOH39	1.04	193	0.343	0.96	-57	0.197	2.00	136	0.479	Protein A	1
	HOH66	0.89	-14	0.220	1.09	66	0.314	1.98	52	0.372	Protein B	1
	HOH73	3.70	38	0.583	1.38	-61	0.205	5.08	-22	0.626	Protein A	0
	HOH84	0.82	-202	-0.202	0.00	-21	-0.039	0.82	-223	-0.251	Neither	16
	HOH87	3.43	510	0.864	0.00	-41	-0.039	3.43	469	0.848	Protein A	5
	HOH90	0.00	-73	-0.040	0.74	-107	0.118	0.74	-180	-0.152	Neither	12
	HOH110	0.99	4	0.251	0.89	62	0.277	1.88	67	0.386	Both	5
	HOH340	0.00	-214	-0.194	2.02	263	0.595	2.02	49	0.374	Protein B	18
	HOH345	1.11	-229	-0.262	2.37	-189	-0.171	3.48	-417	-0.737	Neither	9
	HOH349	2.16	48	0.395	1.34	-349	-0.557	3.49	-301	-0.436	Protein A	40
	HOH355	0.98	15	0.258	1.03	-544	-1.098	2.01	-529	-1.054	Protein A	20
	HOH362	1.16	-373	-0.619	3.62	-127	-0.030	4.79	-500	-0.968	Neither	8
	HOH364	0.00	-173	-0.103	2.16	475	0.746	2.16	302	0.635	Protein B	0
	HOH377	2.23	-27	0.317	1.33	-1	0.276	3.56	-28	0.500	Both	51
	HOH382	1.03	-144	-0.071	1.20	197	0.392	2.23	53	0.413	Protein B	13
	HOH385	0.00	-28	-0.039	1.87	-65	0.212	1.87	-93	0.171	Neither	40
	HOH393	0.00	-41	-0.039	0.78	-2	0.209	0.78	-43	0.177	Neither	19
	HOH406	0.00	-82	-0.040	0.00	-394	-0.668	0.00	-476	-0.896	Neither	32
1ZE3	HOH2032	2.43	-240	-0.289	1.25	-213	-0.227	3.67	-453	-0.835	Neither	35
	HOH2056	2.60	27	0.436	2.69	34	0.456	5.29	61	0.709	Both	21
	HOH2065	1.22	63	0.332	0.96	-120	-0.017	2.18	-57	0.271	Protein A	19
	HOH2067	1.17	-226	-0.256	1.14	65	0.321	2.31	-161	-0.108	Protein B	7
	HOH2076	2.26	-48	0.297	1.20	278	0.447	3.46	230	0.757	Both	42
	HOH2123	2.20	-230	-0.266	1.03	-2	0.253	3.24	-232	-0.270	Protein B	1

	HOH2155	0.00	-239	-0.256	0.00	-108	-0.040	0.00	-347	-0.541	Neither	31
	HOH2170	2.33	261	0.624	1.08	-71	0.206	3.41	190	0.730	Protein A	0
	HOH2180	1.27	-57	0.214	2.31	81	0.455	3.58	24	0.552	Protein B	0
	HOH2199	1.29	-154	-0.094	2.26	130	0.505	3.55	-24	0.503	Protein B	0
	HOH2224	1.93	-8	0.292	0.00	-237	-0.251	1.93	-244	-0.300	Protein A	19
	HOH2238	1.17	-163	-0.114	1.14	-48	0.229	2.31	-211	-0.223	Neither	28
	HOH2086	1.14	-201	-0.199	1.14	159	0.370	2.27	-42	0.308	Protein B	0
	HOH2028	4.07	111	0.686	1.51	192	0.472	5.58	303	0.945	Both	3
	HOH2035	1.48	-388	-0.661	3.92	108	0.671	5.40	-280	-0.386	Protein B	8
	HOH2036	1.24	94	0.363	2.42	130	0.526	3.66	224	0.784	Both	23
	HOH2055	1.20	10	0.282	2.22	401	0.706	3.42	412	0.823	Both	0
	HOH2064	2.63	-122	-0.021	2.51	-97	0.283	5.14	-219	-0.239	Protein B	20
	HOH2066	0.00	-333	-0.503	1.93	341	0.653	1.93	9	0.314	Protein B	39
	HOH2073	0.00	-370	-0.604	2.16	93	0.447	2.16	-277	-0.378	Protein B	19
	HOH2107	2.09	-221	-0.246	1.08	159	0.354	3.17	-62	0.411	Protein B	0
	HOH2113	0.00	-157	-0.075	0.76	-272	-0.365	0.76	-428	-0.768	Neither	12
	HOH2116	2.21	-181	-0.153	1.19	156	0.387	3.39	-25	0.480	Protein B	28
	HOH2128	2.39	-97	0.262	1.17	-90	0.188	3.56	-187	-0.166	Protein A	32
	HOH2144	1.25	34	0.308	3.38	-62	0.439	4.62	-28	0.597	Both	11
	HOH2145	0.00	-341	-0.525	1.71	290	0.583	1.71	-51	0.223	Protein B	1
	HOH2149	0.72	-85	0.134	0.00	-183	-0.123	0.72	-267	-0.355	Neither	15
1ZHH	HOH375	1.33	194	0.431	2.34	-67	0.290	3.67	127	0.669	Both	0
	HOH389	1.27	-13	0.262	2.21	-15	0.329	3.48	-28	0.489	Both	4
	HOH409	2.65	757	0.861	1.27	33	0.309	3.92	790	0.968	Both	0
	HOH420	2.52	364	0.707	1.23	-183	-0.158	3.75	181	0.766	Protein A	20
	HOH423	0.88	73	0.283	0.00	-205	-0.173	0.88	-132	-0.044	Protein A	15
	HOH459	3.45	114	0.623	0.00	-39	-0.039	3.45	75	0.583	Protein A	5
	HOH461	2.28	-20	0.336	1.07	-9	0.254	3.35	-29	0.469	Both	14
	HOH462	4.16	398	0.888	1.17	-22	0.252	5.33	376	0.957	Both	27
	HOH470	1.25	-42	0.232	1.88	81	0.403	3.13	39	0.511	Protein B	23
	HOH471	2.55	138	0.553	1.14	-370	-0.611	3.69	-231	-0.268	Protein A	37
	HOH474	1.16	-11	0.261	0.94	23	0.257	2.10	11	0.341	Both	0
	HOH504	1.44	560	0.700	2.30	17	0.382	3.74	577	0.912	Both	18
	HOH517	0.82	25	0.237	0.90	57	0.275	1.72	82	0.394	Protein B	35
	HOH530	0.00	-41	-0.039	0.76	34	0.232	0.76	-7	0.201	Neither	12
	HOH573	1.17	375	0.487	0.00	-238	-0.255	1.17	137	0.376	Protein A	10
	HOH582	0.88	12	0.238	0.95	-43	0.207	1.82	-32	0.256	Neither	13
	HOH608	1.05	78	0.316	1.05	69	0.309	2.10	148	0.506	Both	2
	HOH609	0.78	86	0.275	0.00	-107	-0.040	0.78	-21	0.196	Protein A	0
	HOH3262	1.42	513	0.673	3.85	28	0.597	5.27	541	1.012	Both	33
	HOH3269	2.56	488	0.774	1.36	-28	0.245	3.91	460	0.890	Protein A	22
	HOH3274	1.14	-111	0.003	1.20	60	0.327	2.34	-51	0.309	Protein B	22
	HOH3275	0.00	59	-0.038	2.17	44	0.393	2.17	103	0.460	Protein B	0
	HOH3276	0.93	-361	-0.588	1.20	95	0.356	2.12	-266	-0.352	Protein B	6
	HOH3281	1.17	-98	0.180	2.64	196	0.632	3.82	99	0.651	Protein B	1
	HOH3293	0.84	-17	0.210	0.90	-232	-0.270	1.75	-249	-0.311	Neither	18
	HOH3295	1.03	71	0.308	2.08	-83	0.211	3.11	-12	0.457	Protein A	4
	HOH3297	0.00	-119	-0.034	0.00	-67	-0.039	0.00	-186	-0.129	Neither	40
	HOH3304	0.87	134	0.312	1.17	12	0.282	2.04	146	0.497	Both	38
	HOH3319	1.19	-36	0.239	2.48	180	0.593	3.68	143	0.697	Protein B	44
	HOH3332	0.90	-116	-0.010	0.96	39	0.273	1.86	-77	0.195	Protein B	39
	HOH3333	1.01	64	0.298	0.97	-10	0.236	1.97	53	0.373	Protein A	26
	HOH3334	0.99	-84	0.181	2.48	100	0.496	3.47	16	0.530	Protein B	30
2A2Q	HOH1012	2.56	326	0.690	1.30	166	0.421	3.87	492	0.897	Both	0
	HOH1015	1.23	69	0.339	1.95	-277	-0.378	3.18	-208	-0.215	Protein A	4
	HOH1017	1.22	316	0.480	1.20	57	0.325	2.42	373	0.703	Both	7
	HOH1020	4.00	390	0.872	1.33	15	0.293	5.34	405	0.968	Both	3
	HOH1024	2.08	5	0.330	0.00	-57	-0.039	2.08	-52	0.256	Protein A	1
	HOH1025	2.74	-235	-0.279	1.30	-149	-0.082	4.04	-384	-0.649	Neither	1
	HOH1029	2.27	9	0.368	1.29	-129	-0.040	3.56	-120	-0.013	Protein A	5

	HOH1036	2.36	125	0.512	0.00	-94	-0.040	2.36	31	0.408	Protein A	7
	HOH1038	1.04	-8	0.250	0.92	2	0.238	1.96	-6	0.295	Protein A	5
	HOH1040	1.26	-8	0.267	2.27	177	0.561	3.54	169	0.719	Both	4
	HOH1055	0.87	-11	0.219	0.00	-82	-0.040	0.87	-93	0.153	Neither	0
	HOH1067	1.10	98	0.337	0.00	-296	-0.406	1.10	-198	-0.192	Protein A	3
	HOH1082	1.02	69	0.304	0.95	88	0.304	1.97	157	0.502	Both	9
	HOH1094	0.83	17	0.232	0.00	-130	-0.041	0.83	-113	-0.003	Neither	0
	HOH1098	1.08	-273	-0.368	0.82	-78	0.156	1.89	-351	-0.563	Neither	1
	HOH1104	0.98	-200	-0.196	0.93	-65	0.186	1.90	-264	-0.348	Neither	23
	HOH1121	1.33	437	0.602	2.31	-22	0.338	3.65	414	0.847	Both	7
	HOH1122	0.00	-259	-0.309	0.80	51	0.252	0.80	-208	-0.215	Protein B	5
	HOH1148	0.80	75	0.269	0.00	-90	-0.040	0.80	-15	0.203	Protein A	6
	HOH1151	1.19	25	0.295	1.02	106	0.329	2.21	131	0.501	Both	1
	HOH1018	2.64	120	0.540	1.30	376	0.555	3.95	496	0.906	Both	4
	HOH1019	1.16	110	0.360	1.08	82	0.323	2.24	192	0.569	Both	5
	HOH1033	2.67	323	0.700	1.26	106	0.377	3.93	429	0.881	Both	6
	HOH1065	2.88	622	0.850	2.38	-56	0.310	5.26	566	1.019	Both	43
	HOH1083	1.20	254	0.429	0.97	-89	0.174	2.17	165	0.536	Protein A	7
	HOH1143	2.49	2	0.394	0.98	-6	0.242	3.47	-3	0.512	Protein A	9
	HOH1194	0.00	-110	-0.029	0.77	-50	0.170	0.77	-160	-0.108	Neither	10
	HOH1231	1.23	204	0.403	1.17	-398	-0.686	2.41	-193	-0.182	Protein A	7
	HOH1295	0.88	63	0.276	0.00	-172	-0.101	0.88	-108	0.142	Protein A	41
	HOH1298	0.77	18	0.222	0.00	-33	-0.039	0.77	-15	0.197	Neither	21
	HOH1016	1.26	-224	-0.251	2.45	343	0.688	3.71	120	0.663	Protein B	27
	HOH1021	2.10	30	0.364	1.34	-86	0.176	3.44	-56	0.455	Protein A	20
	HOH1022	0.00	5	-0.039	1.90	28	0.339	1.90	34	0.346	Protein B	37
	HOH1027	0.00	-61	-0.039	2.24	-8	0.344	2.24	-69	0.268	Protein B	20
	HOH1028	0.86	-378	-0.633	1.13	297	0.423	1.99	-81	0.192	Protein B	24
	HOH1030	1.03	-102	0.173	2.25	202	0.578	3.27	101	0.585	Protein B	37
	HOH1031	3.12	-221	-0.245	0.00	441	-0.037	3.12	221	0.710	Neither	32
	HOH1035	0.90	-534	-1.066	1.19	67	0.331	2.08	-467	-0.874	Protein B	29
	HOH1043	1.07	13	0.271	2.41	272	0.640	3.48	285	0.775	Both	5
	HOH1049	1.18	82	0.343	1.26	44	0.319	2.44	126	0.523	Both	9
	HOH1053	0.00	-113	-0.030	0.00	-184	-0.126	0.00	-297	-0.408	Neither	39
	HOH1080	0.00	52	-0.038	1.78	-182	-0.157	1.78	-130	-0.043	Neither	23
	HOH1088	0.00	-33	-0.039	2.32	294	0.644	2.32	261	0.623	Protein B	7
	HOH1091	1.97	-158	-0.103	1.23	86	0.355	3.20	-72	0.406	Protein B	1
	HOH1107	1.13	183	0.368	1.21	436	0.536	2.34	619	0.809	Both	11
	HOH1115	0.00	-319	-0.466	1.10	16	0.278	1.10	-303	-0.442	Protein B	49
	HOH1117	1.11	-10	0.260	2.28	41	0.406	3.39	31	0.534	Both	3
	HOH1118	1.15	-10	0.262	4.11	309	0.850	5.26	299	0.929	Both	38
	HOH1131	0.94	-5	0.236	0.98	105	0.321	1.93	99	0.428	Protein B	25
	HOH1136	2.22	438	0.727	1.19	-117	-0.012	3.42	321	0.782	Protein A	28
	HOH1142	1.05	-45	0.223	1.17	453	0.520	2.22	408	0.710	Protein B	41
	HOH1149	1.35	-152	-0.090	2.38	237	0.616	3.73	85	0.627	Protein B	56
	HOH1169	0.84	-46	0.185	1.09	107	0.340	1.92	60	0.380	Protein B	24
	HOH1173	2.04	166	0.518	1.36	-78	0.185	3.39	88	0.587	Protein A	0
	HOH1178	1.07	271	0.387	1.18	21	0.290	2.24	292	0.635	Both	7
	HOH1188	0.00	-45	-0.039	0.88	359	0.396	0.88	314	0.373	Protein B	38
	HOH1206	1.23	151	0.399	2.53	218	0.626	3.76	369	0.840	Both	42
2A9K	HOH506	3.41	110	0.612	0.00	-257	-0.304	3.41	-147	-0.076	Protein A	2
	HOH516	2.66	533	0.800	1.10	-504	-0.980	3.76	29	0.584	Protein A	0
	HOH522	1.29	491	0.606	2.07	-97	0.189	3.35	394	0.809	Protein A	7
	HOH527	4.23	-67	0.536	1.29	-282	-0.390	5.52	-349	-0.558	Protein A	0
	HOH532	1.07	-344	-0.546	1.03	325	0.410	2.11	-20	0.303	Protein B	0
	HOH535	1.00	-213	-0.225	0.00	-86	-0.040	1.00	-298	-0.430	Neither	2
	HOH548	2.51	358	0.703	1.03	-92	0.181	3.54	266	0.775	Protein A	13
	HOH552	2.41	280	0.645	1.17	-166	-0.119	3.59	115	0.640	Protein A	11
	HOH563	1.78	36	0.342	0.00	-52	-0.039	1.78	-17	0.274	Protein A	21
	HOH402	1.23	-128	-0.036	2.65	104	0.520	3.88	-24	0.551	Protein B	28

	HOH407	1.30	-26	0.247	3.87	-41	0.538	5.16	-67	0.588	Protein B	52
	HOH410	1.14	-131	-0.042	1.19	32	0.301	2.33	-99	0.246	Protein B	4
	HOH420	3.48	-142	-0.065	1.28	-53	0.218	4.76	-195	-0.185	Neither	10
	HOH428	2.14	-101	0.202	1.11	-2	0.266	3.25	-103	0.378	Protein B	7
	HOH437	0.92	-76	0.177	1.17	73	0.333	2.09	-3	0.322	Protein B	31
	HOH440	2.09	68	0.409	1.21	9	0.282	3.31	77	0.567	Both	0
	HOH483	0.00	-311	-0.445	1.01	-112	0.001	1.01	-422	-0.752	Neither	26
	HOH488	2.38	400	0.715	1.39	-123	-0.026	3.77	277	0.808	Protein A	0
	HOH490	2.31	105	0.481	2.48	39	0.433	4.79	144	0.790	Both	17
	HOH494	0.00	-80	-0.040	0.00	-28	-0.039	0.00	-108	-0.040	Neither	6
	HOH505	0.00	164	-0.038	3.47	-60	0.455	3.47	104	0.612	Protein B	11
2AQ2	HOH1	2.63	415	0.745	1.25	120	0.386	3.87	534	0.911	Both	0
	HOH2	1.35	35	0.316	2.64	115	0.533	3.99	150	0.744	Both	7
	HOH3	4.22	60	0.650	1.46	-214	-0.230	5.68	-154	-0.090	Protein A	12
	HOH4	1.35	-161	-0.109	2.22	-106	0.214	3.57	-267	-0.353	Neither	4
	HOH9	1.50	468	0.674	3.97	83	0.650	5.47	551	1.024	Both	41
	HOH10	2.17	524	0.770	2.51	2	0.397	4.67	527	0.973	Both	7
	HOH11	1.12	155	0.364	0.83	-23	0.203	1.95	132	0.471	Protein A	11
	HOH14	1.23	-144	-0.072	1.06	-149	-0.081	2.29	-293	-0.417	Neither	14
	HOH16	0.98	-39	0.216	2.05	-2	0.316	3.03	-41	0.417	Protein B	17
	HOH19	1.24	225	0.421	2.30	129	0.509	3.54	354	0.810	Both	14
	HOH21	1.12	89	0.337	0.87	-19	0.212	1.99	70	0.396	Protein A	11
	HOH27	2.12	18	0.352	0.96	6	0.248	3.08	24	0.490	Protein A	6
	HOH45	0.87	-51	0.188	0.85	-47	0.187	1.73	-98	0.158	Neither	0
	HOH59	0.93	75	0.293	0.83	15	0.232	1.77	90	0.407	Protein A	27
	HOH100	2.24	-93	0.236	1.40	178	0.447	3.64	86	0.616	Protein B	4
	HOH108	0.94	-137	-0.055	2.15	-52	0.271	3.10	-189	-0.170	Protein B	29
	HOH113	1.37	15	0.294	3.92	-78	0.503	5.29	-63	0.597	Both	0
	HOH120	0.00	-67	-0.039	2.00	392	0.698	2.00	325	0.646	Protein B	3
	HOH133	0.84	20	0.237	0.91	-75	0.175	1.74	-54	0.221	Neither	5
	HOH154	0.00	-16	-0.039	3.49	89	0.600	3.49	73	0.586	Protein B	47
	HOH229	0.00	-243	-0.268	1.95	71	0.395	1.95	-172	-0.134	Protein B	0
2ARP	HOH3	1.18	-174	-0.137	0.97	104	0.318	2.14	-69	0.245	Protein B	5
	HOH14	1.10	-101	0.186	1.07	-52	0.221	2.17	-153	-0.091	Neither	5
	HOH19	1.44	96	0.390	2.66	-494	-0.951	4.10	-398	-0.685	Protein A	11
	HOH21	1.00	63	0.296	0.89	-205	-0.209	1.89	-142	-0.069	Protein A	1
	HOH22	1.11	16	0.280	1.97	18	0.327	3.08	34	0.499	Both	13
	HOH25	1.89	67	0.387	0.00	-602	-1.269	1.89	-534	-1.069	Protein A	1
	HOH34	1.25	487	0.581	1.11	-9	0.261	2.35	478	0.754	Both	47
	HOH37	1.13	34	0.296	2.04	225	0.566	3.17	259	0.727	Both	43
	HOH41	1.18	-430	-0.772	1.11	154	0.359	2.28	-276	-0.376	Protein B	18
	HOH43	0.97	-233	-0.273	0.00	-267	-0.329	0.97	-500	-0.968	Neither	7
	HOH46	0.89	291	0.363	0.80	-124	-0.027	1.69	168	0.483	Protein A	35
	HOH48	1.19	-173	-0.136	1.12	-58	0.221	2.31	-231	-0.269	Neither	36
	HOH53	3.17	-81	0.392	0.00	-52	-0.039	3.17	-132	-0.043	Protein A	0
	HOH77	0.00	-129	-0.040	0.74	-18	0.191	0.74	-147	-0.077	Neither	8
	HOH94	1.13	-243	-0.297	2.06	-165	-0.118	3.19	-408	-0.713	Neither	45
	HOH111	2.26	-87	0.247	1.30	100	0.378	3.56	13	0.539	Protein B	15
	HOH118	0.00	-33	-0.039	3.31	7	0.500	3.31	-26	0.467	Protein B	14
	HOH130	2.23	-99	0.225	1.22	511	0.576	3.45	412	0.826	Protein B	34
	HOH148	1.23	-78	0.194	2.24	565	0.787	3.48	488	0.859	Protein B	0
2B2X	HOH7	1.47	91	0.387	2.51	78	0.478	3.99	170	0.776	Both	2
	HOH10	2.51	282	0.657	1.19	-148	-0.081	3.70	133	0.683	Protein A	0
	HOH11	2.36	30	0.407	1.07	-166	-0.120	3.43	-136	-0.050	Protein A	11
	HOH13	5.13	106	0.742	0.00	-98	-0.040	5.13	8	0.656	Protein A	7
	HOH67	0.00	64	-0.038	3.52	154	0.694	3.52	218	0.764	Protein B	8
	HOH68	1.29	107	0.382	3.97	-77	0.507	5.26	30	0.681	Both	0
	HOH74	1.20	-19	0.255	2.36	-30	0.337	3.55	-49	0.479	Both	0
	HOH108	1.34	81	0.364	4.00	-7	0.576	5.35	74	0.721	Both	15
2BCG	HOH3	2.57	-301	-0.436	1.26	55	0.330	3.83	-245	-0.302	Protein B	26

	HOH11	5.27	242	0.923	0.00	-39	-0.039	5.27	202	0.926	Protein A	2
	HOH15	1.42	-167	-0.122	4.08	-47	0.544	5.50	-214	-0.227	Protein B	19
	HOH37	2.35	41	0.416	1.04	-41	0.225	3.39	0	0.504	Protein A	0
	HOH46	1.00	-75	0.191	0.89	20	0.246	1.89	-55	0.225	Neither	20
	HOH56	1.27	38	0.314	2.15	-197	-0.190	3.42	-159	-0.102	Protein A	15
	HOH107	1.23	128	0.388	2.02	74	0.405	3.25	202	0.723	Both	10
	HOH141	0.90	124	0.316	0.87	-101	0.146	1.77	24	0.326	Protein A	17
	HOH150	1.13	-73	0.208	1.04	-23	0.238	2.16	-96	0.215	Neither	9
	HOH168	3.48	67	0.579	1.27	213	0.422	4.75	280	0.894	Both	16
	HOH175	2.41	-112	0.000	1.19	136	0.380	3.60	24	0.556	Protein B	12
	HOH176	0.90	146	0.318	0.88	29	0.250	1.78	174	0.499	Both	29
	HOH179	3.86	189	0.793	1.20	52	0.321	5.06	241	0.911	Both	19
	HOH221	2.59	233	0.642	1.27	155	0.409	3.85	388	0.858	Both	32
	HOH240	2.41	115	0.506	1.32	-93	0.169	3.73	22	0.573	Protein A	38
	HOH244	0.92	-130	-0.039	0.84	-2	0.220	1.75	-132	-0.045	Neither	0
	HOH276	0.71	84	0.260	0.00	-63	-0.039	0.71	21	0.215	Protein A	41
	HOH277	0.00	-297	-0.409	0.70	-6	0.193	0.70	-303	-0.443	Neither	7
	HOH292	1.15	392	0.482	0.91	-64	0.184	2.06	328	0.650	Protein A	30
	HOH307	0.00	-288	-0.385	0.74	14	0.215	0.74	-275	-0.372	Neither	22
	HOH316	2.36	-187	-0.168	0.00	-187	-0.133	2.36	-375	-0.624	Neither	12
	HOH318	1.32	13	0.291	0.95	-112	-0.001	2.27	-99	0.234	Protein A	27
	HOH336	2.05	68	0.402	0.00	-287	-0.383	2.05	-219	-0.242	Protein A	0
	HOH379	1.06	249	0.373	0.88	45	0.262	1.94	294	0.615	Both	46
	HOH498	0.95	172	0.326	0.00	-100	-0.040	0.95	72	0.294	Protein A	9
	HOH589	0.00	125	-0.038	0.72	-41	0.168	0.72	84	0.262	Neither	42
	HOH606	1.10	-13	0.257	1.19	-136	-0.054	2.30	-149	-0.083	Protein A	6
	HOH610	2.26	80	0.447	1.31	-113	-0.004	3.57	-33	0.498	Protein A	2
	HOH620	1.18	-164	-0.116	2.13	-86	0.220	3.31	-250	-0.313	Neither	1
	HOH629	1.10	-19	0.253	1.99	-107	0.151	3.09	-126	-0.028	Protein A	14
	HOH636	1.11	-22	0.250	1.17	148	0.379	2.28	126	0.503	Both	24
	HOH639	1.29	34	0.311	4.05	25	0.607	5.35	58	0.709	Both	0
	HOH642	0.00	-167	-0.093	1.89	-156	-0.098	1.89	-323	-0.493	Neither	10
	HOH661	1.14	-26	0.248	3.93	-4	0.572	5.06	-30	0.618	Protein B	11
	HOH670	3.86	-388	-0.660	1.48	-23	0.252	5.34	-411	-0.720	Protein B	0
	HOH673	2.25	100	0.467	1.20	3	0.276	3.46	103	0.609	Both	4
	HOH726	1.19	-222	-0.248	2.33	63	0.438	3.52	-159	-0.103	Protein B	0
	HOH738	0.00	-134	-0.046	1.94	13	0.319	1.94	-122	-0.024	Protein B	13
	HOH748	0.00	-14	-0.039	0.77	-7	0.204	0.77	-20	0.193	Neither	4
	HOH773	0.00	-82	-0.040	0.96	8	0.250	0.96	-74	0.185	Protein B	41
	HOH810	0.82	-167	-0.123	1.07	24	0.280	1.89	-144	-0.071	Protein B	28
	HOH818	0.98	0	0.247	2.43	214	0.611	3.41	215	0.749	Protein B	33
	HOH826	0.00	-200	-0.162	0.75	28	0.227	0.75	-172	-0.134	Neither	50
	HOH827	0.00	-40	-0.039	0.78	15	0.223	0.78	-26	0.192	Neither	37
	HOH843	1.06	-2	0.258	2.51	-327	-0.501	3.57	-329	-0.507	Protein A	0
	HOH850	0.94	-269	-0.358	1.19	73	0.336	2.12	-196	-0.188	Protein B	20
	HOH883	0.00	-71	-0.040	3.33	40	0.535	3.33	-31	0.465	Protein B	0
	HOH904	0.81	-225	-0.255	1.12	92	0.338	1.93	-133	-0.049	Protein B	34
2BEX	HOH2002	2.31	496	0.761	1.21	101	0.365	3.53	597	0.899	Both	27
	HOH2004	2.35	244	0.616	1.07	85	0.324	3.43	329	0.787	Both	27
	HOH2005	0.94	67	0.288	0.85	-140	-0.063	1.79	-73	0.196	Protein A	27
	HOH2048	0.71	-104	0.116	0.00	-200	-0.160	0.71	-303	-0.442	Neither	29
	HOH2051	0.83	114	0.300	0.00	-193	-0.145	0.83	-79	0.158	Protein A	7
	HOH2054	1.26	202	0.412	2.05	-34	0.273	3.31	168	0.689	Both	0
	HOH2073	0.82	137	0.302	0.79	-313	-0.467	1.60	-176	-0.144	Protein A	3
	HOH2151	2.12	355	0.672	0.98	-237	-0.283	3.10	118	0.589	Protein A	0
	HOH2153	1.17	505	0.542	1.13	-281	-0.387	2.31	225	0.599	Protein A	16
	HOH2181	1.02	45	0.286	3.57	-78	0.453	4.59	-33	0.591	Both	21
	HOH2195	1.07	372	0.441	0.00	-266	-0.328	1.07	106	0.338	Protein A	11
	HOH2230	1.07	553	0.496	0.93	-167	-0.121	2.00	386	0.693	Protein A	31
	HOH2232	3.85	102	0.659	1.43	-22	0.252	5.28	80	0.723	Both	12

	HOH2233	1.34	104	0.388	2.23	-25	0.321	3.57	80	0.602	Both	5
	HOH2247	2.01	154	0.503	1.27	-33	0.240	3.28	121	0.612	Protein A	5
	HOH2248	1.75	263	0.564	0.00	-207	-0.179	1.75	56	0.365	Protein A	22
	HOH2249	1.38	442	0.624	2.34	89	0.467	3.72	531	0.897	Both	0
	HOH2251	2.45	-164	-0.116	1.07	67	0.311	3.52	-97	0.423	Protein B	0
	HOH2252	0.95	3	0.243	0.84	20	0.237	1.79	23	0.326	Neither	20
	HOH2254	1.16	116	0.363	0.87	-57	0.182	2.03	59	0.388	Protein A	25
	HOH2001	0.85	-254	-0.323	0.86	-6	0.220	1.71	-260	-0.337	Neither	3
	HOH2014	4.06	216	0.831	1.46	149	0.442	5.53	365	0.962	Both	20
	HOH2019	1.19	-6	0.267	3.99	211	0.825	5.18	205	0.925	Both	6
	HOH2022	0.00	-42	-0.039	4.92	-14	0.626	4.92	-55	0.588	Protein B	19
	HOH2023	1.41	59	0.346	4.19	-38	0.560	5.60	21	0.686	Both	15
	HOH2026	1.13	-120	-0.018	1.16	25	0.292	2.29	-95	0.245	Protein B	10
	HOH2027	1.01	32	0.275	2.11	-170	-0.130	3.12	-139	-0.057	Protein A	13
	HOH2029	1.12	262	0.398	2.44	4	0.390	3.56	266	0.778	Both	7
	HOH2041	1.34	550	0.656	2.37	-67	0.295	3.71	484	0.880	Both	0
	HOH2042	2.18	-41	0.290	2.77	262	0.679	4.95	220	0.906	Both	0
	HOH2043	1.13	-40	0.236	2.67	111	0.531	3.80	71	0.626	Protein B	9
	HOH2047	2.21	-35	0.304	1.15	-57	0.221	3.36	-92	0.406	Protein A	16
	HOH2049	0.00	-361	-0.578	1.95	-75	0.197	1.95	-436	-0.789	Neither	11
	HOH2050	4.79	-109	0.528	0.00	70	-0.038	4.79	-39	0.596	Protein A	5
	HOH2055	4.00	47	0.622	1.53	66	0.363	5.53	113	0.768	Both	37
	HOH2056	1.06	42	0.291	1.08	-113	-0.001	2.14	-71	0.244	Protein A	21
2BKK	HOH69	1.18	295	0.445	2.08	-211	-0.222	3.25	85	0.567	Protein A	0
	HOH74	0.00	-52	-0.039	1.82	-49	0.232	1.82	-101	0.158	Neither	22
	HOH107	4.06	201	0.828	1.31	-15	0.260	5.37	187	0.907	Both	7
	HOH118	0.90	-515	-1.012	1.20	-35	0.240	2.09	-550	-1.116	Neither	20
	HOH120	0.86	-296	-0.426	1.15	285	0.428	2.01	-11	0.294	Protein B	10
	HOH122	1.05	10	0.265	1.05	-96	0.181	2.10	-86	0.211	Protein A	36
	HOH136	1.02	-59	0.207	2.36	-150	-0.084	3.38	-209	-0.216	Neither	7
	HOH137	1.06	2	0.261	1.12	235	0.382	2.18	237	0.590	Both	16
	HOH138	0.00	170	-0.038	5.28	108	0.752	5.28	279	0.926	Protein B	0
	HOH141	1.01	-245	-0.302	2.56	-1419	-4.449	3.58	-1664	-5.665	Neither	11
	HOH144	1.11	-72	0.210	3.83	-72	0.502	4.93	-143	-0.065	Protein B	3
	HOH148	0.00	-98	-0.040	1.06	-272	-0.365	1.06	-370	-0.612	Neither	8
	HOH225	0.00	-69	-0.039	0.74	-127	-0.034	0.74	-195	-0.186	Neither	60
	HOH279	1.38	69	0.356	2.34	81	0.459	3.72	150	0.715	Both	19
	HOH283	1.08	407	0.456	0.86	-23	0.207	1.94	384	0.686	Protein A	2
	HOH288	0.87	-57	0.183	0.79	-18	0.199	1.66	-75	0.188	Neither	13
2BO9	HOH81	0.00	-204	-0.169	0.71	4	0.203	0.71	-199	-0.195	Neither	20
	HOH115	1.11	-264	-0.346	0.00	-173	-0.104	1.11	-437	-0.792	Neither	30
	HOH120	1.21	-183	-0.159	0.94	-189	-0.173	2.15	-373	-0.619	Neither	3
	HOH123	2.12	-177	-0.145	0.96	22	0.259	3.08	-155	-0.095	Protein B	1
	HOH130	0.00	-17	-0.039	0.71	-80	0.136	0.71	-96	0.122	Neither	9
	HOH131	0.00	-275	-0.351	0.00	15	-0.039	0.00	-261	-0.314	Neither	24
	HOH188	1.15	-281	-0.387	1.12	114	0.352	2.27	-166	-0.121	Protein B	0
	HOH274	5.17	128	0.780	0.00	8	-0.039	5.17	136	0.796	Protein A	2
	HOH635	1.07	-66	0.209	0.86	23	0.243	1.93	-44	0.243	Neither	27
	HOH636	2.15	-360	-0.587	1.01	9	0.258	3.16	-351	-0.564	Protein B	3
	HOH738	4.32	667	0.983	1.31	-55	0.215	5.63	612	1.047	Protein A	13
	HOH779	3.39	36	0.539	1.14	94	0.345	4.53	130	0.748	Both	7
	HOH782	0.97	53	0.283	0.73	-6	0.199	1.70	47	0.351	Protein A	33
	HOH823	1.22	1	0.275	1.20	504	0.556	2.41	506	0.770	Both	16
	HOH825	2.22	308	0.644	1.00	50	0.287	3.22	358	0.778	Both	1
	HOH826	3.65	498	0.880	1.12	-21	0.251	4.77	477	0.962	Both	0
	HOH863	3.54	324	0.797	1.34	-26	0.248	4.88	298	0.907	Protein A	14
	HOH864	2.03	59	0.388	1.07	-201	-0.200	3.09	-143	-0.066	Protein A	13
	HOH865	1.02	119	0.335	0.00	-321	-0.471	1.02	-202	-0.200	Protein A	10
	HOH866	2.29	-35	0.319	0.00	-163	-0.085	2.29	-198	-0.192	Protein A	6
	HOH905	1.18	108	0.364	0.98	7	0.251	2.16	115	0.474	Both	6

	HOH906	3.84	232	0.809	0.00	-224	-0.220	3.84	8	0.578	Protein A	45
	HOH907	2.24	-67	0.270	1.10	-42	0.235	3.34	-108	0.384	Protein A	33
	HOH908	1.12	-166	-0.120	1.11	22	0.285	2.23	-144	-0.073	Protein B	1
	HOH910	1.44	-109	0.003	4.10	249	0.836	5.54	140	0.820	Protein B	8
	HOH923	5.11	152	0.827	0.00	122	-0.038	5.11	274	0.916	Protein A	38
	HOH924	2.63	-142	-0.065	2.56	180	0.605	5.20	39	0.685	Protein B	3
	HOH925	1.35	-193	-0.182	2.15	143	0.507	3.50	-51	0.468	Protein B	0
	HOH926	2.33	57	0.432	1.22	25	0.297	3.55	82	0.601	Both	22
	HOH927	0.92	-168	-0.125	0.00	71	-0.038	0.92	-97	0.158	Neither	30
	HOH928	1.11	82	0.329	0.88	-320	-0.483	1.99	-237	-0.284	Protein A	27
	HOH932	3.36	-138	-0.056	0.00	-366	-0.591	3.36	-504	-0.980	Neither	19
	HOH966	5.20	459	0.981	0.00	-5	-0.039	5.20	455	0.980	Protein A	16
	HOH974	2.20	41	0.394	1.19	-246	-0.304	3.39	-205	-0.209	Protein A	4
	HOH976	0.00	-282	-0.368	1.97	4	0.309	1.97	-278	-0.379	Protein B	1
	HOH979	3.91	-38	0.540	1.46	155	0.446	5.37	117	0.769	Both	5
	HOH980	0.00	-128	-0.040	1.02	34	0.279	1.02	-94	0.178	Protein B	0
	HOH988	0.00	-171	-0.099	1.01	126	0.336	1.01	-45	0.217	Protein B	15
	HOH1007	0.00	-171	-0.099	1.05	139	0.345	1.05	-32	0.233	Protein B	11
	HOH1010	0.00	-290	-0.390	0.00	-1	-0.039	0.00	-291	-0.392	Neither	10
	HOH1056	1.10	20	0.282	1.21	113	0.372	2.31	133	0.515	Both	22
	HOH1058	2.66	80	0.498	2.41	59	0.444	5.07	139	0.797	Both	19
	HOH1059	1.12	-193	-0.179	2.57	262	0.654	3.68	69	0.608	Protein B	3
	HOH1060	1.25	34	0.308	2.37	-31	0.338	3.62	3	0.539	Both	9
	HOH1091	1.17	-196	-0.188	1.21	-203	-0.204	2.38	-399	-0.690	Neither	8
	HOH1092	0.00	-293	-0.399	5.23	250	0.921	5.23	-43	0.613	Protein B	10
	HOH1093	0.00	-263	-0.320	0.82	-59	0.172	0.82	-322	-0.490	Neither	0
	HOH1095	1.12	-69	0.212	3.71	-53	0.500	4.84	-121	-0.014	Protein B	34
	HOH1110	0.00	-204	-0.171	1.04	-349	-0.557	1.04	-553	-1.123	Neither	1
	HOH1111	2.00	-96	0.171	1.21	52	0.321	3.21	-44	0.436	Protein B	0
	HOH1112	0.00	-119	-0.033	1.02	-8	0.246	1.02	-127	-0.033	Neither	1
	HOH1114	1.32	-96	0.165	2.72	402	0.747	4.04	305	0.844	Protein B	19
	HOH1134	0.87	-357	-0.579	1.19	-204	-0.206	2.06	-561	-1.148	Neither	16
	HOH1136	1.52	263	0.531	4.44	456	0.933	5.97	719	1.080	Both	4
	HOH1139	1.16	-136	-0.054	2.34	263	0.626	3.50	127	0.647	Protein B	25
	HOH1140	1.30	122	0.397	2.46	38	0.430	3.77	160	0.737	Both	4
2CIO	HOH17	1.99	108	0.443	0.00	-115	-0.031	1.99	-7	0.296	Protein A	6
	HOH52	0.73	-65	0.152	0.00	-231	-0.237	0.73	-296	-0.424	Neither	4
	HOH159	1.98	-441	-0.803	1.16	120	0.365	3.14	-321	-0.487	Protein B	17
	HOH161	2.32	39	0.410	1.32	25	0.303	3.64	64	0.597	Both	47
2CO7	HOH3	2.48	65	0.460	1.23	-82	0.189	3.71	-17	0.534	Protein A	11
	HOH36	0.85	206	0.297	0.84	-3	0.220	1.69	203	0.504	Protein A	18
	HOH38	1.20	-32	0.243	1.06	-115	-0.007	2.25	-147	-0.078	Neither	30
	HOH46	0.97	-123	-0.025	0.86	-67	0.172	1.83	-190	-0.174	Neither	31
	HOH49	1.24	-33	0.241	1.20	-16	0.258	2.44	-49	0.328	Protein B	25
	HOH51	2.17	64	0.415	0.00	-339	-0.521	2.17	-276	-0.375	Protein A	29
	HOH52	2.09	-494	-0.951	0.00	-208	-0.181	2.09	-702	-1.590	Neither	29
	HOH53	2.24	-248	-0.308	1.04	-185	-0.162	3.28	-433	-0.780	Neither	29
	HOH58	0.00	-307	-0.434	1.97	-35	0.255	1.97	-342	-0.540	Protein B	20
	HOH60	2.50	249	0.637	1.29	-387	-0.658	3.79	-139	-0.055	Protein A	9
	HOH66	1.49	10	0.293	4.04	79	0.653	5.54	89	0.741	Both	20
	HOH77	0.00	-261	-0.313	0.00	-309	-0.439	0.00	-569	-1.171	Neither	2
	HOH101	0.72	-32	0.175	0.00	-71	-0.040	0.72	-103	0.117	Neither	11
	HOH108	0.79	-12	0.204	1.12	-126	-0.030	1.91	-138	-0.059	Neither	12
	HOH114	0.96	17	0.255	1.17	39	0.305	2.13	56	0.400	Both	24
	HOH117	2.15	159	0.526	1.25	153	0.402	3.40	313	0.776	Both	2
	HOH118	0.00	-95	-0.040	0.78	63	0.257	0.78	-32	0.186	Protein B	4
	HOH122	0.00	26	-0.038	3.52	54	0.572	3.52	80	0.595	Protein B	0
	HOH184	0.00	-89	-0.040	0.00	-331	-0.499	0.00	-420	-0.740	Neither	54
	HOH186	1.12	230	0.379	2.32	-31	0.328	3.44	199	0.745	Both	25
	HOH187	2.40	384	0.708	2.59	33	0.441	4.99	417	0.954	Both	47

	HOH196	1.27	-39	0.234	3.90	287	0.824	5.17	248	0.917	Protein B	1
	HOH201	0.86	-146	-0.076	0.97	-53	0.204	1.83	-199	-0.194	Neither	0
	HOH235	1.14	4	0.273	1.30	-256	-0.328	2.44	-252	-0.318	Protein A	0
	HOH236	1.04	-9	0.250	2.45	-129	-0.038	3.49	-138	-0.056	Protein A	0
	HOH241	1.26	84	0.357	2.51	15	0.412	3.78	99	0.646	Both	36
	HOH272	1.90	1	0.302	0.00	21	-0.039	1.90	22	0.330	Protein A	0
	HOH273	0.00	-107	-0.040	1.70	-314	-0.469	1.70	-421	-0.748	Neither	0
	HOH274	1.29	47	0.325	3.97	-89	0.496	5.25	-42	0.616	Both	13
2DFK	HOH2	1.92	-33	0.257	0.00	-35	-0.039	1.92	-69	0.207	Protein A	10
	HOH11	2.16	135	0.500	0.96	-25	0.224	3.13	110	0.581	Protein A	27
	HOH15	0.00	-67	-0.039	0.78	-91	0.139	0.78	-158	-0.102	Neither	17
	HOH28	1.15	-94	0.187	0.92	105	0.311	2.07	11	0.336	Protein B	19
	HOH32	1.22	-131	-0.043	0.89	-96	0.153	2.11	-227	-0.258	Neither	30
	HOH39	1.17	-220	-0.243	1.01	10	0.258	2.18	-210	-0.221	Protein B	0
	HOH55	0.99	-90	0.176	0.00	-221	-0.211	0.99	-311	-0.461	Neither	0
	HOH71	3.73	177	0.758	0.00	-225	-0.222	3.73	-48	0.507	Protein A	0
	HOH76	2.09	283	0.616	0.96	-84	0.176	3.05	199	0.692	Protein A	0
	HOH79	0.00	-264	-0.322	0.71	-164	-0.115	0.71	-428	-0.766	Neither	4
	HOH97	0.94	-136	-0.054	0.00	-164	-0.086	0.94	-300	-0.434	Neither	1
	HOH111	2.02	424	0.719	0.00	-154	-0.070	2.02	270	0.602	Protein A	0
	HOH146	1.04	-124	-0.026	0.00	-97	-0.040	1.04	-220	-0.243	Neither	0
	HOH157	0.00	-159	-0.078	0.00	-241	-0.262	0.00	-400	-0.683	Neither	2
	HOH175	1.17	-238	-0.286	2.29	-51	0.300	3.46	-289	-0.407	Protein B	4
	HOH178	0.00	-14	-0.039	5.38	227	0.931	5.38	213	0.935	Protein B	10
	HOH181	1.43	-199	-0.196	4.35	-77	0.533	5.78	-277	-0.377	Protein B	15
	HOH185	1.28	-102	0.163	2.56	110	0.517	3.84	8	0.578	Protein B	0
	HOH189	0.88	-89	0.157	1.01	507	0.474	1.89	418	0.703	Protein B	30
	HOH190	0.00	-58	-0.039	2.37	-54	0.311	2.37	-112	-0.001	Protein B	5
	HOH192	2.05	-91	0.194	1.25	13	0.288	3.30	-78	0.412	Protein B	13
	HOH195	0.98	-79	0.183	2.15	-641	-1.394	3.12	-721	-1.650	Neither	39
	HOH197	2.90	-90	0.346	2.68	316	0.697	5.58	225	0.942	Both	2
	HOH201	0.00	-28	-0.039	3.77	210	0.799	3.77	182	0.772	Protein B	11
	HOH210	1.28	100	0.375	2.40	-134	-0.048	3.68	-34	0.513	Protein A	25
	HOH218	1.99	-437	-0.793	1.16	71	0.330	3.15	-367	-0.604	Protein B	3
	HOH224	0.00	-244	-0.271	1.97	-87	0.180	1.97	-331	-0.513	Neither	25
	HOH231	0.00	-285	-0.377	3.38	14	0.517	3.38	-271	-0.363	Protein B	0
	HOH232	0.90	-170	-0.128	1.03	175	0.343	1.93	5	0.309	Protein B	5
	HOH233	1.10	-227	-0.259	1.12	52	0.308	2.21	-175	-0.141	Protein B	1
	HOH234	1.92	26	0.336	1.20	46	0.315	3.12	72	0.541	Both	4
	HOH239	2.42	-245	-0.301	1.25	25	0.299	3.67	-220	-0.243	Protein B	14
	HOH262	0.92	5	0.240	1.17	426	0.508	2.09	431	0.721	Protein B	1
	HOH263	0.88	-292	-0.414	2.32	325	0.664	3.20	34	0.514	Protein B	15
	HOH265	2.35	63	0.442	2.82	317	0.714	5.17	381	0.951	Both	9
	HOH275	2.57	-113	-0.002	1.19	-31	0.244	3.76	-144	-0.068	Neither	22
	HOH278	2.51	15	0.412	1.34	-13	0.262	3.85	2	0.574	Both	2
	HOH311	0.97	70	0.296	0.00	-390	-0.658	0.97	-321	-0.486	Protein A	2
	HOH323	1.29	427	0.577	1.14	-153	-0.092	2.43	274	0.644	Protein A	13
	HOH330	0.00	-394	-0.668	0.00	-233	-0.242	0.00	-627	-1.348	Neither	3
	HOH360	0.83	-251	-0.315	0.00	-91	-0.040	0.83	-342	-0.539	Neither	45
	HOH369	2.11	126	0.482	0.00	-54	-0.039	2.11	72	0.417	Protein A	15
	HOH380	0.91	120	0.316	0.89	-49	0.193	1.80	72	0.388	Protein A	36
	HOH382	2.33	361	0.688	0.00	-40	-0.039	2.33	321	0.663	Protein A	9
	HOH418	1.02	-34	0.227	0.00	-89	-0.040	1.02	-123	-0.025	Neither	16
	HOH426	0.73	22	0.218	0.00	-175	-0.108	0.73	-154	-0.092	Neither	51
	HOH442	2.80	171	0.628	1.18	401	0.505	3.98	573	0.932	Both	2
2E2D	HOH3	4.48	170	0.819	1.37	-14	0.262	5.85	156	0.866	Both	55
	HOH4	1.97	-40	0.248	0.00	-145	-0.057	1.97	-184	-0.162	Neither	17
	HOH11	1.90	23	0.332	0.00	-172	-0.101	1.90	-148	-0.082	Protein A	28
	HOH21	0.90	8	0.239	0.00	-517	-1.015	0.90	-509	-0.995	Neither	9
	HOH35	2.31	128	0.509	1.18	-363	-0.593	3.49	-235	-0.278	Protein A	1

	HOH37	1.10	-2	0.265	0.96	-709	-1.613	2.06	-711	-1.619	Protein A	9
	HOH38	1.21	163	0.395	0.97	-566	-1.164	2.18	-403	-0.700	Protein A	9
	HOH50	2.31	480	0.753	1.27	19	0.295	3.58	499	0.874	Both	2
	HOH51	2.46	39	0.430	0.00	-82	-0.040	2.46	-43	0.338	Protein A	17
	HOH64	0.00	-195	-0.149	0.00	-271	-0.341	0.00	-466	-0.869	Neither	20
	HOH73	2.15	146	0.511	0.00	-227	-0.228	2.15	-82	0.232	Protein A	0
	HOH77	0.00	-252	-0.291	0.73	3	0.205	0.73	-250	-0.312	Neither	3
	HOH106	1.11	588	0.516	0.00	-252	-0.290	1.11	337	0.434	Protein A	23
	HOH113	0.98	50	0.284	0.94	-249	-0.312	1.92	-199	-0.196	Protein A	33
	HOH126	2.34	-501	-0.973	1.02	-87	0.185	3.36	-588	-1.228	Neither	39
	HOH154	1.09	22	0.281	3.58	-146	-0.074	4.66	-124	-0.020	Protein A	8
	HOH155	2.04	-62	0.232	1.02	14	0.263	3.06	-48	0.413	Protein B	17
	HOH156	2.21	-62	0.269	1.30	-82	0.183	3.51	-144	-0.069	Protein A	28
	HOH158	1.27	-167	-0.122	2.35	-94	0.257	3.62	-261	-0.339	Protein B	28
	HOH178	0.87	-93	0.153	0.98	12	0.255	1.85	-81	0.188	Protein B	12
	HOH181	0.84	26	0.241	1.13	247	0.391	1.96	273	0.600	Protein B	41
	HOH182	2.33	21	0.392	1.37	-358	-0.580	3.70	-336	-0.525	Protein A	0
	HOH201	0.00	-194	-0.147	2.24	-88	0.243	2.24	-282	-0.390	Neither	5
	HOH215	0.78	-99	0.131	0.00	-371	-0.606	0.78	-470	-0.885	Neither	23
	HOH235	1.11	-124	-0.026	1.18	202	0.385	2.30	78	0.449	Protein B	40
	HOH238	0.00	-452	-0.829	0.96	-183	-0.158	0.96	-635	-1.374	Neither	7
	HOH240	1.05	15	0.270	2.20	123	0.489	3.25	138	0.635	Both	20
	HOH253	0.00	-347	-0.541	1.90	255	0.577	1.90	-91	0.174	Protein B	15
	HOH286	0.00	-191	-0.140	0.96	69	0.293	0.96	-122	-0.022	Protein B	3
	HOH290	0.00	-202	-0.166	2.13	125	0.482	2.13	-77	0.232	Protein B	9
	HOH297	0.90	36	0.260	0.88	14	0.239	1.78	50	0.360	Protein A	28
	HOH302	1.07	55	0.302	2.33	-124	-0.027	3.40	-69	0.436	Protein A	31
	HOH303	0.96	68	0.293	0.97	-100	0.165	1.94	-33	0.258	Protein A	29
	HOH310	0.00	-164	-0.087	1.81	85	0.404	1.81	-79	0.189	Protein B	31
2EKE	HOH166	1.25	-251	-0.316	0.87	64	0.274	2.12	-187	-0.167	Protein B	0
	HOH177	1.16	2	0.273	1.08	378	0.444	2.24	380	0.694	Both	1
	HOH182	2.30	181	0.569	0.94	-22	0.223	3.24	159	0.666	Protein A	9
	HOH183	1.26	75	0.349	2.28	-41	0.309	3.53	34	0.556	Both	17
	HOH1099	3.86	49	0.617	1.53	374	0.622	5.39	423	0.977	Both	2
	HOH1101	1.08	16	0.275	2.39	250	0.624	3.47	267	0.766	Both	5
	HOH1103	1.19	100	0.360	1.21	-85	0.189	2.41	14	0.396	Protein A	5
	HOH1104	1.00	184	0.334	1.13	-199	-0.195	2.13	-15	0.314	Protein A	8
	HOH1105	1.02	21	0.269	2.49	387	0.718	3.51	408	0.831	Both	54
	HOH1109	0.00	-136	-0.047	3.51	438	0.843	3.51	302	0.785	Protein B	15
	HOH1112	2.17	152	0.520	1.31	152	0.417	3.48	303	0.782	Both	20
	HOH1114	0.00	28	-0.038	4.00	360	0.860	4.00	387	0.871	Protein B	15
	HOH1115	0.00	-28	-0.039	0.98	49	0.282	0.98	21	0.262	Protein B	30
	HOH1118	1.04	61	0.301	2.08	115	0.463	3.11	176	0.674	Both	2
	HOH1123	0.90	-15	0.221	1.16	-202	-0.201	2.06	-217	-0.235	Neither	53
	HOH1127	0.00	-184	-0.125	0.84	16	0.234	0.84	-168	-0.124	Neither	16
	HOH1128	0.88	-39	0.199	1.21	-172	-0.133	2.09	-211	-0.221	Neither	20
	HOH1131	0.82	-21	0.203	1.19	247	0.418	2.01	226	0.564	Protein B	8
	HOH1138	0.86	24	0.243	1.03	33	0.278	1.88	57	0.374	Protein B	5
	HOH1140	1.22	103	0.367	2.14	-122	-0.023	3.36	-19	0.481	Protein A	4
	HOH1141	0.00	-13	-0.039	2.05	410	0.708	2.05	397	0.700	Protein B	0
2ES4	HOH3	2.36	41	0.418	0.00	-37	-0.039	2.36	4	0.377	Protein A	0
	HOH8	1.34	-238	-0.285	2.20	-168	-0.125	3.53	-406	-0.708	Neither	29
	HOH11	1.33	52	0.333	0.98	-129	-0.038	2.31	-78	0.271	Protein A	21
	HOH18	0.79	-196	-0.188	0.00	-99	-0.040	0.79	-296	-0.424	Neither	7
	HOH19	2.50	-386	-0.654	1.01	-63	0.202	3.51	-449	-0.824	Neither	9
	HOH20	1.13	-317	-0.476	2.09	177	0.536	3.21	-140	-0.060	Protein B	25
	HOH29	1.25	-125	-0.029	1.17	-277	-0.377	2.42	-401	-0.695	Neither	16
	HOH39	0.99	-53	0.207	0.00	-646	-1.408	0.99	-699	-1.578	Neither	10
	HOH42	2.65	-36	0.373	1.27	85	0.359	3.92	49	0.618	Both	23
	HOH43	1.35	-422	-0.751	2.48	-233	-0.274	3.83	-655	-1.439	Neither	5

	HOH48	3.85	29	0.598	1.20	269	0.439	5.05	298	0.917	Both	12
	HOH53	1.23	-154	-0.093	2.24	-220	-0.243	3.47	-374	-0.622	Neither	24
	HOH63	2.05	-9	0.306	0.00	-31	-0.039	2.05	-40	0.264	Protein A	31
	HOH67	0.75	-134	-0.049	0.00	-287	-0.381	0.75	-420	-0.747	Neither	36
	HOH70	1.22	-58	0.217	1.16	-125	-0.028	2.38	-182	-0.156	Neither	12
	HOH73	0.89	-74	0.173	0.00	-30	-0.039	0.89	-104	0.148	Neither	26
	HOH75	2.47	-34	0.350	1.05	-161	-0.109	3.52	-195	-0.185	Protein A	0
	HOH85	3.39	414	0.821	0.00	-56	-0.039	3.39	358	0.796	Protein A	0
	HOH100	2.44	134	0.534	1.18	-295	-0.422	3.63	-161	-0.107	Protein A	1
	HOH106	1.22	281	0.455	0.84	46	0.256	2.06	327	0.649	Both	0
	HOH113	2.36	103	0.485	1.07	107	0.338	3.43	211	0.751	Both	1
	HOH114	4.00	372	0.865	1.29	-101	0.162	5.29	271	0.926	Protein A	11
	HOH121	1.14	-353	-0.569	0.92	-13	0.226	2.06	-366	-0.603	Neither	8
	HOH128	1.06	-355	-0.574	0.00	-575	-1.188	1.06	-930	-2.388	Neither	12
	HOH129	2.45	186	0.594	2.37	-64	0.298	4.82	122	0.750	Both	38
	HOH158	0.76	43	0.239	0.00	-363	-0.583	0.76	-320	-0.484	Neither	9
	HOH161	2.71	-92	0.318	2.47	-120	-0.017	5.18	-212	-0.224	Protein A	10
	HOH164	1.38	26	0.307	3.76	416	0.859	5.15	442	0.972	Both	45
	HOH172	2.06	249	0.587	0.00	-267	-0.331	2.06	-18	0.294	Protein A	4
	HOH173	0.96	10	0.251	0.92	-98	0.158	1.89	-88	0.179	Protein A	28
	HOH185	2.07	-44	0.263	1.15	43	0.305	3.22	-1	0.481	Both	4
	HOH186	0.93	-47	0.200	1.06	-140	-0.061	1.99	-187	-0.168	Neither	19
	HOH190	2.33	14	0.383	2.38	-288	-0.406	4.71	-274	-0.371	Protein A	30
	HOH193	0.00	-242	-0.264	2.07	547	0.783	2.07	306	0.633	Protein B	1
	HOH197	1.52	308	0.569	3.77	-176	-0.141	5.29	132	0.794	Protein A	42
	HOH204	1.13	-60	0.219	3.94	643	0.946	5.06	583	1.013	Protein B	2
	HOH207	1.04	-341	-0.536	2.48	-159	-0.105	3.52	-500	-0.968	Neither	8
	HOH214	0.92	5	0.240	1.11	52	0.307	2.03	57	0.386	Protein B	17
	HOH219	0.75	-51	0.166	0.93	51	0.276	1.68	0	0.290	Protein B	11
	HOH224	0.89	85	0.293	1.13	-14	0.258	2.02	71	0.403	Both	18
	HOH230	2.22	159	0.535	1.33	282	0.498	3.55	441	0.848	Both	13
	HOH237	0.00	-193	-0.144	0.79	184	0.287	0.79	-9	0.206	Protein B	21
	HOH238	0.00	-44	-0.039	0.97	57	0.287	0.97	13	0.254	Protein B	16
	HOH241	0.71	-3	0.197	0.00	-43	-0.039	0.71	-46	0.163	Neither	7
	HOH259	0.00	125	-0.038	1.09	95	0.334	1.09	221	0.361	Protein B	0
	HOH261	1.37	-117	-0.014	2.59	295	0.675	3.96	178	0.787	Protein B	23
	HOH282	0.00	78	-0.038	2.16	368	0.682	2.16	446	0.730	Protein B	0
	HOH286	2.75	363	0.730	2.79	510	0.803	5.53	874	1.081	Both	43
	HOH289	0.00	-107	-0.040	0.00	-274	-0.348	0.00	-381	-0.632	Neither	15
	HOH290	1.05	-51	0.218	2.38	398	0.714	3.42	347	0.794	Protein B	31
	HOH298	0.00	-71	-0.040	0.75	-322	-0.488	0.75	-392	-0.672	Neither	0
	HOH317	1.04	13	0.267	2.66	-138	-0.057	3.70	-125	-0.024	Protein A	43
	HOH330	2.19	-101	0.214	1.25	135	0.396	3.44	34	0.544	Protein B	0
	HOH339	0.90	-108	0.145	1.10	503	0.486	1.99	396	0.700	Protein B	0
	HOH586	1.05	53	0.298	0.79	-101	0.133	1.84	-47	0.235	Protein A	3
2F2L	HOH3	1.11	-47	0.231	1.02	-213	-0.226	2.13	-259	-0.335	Neither	5
	HOH6	1.01	-87	0.182	0.00	-370	-0.603	1.01	-457	-0.847	Neither	4
	HOH51	0.00	-309	-0.439	0.75	-43	0.173	0.75	-351	-0.564	Neither	28
	HOH75	0.94	-11	0.231	0.80	111	0.293	1.74	100	0.417	Protein B	1
	HOH93	0.00	-322	-0.476	0.97	-460	-0.855	0.97	-782	-1.859	Neither	24
	HOH99	1.00	-38	0.220	2.18	-273	-0.369	3.18	-312	-0.463	Neither	0
	HOH104	0.84	-136	-0.053	1.14	325	0.446	1.98	190	0.534	Protein B	36
	HOH135	0.00	-313	-0.452	3.46	-90	0.422	3.46	-404	-0.702	Protein B	0
	HOH193	0.99	165	0.336	1.24	162	0.403	2.23	327	0.659	Both	6
2F93	HOH318	2.51	-108	0.269	1.23	274	0.455	3.74	166	0.744	Both	0
	HOH319	1.27	-58	0.212	1.23	-217	-0.236	2.50	-275	-0.374	Neither	24
	HOH124	0.00	-448	-0.818	3.82	10	0.577	3.82	-438	-0.794	Protein B	13
2F95	HOH17	2.51	-183	-0.157	1.15	236	0.395	3.66	54	0.591	Protein B	21
	HOH18	1.29	-77	0.191	1.22	-214	-0.229	2.51	-291	-0.412	Neither	12
	HOH33	0.00	-459	-0.848	3.79	-27	0.538	3.79	-486	-0.928	Protein B	0

2FD6	HOH10	2.49	356	0.699	1.07	-169	-0.126	3.56	187	0.749	Protein A	17
	HOH13	1.12	139	0.363	2.05	-514	-1.008	3.17	-375	-0.625	Protein A	1
	HOH15	2.00	-288	-0.405	0.00	-88	-0.040	2.00	-376	-0.628	Neither	22
	HOH21	1.10	61	0.310	1.03	-192	-0.179	2.12	-132	-0.045	Protein A	17
	HOH59	0.94	-113	-0.003	0.88	-53	0.187	1.81	-166	-0.120	Neither	39
	HOH159	1.02	532	0.482	2.53	-43	0.348	3.55	489	0.866	Both	33
	HOH207	1.06	145	0.349	0.00	-53	-0.039	1.06	92	0.326	Protein A	6
	HOH265	2.60	116	0.531	2.51	118	0.521	5.11	235	0.914	Both	7
	HOH273	1.19	1	0.273	3.90	-87	0.493	5.10	-86	0.566	Both	13
	HOH293	1.09	-8	0.259	1.33	-63	0.205	2.42	-70	0.299	Protein A	1
	HOH300	1.12	-78	0.204	2.32	-543	-1.092	3.44	-621	-1.330	Neither	33
	HOH314	0.91	38	0.263	0.97	-186	-0.165	1.88	-148	-0.080	Protein A	7
	HOH317	1.06	-35	0.232	1.24	-122	-0.024	2.30	-158	-0.101	Neither	24
2FDB	HOH3	4.51	87	0.690	1.44	105	0.399	5.95	191	0.941	Both	3
	HOH7	2.54	160	0.579	2.58	-175	-0.139	5.12	-15	0.635	Protein A	50
	HOH8	2.25	-225	-0.254	1.17	-170	-0.129	3.42	-395	-0.679	Neither	14
	HOH10	1.37	-151	-0.088	2.70	-186	-0.164	4.08	-337	-0.527	Neither	1
	HOH14	1.09	-114	-0.005	0.88	-383	-0.647	1.97	-498	-0.962	Neither	22
	HOH18	1.04	-216	-0.232	0.94	-237	-0.281	1.99	-452	-0.833	Neither	1
	HOH19	2.02	181	0.530	0.00	-187	-0.131	2.02	-6	0.302	Protein A	2
	HOH21	2.94	118	0.571	2.37	-146	-0.076	5.31	-28	0.631	Protein A	0
	HOH23	2.77	-80	0.341	1.35	37	0.318	4.12	-43	0.550	Both	1
	HOH31	1.42	85	0.376	2.57	179	0.605	3.99	264	0.828	Both	8
	HOH40	1.27	46	0.322	4.12	-166	-0.119	5.39	-120	-0.011	Protein A	30
	HOH53	1.10	-12	0.258	1.19	218	0.396	2.29	206	0.586	Both	11
	HOH57	1.10	-186	-0.165	2.28	375	0.693	3.37	189	0.726	Protein B	23
	HOH60	2.30	-107	0.230	1.31	96	0.376	3.61	-11	0.524	Protein B	52
	HOH62	0.00	-2	-0.039	1.08	26	0.284	1.08	25	0.282	Protein B	4
	HOH71	0.00	-89	-0.040	2.26	449	0.735	2.26	360	0.682	Protein B	19
	HOH80	2.66	286	0.677	1.31	-160	-0.107	3.96	126	0.700	Protein A	6
	HOH97	2.21	246	0.600	0.00	-14	-0.039	2.21	231	0.591	Protein A	3
	HOH100	1.10	46	0.301	1.02	80	0.311	2.12	125	0.481	Both	41
2FM8	HOH3	2.74	558	0.816	1.28	80	0.356	4.02	638	0.952	Both	31
	HOH5	2.29	104	0.477	2.41	-112	0.000	4.69	-7	0.620	Protein A	24
	HOH8	2.26	-150	-0.085	1.09	-21	0.248	3.34	-171	-0.131	Neither	21
	HOH19	2.46	559	0.794	0.95	-168	-0.125	3.40	391	0.812	Protein A	45
	HOH24	3.48	-439	-0.797	1.11	-46	0.231	4.59	-485	-0.926	Neither	38
	HOH31	2.46	-195	-0.185	1.31	337	0.530	3.76	142	0.707	Protein B	0
	HOH33	1.27	38	0.314	1.05	-15	0.247	2.32	23	0.392	Protein A	7
	HOH43	1.09	48	0.300	0.87	-31	0.204	1.96	17	0.325	Protein A	0
	HOH52	0.99	370	0.423	0.83	-44	0.186	1.82	327	0.627	Protein A	9
	HOH64	0.92	-353	-0.568	0.87	-226	-0.256	1.79	-579	-1.200	Neither	14
	HOH108	0.81	-180	-0.151	0.86	-4	0.222	1.67	-184	-0.161	Neither	15
	HOH124	1.24	-190	-0.174	0.95	57	0.283	2.19	-133	-0.049	Protein B	35
	HOH130	2.26	169	0.552	1.16	-220	-0.243	3.42	-51	0.457	Protein A	2
	HOH136	1.21	75	0.342	1.16	134	0.371	2.37	209	0.600	Both	0
	HOH146	0.96	21	0.258	0.95	-138	-0.058	1.91	-117	-0.013	Protein A	0
	HOH156	2.53	184	0.604	1.25	-177	-0.144	3.78	7	0.567	Protein A	17
	HOH159	2.74	589	0.826	1.23	-99	0.172	3.97	490	0.906	Protein A	1
	HOH162	2.30	-196	-0.187	0.00	-329	-0.494	2.30	-525	-1.042	Neither	45
	HOH164	2.60	184	0.614	1.30	91	0.369	3.90	275	0.821	Both	0
	HOH166	2.51	320	0.680	0.00	-28	-0.039	2.51	293	0.664	Protein A	8
	HOH168	1.05	75	0.313	0.88	-267	-0.354	1.93	-192	-0.180	Protein A	17
	HOH169	1.17	-2	0.270	2.16	103	0.459	3.33	102	0.592	Both	3
	HOH181	4.24	148	0.760	1.40	249	0.492	5.64	397	0.979	Both	20
	HOH182	2.12	358	0.674	0.00	-588	-1.228	2.12	-230	-0.267	Protein A	17
	HOH187	1.34	58	0.340	2.46	-266	-0.351	3.79	-208	-0.213	Protein A	20
	HOH195	3.52	23	0.544	0.00	-233	-0.243	3.52	-210	-0.220	Protein A	0
	HOH226	1.04	95	0.325	0.96	12	0.252	2.00	107	0.443	Both	0
	HOH293	0.00	-261	-0.313	2.32	565	0.789	2.32	304	0.651	Protein B	24

	HOH323	2.51	-85	0.297	2.55	589	0.811	5.06	504	0.989	Both	36
	HOH327	0.00	-63	-0.039	1.01	-299	-0.433	1.01	-362	-0.593	Neither	34
	HOH341	2.50	-22	0.370	2.79	188	0.645	5.29	167	0.865	Both	16
	HOH351	1.31	378	0.559	2.58	-466	-0.873	3.90	-89	0.490	Protein A	21
	HOH362	0.00	-12	-0.039	0.95	140	0.328	0.95	127	0.326	Protein B	32
	HOH364	0.00	-221	-0.210	1.13	43	0.304	1.13	-177	-0.145	Protein B	17
	HOH389	1.14	-48	0.229	1.43	470	0.656	2.57	421	0.743	Protein B	23
	HOH421	0.97	0	0.245	2.07	-352	-0.566	3.05	-352	-0.567	Neither	13
	HOH426	0.00	-227	-0.227	2.27	92	0.461	2.27	-135	-0.051	Protein B	47
	HOH428	0.00	-270	-0.338	2.00	190	0.536	2.00	-80	0.194	Protein B	7
	HOH450	0.86	-82	0.160	0.86	-10	0.219	1.72	-92	0.167	Neither	18
	HOH455	0.91	-104	0.151	0.99	-146	-0.076	1.90	-251	-0.315	Neither	2
	HOH467	0.00	-300	-0.418	0.92	79	0.294	0.92	-222	-0.246	Protein B	41
	HOH469	1.07	-240	-0.290	1.16	-93	0.186	2.23	-333	-0.518	Neither	8
	HOH474	1.14	-103	0.180	3.90	-16	0.560	5.04	-119	-0.007	Protein B	36
	HOH492	0.89	-87	0.161	1.11	-184	-0.160	2.00	-271	-0.364	Neither	0
	HOH493	1.04	93	0.324	1.07	-68	0.209	2.11	25	0.361	Protein A	30
	HOH515	1.10	-16	0.255	2.30	84	0.456	3.40	68	0.570	Both	55
	HOH518	1.16	73	0.332	2.59	150	0.573	3.75	223	0.796	Both	19
	HOH519	0.00	-274	-0.349	0.84	29	0.244	0.84	-245	-0.301	Neither	49
	HOH562	0.00	-161	-0.082	0.75	164	0.286	0.75	3	0.208	Protein B	8
	HOH570	0.86	-31	0.202	1.14	426	0.489	2.00	395	0.700	Protein B	18
	HOH581	0.92	-183	-0.159	1.20	-90	0.185	2.12	-273	-0.369	Neither	46
	HOH588	0.90	-61	0.185	0.93	-130	-0.039	1.83	-190	-0.175	Neither	4
	HOH608	0.00	-17	-0.039	0.78	-24	0.193	0.78	-41	0.180	Neither	1
2FU5	HOH4345	1.11	402	0.465	0.00	-261	-0.314	1.11	141	0.360	Protein A	4
	HOH4350	1.86	119	0.448	0.00	124	-0.038	1.86	243	0.560	Protein A	28
	HOH4383	0.00	-251	-0.288	0.79	114	0.291	0.79	-137	-0.057	Protein B	10
	HOH4392	1.23	108	0.374	2.14	64	0.412	3.37	172	0.703	Both	26
	HOH184	1.43	127	0.420	4.33	93	0.684	5.76	220	0.951	Both	53
	HOH190	1.11	76	0.324	1.15	4	0.274	2.26	81	0.447	Both	23
	HOH193	0.00	-117	-0.032	3.48	-78	0.438	3.48	-195	-0.185	Protein B	47
	HOH194	1.19	137	0.383	1.20	91	0.354	2.39	229	0.613	Both	38
	HOH198	0.75	23	0.222	0.00	-187	-0.132	0.75	-164	-0.116	Neither	31
	HOH204	0.97	31	0.267	1.19	181	0.389	2.16	211	0.571	Both	34
	HOH207	0.00	-40	-0.039	0.92	38	0.265	0.92	-2	0.235	Protein B	36
	HOH213	0.00	-204	-0.170	0.00	-180	-0.118	0.00	-384	-0.641	Neither	44
2G2U	HOH297	5.10	-53	0.598	0.00	-92	-0.040	5.10	-145	-0.070	Protein A	2
	HOH308	3.96	-28	0.553	1.24	18	0.292	5.20	-10	0.642	Both	5
	HOH316	2.54	319	0.682	2.55	43	0.446	5.08	362	0.939	Both	13
	HOH326	2.53	140	0.552	1.30	-20	0.254	3.83	120	0.680	Both	3
	HOH328	2.14	-45	0.277	1.04	-77	0.195	3.17	-122	-0.019	Protein A	5
	HOH341	1.09	-646	-1.408	0.79	17	0.226	1.88	-629	-1.355	Neither	11
	HOH380	2.62	450	0.762	1.23	73	0.343	3.85	523	0.906	Both	0
	HOH389	2.17	47	0.396	1.05	-8	0.251	3.22	38	0.520	Both	7
	HOH393	1.12	-203	-0.204	0.91	-199	-0.194	2.03	-402	-0.698	Neither	0
	HOH412	0.83	-316	-0.474	0.00	-8	-0.039	0.83	-324	-0.495	Neither	0
	HOH413	2.26	-114	-0.004	0.97	-62	0.196	3.23	-176	-0.141	Neither	0
	HOH418	1.14	-171	-0.132	1.13	69	0.323	2.27	-102	0.229	Protein B	27
	HOH419	1.21	129	0.384	1.10	200	0.356	2.32	329	0.667	Both	1
	HOH427	1.33	261	0.481	2.25	-5	0.348	3.58	256	0.777	Both	25
	HOH459	1.07	433	0.464	0.91	-263	-0.344	1.99	171	0.518	Protein A	13
	HOH476	1.57	407	0.651	4.00	121	0.695	5.57	527	1.021	Both	36
	HOH483	1.23	-53	0.221	0.91	-3	0.232	2.14	-56	0.263	Neither	15
	HOH494	0.85	-177	-0.144	0.76	42	0.239	1.61	-135	-0.052	Neither	45
	HOH500	0.96	-52	0.202	0.94	40	0.270	1.90	-11	0.286	Protein B	4
	HOH508	1.05	-74	0.199	0.87	-96	0.151	1.92	-170	-0.129	Neither	17
	HOH513	0.79	80	0.271	0.00	-26	-0.039	0.79	54	0.252	Protein A	0
	HOH175	1.95	-646	-1.410	1.29	192	0.420	3.23	-455	-0.840	Protein B	0
	HOH181	3.99	-303	-0.442	1.25	389	0.539	5.24	86	0.727	Protein B	0

	HOH194	0.00	-201	-0.164	2.05	61	0.393	2.05	-141	-0.065	Protein B	7
	HOH201	1.15	-67	0.212	2.43	-8	0.374	3.58	-75	0.456	Protein B	8
	HOH204	0.00	-39	-0.039	1.87	-92	0.173	1.87	-131	-0.044	Neither	5
	HOH211	0.00	7	-0.039	2.08	-120	-0.019	2.08	-112	-0.003	Neither	8
	HOH218	1.10	-349	-0.559	4.33	931	1.011	5.44	582	1.031	Protein B	16
	HOH219	0.00	-70	-0.039	2.39	299	0.655	2.39	229	0.613	Protein B	1
	HOH220	1.16	-225	-0.253	3.58	154	0.701	4.75	-71	0.563	Protein B	10
	HOH222	1.18	92	0.350	2.68	11	0.429	3.85	103	0.662	Both	32
	HOH232	2.40	66	0.451	1.39	26	0.308	3.78	93	0.641	Both	5
	HOH237	0.00	-28	-0.039	1.77	-29	0.258	1.77	-56	0.220	Protein B	6
	HOH243	1.17	-62	0.215	2.64	324	0.697	3.81	262	0.809	Protein B	0
	HOH244	1.18	57	0.322	2.39	308	0.661	3.57	366	0.819	Both	26
	HOH245	0.79	-4	0.209	0.82	-93	0.145	1.61	-97	0.155	Neither	7
	HOH254	0.93	8	0.244	1.03	277	0.383	1.96	285	0.610	Protein B	7
	HOH260	0.00	-205	-0.174	1.94	-176	-0.144	1.94	-382	-0.643	Neither	19
	HOH263	0.00	-165	-0.088	1.04	148	0.346	1.04	-17	0.244	Protein B	53
2GC7	HOH85	3.59	-85	0.447	0.00	-163	-0.084	3.59	-248	-0.308	Protein A	0
	HOH105	2.35	161	0.554	0.00	-208	-0.179	2.35	-47	0.315	Protein A	0
	HOH118	1.35	-133	-0.047	2.62	42	0.454	3.97	-91	0.494	Protein B	0
	HOH120	4.09	-45	0.547	1.23	-55	0.219	5.32	-100	0.563	Protein A	1
	HOH182	2.15	-130	-0.042	1.13	-281	-0.388	3.28	-411	-0.722	Neither	1
	HOH204	1.41	-160	-0.106	2.70	-58	0.357	4.11	-218	-0.236	Protein B	10
	HOH216	1.24	-145	-0.075	1.97	52	0.372	3.20	-93	0.382	Protein B	9
	HOH218	1.94	-149	-0.084	0.00	-387	-0.650	1.94	-536	-1.074	Neither	0
	HOH237	0.87	-173	-0.135	1.07	-464	-0.866	1.94	-636	-1.379	Neither	24
	HOH250	2.06	74	0.411	1.12	45	0.303	3.18	119	0.598	Both	26
	HOH253	0.00	-245	-0.271	0.95	-161	-0.108	0.95	-405	-0.705	Neither	61
	HOH256	0.00	-76	-0.040	0.75	24	0.225	0.75	-52	0.166	Neither	6
	HOH258	1.89	-12	0.285	1.48	254	0.514	3.37	241	0.747	Both	0
	HOH269	3.82	183	0.781	1.36	379	0.579	5.18	563	1.014	Both	17
	HOH284	1.03	-346	-0.550	0.00	-46	-0.039	1.03	-392	-0.670	Neither	46
	HOH300	0.92	90	0.302	0.00	-194	-0.148	0.92	-104	0.153	Protein A	5
	HOH413	2.54	-225	-0.254	1.06	-30	0.236	3.60	-255	-0.326	Neither	0
2GH0	HOH9	2.57	342	0.700	2.62	126	0.545	5.20	468	0.984	Both	6
	HOH18	1.09	-70	0.210	1.22	-64	0.210	2.31	-133	-0.048	Neither	23
	HOH27	0.97	-84	0.178	1.17	-190	-0.174	2.13	-274	-0.371	Neither	20
	HOH72	2.60	-369	-0.610	0.99	-115	-0.008	3.59	-484	-0.924	Neither	2
	HOH87	2.53	354	0.703	1.25	-133	-0.048	3.78	221	0.800	Protein A	0
	HOH90	2.62	-262	-0.341	2.38	-77	0.285	5.00	-338	-0.531	Protein B	0
	HOH144	2.62	515	0.790	1.14	7	0.275	3.76	521	0.897	Both	4
	HOH150	0.94	-59	0.194	0.86	44	0.258	1.81	-15	0.278	Protein B	0
2GOO	HOH4	2.25	14	0.370	1.28	-117	-0.013	3.52	-103	0.418	Protein A	0
	HOH6	2.51	-568	-1.167	1.17	1	0.272	3.68	-567	-1.165	Protein B	10
	HOH10	1.01	-371	-0.616	2.16	298	0.633	3.18	-73	0.400	Protein B	28
	HOH15	1.19	35	0.305	1.05	112	0.337	2.24	147	0.525	Both	12
	HOH24	0.88	-98	0.150	0.00	-240	-0.261	0.88	-338	-0.530	Neither	33
	HOH28	1.21	-278	-0.380	1.19	205	0.389	2.40	-73	0.293	Protein B	17
	HOH29	2.32	482	0.754	0.00	-94	-0.040	2.32	387	0.703	Protein A	27
	HOH34	1.17	-92	0.186	1.13	-87	0.195	2.30	-180	-0.151	Neither	0
	HOH37	1.14	-143	-0.069	0.96	50	0.280	2.10	-93	0.204	Protein B	35
	HOH38	1.06	564	0.498	2.03	-167	-0.123	3.09	397	0.783	Protein A	0
	HOH40	1.14	-6	0.265	1.13	343	0.449	2.27	338	0.668	Both	14
	HOH50	1.13	-118	-0.013	1.04	-111	0.002	2.17	-229	-0.263	Neither	5
	HOH53	2.38	-172	-0.134	1.02	-243	-0.296	3.40	-415	-0.732	Neither	21
	HOH72	1.03	-213	-0.227	0.85	119	0.305	1.88	-94	0.170	Protein B	0
	HOH76	1.07	-354	-0.570	0.90	4	0.236	1.97	-349	-0.558	Neither	22
	HOH82	0.99	-291	-0.413	0.97	-184	-0.159	1.95	-475	-0.897	Neither	28
	HOH84	1.11	-156	-0.097	1.09	188	0.354	2.20	32	0.384	Protein B	1
	HOH86	1.87	145	0.481	0.00	-162	-0.084	1.87	-17	0.278	Protein A	36
	HOH91	1.00	-3	0.248	1.19	635	0.587	2.19	631	0.812	Protein B	25

	HOH93	1.13	60	0.316	1.14	-289	-0.408	2.27	-229	-0.265	Protein A	20
	HOH94	0.00	-241	-0.263	5.26	85	0.727	5.26	-157	-0.096	Protein B	17
	HOH107	0.00	-9	-0.039	2.34	12	0.382	2.34	3	0.372	Protein B	29
	HOH111	1.16	462	0.515	1.18	-39	0.237	2.33	423	0.725	Protein A	10
	HOH115	0.88	95	0.298	0.99	-156	-0.097	1.87	-61	0.217	Protein A	4
	HOH118	0.91	-54	0.192	0.95	-77	0.179	1.86	-131	-0.045	Neither	19
	HOH120	1.01	-53	0.210	2.26	302	0.643	3.26	248	0.736	Protein B	6
	HOH128	1.15	-32	0.243	2.26	132	0.508	3.41	100	0.600	Protein B	0
	HOH131	1.08	-791	-1.888	1.15	127	0.366	2.23	-664	-1.467	Protein B	0
	HOH136	1.19	97	0.357	1.19	130	0.379	2.39	227	0.611	Both	24
	HOH139	1.10	-49	0.228	1.14	91	0.341	2.24	41	0.400	Protein B	6
	HOH140	0.00	-208	-0.180	1.94	406	0.703	1.94	198	0.536	Protein B	15
	HOH142	0.00	-164	-0.086	2.89	65	0.509	2.89	-98	0.336	Protein B	5
	HOH144	0.00	-216	-0.199	1.04	185	0.344	1.04	-31	0.233	Protein B	21
	HOH147	0.00	-291	-0.392	2.92	417	0.775	2.92	127	0.580	Protein B	58
	HOH149	0.00	-215	-0.196	0.75	-31	0.181	0.75	-246	-0.304	Neither	11
	HOH155	0.00	-472	-0.885	0.70	161	0.276	0.70	-311	-0.463	Protein B	0
2HQS	HOH1451	4.40	131	0.741	1.48	-87	0.169	5.88	45	0.714	Protein A	0
	HOH1572	1.04	421	0.453	0.00	-111	-0.029	1.04	310	0.404	Protein A	12
	HOH1640	1.09	70	0.316	0.97	-76	0.185	2.05	-5	0.311	Protein A	33
	HOH1746	2.23	89	0.453	1.11	-7	0.262	3.34	82	0.575	Both	23
	HOH1786	2.75	7	0.435	1.18	34	0.303	3.93	42	0.613	Both	11
	HOH1825	1.17	97	0.352	1.11	116	0.349	2.27	214	0.588	Both	17
	HOH2135	1.37	-546	-1.104	2.64	295	0.680	4.01	-251	-0.316	Protein B	0
	HOH2137	2.22	350	0.673	1.21	-351	-0.564	3.43	-2	0.507	Protein A	0
	HOH2139	5.04	202	0.911	0.00	-272	-0.341	5.04	-70	0.580	Protein A	0
	HOH2163	4.20	-106	0.495	1.47	294	0.547	5.67	187	0.923	Both	0
	HOH2170	1.00	-140	-0.061	0.00	-203	-0.168	1.00	-343	-0.541	Neither	20
	HOH2197	2.06	-46	0.259	0.00	-112	-0.030	2.06	-157	-0.102	Protein A	5
	HOH2210	1.08	131	0.351	0.94	-159	-0.104	2.02	-28	0.273	Protein A	15
	HOH2272	1.89	-96	0.167	0.00	-64	-0.039	1.89	-160	-0.107	Neither	8
	HOH2294	1.01	83	0.312	0.00	3	-0.039	1.01	86	0.314	Protein A	8
	HOH2307	2.52	178	0.597	1.35	2	0.279	3.87	179	0.782	Both	9
	HOH2325	1.06	43	0.292	0.88	-11	0.221	1.94	33	0.345	Protein A	22
	HOH2342	2.40	218	0.609	0.97	-41	0.214	3.38	177	0.710	Protein A	19
	HOH2362	2.23	95	0.458	1.17	-83	0.196	3.39	12	0.516	Protein A	3
	HOH2373	1.20	437	0.534	0.92	-129	-0.039	2.12	308	0.637	Protein A	2
	HOH2397	2.19	48	0.401	1.00	156	0.338	3.19	204	0.716	Both	13
	HOH2427	1.85	156	0.490	0.00	-336	-0.511	1.85	-179	-0.151	Protein A	6
	HOH2439	0.82	45	0.252	0.00	-316	-0.460	0.82	-272	-0.365	Protein A	31
	HOH2482	1.09	85	0.326	0.00	-492	-0.943	1.09	-407	-0.710	Protein A	12
	HOH2497	0.80	40	0.246	0.00	-61	-0.039	0.80	-21	0.199	Neither	24
	HOH2663	0.91	45	0.267	0.00	-305	-0.429	0.91	-260	-0.337	Protein A	29
	HOH2715	0.97	-142	-0.066	2.46	129	0.529	3.42	-13	0.496	Protein B	0
	HOH2716	0.00	-209	-0.181	4.03	41	0.620	4.03	-168	-0.121	Protein B	0
	HOH2717	3.64	-34	0.506	1.39	-228	-0.261	5.03	-262	-0.341	Protein A	0
	HOH2718	0.91	-119	-0.016	2.34	164	0.557	3.24	45	0.530	Protein B	0
	HOH2724	1.21	-259	-0.334	4.14	-12	0.581	5.34	-270	-0.362	Protein B	0
	HOH2725	1.17	-66	0.211	2.25	-102	0.226	3.42	-168	-0.123	Neither	0
	HOH2727	1.29	-155	-0.097	2.57	405	0.734	3.86	249	0.813	Protein B	2
	HOH2729	0.90	-171	-0.130	1.11	52	0.308	2.02	-118	-0.016	Protein B	18
	HOH2731	0.00	-22	-0.039	0.94	-147	-0.079	0.94	-170	-0.128	Neither	30
	HOH2732	1.14	-123	-0.024	1.16	301	0.442	2.31	179	0.567	Protein B	22
	HOH2733	0.92	-127	-0.033	0.95	-78	0.180	1.88	-204	-0.207	Neither	2
	HOH2737	1.09	5	0.269	1.19	-120	-0.019	2.28	-115	-0.008	Protein A	12
	HOH2738	3.55	-122	-0.019	1.42	-32	0.240	4.97	-155	-0.091	Neither	10
	HOH2743	1.02	-52	0.213	2.59	-4	0.402	3.62	-56	0.482	Protein B	4
	HOH2744	2.36	99	0.481	1.32	478	0.617	3.69	577	0.907	Both	1
	HOH2746	0.00	-30	-0.039	0.00	-46	-0.039	0.00	-76	-0.040	Neither	41
	HOH2750	1.06	-38	0.231	2.24	-89	0.242	3.30	-127	-0.031	Neither	17

	HOH2752	0.80	-203	-0.203	1.18	338	0.471	1.97	135	0.476	Protein B	20
	HOH2760	1.00	-171	-0.131	1.11	-124	-0.025	2.11	-295	-0.421	Neither	13
	HOH2765	2.24	-341	-0.537	1.36	127	0.411	3.60	-213	-0.226	Protein B	14
	HOH2769	0.94	98	0.309	2.44	135	0.536	3.38	234	0.747	Both	29
	HOH2773	0.00	-164	-0.086	0.96	5	0.247	0.96	-159	-0.105	Neither	40
	HOH2783	0.00	73	-0.038	0.93	-97	0.159	0.93	-25	0.218	Neither	45
	HOH2795	1.00	123	0.333	1.03	70	0.306	2.03	193	0.542	Both	66
	HOH2799	1.06	49	0.297	2.15	-96	0.213	3.22	-47	0.434	Protein A	28
	HOH2809	0.79	-131	-0.043	1.15	216	0.381	1.94	85	0.411	Protein B	0
	HOH2812	0.00	-172	-0.101	1.04	-64	0.206	1.04	-236	-0.280	Neither	8
2IAA	HOH547	5.19	196	0.913	0.00	-359	-0.575	5.19	-163	-0.110	Protein A	20
	HOH645	0.00	-443	-0.803	0.86	-193	-0.181	0.86	-636	-1.377	Neither	20
	HOH655	3.57	-343	-0.544	0.00	-125	-0.038	3.57	-469	-0.879	Neither	10
	HOH174	1.03	-131	-0.043	0.90	-66	0.180	1.92	-197	-0.190	Neither	49
	HOH196	1.00	-227	-0.259	0.91	-37	0.205	1.91	-264	-0.348	Neither	4
	HOH199	1.97	-169	-0.126	0.00	-256	-0.301	1.97	-425	-0.758	Neither	30
	HOH201	0.98	-86	0.178	0.00	-215	-0.198	0.98	-301	-0.438	Neither	6
	HOH367	0.00	-185	-0.128	2.15	61	0.410	2.15	-124	-0.029	Protein B	0
	HOH680	0.88	-296	-0.425	2.45	-137	-0.056	3.33	-433	-0.782	Neither	5
	HOH824	0.00	-154	-0.070	2.18	77	0.433	2.18	-76	0.245	Protein B	0
	HOH960	0.00	-406	-0.701	3.33	-31	0.464	3.33	-437	-0.793	Protein B	18
	HOH981	0.00	-334	-0.505	0.85	67	0.272	0.85	-267	-0.353	Protein B	13
	HOH510	0.83	-114	-0.006	0.87	-28	0.206	1.70	-142	-0.068	Neither	22
2J12	HOH2004	2.40	-183	-0.158	1.28	-58	0.213	3.68	-241	-0.291	Neither	0
	HOH2005	2.41	387	0.710	0.00	-280	-0.363	2.41	107	0.496	Protein A	1
	HOH2008	2.15	-92	0.218	1.20	266	0.435	3.35	174	0.702	Protein B	0
	HOH2009	3.49	-199	-0.194	0.00	149	-0.038	3.49	-51	0.467	Neither	3
	HOH2013	1.04	-278	-0.381	1.02	42	0.284	2.06	-236	-0.281	Protein B	12
	HOH2014	3.65	-146	-0.073	0.00	-231	-0.236	3.65	-377	-0.630	Neither	0
	HOH2016	1.10	-176	-0.141	0.85	-330	-0.509	1.95	-505	-0.984	Neither	11
	HOH2024	1.27	-45	0.228	2.46	13	0.402	3.73	-32	0.523	Protein B	21
	HOH2025	2.10	-96	0.200	1.01	-134	-0.049	3.11	-230	-0.267	Neither	0
	HOH2057	3.69	91	0.627	0.00	-324	-0.480	3.69	-233	-0.272	Protein A	8
	HOH2066	2.02	-69	0.216	0.00	-99	-0.040	2.02	-168	-0.125	Neither	1
	HOH2067	0.98	27	0.266	0.00	-126	-0.038	0.98	-99	0.167	Protein A	0
	HOH2074	1.80	-100	0.159	0.00	-458	-0.846	1.80	-558	-1.138	Neither	0
	HOH2075	2.82	200	0.660	2.54	-746	-1.734	5.35	-546	-1.102	Protein A	11
	HOH2076	3.58	89	0.610	0.00	-274	-0.347	3.58	-185	-0.162	Protein A	30
	HOH2078	0.94	-42	0.206	0.00	-128	-0.040	0.94	-170	-0.129	Neither	27
	HOH2079	0.00	-198	-0.156	0.00	-155	-0.072	0.00	-353	-0.558	Neither	0
	HOH2161	1.20	302	0.459	1.10	-328	-0.503	2.30	-26	0.332	Protein A	2
	HOH2162	2.36	107	0.489	0.00	-215	-0.198	2.36	-108	0.241	Protein A	21
	HOH2164	2.00	423	0.719	0.00	-229	-0.231	2.00	194	0.539	Protein A	0
	HOH2165	1.05	190	0.345	0.00	-237	-0.253	1.05	-47	0.222	Protein A	6
	HOH2022	1.02	-43	0.220	1.13	-64	0.216	2.14	-106	0.196	Neither	29
	HOH2029	0.86	-164	-0.116	2.52	471	0.762	3.38	306	0.771	Protein B	32
	HOH2030	1.05	-159	-0.105	2.47	92	0.487	3.52	-67	0.455	Protein B	0
	HOH2032	1.16	-187	-0.167	3.91	374	0.857	5.07	187	0.891	Protein B	0
	HOH2033	2.56	32	0.436	2.87	387	0.756	5.43	419	0.978	Both	6
	HOH2046	0.00	-136	-0.047	0.97	59	0.289	0.97	-77	0.184	Protein B	21
	HOH2047	0.98	-176	-0.143	1.14	101	0.348	2.11	-76	0.231	Protein B	46
	HOH2050	0.00	-185	-0.129	1.02	-85	0.186	1.02	-270	-0.362	Neither	30
	HOH2051	0.00	43	-0.038	2.13	-19	0.309	2.13	24	0.362	Protein B	14
	HOH2053	0.92	-25	0.216	0.93	-55	0.194	1.84	-80	0.190	Neither	29
	HOH2059	2.61	-101	0.293	2.19	-66	0.261	4.80	-167	-0.119	Both	31
	HOH2092	0.90	-90	0.161	1.22	-73	0.201	2.12	-163	-0.114	Neither	32
	HOH2104	0.00	-165	-0.089	0.75	-245	-0.302	0.75	-410	-0.719	Neither	0
	HOH2108	0.87	22	0.243	0.88	-70	0.174	1.75	-48	0.230	Neither	0
	HOH2109	0.00	-140	-0.052	0.00	-82	-0.040	0.00	-222	-0.215	Neither	47
	HOH2112	0.00	65	-0.038	0.00	-313	-0.450	0.00	-247	-0.279	Neither	13

2J59	HOH56	0.87	-59	0.182	0.87	191	0.305	1.75	132	0.455	Protein B	16
	HOH76	0.00	-85	-0.040	0.00	-200	-0.161	0.00	-285	-0.377	Neither	53
	HOH153	1.23	-18	0.256	1.14	102	0.351	2.38	84	0.467	Both	45
	HOH210	2.46	252	0.634	1.19	87	0.349	3.65	339	0.816	Both	22
	HOH214	1.06	-15	0.249	1.05	-69	0.204	2.11	-84	0.219	Neither	1
	HOH215	1.07	-104	0.178	0.96	-34	0.216	2.03	-139	-0.060	Neither	39
	HOH216	1.00	-178	-0.147	0.00	-321	-0.473	1.00	-499	-0.967	Neither	18
	HOH222	1.11	-694	-1.562	0.97	93	0.311	2.08	-601	-1.269	Protein B	22
	HOH223	1.11	-102	0.184	0.87	-160	-0.107	1.98	-262	-0.343	Neither	4
	HOH224	2.57	-212	-0.225	1.28	272	0.472	3.85	60	0.624	Protein B	5
	HOH225	2.42	-66	0.305	2.24	-72	0.263	4.66	-139	-0.054	Both	0
	HOH265	2.36	-504	-0.979	0.00	-298	-0.412	2.36	-802	-1.926	Neither	0
	HOH389	1.15	-56	0.222	1.04	36	0.282	2.19	-20	0.318	Protein B	0
	HOH393	4.26	86	0.673	1.47	19	0.303	5.72	105	0.763	Both	5
	HOH394	1.18	43	0.311	1.10	-57	0.222	2.28	-14	0.343	Protein A	36
	HOH397	1.28	-176	-0.143	2.31	92	0.466	3.58	-84	0.447	Protein B	28
	HOH528	1.27	1	0.276	2.01	34	0.354	3.28	35	0.525	Both	10
	HOH1025	2.82	59	0.495	1.37	180	0.439	4.18	239	0.843	Both	0
	HOH1027	0.94	72	0.292	0.00	-24	-0.039	0.94	49	0.275	Protein A	9
	HOH1029	1.09	102	0.338	0.00	-63	-0.039	1.09	39	0.294	Protein A	58
	HOH1213	0.00	-432	-0.773	0.72	-54	0.158	0.72	-486	-0.929	Neither	30
	HOH1222	1.15	-2	0.269	3.76	10	0.567	4.91	8	0.645	Both	27
	HOH1223	0.84	-8	0.215	1.06	82	0.319	1.90	73	0.395	Protein B	13
	HOH1225	0.83	-69	0.166	1.02	178	0.340	1.85	109	0.436	Protein B	22
	HOH1236	0.00	-243	-0.267	1.08	231	0.365	1.08	-12	0.254	Protein B	6
	HOH1247	0.00	-183	-0.124	1.88	-227	-0.259	1.88	-410	-0.719	Neither	10
	HOH1248	0.00	-381	-0.633	2.25	-31	0.318	2.25	-412	-0.723	Protein B	9
	HOH1249	3.93	-71	0.511	1.59	588	0.750	5.52	518	1.016	Both	27
	HOH1250	1.22	-95	0.177	2.66	507	0.790	3.89	412	0.870	Protein B	2
	HOH1251	1.28	-264	-0.347	2.65	490	0.782	3.92	226	0.816	Protein B	25
	HOH1252	1.26	99	0.370	2.56	230	0.637	3.82	330	0.831	Both	18
	HOH1255	0.00	-230	-0.234	1.05	126	0.342	1.05	-104	0.174	Protein B	12
	HOH1257	1.13	58	0.314	1.28	66	0.343	2.41	124	0.516	Both	38
	HOH1258	0.95	-45	0.206	2.24	251	0.607	3.19	206	0.718	Protein B	9
	HOH1264	1.32	22	0.300	4.22	116	0.704	5.54	138	0.816	Both	1
	HOH1265	1.95	-50	0.234	0.99	237	0.350	2.94	187	0.665	Protein B	0
	HOH1266	0.00	-102	-0.040	0.95	355	0.409	0.95	253	0.353	Protein B	1
	HOH1279	0.00	-397	-0.677	0.92	86	0.298	0.92	-311	-0.462	Protein B	0
	HOH1284	0.00	-58	-0.039	0.94	183	0.323	0.94	125	0.324	Protein B	4
	HOH1289	0.85	-268	-0.356	1.06	-75	0.200	1.90	-342	-0.541	Neither	0
	HOH1291	0.00	-169	-0.095	2.30	35	0.402	2.30	-134	-0.049	Protein B	6
	HOH1292	0.00	-249	-0.282	0.93	-159	-0.105	0.93	-408	-0.713	Neither	12
	HOH1294	0.92	10	0.242	1.04	-20	0.241	1.96	-11	0.288	Neither	28
	HOH1304	3.61	20	0.554	1.31	-95	0.168	4.92	-74	0.569	Protein A	40
	HOH1306	0.90	-103	0.150	1.12	150	0.364	2.02	48	0.372	Protein B	34
2JJS	HOH2066	1.04	47	0.292	0.00	-216	-0.200	1.04	-170	-0.128	Protein A	5
	HOH2071	1.02	133	0.339	0.00	-57	-0.039	1.02	76	0.308	Protein A	4
	HOH2073	2.24	-245	-0.300	1.27	165	0.413	3.51	-79	0.441	Protein B	8
	HOH2074	2.45	56	0.447	2.60	67	0.477	5.05	123	0.765	Both	0
	HOH2082	0.97	3	0.247	0.00	-79	-0.040	0.97	-76	0.184	Neither	36
	HOH2087	1.15	-114	-0.004	1.13	409	0.480	2.28	296	0.641	Protein B	33
	HOH2040	2.55	-654	-1.436	1.28	-171	-0.132	3.82	-826	-2.009	Neither	19
	HOH2064	1.05	387	0.442	0.00	-159	-0.078	1.05	228	0.357	Protein A	53
	HOH2065	1.04	65	0.304	0.90	-73	0.174	1.94	-8	0.291	Protein A	25
	HOH2084	5.15	102	0.738	0.00	-77	-0.040	5.15	25	0.672	Protein A	0
	HOH2086	2.30	-24	0.335	1.20	310	0.466	3.50	287	0.778	Both	53
	HOH2088	2.25	-57	0.285	0.00	-62	-0.039	2.25	-119	-0.016	Protein A	17
	HOH2109	1.04	115	0.336	0.00	-337	-0.515	1.04	-223	-0.249	Protein A	44
	HOH2111	0.97	-189	-0.172	0.93	173	0.322	1.90	-17	0.279	Protein B	16
	HOH2113	1.08	9	0.270	0.95	-11	0.233	2.03	-2	0.311	Protein A	8

	HOH2003	0.00	-75	-0.040	1.00	-59	0.203	1.00	-133	-0.047	Neither	47
	HOH2004	0.81	178	0.296	0.95	-108	0.155	1.77	70	0.383	Protein A	1
	HOH2012	2.09	-186	-0.165	1.28	37	0.314	3.37	-149	-0.080	Protein B	17
	HOH2043	0.87	-36	0.200	1.05	-82	0.193	1.92	-117	-0.014	Neither	18
	HOH2048	0.92	-7	0.230	2.53	162	0.580	3.44	155	0.685	Protein B	23
	HOH2049	0.00	33	-0.038	3.31	256	0.743	3.31	289	0.756	Protein B	0
	HOH2055	0.95	-18	0.228	1.11	315	0.425	2.07	297	0.626	Protein B	18
	HOH2061	0.00	-324	-0.480	0.97	-91	0.171	0.97	-415	-0.733	Neither	13
	HOH2062	0.00	-251	-0.287	3.82	-161	-0.106	3.82	-411	-0.722	Neither	0
	HOH2067	1.30	-79	0.187	2.70	-33	0.384	4.00	-113	0.006	Protein B	25
	HOH2070	0.00	-182	-0.122	2.39	649	0.820	2.39	467	0.751	Protein B	40
	HOH2075	0.00	-151	-0.065	1.98	128	0.467	1.98	-23	0.271	Protein B	28
	HOH2121	0.00	-43	-0.039	5.32	188	0.907	5.32	144	0.820	Protein B	0
	HOH2122	0.00	-18	-0.039	4.01	682	0.960	4.01	664	0.956	Protein B	7
	HOH2123	0.00	-117	-0.032	3.95	155	0.749	3.95	38	0.611	Protein B	0
	HOH2124	0.95	-215	-0.232	0.97	-141	-0.064	1.91	-356	-0.577	Neither	0
	HOH2125	2.03	-108	0.164	1.34	-69	0.196	3.37	-177	-0.143	Neither	0
	HOH2126	2.61	-196	-0.186	1.38	325	0.549	3.99	130	0.708	Protein B	0
	HOH2127	1.31	-293	-0.416	2.72	389	0.741	4.03	96	0.666	Protein B	0
	HOH2128	1.08	-428	-0.768	1.22	613	0.607	2.30	185	0.572	Protein B	8
	HOH2129	1.11	-255	-0.326	2.57	710	0.846	3.68	455	0.866	Protein B	9
	HOH2130	1.04	-6	0.251	1.04	-30	0.233	2.08	-37	0.274	Protein A	0
	HOH2131	1.05	108	0.334	2.40	41	0.424	3.44	149	0.675	Both	6
	HOH2134	0.00	-156	-0.073	3.48	246	0.762	3.48	91	0.599	Protein B	5
2NPT	HOH109	2.47	-137	-0.056	1.27	-9	0.266	3.74	-146	-0.072	Protein B	0
	HOH110	2.69	-470	-0.883	1.23	-51	0.223	3.92	-521	-1.028	Neither	0
	HOH112	2.62	275	0.668	1.26	-64	0.207	3.89	212	0.813	Protein A	0
	HOH114	2.96	774	0.889	2.63	-230	-0.267	5.59	544	1.027	Protein A	0
	HOH117	1.32	-185	-0.162	2.30	87	0.460	3.62	-97	0.440	Protein B	3
	HOH129	0.93	298	0.374	0.82	-82	0.154	1.75	217	0.523	Protein A	0
	HOH150	1.39	58	0.345	2.12	-18	0.307	3.51	41	0.559	Both	1
	HOH168	0.98	200	0.328	0.90	-52	0.191	1.88	148	0.484	Protein A	16
	HOH191	2.79	803	0.879	2.40	-36	0.337	5.19	767	1.056	Both	9
	HOH124	3.96	-19	0.562	1.39	-136	-0.054	5.35	-155	-0.091	Protein A	18
	HOH125	1.20	-34	0.241	2.52	-59	0.329	3.72	-92	0.462	Protein B	19
	HOH145	1.13	336	0.446	2.24	-281	-0.388	3.37	56	0.555	Protein A	26
	HOH166	1.81	106	0.429	0.00	-140	-0.052	1.81	-34	0.252	Protein A	29
	HOH172	0.99	218	0.337	1.18	-49	0.227	2.17	169	0.539	Protein A	18
	HOH190	0.99	-95	0.173	1.02	3	0.255	2.01	-92	0.182	Protein B	3
2NQD	HOH569	2.49	-203	-0.204	2.53	-268	-0.357	5.02	-471	-0.887	Neither	9
	HOH570	1.27	-93	0.174	2.18	-256	-0.328	3.46	-349	-0.557	Neither	42
	HOH579	1.07	51	0.299	0.00	-313	-0.450	1.07	-261	-0.340	Protein A	11
	HOH580	1.20	-432	-0.777	1.18	306	0.456	2.38	-126	-0.031	Protein B	14
	HOH582	2.02	-159	-0.105	0.00	-288	-0.384	2.02	-447	-0.818	Neither	23
	HOH584	2.19	-375	-0.625	0.00	-81	-0.040	2.19	-456	-0.843	Neither	24
	HOH600	2.43	34	0.421	0.00	-58	-0.039	2.43	-25	0.356	Protein A	36
	HOH610	0.91	-212	-0.223	0.93	21	0.253	1.84	-190	-0.175	Protein B	22
	HOH613	1.02	-8	0.247	0.86	22	0.243	1.88	14	0.319	Neither	4
	HOH620	3.44	212	0.752	0.00	-44	-0.039	3.44	169	0.706	Protein A	25
	HOH623	0.00	-14	-0.039	0.00	1	-0.039	0.00	-13	-0.039	Neither	8
	HOH628	2.05	-94	0.191	0.00	-72	-0.040	2.05	-166	-0.120	Neither	1
	HOH648	2.16	105	0.462	0.97	60	0.288	3.13	166	0.662	Both	1
	HOH650	2.03	152	0.502	0.00	7	-0.039	2.03	158	0.509	Protein A	43
	HOH695	0.88	80	0.287	0.00	-78	-0.040	0.88	3	0.231	Protein A	1
	HOH714	0.75	55	0.246	0.00	-33	-0.039	0.75	22	0.222	Neither	18
	HOH1037	1.24	77	0.348	2.71	315	0.700	3.95	392	0.868	Both	0
	HOH1038	0.91	49	0.271	0.95	-77	0.180	1.86	-28	0.262	Protein A	14
	HOH1077	0.00	-190	-0.140	0.92	-906	-2.297	0.92	-1096	-3.034	Neither	32
	HOH1079	0.00	-223	-0.216	0.00	-756	-1.768	0.00	-979	-2.571	Neither	30
	HOH1087	1.15	-182	-0.157	1.15	81	0.337	2.30	-101	0.238	Protein B	0

	HOH1088	0.90	-134	-0.049	1.06	231	0.361	1.96	97	0.427	Protein B	0
	HOH1099	0.00	-302	-0.422	2.36	397	0.711	2.36	95	0.475	Protein B	26
	HOH1100	0.00	-309	-0.441	2.41	420	0.728	2.41	111	0.500	Protein B	1
	HOH1149	0.00	-50	-0.039	3.26	205	0.726	3.26	154	0.661	Protein B	11
	HOH1150	0.89	-17	0.218	0.95	45	0.275	1.84	29	0.336	Protein B	4
	HOH1155	0.00	-71	-0.040	1.96	280	0.606	1.96	209	0.545	Protein B	11
	HOH1158	0.84	-275	-0.373	1.14	226	0.382	1.98	-49	0.235	Protein B	0
	HOH1161	0.00	-78	-0.040	0.97	101	0.317	0.97	23	0.261	Protein B	24
	HOH1175	0.00	-51	-0.039	0.00	-40	-0.039	0.00	-91	-0.040	Neither	0
	HOH1176	0.90	-184	-0.161	2.17	-42	0.288	3.07	-226	-0.256	Protein B	40
	HOH1178	2.24	216	0.585	1.00	180	0.336	3.24	397	0.798	Both	14
	HOH1193	1.19	-388	-0.659	3.96	-76	0.508	5.15	-464	-0.866	Protein B	23
	HOH1276	2.61	59	0.470	2.58	60	0.467	5.19	118	0.763	Both	34
	HOH1327	0.84	-30	0.199	1.09	90	0.330	1.93	59	0.379	Protein B	44
	HOH1339	1.00	29	0.271	1.13	-178	-0.147	2.13	-149	-0.084	Protein A	30
2NS1	HOH23	2.40	-295	-0.422	1.24	154	0.402	3.64	-141	-0.061	Protein B	19
	HOH27	0.00	-307	-0.435	0.00	-73	-0.040	0.00	-380	-0.629	Neither	10
	HOH36	4.04	320	0.849	1.30	-62	0.207	5.34	258	0.927	Protein A	21
	HOH39	3.93	231	0.817	1.38	79	0.366	5.31	310	0.935	Both	45
	HOH73	2.16	1	0.339	0.00	-157	-0.076	2.16	-157	-0.100	Protein A	48
	HOH108	1.12	54	0.310	0.88	-116	-0.009	2.00	-61	0.221	Protein A	38
	HOH142	2.03	-61	0.231	0.00	-183	-0.124	2.03	-244	-0.300	Neither	2
	HOH199	3.62	149	0.699	0.00	-61	-0.039	3.62	88	0.615	Protein A	0
	HOH202	0.95	-39	0.210	0.80	-131	-0.042	1.74	-170	-0.130	Neither	9
	HOH212	1.20	152	0.388	1.04	64	0.303	2.23	216	0.584	Both	11
	HOH228	0.00	-70	-0.039	0.81	3	0.218	0.81	-67	0.163	Neither	4
	HOH232	2.46	362	0.700	0.00	-154	-0.070	2.46	208	0.613	Protein A	1
	HOH390	3.49	-74	0.444	1.41	100	0.392	4.90	27	0.662	Both	14
	HOH403	1.22	173	0.399	1.22	-58	0.216	2.44	115	0.510	Protein A	26
	HOH405	1.03	-126	-0.032	2.42	212	0.608	3.45	85	0.591	Protein B	17
	HOH409	0.00	-150	-0.065	0.86	-79	0.163	0.86	-229	-0.264	Neither	13
	HOH423	0.00	-26	-0.039	3.51	78	0.593	3.51	52	0.569	Protein B	26
	HOH431	1.09	7	0.270	2.18	474	0.745	3.27	481	0.837	Both	14
	HOH471	1.05	-103	0.175	1.09	-120	-0.017	2.14	-223	-0.250	Neither	16
	HOH477	0.00	-62	-0.039	0.73	-244	-0.299	0.73	-307	-0.451	Neither	0
2NXY	HOH8	1.39	65	0.351	3.91	361	0.852	5.30	426	0.974	Both	11
	HOH10	0.98	26	0.267	1.12	-11	0.260	2.11	16	0.349	Both	18
	HOH16	1.24	13	0.288	2.47	-50	0.331	3.71	-37	0.515	Both	9
	HOH24	1.15	409	0.493	1.17	-404	-0.702	2.32	6	0.373	Protein A	5
	HOH27	1.09	-363	-0.595	1.18	76	0.337	2.26	-287	-0.403	Protein B	0
	HOH28	1.18	100	0.357	2.36	22	0.398	3.54	122	0.646	Both	2
	HOH36	1.30	-5	0.271	2.65	95	0.511	3.95	91	0.655	Both	3
	HOH46	1.10	409	0.460	1.16	-7	0.265	2.26	402	0.708	Both	14
	HOH60	0.00	-187	-0.131	1.06	141	0.349	1.06	-46	0.225	Protein B	43
	HOH61	1.16	-88	0.192	2.30	-161	-0.109	3.46	-248	-0.309	Neither	0
	HOH74	0.79	-229	-0.265	1.05	-24	0.240	1.85	-253	-0.321	Neither	21
	HOH82	0.98	-15	0.234	2.57	-647	-1.413	3.54	-662	-1.461	Neither	46
	HOH94	1.24	41	0.314	2.40	538	0.783	3.64	579	0.904	Both	29
	HOH99	0.90	-78	0.170	1.27	16	0.292	2.17	-62	0.260	Protein B	42
	HOH102	0.00	-264	-0.322	2.09	441	0.727	2.09	177	0.535	Protein B	14
	HOH115	0.00	-359	-0.573	0.91	70	0.285	0.91	-289	-0.408	Protein B	14
	HOH118	0.00	-276	-0.352	1.04	-361	-0.589	1.04	-637	-1.379	Neither	0
	HOH134	1.45	41	0.328	3.83	22	0.589	5.28	63	0.710	Both	9
	HOH142	1.08	-173	-0.135	2.49	168	0.581	3.57	-5	0.523	Protein B	0
	HOH144	2.26	72	0.438	1.21	-107	0.166	3.46	-35	0.479	Protein A	3
	HOH152	2.18	233	0.588	1.00	-89	0.179	3.18	145	0.637	Protein A	0
	HOH153	0.00	-109	-0.040	1.03	8	0.261	1.03	-101	0.174	Protein B	30
	HOH161	0.00	-21	-0.039	0.75	-250	-0.314	0.75	-271	-0.364	Neither	3
	HOH180	0.00	-57	-0.039	0.95	39	0.270	0.95	-18	0.227	Protein B	34
	HOH218	0.87	-22	0.210	1.18	-138	-0.058	2.04	-160	-0.108	Neither	21

	HOH236	0.86	-45	0.191	0.93	143	0.324	1.79	99	0.420	Protein B	4
	HOH268	1.18	462	0.531	1.11	-212	-0.225	2.29	250	0.612	Protein A	5
	HOH275	2.30	-91	0.253	0.98	36	0.274	3.29	-54	0.435	Both	0
	HOH282	2.12	8	0.341	0.93	-172	-0.134	3.05	-165	-0.116	Protein A	36
	HOH284	3.80	296	0.817	0.00	-382	-0.634	3.80	-85	0.483	Protein A	31
	HOH314	2.16	147	0.514	1.03	-148	-0.080	3.19	-1	0.477	Protein A	3
	HOH328	2.06	-86	0.202	0.93	-62	0.189	2.99	-148	-0.078	Neither	10
	HOH341	0.99	-43	0.215	0.98	23	0.264	1.98	-20	0.276	Protein B	34
	HOH357	1.00	-136	-0.053	0.86	63	0.272	1.86	-73	0.200	Protein B	13
	HOH358	5.44	-96	0.571	0.00	-185	-0.127	5.44	-281	-0.387	Protein A	18
	HOH396	2.49	133	0.538	1.23	-84	0.187	3.72	49	0.595	Protein A	31
	HOH605	1.17	38	0.304	1.17	-349	-0.558	2.34	-311	-0.462	Protein A	2
	HOH676	0.93	182	0.320	0.00	-125	-0.037	0.93	57	0.280	Protein A	33
	HOH698	1.83	-19	0.273	0.00	-504	-0.976	1.83	-523	-1.035	Protein A	36
	HOH717	1.09	196	0.353	1.09	154	0.355	2.18	349	0.670	Both	42
	HOH719	0.00	-160	-0.079	0.00	-176	-0.110	0.00	-336	-0.511	Neither	0
	HOH754	0.00	-246	-0.275	0.75	-119	-0.017	0.75	-365	-0.599	Neither	0
	HOH762	0.88	33	0.254	0.00	-280	-0.363	0.88	-247	-0.306	Protein A	8
	HOH781	2.03	172	0.524	0.00	-166	-0.090	2.03	6	0.321	Protein A	11
2NZ8	HOH1	4.28	20	0.619	1.42	-152	-0.090	5.70	-132	-0.039	Protein A	0
	HOH13	2.08	-247	-0.306	0.00	-230	-0.233	2.08	-476	-0.901	Neither	15
	HOH14	1.27	1	0.276	2.22	-9	0.338	3.48	-8	0.509	Both	0
	HOH15	2.41	-506	-0.986	0.00	-158	-0.077	2.41	-664	-1.467	Neither	0
	HOH16	5.24	132	0.790	0.00	-403	-0.694	5.24	-272	-0.365	Protein A	10
	HOH18	2.63	133	0.555	1.14	-584	-1.218	3.77	-452	-0.832	Protein A	8
	HOH21	1.19	0	0.273	0.94	-78	0.177	2.12	-78	0.230	Protein A	0
	HOH24	5.45	245	0.933	0.00	-12	-0.039	5.45	233	0.934	Protein A	27
	HOH26	2.59	189	0.618	0.99	76	0.304	3.59	265	0.781	Both	2
	HOH30	2.48	394	0.720	1.03	-253	-0.319	3.51	141	0.671	Protein A	14
	HOH37	1.06	133	0.348	1.02	-150	-0.084	2.08	-17	0.302	Protein A	11
	HOH39	1.21	29	0.300	1.15	-199	-0.194	2.36	-170	-0.130	Protein A	22
	HOH41	1.32	126	0.402	2.18	-34	0.301	3.50	92	0.604	Both	0
	HOH43	4.44	221	0.867	1.20	-208	-0.215	5.64	13	0.680	Protein A	26
	HOH46	0.72	-163	-0.113	0.00	-237	-0.253	0.72	-400	-0.693	Neither	14
	HOH50	0.84	96	0.291	0.00	-128	-0.040	0.84	-32	0.197	Protein A	19
	HOH58	1.34	-110	0.001	2.23	72	0.433	3.57	-39	0.491	Protein B	0
	HOH59	1.06	-45	0.225	0.93	3	0.239	1.99	-42	0.247	Neither	39
	HOH66	1.08	64	0.311	0.95	32	0.264	2.03	96	0.433	Both	26
	HOH69	1.01	136	0.339	0.85	-58	0.178	1.86	78	0.399	Protein A	0
	HOH70	2.18	70	0.424	1.08	-94	0.188	3.26	-24	0.463	Protein A	4
	HOH80	1.87	76	0.397	0.00	-32	-0.039	1.87	44	0.357	Protein A	0
	HOH89	0.95	97	0.312	0.83	-10	0.214	1.79	88	0.406	Protein A	0
	HOH97	1.09	445	0.472	0.96	67	0.291	2.05	512	0.768	Both	25
	HOH99	1.36	66	0.350	0.00	-450	-0.824	1.36	-384	-0.649	Protein A	0
	HOH109	3.65	206	0.782	1.25	-86	0.184	4.90	120	0.753	Protein A	20
	HOH113	0.93	179	0.320	1.18	-314	-0.470	2.10	-135	-0.053	Protein A	5
	HOH114	2.82	287	0.697	2.96	203	0.683	5.78	489	1.016	Both	20
	HOH115	1.30	29	0.307	2.64	190	0.626	3.94	219	0.818	Both	21
	HOH116	3.53	-6	0.517	1.46	-174	-0.138	4.99	-180	-0.149	Protein A	0
	HOH119	0.00	-137	-0.049	0.98	6	0.250	0.98	-131	-0.043	Protein B	6
	HOH123	1.05	-360	-0.586	1.26	218	0.421	2.31	-142	-0.067	Protein B	13
	HOH125	1.02	266	0.375	1.14	126	0.362	2.16	391	0.697	Both	18
	HOH134	0.00	270	-0.038	3.16	110	0.583	3.16	379	0.781	Protein B	30
	HOH138	0.00	-283	-0.372	0.00	-221	-0.211	0.00	-504	-0.977	Neither	27
	HOH142	1.06	8	0.266	2.11	-117	-0.013	3.17	-109	0.009	Protein A	21
	HOH152	1.15	213	0.378	2.04	312	0.637	3.19	525	0.847	Both	2
	HOH161	0.83	-47	0.183	1.06	433	0.461	1.88	385	0.680	Protein B	20
	HOH162	1.15	-125	-0.029	1.22	162	0.397	2.37	37	0.415	Protein B	25
	HOH163	4.30	-96	0.512	1.62	149	0.461	5.92	53	0.723	Both	44
	HOH164	0.00	-132	-0.043	1.98	-2	0.302	1.98	-134	-0.049	Protein B	19

	HOH166	1.27	-201	-0.198	2.53	-172	-0.134	3.80	-373	-0.619	Neither	14
2ODE	HOH5	2.77	363	0.732	1.21	-248	-0.308	3.98	115	0.685	Protein A	34
	HOH8	2.30	57	0.427	1.14	51	0.311	3.44	108	0.613	Both	34
	HOH17	2.55	610	0.818	1.05	-24	0.240	3.60	586	0.903	Protein A	14
	HOH20	0.77	-260	-0.336	0.00	-40	-0.039	0.77	-300	-0.433	Neither	2
	HOH22	2.68	196	0.637	2.37	68	0.449	5.05	264	0.911	Both	43
	HOH26	1.14	104	0.351	1.12	-228	-0.261	2.25	-124	-0.027	Protein A	26
	HOH44	0.98	-161	-0.108	0.00	-26	-0.039	0.98	-187	-0.166	Neither	1
	HOH59	1.23	-47	0.227	1.17	77	0.335	2.40	30	0.412	Protein B	0
	HOH92	2.67	-78	0.329	1.37	417	0.606	4.04	339	0.856	Both	40
	HOH98	0.00	-27	-0.039	0.00	-62	-0.039	0.00	-89	-0.040	Neither	29
	HOH120	0.97	531	0.473	1.91	-11	0.287	2.89	520	0.816	Both	16
	HOH123	1.04	283	0.388	0.87	-30	0.205	1.91	253	0.576	Protein A	28
	HOH139	1.15	-226	-0.258	0.84	-28	0.200	1.98	-254	-0.324	Neither	4
	HOH187	0.00	-212	-0.190	0.70	92	0.264	0.70	-121	-0.021	Protein B	0
	HOH190	0.95	-207	-0.213	0.89	17	0.244	1.85	-191	-0.176	Neither	0
	HOH234	0.75	7	0.211	0.00	-132	-0.044	0.75	-125	-0.030	Neither	0
	HOH259	0.86	-209	-0.217	0.90	86	0.295	1.76	-123	-0.026	Protein B	17
	HOH261	1.02	330	0.411	1.92	52	0.369	2.94	382	0.760	Both	20
	HOH262	0.00	-262	-0.316	2.37	248	0.621	2.37	-14	0.358	Protein B	5
	HOH264	1.47	-188	-0.169	4.63	484	0.956	6.10	296	0.961	Protein B	6
	HOH265	1.21	-97	0.177	4.40	273	0.866	5.61	176	0.899	Protein B	4
	HOH267	0.00	-179	-0.116	3.71	-648	-1.416	3.71	-827	-2.015	Neither	20
	HOH271	1.23	60	0.331	2.70	198	0.642	3.93	258	0.820	Both	49
	HOH274	0.00	-406	-0.701	1.05	-18	0.244	1.05	-423	-0.754	Neither	23
	HOH279	0.00	-139	-0.051	0.99	233	0.346	0.99	94	0.315	Protein B	23
	HOH282	1.35	278	0.501	3.67	-164	-0.114	5.02	114	0.747	Protein A	23
	HOH284	2.39	38	0.420	1.37	63	0.348	3.76	101	0.646	Both	8
	HOH288	0.00	-273	-0.346	1.02	346	0.419	1.02	73	0.307	Protein B	22
	HOH307	0.00	-29	-0.039	0.89	26	0.251	0.89	-2	0.229	Protein B	26
	HOH315	0.00	-274	-0.347	0.97	350	0.410	0.97	76	0.301	Protein B	28
	HOH319	2.33	395	0.708	1.45	467	0.660	3.78	862	0.962	Both	4
	HOH320	0.00	-57	-0.039	0.93	79	0.295	0.93	21	0.254	Protein B	21
	HOH329	1.07	-42	0.230	2.32	332	0.669	3.39	290	0.766	Protein B	0
	HOH334	0.00	-30	-0.039	1.97	173	0.518	1.97	143	0.486	Protein B	16
	HOH348	0.92	69	0.286	0.97	47	0.280	1.89	116	0.447	Both	0
	HOH351	0.00	-126	-0.039	1.72	-94	0.163	1.72	-221	-0.245	Neither	21
	HOH359	2.20	-59	0.271	1.18	199	0.384	3.38	140	0.653	Both	2
	HOH383	2.20	660	0.821	0.00	131	-0.038	2.20	791	0.851	Protein A	2
	HOH385	2.01	94	0.428	1.33	128	0.406	3.34	222	0.740	Both	0
	HOH504	1.05	-46	0.223	0.00	-92	-0.040	1.05	-138	-0.056	Neither	22
	HOH559	2.26	101	0.470	1.11	224	0.371	3.38	325	0.779	Both	24
	HOH560	1.37	21	0.301	0.88	-104	0.146	2.26	-83	0.252	Protein A	18
	HOH562	1.18	45	0.311	1.12	-378	-0.633	2.30	-333	-0.518	Protein A	3
	HOH571	1.22	-135	-0.051	1.02	-77	0.193	2.24	-212	-0.224	Neither	39
2OMZ	HOH2	3.54	113	0.632	1.19	-23	0.251	4.74	90	0.706	Both	0
	HOH30	2.48	-320	-0.483	0.93	13	0.249	3.42	-306	-0.450	Neither	4
	HOH34	1.38	153	0.431	2.64	160	0.593	4.02	312	0.844	Both	0
	HOH37	4.03	-60	0.529	1.29	-93	0.171	5.32	-153	-0.087	Protein A	11
	HOH69	3.70	28	0.575	1.13	-70	0.210	4.83	-42	0.596	Protein A	0
	HOH109	1.16	231	0.392	1.08	-65	0.212	2.23	166	0.544	Protein A	1
	HOH111	3.93	-20	0.558	1.27	-113	-0.003	5.20	-133	-0.041	Protein A	4
	HOH115	2.96	-44	0.405	0.00	-179	-0.115	2.96	-223	-0.249	Protein A	1
	HOH123	1.12	149	0.364	1.07	-165	-0.117	2.19	-16	0.323	Protein A	1
	HOH126	2.63	-141	-0.063	2.62	38	0.450	5.25	-103	0.556	Protein B	17
	HOH127	2.31	-208	-0.214	0.97	-9	0.239	3.28	-216	-0.234	Neither	17
	HOH135	2.59	272	0.662	2.59	44	0.452	5.18	315	0.929	Both	0
	HOH146	2.47	527	0.782	0.00	-383	-0.638	2.47	144	0.550	Protein A	12
	HOH157	3.71	152	0.716	0.00	-188	-0.133	3.71	-35	0.516	Protein A	21
	HOH172	2.36	185	0.580	1.11	7	0.274	3.47	192	0.742	Both	18

	HOH182	2.08	-75	0.223	0.00	-215	-0.197	2.08	-290	-0.410	Neither	0
	HOH188	4.24	-25	0.576	1.12	4	0.272	5.36	-21	0.639	Both	0
	HOH190	1.79	164	0.491	0.00	-440	-0.796	1.79	-277	-0.377	Protein A	22
	HOH272	1.78	79	0.395	0.00	-148	-0.061	1.78	-69	0.202	Protein A	22
	HOH295	3.40	172	0.706	0.00	-505	-0.981	3.40	-334	-0.519	Protein A	4
	HOH308	1.97	542	0.787	0.00	-202	-0.166	1.97	340	0.657	Protein A	8
	HOH327	4.10	718	0.975	0.99	35	0.274	5.09	753	1.049	Both	34
	HOH372	0.83	45	0.253	0.00	-250	-0.286	0.83	-206	-0.210	Protein A	7
	HOH375	1.20	341	0.487	1.20	-89	0.186	2.40	251	0.626	Protein A	1
	HOH400	1.24	12	0.286	1.20	-31	0.243	2.44	-19	0.364	Protein A	11
	HOH454	2.42	-235	-0.278	0.00	-229	-0.231	2.42	-464	-0.866	Neither	26
	HOH483	1.22	378	0.514	1.17	158	0.382	2.39	536	0.781	Both	2
	HOH501	2.82	189	0.650	2.33	-127	-0.034	5.15	62	0.703	Protein A	38
	HOH504	0.91	-67	0.182	0.92	66	0.285	1.84	-1	0.298	Protein B	4
	HOH506	3.48	23	0.538	0.00	-399	-0.683	3.48	-377	-0.630	Protein A	22
	HOH538	1.19	-230	-0.267	2.10	263	0.602	3.30	33	0.525	Protein B	6
	HOH551	3.87	680	0.947	1.33	79	0.361	5.20	759	1.056	Both	21
	HOH573	2.01	46	0.370	0.00	-323	-0.477	2.01	-276	-0.377	Protein A	30
	HOH598	1.14	68	0.323	2.05	-76	0.215	3.19	-8	0.470	Protein A	0
	HOH624	2.37	-193	-0.182	1.05	17	0.271	3.42	-176	-0.142	Protein B	8
	HOH633	1.04	153	0.346	1.00	-4	0.246	2.04	150	0.501	Protein A	35
	HOH637	1.11	213	0.364	0.90	-98	0.153	2.01	115	0.455	Protein A	49
	HOH647	1.18	584	0.568	0.97	47	0.280	2.15	631	0.813	Both	24
	HOH654	1.12	75	0.325	1.02	60	0.297	2.13	135	0.495	Both	11
	HOH660	3.66	129	0.670	1.08	-13	0.254	4.74	116	0.736	Both	1
	HOH687	1.07	-260	-0.337	2.27	-69	0.272	3.34	-329	-0.508	Protein B	1
	HOH688	0.00	-168	-0.094	0.98	-17	0.234	0.98	-185	-0.162	Neither	0
	HOH689	2.67	70	0.489	2.92	212	0.681	5.60	281	0.942	Both	7
	HOH696	2.03	-126	-0.033	0.00	-5	-0.039	2.03	-131	-0.043	Neither	31
	HOH697	1.08	59	0.308	2.20	-135	-0.052	3.28	-76	0.411	Protein A	19
	HOH700	1.14	-58	0.220	3.64	-6	0.534	4.79	-64	0.572	Protein B	0
	HOH710	1.01	235	0.351	1.10	-477	-0.902	2.11	-242	-0.295	Protein A	12
	HOH713	0.00	-299	-0.412	0.99	70	0.299	0.99	-229	-0.264	Protein B	0
	HOH716	0.95	27	0.262	1.19	-143	-0.069	2.14	-116	-0.010	Protein A	9
	HOH717	2.51	-112	0.001	1.35	403	0.588	3.86	291	0.822	Protein B	0
	HOH721	0.00	197	-0.039	2.40	172	0.574	2.40	369	0.698	Protein B	0
	HOH725	0.00	-31	-0.039	0.83	-487	-0.930	0.83	-517	-1.019	Neither	18
	HOH731	0.00	-355	-0.562	3.10	-16	0.452	3.10	-370	-0.613	Protein B	3
	HOH735	2.27	224	0.593	1.23	-431	-0.775	3.50	-207	-0.212	Protein A	0
	HOH736	1.10	-315	-0.471	2.25	71	0.436	3.35	-243	-0.297	Protein B	5
	HOH738	1.04	-101	0.176	2.72	581	0.822	3.76	480	0.883	Protein B	0
	HOH750	0.00	-188	-0.134	0.00	-28	-0.039	0.00	-216	-0.200	Neither	21
	HOH755	2.41	23	0.406	1.30	16	0.293	3.71	40	0.586	Both	6
	HOH774	0.00	49	-0.038	0.92	-99	0.156	0.92	-50	0.197	Neither	21
	HOH782	1.23	58	0.329	3.67	10	0.553	4.90	68	0.696	Both	2
	HOH787	0.99	73	0.302	2.08	321	0.645	3.07	393	0.779	Both	16
2OT3	HOH24	2.67	-59	0.351	1.33	173	0.430	4.00	114	0.685	Both	20
	HOH40	2.42	-117	-0.012	1.24	98	0.366	3.65	-20	0.523	Protein B	0
	HOH50	0.00	-65	-0.039	0.00	-296	-0.405	0.00	-361	-0.579	Neither	33
	HOH56	0.99	-62	0.199	0.82	-39	0.189	1.81	-101	0.158	Neither	17
	HOH57	0.00	9	-0.039	0.79	-161	-0.108	0.79	-152	-0.088	Neither	27
	HOH60	2.24	70	0.433	1.14	55	0.314	3.38	125	0.630	Both	0
	HOH65	0.95	-1	0.241	0.85	-85	0.156	1.80	-87	0.178	Neither	0
	HOH74	2.80	398	0.753	0.97	-5	0.240	3.76	393	0.850	Protein A	2
	HOH76	0.99	-154	-0.094	0.00	-350	-0.548	0.99	-504	-0.980	Neither	0
	HOH104	1.25	394	0.541	1.15	-141	-0.065	2.40	253	0.627	Protein A	12
	HOH117	4.22	-292	-0.414	1.59	50	0.347	5.81	-242	-0.293	Protein B	6
	HOH177	1.23	-37	0.237	0.88	-49	0.191	2.12	-86	0.217	Neither	29
	HOH186	1.92	108	0.439	0.00	-162	-0.083	1.92	-54	0.228	Protein A	2
	HOH231	2.17	287	0.625	0.00	-29	-0.039	2.17	258	0.604	Protein A	25

	HOH300	1.43	297	0.540	4.23	-452	-0.833	5.66	-155	-0.093	Protein A	15
	HOH301	0.00	-131	-0.042	4.00	253	0.827	4.00	123	0.698	Protein B	11
	HOH303	3.88	-348	-0.556	1.56	-36	0.237	5.45	-385	-0.651	Neither	26
	HOH319	2.19	4	0.349	1.24	53	0.325	3.43	57	0.563	Both	33
	HOH322	1.29	-20	0.255	2.62	95	0.508	3.91	75	0.640	Both	9
	HOH331	0.00	-43	-0.039	1.10	175	0.356	1.10	132	0.353	Protein B	46
	HOH359	1.17	-439	-0.797	2.23	1	0.352	3.41	-438	-0.796	Protein B	24
	HOH362	0.00	-46	-0.039	1.06	-182	-0.155	1.06	-227	-0.260	Neither	22
	HOH380	0.00	-336	-0.512	3.05	110	0.572	3.05	-226	-0.256	Protein B	39
	HOH430	1.80	-111	-0.002	0.00	-139	-0.050	1.80	-250	-0.314	Neither	8
	HOH444	0.93	109	0.314	1.14	134	0.367	2.07	243	0.583	Both	13
2OUL	HOH233	3.47	-183	-0.158	1.11	-193	-0.180	4.57	-376	-0.628	Neither	2
	HOH237	3.91	-48	0.530	1.35	-303	-0.441	5.25	-350	-0.562	Protein A	0
	HOH246	1.01	-297	-0.426	0.00	-209	-0.181	1.01	-505	-0.984	Neither	0
	HOH253	3.59	198	0.765	0.00	-47	-0.039	3.59	150	0.697	Protein A	1
	HOH255	3.74	20	0.573	1.29	51	0.329	5.03	71	0.706	Both	0
	HOH308	1.53	219	0.491	2.70	48	0.470	4.22	267	0.850	Both	21
	HOH309	1.09	428	0.465	0.00	-26	-0.039	1.09	402	0.455	Protein A	19
	HOH317	2.28	34	0.398	1.04	-42	0.223	3.31	-9	0.486	Protein A	1
	HOH334	1.10	-82	0.202	0.88	-122	-0.022	1.98	-203	-0.205	Neither	0
	HOH337	1.20	-32	0.243	0.96	-59	0.197	2.16	-91	0.221	Neither	3
	HOH339	1.05	135	0.345	0.00	-312	-0.449	1.05	-177	-0.144	Protein A	4
	HOH112	0.00	-328	-0.489	3.59	-303	-0.442	3.59	-631	-1.360	Neither	9
	HOH114	1.03	-23	0.237	1.07	-307	-0.451	2.10	-330	-0.509	Neither	10
	HOH115	0.00	-79	-0.040	1.03	-316	-0.474	1.03	-395	-0.678	Neither	45
	HOH117	2.14	-249	-0.310	1.32	-83	0.181	3.46	-331	-0.513	Neither	14
	HOH120	2.36	94	0.475	1.27	-185	-0.163	3.63	-91	0.448	Protein A	11
	HOH123	0.00	-234	-0.243	0.95	-148	-0.079	0.95	-381	-0.642	Neither	11
	HOH129	1.20	-45	0.230	3.53	-82	0.441	4.72	-127	-0.026	Protein B	5
2P45	HOH195	2.70	0	0.421	2.18	-260	-0.336	4.88	-260	-0.336	Protein A	37
	HOH250	3.79	169	0.755	1.18	-16	0.258	4.97	153	0.820	Both	44
	HOH251	0.00	-76	-0.040	0.78	14	0.222	0.78	-62	0.163	Neither	39
	HOH263	1.17	126	0.373	0.96	-1	0.242	2.13	125	0.483	Protein A	41
	HOH274	2.12	53	0.395	1.06	14	0.270	3.17	67	0.542	Both	20
	HOH312	0.00	-94	-0.040	0.73	-86	0.133	0.73	-180	-0.152	Neither	33
	HOH314	1.48	180	0.462	4.14	-168	-0.122	5.62	12	0.678	Protein A	2
	HOH315	2.61	-11	0.396	2.58	-193	-0.180	5.19	-203	-0.203	Protein A	5
	HOH316	1.18	78	0.339	1.12	20	0.283	2.30	98	0.470	Both	24
	HOH317	1.18	70	0.333	0.91	-190	-0.174	2.09	-120	-0.019	Protein A	34
	HOH318	1.92	130	0.467	0.00	-62	-0.039	1.92	69	0.390	Protein A	0
	HOH319	1.01	-26	0.232	1.94	8	0.313	2.95	-18	0.432	Protein B	34
	HOH124	0.89	-30	0.208	1.11	153	0.360	2.00	123	0.463	Protein B	36
	HOH127	0.85	13	0.233	1.20	-33	0.242	2.05	-20	0.292	Neither	26
	HOH129	0.99	21	0.264	1.16	124	0.368	2.15	145	0.509	Both	20
	HOH131	0.75	-121	-0.021	0.00	35	-0.038	0.75	-86	0.138	Neither	0
	HOH173	0.97	234	0.342	0.97	-153	-0.092	1.93	81	0.406	Protein A	0
	HOH192	0.00	-27	-0.039	0.85	-17	0.210	0.85	-45	0.188	Neither	19
	HOH238	1.18	49	0.315	1.21	57	0.326	2.39	105	0.491	Both	9
	HOH239	1.18	23	0.292	2.24	-146	-0.075	3.41	-123	-0.021	Protein A	0
	HOH242	0.00	-47	-0.039	1.07	141	0.350	1.07	94	0.329	Protein B	7
2Q0O	HOH1004	1.18	216	0.390	1.08	-235	-0.277	2.26	-19	0.333	Protein A	19
	HOH1005	2.36	189	0.585	0.95	-49	0.203	3.31	140	0.645	Protein A	2
	HOH1007	1.16	-88	0.192	1.14	-6	0.265	2.30	-94	0.248	Protein B	8
	HOH1008	1.21	-79	0.195	1.13	-168	-0.125	2.34	-247	-0.306	Neither	1
	HOH1011	1.31	-116	-0.011	2.52	-178	-0.147	3.82	-294	-0.419	Neither	0
	HOH1016	1.11	-465	-0.870	0.91	-60	0.187	2.02	-526	-1.043	Neither	1
	HOH1023	0.86	-209	-0.217	0.00	-238	-0.255	0.86	-447	-0.820	Neither	30
	HOH1027	3.90	219	0.814	1.15	-29	0.245	5.05	190	0.894	Protein A	4
	HOH1046	2.37	291	0.647	1.22	-176	-0.143	3.59	115	0.641	Protein A	30
	HOH1048	1.10	-416	-0.735	0.89	86	0.294	1.99	-330	-0.509	Protein B	0

	HOH1066	2.33	-69	0.285	1.04	-57	0.211	3.37	-127	-0.029	Protein A	13
	HOH1073	1.08	-51	0.223	0.00	-372	-0.608	1.08	-423	-0.755	Neither	31
	HOH1077	0.89	57	0.273	0.00	-230	-0.234	0.89	-173	-0.136	Protein A	3
	HOH1101	0.00	-29	-0.039	0.00	-335	-0.509	0.00	-364	-0.588	Neither	36
	HOH1111	1.18	-39	0.236	1.96	150	0.493	3.14	111	0.583	Protein B	24
	HOH1153	2.39	-2	0.375	1.11	-147	-0.078	3.50	-149	-0.080	Protein A	12
	HOH1154	0.97	-78	0.183	0.00	8	-0.039	0.97	-70	0.189	Neither	2
	HOH1158	0.00	-272	-0.343	0.70	-85	0.130	0.70	-357	-0.578	Neither	25
	HOH1165	1.39	453	0.633	2.60	134	0.553	3.98	587	0.936	Both	34
	HOH1166	0.97	25	0.264	0.00	-12	-0.039	0.97	13	0.255	Protein A	19
	HOH1177	0.96	4	0.245	1.92	-111	-0.001	2.88	-107	0.324	Neither	36
	HOH1186	0.79	-152	-0.089	0.00	-456	-0.839	0.79	-608	-1.289	Neither	0
	HOH1040	1.09	88	0.328	0.87	-194	-0.182	1.96	-106	0.152	Protein A	16
	HOH1055	2.15	251	0.597	0.00	-180	-0.117	2.15	71	0.420	Protein A	2
	HOH1072	1.25	79	0.351	1.06	-77	0.198	2.30	2	0.366	Protein A	49
	HOH108	0.00	-321	-0.471	3.19	-138	-0.055	3.19	-458	-0.850	Neither	32
	HOH116	3.80	268	0.809	1.35	113	0.397	5.15	382	0.950	Both	17
	HOH123	0.00	-213	-0.192	2.29	183	0.569	2.29	-30	0.325	Protein B	0
	HOH126	0.00	-143	-0.056	1.99	-38	0.255	1.99	-181	-0.154	Protein B	2
	HOH127	2.00	152	0.499	1.35	88	0.371	3.35	240	0.744	Both	20
	HOH131	0.00	-272	-0.343	3.39	-31	0.474	3.39	-303	-0.443	Protein B	8
	HOH135	0.00	-106	-0.040	5.02	-36	0.611	5.02	-142	-0.061	Protein B	12
	HOH144	0.00	-212	-0.189	0.91	119	0.314	0.91	-93	0.159	Protein B	40
	HOH149	1.26	1	0.276	4.13	321	0.857	5.39	322	0.942	Both	1
	HOH151	1.35	23	0.303	2.79	620	0.840	4.14	643	0.963	Both	17
	HOH153	0.00	-263	-0.318	3.24	-21	0.464	3.24	-283	-0.393	Protein B	0
	HOH155	2.31	87	0.461	1.27	85	0.359	3.58	171	0.729	Both	28
	HOH159	0.00	-353	-0.557	1.99	-74	0.202	1.99	-427	-0.763	Neither	6
	HOH168	0.00	-142	-0.054	3.71	653	0.928	3.71	511	0.889	Protein B	12
	HOH172	0.89	62	0.275	1.16	-190	-0.175	2.05	-129	-0.039	Protein A	20
	HOH173	1.00	-135	-0.051	2.05	53	0.385	3.05	-82	0.375	Protein B	19
	HOH174	0.93	64	0.284	1.30	52	0.330	2.22	116	0.483	Both	24
	HOH182	0.00	-645	-1.403	2.16	-9	0.327	2.16	-653	-1.431	Protein B	0
2Q4G	HOH13	0.00	-62	-0.039	2.02	13	0.327	2.02	-49	0.244	Protein B	0
	HOH23	1.08	-41	0.232	2.37	99	0.482	3.45	58	0.567	Protein B	1
	HOH43	2.09	39	0.373	1.38	185	0.443	3.47	224	0.757	Both	18
	HOH52	1.20	36	0.306	2.17	145	0.512	3.37	180	0.714	Both	10
	HOH54	0.85	65	0.271	0.94	2	0.240	1.78	67	0.380	Protein A	6
	HOH56	0.98	41	0.278	2.34	44	0.418	3.32	85	0.576	Both	1
	HOH66	1.13	-144	-0.072	2.53	129	0.538	3.66	-15	0.527	Protein B	32
	HOH69	0.81	-37	0.189	1.12	-64	0.216	1.93	-101	0.160	Neither	8
	HOH72	2.09	236	0.579	1.02	-135	-0.050	3.10	101	0.567	Protein A	3
	HOH73	2.41	49	0.435	1.31	114	0.392	3.72	163	0.736	Both	22
	HOH81	2.02	229	0.568	1.35	129	0.411	3.37	358	0.794	Both	0
	HOH96	0.00	157	-0.038	2.00	-635	-1.374	2.00	-478	-0.905	Neither	28
	HOH125	2.46	359	0.699	1.18	-123	-0.025	3.65	236	0.783	Protein A	38
	HOH135	3.80	450	0.875	1.17	-14	0.259	4.97	436	0.960	Both	14
	HOH151	2.04	-100	0.177	0.00	-188	-0.135	2.04	-289	-0.407	Neither	20
	HOH159	1.94	138	0.478	0.00	-222	-0.215	1.94	-84	0.185	Protein A	12
	HOH170	2.47	-205	-0.208	1.33	98	0.380	3.80	-108	0.462	Protein B	31
	HOH171	0.77	127	0.292	0.00	-172	-0.101	0.77	-45	0.175	Protein A	16
	HOH172	0.85	-201	-0.200	0.00	-134	-0.045	0.85	-335	-0.523	Neither	34
	HOH178	1.04	-16	0.244	0.00	-59	-0.039	1.04	-75	0.197	Neither	10
	HOH182	0.92	215	0.319	0.82	2	0.221	1.75	217	0.523	Protein A	2
	HOH184	1.07	239	0.368	0.00	-241	-0.262	1.07	-2	0.260	Protein A	37
	HOH186	0.85	271	0.342	0.00	-49	-0.039	0.85	222	0.308	Protein A	8
	HOH197	0.95	219	0.327	0.83	8	0.226	1.77	227	0.535	Protein A	28
	HOH208	0.86	77	0.282	0.00	-29	-0.039	0.86	48	0.261	Protein A	28
	HOH219	1.20	62	0.329	2.13	-236	-0.280	3.33	-174	-0.137	Protein A	0
	HOH226	1.43	-87	0.169	3.75	-39	0.520	5.18	-126	-0.025	Protein B	0

	HOH232	1.02	248	0.362	0.92	-141	-0.065	1.94	107	0.438	Protein A	18
	HOH268	0.93	31	0.262	0.00	-7	-0.039	0.93	25	0.257	Protein A	19
	HOH280	2.10	103	0.451	0.00	-115	-0.031	2.10	-12	0.313	Protein A	17
	HOH288	0.76	-219	-0.240	0.00	-168	-0.094	0.76	-387	-0.657	Neither	22
	HOH310	1.05	-16	0.246	0.92	-132	-0.045	1.97	-148	-0.082	Neither	27
	HOH333	2.03	37	0.360	1.11	25	0.287	3.13	62	0.533	Both	7
	HOH344	0.92	52	0.274	0.84	-275	-0.372	1.76	-222	-0.249	Protein A	16
	HOH360	2.51	386	0.718	1.22	5	0.278	3.73	390	0.846	Both	4
	HOH392	1.41	420	0.620	2.76	586	0.827	4.16	1006	0.996	Both	53
	HOH400	2.05	20	0.342	0.00	-93	-0.040	2.05	-73	0.218	Protein A	9
	HOH403	2.32	463	0.745	2.45	-56	0.322	4.77	407	0.937	Both	19
	HOH569	2.25	-110	0.002	1.16	186	0.378	3.41	76	0.578	Protein B	18
	HOH719	0.96	73	0.296	0.95	44	0.274	1.91	118	0.450	Both	18
2R25	HOH8	1.02	-582	-1.211	0.00	-406	-0.701	1.02	-988	-2.606	Neither	21
	HOH17	3.64	56	0.590	1.15	-135	-0.051	4.79	-79	0.558	Protein A	11
	HOH52	1.02	321	0.406	0.00	-142	-0.054	1.02	179	0.340	Protein A	1
	HOH59	1.11	82	0.329	0.95	-169	-0.125	2.06	-87	0.203	Protein A	19
	HOH81	2.23	-50	0.289	0.00	-86	-0.040	2.23	-136	-0.054	Protein A	0
	HOH102	2.13	254	0.597	0.00	-230	-0.235	2.13	23	0.361	Protein A	1
	HOH125	1.18	152	0.384	2.16	-641	-1.393	3.34	-488	-0.936	Protein A	1
	HOH159	0.00	-68	-0.039	2.27	880	0.859	2.27	812	0.853	Protein B	9
	HOH161	1.22	57	0.327	3.85	85	0.644	5.07	141	0.801	Both	10
	HOH163	0.00	-261	-0.313	0.91	-680	-1.517	0.91	-940	-2.426	Neither	2
	HOH164	2.34	-42	0.320	1.32	146	0.417	3.66	103	0.636	Both	35
	HOH171	1.03	-21	0.238	2.22	302	0.640	3.24	281	0.745	Protein B	33
	HOH172	0.00	-262	-0.316	2.26	-80	0.257	2.26	-342	-0.540	Protein B	11
	HOH176	0.00	-226	-0.225	0.99	-422	-0.750	0.99	-648	-1.416	Neither	1
	HOH177	0.99	-291	-0.411	0.90	53	0.271	1.89	-238	-0.285	Protein B	36
	HOH182	1.02	-40	0.222	1.09	294	0.406	2.11	254	0.595	Protein B	38
	HOH186	0.00	-77	-0.040	1.73	63	0.372	1.73	-15	0.274	Protein B	0
	HOH189	2.22	-179	-0.148	2.77	248	0.673	4.99	70	0.702	Protein B	39
	HOH190	0.90	8	0.239	1.16	334	0.460	2.06	342	0.661	Protein B	25
	HOH215	0.98	-78	0.184	1.10	231	0.369	2.07	153	0.509	Protein B	0
	HOH227	1.21	78	0.344	1.08	-365	-0.599	2.29	-287	-0.403	Protein A	11
2SIC	HOH49	2.55	428	0.744	0.92	-259	-0.335	3.47	168	0.710	Protein A	28
	HOH50	1.23	86	0.355	3.62	-325	-0.496	4.85	-239	-0.286	Protein A	9
	HOH54	3.34	-305	-0.447	1.14	-139	-0.059	4.48	-444	-0.811	Neither	7
	HOH55	3.42	-1930	-7.117	0.00	-120	-0.034	3.42	-2050	-7.817	Neither	23
	HOH59	0.91	-1	0.234	0.88	-128	-0.036	1.80	-129	-0.039	Neither	12
	HOH70	2.54	-174	-0.138	2.61	117	0.533	5.15	-57	0.597	Protein B	32
	HOH71	1.35	-359	-0.585	2.23	71	0.432	3.58	-289	-0.406	Protein B	14
	HOH146	0.88	63	0.274	0.00	-362	-0.581	0.88	-299	-0.433	Protein A	16
	HOH155	0.00	-66	-0.039	1.91	-323	-0.492	1.91	-389	-0.662	Neither	14
	HOH157	0.90	-242	-0.293	1.26	197	0.411	2.15	-44	0.281	Protein B	11
	HOH159	1.24	24	0.298	2.65	-97	0.304	3.89	-73	0.505	Both	2
	HOH160	1.05	-7	0.252	1.28	32	0.308	2.32	24	0.394	Both	0
	HOH172	1.01	-88	0.180	1.03	310	0.403	2.04	222	0.564	Protein B	3
	HOH173	1.09	-140	-0.061	2.40	189	0.590	3.49	50	0.565	Protein B	0
	HOH175	0.95	270	0.362	2.03	-147	-0.079	2.98	123	0.582	Protein A	22
	HOH176	0.87	-119	-0.016	1.02	135	0.341	1.90	16	0.323	Protein B	0
	HOH182	1.88	62	0.380	0.98	-63	0.197	2.86	-1	0.439	Protein A	10
2V9T	HOH2016	0.80	-159	-0.104	0.00	-316	-0.459	0.80	-475	-0.897	Neither	15
	HOH2017	3.71	266	0.797	1.40	182	0.447	5.11	448	0.973	Both	1
	HOH2018	2.40	275	0.640	0.00	-217	-0.201	2.40	58	0.442	Protein A	4
	HOH2019	4.14	358	0.871	1.33	148	0.419	5.47	506	1.010	Both	0
	HOH2027	2.51	-151	-0.086	1.15	16	0.284	3.66	-135	-0.047	Protein B	1
	HOH2028	1.09	-228	-0.260	0.90	21	0.248	1.99	-207	-0.213	Neither	12
	HOH2037	1.17	-448	-0.821	1.11	79	0.326	2.28	-369	-0.610	Protein B	35
	HOH2039	1.13	-368	-0.606	0.89	-387	-0.656	2.02	-754	-1.763	Neither	7
	HOH2040	0.90	-229	-0.263	0.90	79	0.290	1.80	-149	-0.084	Protein B	0

	HOH2041	3.83	-261	-0.340	1.12	36	0.297	4.95	-225	-0.255	Protein B	1
	HOH2044	1.22	151	0.396	1.00	-292	-0.415	2.23	-141	-0.065	Protein A	1
	HOH2091	1.21	-159	-0.106	0.85	-261	-0.339	2.05	-420	-0.747	Neither	20
	HOH2093	1.98	-44	0.242	0.00	16	-0.039	1.98	-29	0.264	Neither	12
	HOH2095	1.32	-321	-0.487	2.47	-81	0.295	3.79	-402	-0.697	Protein B	33
	HOH2025	0.00	-322	-0.475	0.72	-192	-0.178	0.72	-514	-1.009	Neither	7
	HOH2042	0.00	-56	-0.039	0.71	-5	0.195	0.71	-61	0.151	Neither	20
	HOH2045	1.12	-8	0.262	1.13	126	0.361	2.25	118	0.489	Both	0
	HOH2081	0.00	-188	-0.134	0.73	-208	-0.214	0.73	-395	-0.679	Neither	1
	HOH2084	0.00	-93	-0.040	0.00	-191	-0.142	0.00	-285	-0.376	Neither	3
	HOH2088	1.32	-56	0.213	2.28	451	0.737	3.60	395	0.834	Protein B	9
	HOH2092	0.00	-45	-0.039	2.26	290	0.636	2.26	245	0.605	Protein B	0
	HOH2118	0.00	-83	-0.040	0.99	157	0.336	0.99	75	0.302	Protein B	20
	HOH2119	1.29	-90	0.175	4.04	623	0.950	5.33	532	1.012	Protein B	1
	HOH2121	1.12	-64	0.216	3.93	256	0.820	5.05	191	0.897	Protein B	0
	HOH2123	1.08	-343	-0.542	1.09	83	0.325	2.17	-260	-0.337	Protein B	5
	HOH2147	0.00	-105	-0.040	2.11	-313	-0.466	2.11	-417	-0.739	Neither	6
	HOH2149	1.14	-392	-0.670	2.33	171	0.563	3.48	-221	-0.244	Protein B	1
	HOH2173	0.00	-184	-0.125	1.97	97	0.427	1.97	-87	0.180	Protein B	20
	HOH2197	0.00	-150	-0.064	1.11	63	0.315	1.11	-87	0.197	Protein B	18
	HOH2199	0.00	-20	-0.039	2.19	150	0.521	2.19	130	0.497	Protein B	29
	HOH2200	1.11	12	0.278	1.20	37	0.306	2.31	49	0.420	Both	24
	HOH2201	0.00	-213	-0.193	0.77	-54	0.167	0.77	-267	-0.355	Neither	22
	HOH2202	1.16	-64	0.214	1.23	206	0.403	2.39	143	0.538	Protein B	1
	HOH2234	0.99	-221	-0.246	2.58	11	0.416	3.56	-210	-0.219	Protein B	7
2VOL	HOH10	2.36	16	0.391	1.29	56	0.333	3.65	72	0.606	Both	30
	HOH11	2.36	-492	-0.947	1.01	12	0.260	3.37	-481	-0.913	Protein B	1
	HOH35	2.39	-188	-0.170	2.41	25	0.408	4.80	-163	-0.110	Protein B	10
	HOH94	2.39	-84	0.277	0.00	27	-0.038	2.39	-58	0.310	Protein A	6
	HOH97	1.04	-381	-0.641	0.97	15	0.255	2.01	-366	-0.601	Protein B	5
	HOH99	1.29	133	0.403	2.19	-235	-0.277	3.47	-102	0.412	Protein A	0
	HOH100	1.19	41	0.310	2.04	-60	0.234	3.23	-19	0.465	Protein A	5
	HOH104	2.68	526	0.799	2.33	9	0.378	5.01	535	0.996	Both	5
	HOH150	1.03	-208	-0.214	1.21	138	0.389	2.25	-70	0.267	Protein B	30
	HOH151	1.48	-43	0.227	2.68	211	0.646	4.15	169	0.790	Protein B	9
	HOH152	0.00	-77	-0.040	0.88	-80	0.166	0.88	-157	-0.099	Neither	2
	HOH168	0.96	-231	-0.269	1.19	131	0.378	2.14	-101	0.204	Protein B	0
	HOH169	0.99	57	0.290	1.27	-146	-0.077	2.26	-89	0.246	Protein A	29
	HOH170	1.18	98	0.355	2.12	-20	0.306	3.30	78	0.567	Both	28
	HOH171	1.27	101	0.374	2.58	97	0.505	3.85	198	0.803	Both	12
	HOH176	1.07	34	0.287	3.64	226	0.781	4.72	260	0.888	Both	19
	HOH179	1.22	-78	0.196	2.25	69	0.433	3.47	-8	0.506	Protein B	32
	HOH180	0.00	-195	-0.150	2.03	85	0.421	2.03	-110	0.002	Protein B	11
	HOH186	0.00	-47	-0.039	3.53	246	0.769	3.53	199	0.759	Protein B	14
	HOH218	0.92	-100	0.156	1.16	144	0.375	2.08	45	0.379	Protein B	23
	HOH223	0.92	-123	-0.024	1.05	-204	-0.207	1.97	-327	-0.502	Neither	0
2VSM	HOH23	2.24	-71	0.263	0.00	-16	-0.039	2.24	-87	0.243	Protein A	30
	HOH24	3.99	284	0.832	1.11	25	0.287	5.10	308	0.923	Both	47
	HOH47	1.30	-263	-0.343	2.50	433	0.742	3.80	170	0.759	Protein B	0
	HOH48	2.17	-73	0.248	0.99	19	0.262	3.17	-54	0.420	Protein B	2
	HOH49	1.38	-37	0.233	2.39	347	0.685	3.77	310	0.818	Protein B	10
	HOH51	0.90	149	0.318	0.00	-350	-0.549	0.90	-201	-0.198	Protein A	45
	HOH134	2.37	-8	0.365	1.00	-22	0.233	3.37	-30	0.472	Protein A	36
	HOH173	0.75	28	0.227	0.00	-173	-0.103	0.75	-145	-0.073	Neither	3
	HOH242	0.73	-276	-0.376	0.00	-232	-0.240	0.73	-508	-0.992	Neither	48
	HOH243	0.71	-66	0.147	0.00	-236	-0.250	0.71	-303	-0.441	Neither	3
	HOH278	1.20	-15	0.259	0.85	55	0.265	2.05	41	0.370	Both	20
	HOH279	3.38	336	0.785	0.00	-32	-0.039	3.38	304	0.770	Protein A	13
	HOH281	0.00	-136	-0.048	0.71	-16	0.187	0.71	-152	-0.089	Neither	6
	HOH289	1.37	21	0.301	2.08	-108	0.177	3.45	-88	0.423	Protein A	2

	HOH290	1.34	107	0.390	2.27	-48	0.299	3.60	59	0.588	Both	20
	HOH378	1.21	178	0.396	0.89	-143	-0.070	2.10	35	0.370	Protein A	10
	HOH380	2.34	300	0.650	0.00	-53	-0.039	2.34	246	0.616	Protein A	13
	HOH381	2.12	-87	0.217	1.24	-273	-0.369	3.36	-360	-0.587	Neither	2
	HOH382	3.73	-43	0.512	1.20	-13	0.261	4.93	-56	0.587	Both	16
	HOH383	2.30	87	0.459	0.00	-239	-0.257	2.30	-152	-0.090	Protein A	12
	HOH384	2.35	103	0.484	0.99	-28	0.227	3.34	75	0.570	Protein A	5
	HOH385	1.14	176	0.371	0.98	-307	-0.452	2.12	-131	-0.044	Protein A	0
	HOH396	2.11	478	0.748	0.00	-50	-0.039	2.11	428	0.719	Protein A	2
	HOH397	2.42	442	0.741	0.00	-32	-0.039	2.42	410	0.724	Protein A	6
	HOH398	1.06	3	0.262	0.00	-215	-0.196	1.06	-212	-0.224	Protein A	6
	HOH400	1.43	129	0.421	3.61	-44	0.493	5.04	85	0.717	Both	22
	HOH401	1.19	102	0.360	1.13	-129	-0.037	2.31	-27	0.333	Protein A	13
	HOH404	1.01	-90	0.179	0.00	-276	-0.352	1.01	-366	-0.602	Neither	11
	HOH425	2.64	120	0.539	1.35	-20	0.254	3.99	100	0.666	Both	11
	HOH427	1.18	401	0.507	1.02	-48	0.215	2.20	353	0.673	Protein A	11
	HOH454	2.40	15	0.396	1.03	14	0.265	3.43	29	0.537	Both	0
	HOH459	2.31	186	0.575	2.36	14	0.387	4.67	200	0.881	Both	5
	HOH460	1.08	-33	0.238	0.86	56	0.267	1.94	23	0.333	Protein B	11
	HOH475	3.82	-448	-0.822	1.03	13	0.265	4.86	-435	-0.786	Protein B	34
	HOH477	1.14	133	0.364	0.00	-379	-0.628	1.14	-247	-0.306	Protein A	0
	HOH479	1.37	151	0.428	2.37	21	0.397	3.73	173	0.752	Both	16
	HOH480	3.87	15	0.585	1.14	-356	-0.577	5.02	-341	-0.539	Protein A	1
	HOH483	1.91	-121	-0.022	0.00	-211	-0.187	1.91	-332	-0.514	Neither	56
	HOH536	0.00	-208	-0.180	1.04	17	0.270	1.04	-191	-0.176	Protein B	44
	HOH538	1.02	-164	-0.115	1.11	15	0.280	2.13	-149	-0.082	Protein B	35
	HOH578	0.00	-202	-0.166	1.90	31	0.342	1.90	-171	-0.133	Protein B	10
	HOH582	1.19	-117	-0.012	2.40	-6	0.371	3.58	-123	-0.020	Protein B	4
	HOH586	0.97	82	0.304	1.25	-216	-0.234	2.22	-134	-0.050	Protein A	2
	HOH589	0.89	-23	0.213	1.14	-161	-0.108	2.02	-183	-0.160	Neither	25
	HOH591	0.98	-57	0.202	2.57	165	0.590	3.55	108	0.627	Protein B	24
	HOH601	0.00	-233	-0.242	1.04	339	0.419	1.04	105	0.332	Protein B	28
	HOH602	1.17	-247	-0.306	1.22	503	0.571	2.39	256	0.627	Protein B	8
	HOH603	1.25	-196	-0.187	2.45	-46	0.334	3.70	-241	-0.292	Protein B	2
	HOH605	0.00	-38	-0.039	2.30	65	0.435	2.30	27	0.393	Protein B	11
	HOH608	0.87	-185	-0.162	1.20	-5	0.268	2.07	-190	-0.175	Protein B	22
	HOH609	0.00	-317	-0.461	1.99	32	0.347	1.99	-285	-0.398	Protein B	24
	HOH610	0.00	-221	-0.212	2.07	85	0.427	2.07	-136	-0.054	Protein B	34
	HOH611	1.27	-86	0.182	3.60	123	0.654	4.87	37	0.669	Protein B	12
	HOH615	0.96	-87	0.174	2.45	426	0.735	3.41	339	0.790	Protein B	27
2VXT	HOH60	3.03	488	0.817	2.64	-95	0.304	5.67	392	0.978	Both	21
	HOH61	0.85	250	0.328	0.00	-200	-0.162	0.85	50	0.261	Protein A	10
	HOH62	1.16	471	0.524	1.09	-297	-0.427	2.25	174	0.555	Protein A	16
	HOH63	3.77	-62	0.501	1.46	-70	0.191	5.23	-131	-0.037	Protein A	31
	HOH78	2.41	505	0.769	1.30	-227	-0.258	3.71	279	0.800	Protein A	24
	HOH89	4.12	369	0.874	1.14	-32	0.242	5.26	337	0.940	Protein A	0
	HOH90	2.46	-46	0.335	0.00	-79	-0.040	2.46	-125	-0.028	Protein A	14
	HOH228	0.00	-106	-0.040	0.00	-72	-0.040	0.00	-178	-0.112	Neither	17
	HOH229	0.00	-266	-0.326	0.74	-26	0.185	0.74	-291	-0.413	Neither	55
	HOH231	1.20	-13	0.261	3.49	568	0.888	4.68	556	0.982	Both	0
	HOH292	1.16	58	0.320	1.20	159	0.391	2.36	217	0.602	Both	0
	HOH293	0.95	128	0.326	1.09	10	0.273	2.04	138	0.487	Both	30
	HOH301	1.13	-15	0.257	1.29	392	0.558	2.42	377	0.705	Both	41
	HOH308	0.00	-265	-0.324	0.92	-45	0.201	0.92	-310	-0.459	Neither	3
	HOH310	2.02	-106	0.164	1.42	602	0.708	3.44	496	0.859	Protein B	0
	HOH311	0.89	-28	0.209	1.12	448	0.488	2.01	420	0.716	Protein B	18
	HOH324	0.00	-191	-0.141	2.35	17	0.390	2.35	-174	-0.138	Protein B	55
	HOH326	1.38	39	0.322	2.40	337	0.679	3.78	376	0.845	Both	0
	HOH328	0.98	67	0.296	1.26	374	0.535	2.25	441	0.729	Both	0
	HOH329	0.92	25	0.255	1.18	552	0.563	2.10	577	0.795	Both	0

	HOH330	1.19	2	0.274	1.19	268	0.433	2.38	270	0.635	Both	0
	HOH332	1.16	2	0.272	1.18	66	0.329	2.33	67	0.443	Both	0
	HOH335	0.98	226	0.339	0.99	-115	-0.007	1.96	112	0.446	Protein A	37
	HOH387	1.11	408	0.462	0.00	-86	-0.040	1.11	322	0.424	Protein A	38
	HOH394	0.97	-357	-0.578	0.00	-227	-0.226	0.97	-583	-1.215	Neither	4
	HOH396	0.85	-301	-0.436	0.79	-43	0.180	1.64	-344	-0.544	Neither	4
	HOH397	1.08	-166	-0.120	1.07	293	0.401	2.15	127	0.488	Protein B	7
	HOH398	2.73	78	0.503	2.68	459	0.772	5.41	537	1.017	Both	34
	HOH399	2.60	106	0.517	2.37	72	0.454	4.97	179	0.870	Both	36
	HOH462	1.00	47	0.284	0.00	-132	-0.043	1.00	-85	0.181	Protein A	0
	HOH465	1.17	194	0.383	1.14	30	0.294	2.31	224	0.599	Both	0
	HOH467	2.39	380	0.704	0.95	-415	-0.733	3.34	-36	0.461	Protein A	2
	HOH468	2.67	93	0.511	1.17	-276	-0.376	3.84	-183	-0.156	Protein A	19
2WEL	HOH283	2.30	-204	-0.205	1.25	-385	-0.652	3.54	-589	-1.231	Neither	0
	HOH285	2.26	78	0.444	1.00	-381	-0.642	3.26	-304	-0.444	Protein A	10
	HOH286	3.63	-77	0.463	0.00	-540	-1.082	3.63	-617	-1.317	Protein A	4
	HOH287	2.70	43	0.465	1.32	-47	0.223	4.02	-4	0.580	Protein A	0
	HOH288	3.95	-28	0.552	1.10	-258	-0.331	5.05	-286	-0.400	Protein A	3
	HOH299	1.07	-131	-0.042	2.69	645	0.839	3.76	514	0.894	Protein B	10
	HOH300	0.89	23	0.248	1.24	-134	-0.050	2.13	-111	0.000	Neither	23
	HOH305	0.00	-77	-0.040	0.94	277	0.364	0.94	201	0.317	Protein B	25
	HOH307	0.00	-75	-0.040	3.67	193	0.771	3.67	118	0.655	Protein B	23
	HOH354	1.18	-114	-0.005	1.19	337	0.479	2.37	224	0.607	Protein B	14
	HOH385	0.00	-44	-0.039	1.10	385	0.452	1.10	341	0.432	Protein B	49
	HOH390	1.24	-33	0.241	1.05	246	0.369	2.29	213	0.590	Protein B	26
	HOH392	2.07	-199	-0.196	1.18	360	0.487	3.25	160	0.669	Protein B	32
	HOH405	2.60	272	0.663	1.34	22	0.301	3.94	294	0.830	Both	25
2WWX	HOH2001	1.15	76	0.331	1.13	62	0.318	2.28	138	0.518	Both	50
	HOH2008	0.00	-148	-0.061	0.00	-97	-0.040	0.00	-245	-0.272	Neither	30
	HOH2015	2.88	229	0.680	2.51	-65	0.320	5.39	164	0.865	Both	0
	HOH2016	1.27	6	0.281	2.20	-199	-0.195	3.46	-193	-0.180	Protein A	23
	HOH2018	1.36	249	0.481	3.68	-36	0.511	5.05	212	0.915	Both	2
	HOH2019	2.24	-102	0.223	1.27	76	0.351	3.51	-26	0.494	Protein B	0
	HOH2020	2.48	329	0.682	2.41	-224	-0.251	4.89	105	0.729	Protein A	0
	HOH2026	1.87	-406	-0.708	1.06	30	0.282	2.93	-376	-0.628	Protein B	6
	HOH2028	3.27	350	0.780	0.00	-36	-0.039	3.27	314	0.763	Protein A	1
	HOH2029	4.10	24	0.610	1.37	-6	0.270	5.47	18	0.678	Both	20
	HOH2031	1.28	-194	-0.184	2.27	401	0.708	3.55	207	0.769	Protein B	0
	HOH2032	2.43	38	0.425	1.03	-132	-0.045	3.46	-94	0.417	Protein A	8
	HOH2025	1.00	-171	-0.132	2.35	-119	-0.016	3.35	-290	-0.410	Neither	13
	HOH2034	2.60	-386	-0.655	2.62	178	0.611	5.22	-208	-0.214	Protein B	0
	HOH2035	2.73	-147	-0.078	2.78	-4	0.426	5.51	-151	-0.083	Protein B	0
	HOH2037	2.30	109	0.483	1.39	545	0.676	3.69	654	0.927	Both	16
	HOH2038	1.13	67	0.321	1.18	-222	-0.248	2.30	-155	-0.095	Protein A	29
	HOH2039	0.00	-108	-0.040	3.50	278	0.774	3.50	171	0.717	Protein B	10
	HOH2041	1.20	148	0.387	1.21	-310	-0.459	2.40	-162	-0.111	Protein A	2
	HOH2045	1.28	-42	0.231	2.39	5	0.383	3.67	-36	0.509	Protein B	4
	HOH2046	1.34	-41	0.229	3.90	-8	0.567	5.24	-49	0.608	Protein B	7
2WY3	HOH10	0.00	-177	-0.111	0.00	-148	-0.062	0.00	-325	-0.483	Neither	45
	HOH20	0.00	-160	-0.080	0.00	-226	-0.224	0.00	-386	-0.647	Neither	32
	HOH74	0.95	245	0.346	0.00	-219	-0.207	0.95	26	0.261	Protein A	3
	HOH75	0.82	394	0.397	0.79	-41	0.181	1.61	352	0.619	Protein A	16
	HOH76	2.74	605	0.832	1.35	-68	0.197	4.08	537	0.931	Protein A	29
	HOH77	0.92	349	0.399	0.00	-63	-0.039	0.92	286	0.366	Protein A	0
	HOH80	0.90	106	0.308	0.83	-233	-0.272	1.73	-127	-0.034	Protein A	10
	HOH83	0.96	307	0.386	0.00	-315	-0.457	0.96	-9	0.236	Protein A	15
	HOH90	3.33	527	0.860	0.00	-238	-0.254	3.33	289	0.758	Protein A	43
	HOH91	2.51	725	0.845	0.00	-97	-0.040	2.51	629	0.821	Protein A	18
	HOH105	2.56	244	0.643	1.08	-41	0.231	3.64	203	0.777	Protein A	8
	HOH126	0.82	-151	-0.088	0.00	-128	-0.040	0.82	-279	-0.383	Neither	0

	HOH127	2.20	149	0.520	0.00	-174	-0.106	2.20	-26	0.313	Protein A	41
	HOH155	1.03	-173	-0.136	0.00	-174	-0.104	1.03	-347	-0.552	Neither	56
	HOH156	1.01	-174	-0.138	0.00	-289	-0.387	1.01	-463	-0.863	Neither	26
	HOH158	1.13	254	0.398	1.13	-286	-0.399	2.26	-31	0.319	Protein A	1
	HOH160	1.19	518	0.557	1.15	-233	-0.273	2.34	284	0.640	Protein A	0
	HOH161	1.17	141	0.379	0.88	-38	0.199	2.05	103	0.444	Protein A	47
	HOH164	1.19	25	0.295	1.07	-175	-0.140	2.26	-150	-0.085	Protein A	26
	HOH165	1.17	162	0.383	0.86	-163	-0.114	2.03	-2	0.311	Protein A	30
	HOH166	3.78	169	0.753	0.00	-323	-0.477	3.78	-154	-0.090	Protein A	0
	HOH169	1.06	94	0.328	0.89	-46	0.194	1.95	48	0.365	Protein A	6
	HOH176	0.74	52	0.243	0.00	-35	-0.039	0.74	17	0.218	Neither	2
	HOH225	1.16	142	0.376	1.19	-28	0.247	2.35	115	0.497	Protein A	28
	HOH268	0.00	-11	-0.039	3.98	328	0.846	3.98	317	0.842	Protein B	36
	HOH292	0.00	25	-0.038	1.83	78	0.397	1.83	103	0.427	Protein B	22
	HOH296	0.00	-52	-0.039	2.20	158	0.531	2.20	106	0.467	Protein B	6
	HOH297	0.00	-72	-0.040	0.82	-57	0.174	0.82	-129	-0.038	Neither	42
	HOH301	1.15	-224	-0.253	1.22	-399	-0.688	2.37	-623	-1.336	Neither	12
	HOH305	0.00	-395	-0.671	1.03	-257	-0.330	1.03	-652	-1.428	Neither	10
	HOH323	1.38	94	0.381	4.19	632	0.965	5.56	727	1.067	Both	3
	HOH330	0.00	-77	-0.040	3.47	23	0.537	3.47	-55	0.461	Protein B	8
2XG5	HOH3	1.28	120	0.391	0.90	-371	-0.615	2.17	-251	-0.315	Protein A	2
	HOH4	2.03	-40	0.261	0.94	-201	-0.198	2.98	-240	-0.290	Protein A	10
	HOH7	2.70	-52	0.362	1.31	55	0.334	4.00	3	0.584	Both	0
	HOH8	1.10	-320	-0.483	0.95	-148	-0.079	2.05	-467	-0.876	Neither	28
	HOH20	0.92	-55	0.192	0.90	43	0.265	1.82	-12	0.282	Protein B	0
	HOH21	0.00	-256	-0.300	0.00	-172	-0.100	0.00	-427	-0.759	Neither	2
	HOH22	0.00	-213	-0.191	0.00	-196	-0.151	0.00	-408	-0.708	Neither	30
	HOH74	2.28	-21	0.334	0.00	-67	-0.039	2.28	-88	0.251	Protein A	24
	HOH78	1.89	-122	-0.024	0.00	-161	-0.082	1.89	-283	-0.392	Neither	0
	HOH79	1.19	97	0.356	1.13	156	0.369	2.32	253	0.617	Both	0
	HOH105	2.45	-47	0.332	1.23	243	0.432	3.68	196	0.776	Both	5
	HOH106	1.06	32	0.285	1.05	21	0.274	2.11	53	0.394	Both	0
	HOH124	2.19	83	0.439	0.00	-234	-0.245	2.19	-152	-0.089	Protein A	13
	HOH126	1.24	-97	0.172	0.96	190	0.324	2.20	93	0.452	Protein B	34
	HOH127	1.99	-259	-0.335	0.00	-123	-0.036	1.99	-382	-0.645	Neither	6
	HOH128	1.38	237	0.476	3.62	-110	0.010	5.00	127	0.770	Protein A	10
	HOH143	0.00	-124	-0.037	1.06	340	0.424	1.06	215	0.352	Protein B	0
	HOH144	0.93	-10	0.230	1.16	-48	0.229	2.09	-57	0.250	Neither	0
	HOH147	3.75	106	0.651	1.52	-106	0.142	5.27	0	0.655	Protein A	0
	HOH148	2.53	-344	-0.546	1.31	-145	-0.073	3.84	-489	-0.937	Neither	16
	HOH149	0.00	-165	-0.089	2.33	-151	-0.086	2.33	-316	-0.473	Neither	15
	HOH150	2.90	-163	-0.112	2.91	17	0.463	5.81	-146	-0.070	Protein B	0
	HOH151	0.91	-124	-0.028	0.92	-123	-0.025	1.82	-247	-0.307	Neither	35
	HOH158	0.97	-143	-0.068	1.30	71	0.349	2.27	-72	0.269	Protein B	25
	HOH159	2.12	-151	-0.087	1.32	-45	0.226	3.44	-196	-0.186	Neither	34
	HOH166	1.13	-119	-0.016	2.64	58	0.473	3.77	-61	0.502	Protein B	35
	HOH169	2.89	39	0.484	2.84	89	0.527	5.74	128	0.802	Both	12
	HOH197	1.34	-366	-0.601	2.60	-266	-0.352	3.94	-632	-1.364	Neither	0
	HOH198	2.22	-35	0.307	1.35	56	0.339	3.58	21	0.549	Both	19
	HOH199	0.96	-279	-0.383	0.91	499	0.451	1.87	220	0.543	Protein B	0
	HOH200	1.04	-183	-0.157	2.24	-169	-0.127	3.28	-351	-0.564	Neither	6
	HOH201	1.02	-120	-0.017	2.09	433	0.722	3.11	313	0.744	Protein B	0
2XGY	HOH2040	0.00	-148	-0.062	0.00	-53	-0.039	0.00	-201	-0.163	Neither	40
	HOH2045	1.04	-126	-0.032	1.97	-253	-0.320	3.01	-379	-0.636	Neither	0
	HOH2048	0.00	-145	-0.057	0.71	-230	-0.266	0.71	-374	-0.624	Neither	10
	HOH2087	0.80	-30	0.191	0.00	-83	-0.040	0.80	-113	-0.004	Neither	9
	HOH2089	2.49	-196	-0.188	1.28	-41	0.231	3.77	-237	-0.282	Neither	57
	HOH2090	1.12	-134	-0.048	0.92	-77	0.175	2.04	-210	-0.221	Neither	38
	HOH2092	0.94	-163	-0.113	0.00	-230	-0.233	0.94	-393	-0.672	Neither	0
	HOH2094	1.21	74	0.341	0.92	-75	0.177	2.13	0	0.332	Protein A	16

	HOH2099	0.77	-237	-0.282	0.00	-70	-0.039	0.77	-307	-0.451	Neither	27
	HOH2100	2.09	-139	-0.062	0.98	-32	0.222	3.07	-171	-0.131	Neither	49
	HOH2104	2.62	-113	-0.002	0.99	-47	0.212	3.61	-160	-0.106	Neither	9
	HOH2105	2.23	-2	0.350	1.06	30	0.282	3.29	28	0.519	Both	0
	HOH2115	1.19	-212	-0.223	1.12	363	0.451	2.31	152	0.538	Protein B	30
	HOH2116	2.80	187	0.646	2.62	21	0.433	5.43	209	0.939	Both	8
	HOH2052	0.00	-324	-0.480	0.00	0	-0.039	0.00	-324	-0.480	Neither	6
	HOH2074	0.00	-49	-0.039	0.72	-11	0.193	0.72	-60	0.154	Neither	27
	HOH2096	0.87	-174	-0.137	0.96	28	0.263	1.83	-146	-0.076	Protein B	28
	HOH2098	1.17	-252	-0.319	2.27	-72	0.270	3.44	-324	-0.495	Protein B	0
	HOH2128	0.00	-329	-0.494	1.04	120	0.339	1.04	-209	-0.218	Protein B	24
	HOH2163	1.11	-289	-0.408	2.04	146	0.497	3.15	-143	-0.067	Protein B	9
	HOH2176	0.00	170	-0.038	4.88	-62	0.580	4.88	108	0.733	Protein B	4
	HOH2177	1.26	209	0.416	2.39	506	0.768	3.65	715	0.936	Both	28
	HOH2179	2.53	-194	-0.182	1.35	306	0.524	3.87	113	0.676	Protein B	0
	HOH2180	0.00	-171	-0.099	1.00	268	0.372	1.00	97	0.320	Protein B	22
	HOH2186	1.12	-184	-0.161	1.18	-81	0.196	2.31	-265	-0.349	Neither	7
	HOH2210	0.00	-180	-0.116	0.84	27	0.242	0.84	-152	-0.089	Neither	34
	HOH2212	1.05	-270	-0.361	1.16	-226	-0.257	2.21	-496	-0.958	Neither	0
	HOH2213	0.00	-192	-0.142	2.30	124	0.503	2.30	-68	0.280	Protein B	1
	HOH2229	0.93	-38	0.207	2.50	34	0.430	3.43	-5	0.504	Protein B	5
	HOH2230	1.16	129	0.371	0.83	-3	0.218	2.00	125	0.466	Protein A	37
2XNA	HOH9	2.41	-71	0.297	1.08	-131	-0.041	3.49	-202	-0.200	Protein A	10
	HOH23	1.48	25	0.311	3.96	70	0.639	5.43	95	0.743	Both	0
	HOH24	2.59	51	0.459	1.05	-20	0.244	3.64	31	0.568	Protein A	1
	HOH25	1.26	227	0.430	2.06	-24	0.288	3.32	203	0.734	Both	1
	HOH30	1.45	80	0.373	3.81	-126	-0.027	5.26	-46	0.612	Protein A	0
	HOH32	0.00	-148	-0.062	0.76	-12	0.198	0.76	-160	-0.107	Neither	8
	HOH175	1.08	-54	0.221	1.99	80	0.408	3.07	26	0.491	Protein B	13
	HOH225	1.37	30	0.311	4.11	378	0.877	5.48	408	0.976	Both	3
	HOH226	1.54	-182	-0.156	4.27	556	0.952	5.81	374	0.976	Protein B	41
	HOH227	2.49	-221	-0.245	2.96	614	0.854	5.45	394	0.969	Protein B	3
2XQY	HOH210	1.08	-168	-0.124	0.84	9	0.229	1.93	-160	-0.107	Neither	14
	HOH214	2.94	175	0.650	3.02	249	0.706	5.96	424	0.999	Both	0
	HOH223	1.16	48	0.312	0.83	-230	-0.267	1.99	-183	-0.158	Protein A	21
	HOH224	1.08	-394	-0.675	0.00	-67	-0.039	1.08	-461	-0.859	Neither	6
	HOH225	0.00	332	-0.038	3.04	-238	-0.285	3.04	94	0.552	Neither	3
	HOH226	1.30	10	0.287	2.32	-30	0.331	3.62	-19	0.518	Both	4
	HOH235	1.14	461	0.507	1.07	-39	0.232	2.21	422	0.718	Protein A	17
	HOH236	2.66	232	0.651	2.44	60	0.450	5.09	292	0.918	Both	16
	HOH459	0.00	-163	-0.085	0.96	-85	0.175	0.96	-248	-0.307	Neither	13
	HOH464	0.00	-238	-0.254	5.77	112	0.775	5.77	-126	-0.024	Protein B	10
	HOH465	0.00	-411	-0.714	2.16	42	0.389	2.16	-368	-0.608	Protein B	0
	HOH466	2.57	476	0.769	1.31	-4	0.272	3.88	472	0.891	Both	15
	HOH480	1.18	-73	0.204	2.25	-23	0.326	3.43	-97	0.410	Protein B	6
	HOH481	0.00	-89	-0.040	0.80	-24	0.197	0.80	-113	-0.004	Neither	40
	HOH482	2.10	-114	-0.007	1.31	317	0.520	3.42	203	0.747	Protein B	21
	HOH523	0.00	-80	-0.040	2.08	-14	0.306	2.08	-94	0.198	Protein B	0
	HOH524	0.00	-265	-0.325	2.05	106	0.447	2.05	-159	-0.106	Protein B	7
	HOH529	1.00	-315	-0.471	1.08	333	0.423	2.08	18	0.346	Protein B	5
	HOH531	0.00	-232	-0.238	2.29	16	0.379	2.29	-216	-0.233	Protein B	9
	HOH532	0.00	-391	-0.660	2.29	-193	-0.180	2.29	-584	-1.216	Neither	7
	HOH637	0.86	24	0.244	0.93	35	0.264	1.79	59	0.372	Protein B	35
	HOH650	2.79	33	0.465	2.74	-1	0.424	5.53	32	0.693	Both	0
	HOH654	0.00	211	-0.039	1.94	63	0.384	1.94	273	0.597	Protein B	50
	HOH655	2.49	3	0.395	1.27	161	0.411	3.76	164	0.742	Both	10
	HOH656	2.34	-11	0.356	1.29	17	0.294	3.62	6	0.543	Both	8
	HOH657	1.04	-208	-0.215	2.36	190	0.584	3.40	-18	0.487	Protein B	3
	HOH658	1.10	100	0.338	2.09	-188	-0.169	3.19	-88	0.386	Protein A	17
2YVJ	HOH501	0.00	20	-0.039	1.22	-110	0.003	1.22	-90	0.182	Neither	23

2Z0D	HOH355	4.09	-461	-0.858	1.12	-1	0.268	5.21	-462	-0.862	Protein B	0
	HOH358	2.19	58	0.412	1.11	-252	-0.317	3.30	-194	-0.181	Protein A	3
	HOH361	2.53	-125	-0.030	1.04	-111	0.002	3.57	-237	-0.281	Neither	29
	HOH369	2.42	-28	0.350	1.33	334	0.538	3.75	307	0.815	Both	32
	HOH374	4.07	124	0.706	0.00	24	-0.038	4.07	148	0.747	Protein A	13
	HOH375	1.19	178	0.390	1.05	-54	0.217	2.24	124	0.496	Protein A	1
	HOH380	1.13	-53	0.225	0.90	56	0.274	2.03	3	0.318	Protein B	0
	HOH383	3.64	101	0.630	0.00	-249	-0.284	3.64	-149	-0.079	Protein A	0
	HOH389	2.41	147	0.547	0.99	-65	0.197	3.40	82	0.583	Protein A	20
	HOH390	3.84	276	0.816	0.00	-165	-0.088	3.84	111	0.670	Protein A	0
	HOH393	1.24	268	0.455	2.20	-201	-0.199	3.44	67	0.574	Protein A	22
	HOH403	3.38	-510	-0.997	0.00	-55	-0.039	3.38	-564	-1.157	Neither	13
	HOH406	1.23	349	0.504	1.15	-302	-0.439	2.38	47	0.428	Protein A	0
	HOH412	0.88	159	0.313	0.89	-163	-0.114	1.76	-4	0.289	Protein A	4
	HOH426	2.31	498	0.761	1.11	-50	0.228	3.41	448	0.838	Protein A	0
	HOH428	2.33	104	0.481	1.02	28	0.274	3.35	132	0.636	Both	2
	HOH444	0.83	16	0.233	0.00	50	-0.038	0.83	66	0.269	Neither	33
	HOH450	1.10	435	0.471	0.83	-198	-0.193	1.93	236	0.564	Protein A	2
	HOH459	2.31	148	0.535	0.00	-340	-0.522	2.31	-191	-0.177	Protein A	30
	HOH514	0.71	131	0.281	0.00	-175	-0.107	0.71	-43	0.165	Protein A	29
	HOH523	1.44	174	0.452	3.57	-14	0.516	5.01	160	0.836	Both	20
	HOH539	0.76	18	0.221	0.00	8	-0.039	0.76	26	0.227	Neither	7
	HOH552	2.14	-61	0.256	1.03	-92	0.181	3.17	-153	-0.090	Protein A	0
	HOH559	1.15	5	0.275	0.90	42	0.264	2.05	47	0.377	Both	5
	HOH122	4.13	-250	-0.312	1.47	69	0.361	5.59	-181	-0.151	Protein B	2
	HOH125	2.21	-242	-0.294	1.29	24	0.301	3.50	-218	-0.237	Protein B	2
	HOH132	1.17	-152	-0.089	1.21	-7	0.267	2.38	-159	-0.105	Protein B	1
	HOH133	0.00	-36	-0.039	0.73	-198	-0.191	0.73	-234	-0.275	Neither	2
	HOH141	1.22	17	0.290	2.56	420	0.741	3.78	436	0.869	Both	7
	HOH147	0.79	65	0.261	0.00	-585	-1.218	0.79	-520	-1.025	Protein A	4
	HOH149	0.00	54	-0.038	2.21	-367	-0.605	2.21	-313	-0.467	Neither	8
	HOH150	1.42	-73	0.188	3.85	59	0.623	5.27	-14	0.642	Protein B	9
	HOH152	0.00	-132	-0.043	1.85	-73	0.199	1.85	-205	-0.208	Neither	2
	HOH158	1.06	-20	0.244	1.24	-139	-0.060	2.29	-159	-0.104	Neither	0
	HOH179	0.00	-341	-0.525	1.08	212	0.353	1.08	-129	-0.037	Protein B	0
	HOH190	1.19	64	0.329	3.66	202	0.780	4.85	266	0.898	Both	21
	HOH195	1.33	4	0.281	2.44	-302	-0.440	3.77	-298	-0.429	Protein A	18
	HOH199	0.93	-43	0.205	2.07	108	0.453	3.00	65	0.521	Protein B	17
	HOH200	0.93	-122	-0.023	2.06	0	0.320	3.00	-123	-0.022	Protein B	44
	HOH202	2.24	-14	0.336	1.35	-123	-0.025	3.59	-137	-0.051	Protein A	18
	HOH203	0.00	-17	-0.039	0.97	161	0.332	0.97	145	0.332	Protein B	0
	HOH204	3.64	187	0.759	1.42	-20	0.255	5.06	167	0.854	Both	34
	HOH205	0.00	-211	-0.188	0.72	-26	0.181	0.72	-238	-0.284	Neither	2
	HOH211	0.00	-231	-0.236	0.00	-303	-0.425	0.00	-534	-1.065	Neither	36
	HOH212	0.00	-177	-0.111	0.76	-226	-0.256	0.76	-402	-0.698	Neither	4
	HOH217	0.76	-8	0.202	0.00	-806	-1.940	0.76	-814	-1.967	Neither	23
2Z3Q	HOH115	1.40	-38	0.232	3.52	-68	0.454	4.92	-106	0.538	Protein B	9
	HOH116	1.25	-323	-0.492	2.11	13	0.345	3.36	-310	-0.459	Protein B	1
	HOH122	2.06	562	0.791	1.37	150	0.428	3.43	711	0.918	Both	2
	HOH131	2.49	-24	0.366	2.41	109	0.498	4.90	85	0.711	Both	27
	HOH135	1.07	26	0.281	0.85	-14	0.212	1.92	11	0.317	Protein A	6
	HOH136	0.97	211	0.327	0.00	-158	-0.076	0.97	53	0.284	Protein A	0
	HOH139	0.98	441	0.449	0.00	-388	-0.651	0.98	53	0.286	Protein A	0
	HOH150	0.84	-203	-0.204	0.00	21	-0.039	0.84	-182	-0.155	Neither	21
	HOH151	1.01	344	0.415	0.00	50	-0.038	1.01	394	0.437	Protein A	24
	HOH104	3.63	14	0.551	1.49	-275	-0.372	5.12	-260	-0.337	Protein A	14
	HOH105	2.11	-34	0.285	1.25	-40	0.234	3.36	-74	0.424	Protein A	28
	HOH106	1.48	29	0.315	4.18	-173	-0.135	5.66	-145	-0.068	Protein A	23
	HOH108	0.00	-147	-0.061	0.91	-42	0.202	0.91	-189	-0.172	Neither	24
	HOH109	0.00	-76	-0.040	2.34	198	0.589	2.34	121	0.505	Protein B	33

	HOH119	0.88	147	0.316	1.09	-22	0.248	1.97	125	0.463	Protein A	39
	HOH121	0.00	-343	-0.532	2.26	95	0.462	2.26	-249	-0.310	Protein B	36
	HOH127	0.00	-143	-0.055	0.00	-9	-0.039	0.00	-152	-0.067	Neither	22
	HOH146	2.39	79	0.463	1.23	-104	0.166	3.62	-26	0.511	Protein A	3
	HOH147	0.88	-160	-0.106	1.22	-7	0.267	2.10	-167	-0.122	Protein B	15
	HOH132	0.91	121	0.316	0.00	-42	-0.039	0.91	78	0.292	Protein A	15
	HOH144	2.53	594	0.812	0.99	-35	0.221	3.52	560	0.888	Protein A	55
	HOH159	0.95	-142	-0.067	0.95	-154	-0.094	1.89	-297	-0.426	Neither	11
	HOH154	2.27	-105	0.228	1.12	-49	0.229	3.39	-153	-0.090	Neither	25
	HOH180	1.02	4	0.255	0.95	-155	-0.096	1.97	-151	-0.088	Protein A	27
2ZD1	HOH559	3.40	14	0.520	0.00	-176	-0.109	3.40	-161	-0.108	Protein A	0
	HOH569	2.56	147	0.566	1.22	-158	-0.102	3.78	-11	0.552	Protein A	5
	HOH570	2.16	-79	0.235	1.02	-69	0.199	3.18	-149	-0.080	Neither	6
	HOH579	1.23	149	0.398	1.22	-162	-0.111	2.46	-13	0.373	Protein A	9
	HOH580	1.19	49	0.317	1.13	-140	-0.061	2.32	-91	0.256	Protein A	0
	HOH584	3.93	222	0.817	0.00	-266	-0.326	3.93	-44	0.536	Protein A	23
	HOH596	2.46	9	0.398	1.31	-240	-0.290	3.77	-231	-0.269	Protein A	16
	HOH600	1.32	58	0.338	2.35	-8	0.361	3.66	51	0.589	Both	23
	HOH601	0.95	21	0.257	0.00	-264	-0.321	0.95	-243	-0.296	Protein A	7
	HOH604	1.02	-183	-0.157	0.00	-197	-0.155	1.02	-380	-0.638	Neither	9
	HOH608	0.00	-340	-0.522	0.00	-110	-0.029	0.00	-450	-0.824	Neither	23
	HOH615	1.05	-90	0.186	0.00	-260	-0.311	1.05	-350	-0.560	Neither	26
	HOH651	4.03	-41	0.546	1.16	-37	0.238	5.19	-78	0.578	Protein A	1
	HOH674	1.03	-69	0.200	0.00	-92	-0.040	1.03	-161	-0.108	Neither	12
	HOH711	1.03	-429	-0.770	0.88	-173	-0.135	1.91	-602	-1.270	Neither	27
	HOH712	2.39	132	0.525	0.00	-54	-0.039	2.39	79	0.463	Protein A	2
	HOH717	2.03	-246	-0.304	0.00	-139	-0.051	2.03	-385	-0.652	Neither	25
	HOH730	2.46	99	0.492	0.00	-130	-0.041	2.46	-31	0.352	Protein A	17
	HOH751	0.91	63	0.281	0.00	-108	-0.040	0.91	-45	0.200	Protein A	8
	HOH752	0.00	-193	-0.144	0.71	112	0.276	0.71	-81	0.135	Protein B	2
	HOH755	0.77	-64	0.159	0.00	-150	-0.065	0.77	-214	-0.229	Neither	4
	HOH766	0.73	-260	-0.337	0.00	-177	-0.112	0.73	-437	-0.792	Neither	20
	HOH768	0.79	-2	0.211	0.00	-141	-0.053	0.79	-143	-0.069	Neither	10
	HOH815	1.00	-128	-0.035	0.93	29	0.260	1.93	-99	0.163	Protein B	33
	HOH819	0.75	18	0.219	0.00	-537	-1.073	0.75	-519	-1.024	Neither	0
	HOH837	4.02	467	0.902	1.17	-28	0.246	5.18	438	0.973	Protein A	32
	HOH842	1.14	305	0.433	0.89	-165	-0.118	2.03	139	0.488	Protein A	7
	HOH860	1.17	-201	-0.199	1.03	-82	0.191	2.20	-283	-0.392	Neither	19
	HOH867	0.00	-239	-0.258	0.00	-122	-0.035	0.00	-361	-0.579	Neither	0
	HOH877	2.16	176	0.545	0.00	-198	-0.157	2.16	-22	0.311	Protein A	9
	HOH879	0.00	-188	-0.134	0.00	-175	-0.107	0.00	-363	-0.584	Neither	35
	HOH899	1.03	-190	-0.173	0.00	-176	-0.110	1.03	-366	-0.602	Neither	0
	HOH922	3.61	-90	0.447	1.21	-113	-0.004	4.83	-203	-0.201	Protein A	12
	HOH925	2.64	36	0.451	1.20	52	0.320	3.84	88	0.646	Both	0
	HOH435	1.15	-84	0.196	1.18	-296	-0.424	2.33	-379	-0.637	Neither	7
	HOH436	1.04	-68	0.202	2.49	-103	0.272	3.53	-171	-0.130	Protein B	1
	HOH440	1.11	-151	-0.086	1.17	-35	0.240	2.27	-186	-0.164	Neither	4
	HOH447	2.47	-251	-0.314	2.35	201	0.592	4.82	-50	0.587	Protein B	4
	HOH456	0.85	-29	0.201	1.14	-429	-0.770	1.99	-458	-0.851	Neither	19
	HOH464	2.49	147	0.556	1.45	-155	-0.096	3.94	-8	0.570	Protein A	6
	HOH465	0.93	-103	0.155	1.17	-17	0.256	2.10	-120	-0.020	Protein B	9
	HOH467	0.87	-437	-0.793	1.14	-41	0.235	2.01	-478	-0.905	Neither	36
	HOH473	1.13	-110	0.004	4.01	92	0.660	5.14	-19	0.632	Protein B	15
	HOH479	0.92	-47	0.200	1.14	-143	-0.069	2.06	-190	-0.174	Neither	42
	HOH483	0.90	-6	0.228	1.24	-25	0.250	2.13	-31	0.295	Protein B	57
	HOH485	0.86	7	0.231	1.11	3	0.270	1.96	10	0.317	Protein B	9
	HOH491	2.38	-53	0.314	2.65	-129	-0.038	5.03	-182	-0.154	Protein A	27
	HOH494	1.20	59	0.326	2.13	-252	-0.318	3.33	-193	-0.180	Protein A	3
	HOH502	0.00	27	-0.038	0.91	-83	0.167	0.91	-56	0.189	Neither	12
	HOH508	1.01	53	0.291	1.17	-84	0.193	2.18	-31	0.304	Protein A	15

	HOH518	0.00	-50	-0.039	2.19	236	0.590	2.19	186	0.557	Protein B	10
	HOH528	0.71	-19	0.184	0.00	-117	-0.032	0.71	-136	-0.053	Neither	13
	HOH531	0.00	-155	-0.071	1.02	-4	0.249	1.02	-159	-0.104	Neither	12
	HOH533	2.62	-158	-0.101	2.53	-349	-0.559	5.15	-507	-0.989	Neither	39
	HOH535	0.00	-479	-0.907	3.58	22	0.552	3.58	-457	-0.848	Protein B	48
	HOH537	1.04	4	0.259	2.53	179	0.599	3.56	183	0.743	Both	32
	HOH538	0.00	-433	-0.777	1.98	-40	0.248	1.98	-473	-0.893	Neither	14
	HOH558	0.87	8	0.233	1.09	-15	0.254	1.96	-7	0.294	Protein B	33
	HOH563	0.93	42	0.269	0.97	-10	0.238	1.90	32	0.344	Protein A	32
	HOH564	0.88	-158	-0.103	0.86	-243	-0.298	1.74	-402	-0.696	Neither	2
	HOH567	1.05	30	0.280	1.11	10	0.275	2.16	40	0.386	Both	18
	HOH573	0.00	-318	-0.464	0.82	99	0.289	0.82	-219	-0.239	Protein B	31
	HOH585	0.00	-230	-0.234	2.35	289	0.643	2.35	59	0.436	Protein B	8
	HOH598	0.00	-245	-0.271	0.92	-51	0.196	0.92	-296	-0.424	Neither	49
	HOH602	0.00	-20	-0.039	2.27	577	0.792	2.27	557	0.785	Protein B	12
	HOH627	0.94	51	0.278	1.01	12	0.261	1.96	63	0.385	Both	43
	HOH629	0.00	-117	-0.033	1.06	345	0.426	1.06	228	0.359	Protein B	26
	HOH630	1.08	163	0.354	2.18	-110	0.003	3.26	53	0.540	Protein A	14
	HOH634	0.00	-186	-0.130	0.00	-374	-0.615	0.00	-560	-1.144	Neither	9
2ZFD	HOH234	4.00	-12	0.571	0.00	-95	-0.040	4.00	-108	0.479	Protein A	30
	HOH241	1.36	-96	0.162	2.50	-165	-0.117	3.86	-261	-0.339	Neither	7
	HOH246	1.33	-218	-0.238	2.30	107	0.481	3.63	-111	0.007	Protein B	36
	HOH251	2.88	133	0.585	2.78	-223	-0.250	5.66	-90	0.584	Protein A	23
	HOH252	1.16	-159	-0.103	1.14	-60	0.218	2.30	-219	-0.239	Neither	25
	HOH257	4.03	515	0.919	1.47	9	0.292	5.50	524	1.018	Both	1
	HOH265	1.99	5	0.312	0.00	-63	-0.039	1.99	-58	0.225	Protein A	9
	HOH300	1.16	33	0.298	0.86	-85	0.158	2.01	-52	0.239	Protein A	9
	HOH304	2.29	240	0.606	0.00	-70	-0.039	2.29	170	0.556	Protein A	34
	HOH306	1.00	20	0.265	0.00	-53	-0.039	1.00	-33	0.224	Protein A	1
	HOH332	1.86	-194	-0.185	0.00	-65	-0.039	1.86	-259	-0.336	Neither	0
	HOH339	2.41	454	0.746	1.35	9	0.287	3.75	463	0.876	Both	1
	HOH370	0.00	-57	-0.039	0.78	-11	0.204	0.78	-67	0.159	Neither	8
	HOH25	0.00	-213	-0.192	0.00	-281	-0.365	0.00	-494	-0.947	Neither	14
	HOH52	1.40	-183	-0.158	4.26	201	0.847	5.66	19	0.686	Protein B	5
	HOH78	0.00	-275	-0.349	2.05	-29	0.278	2.05	-303	-0.443	Protein B	36
	HOH82	1.22	298	0.470	2.23	26	0.382	3.45	324	0.787	Both	14
	HOH85	1.41	120	0.411	4.50	298	0.880	5.90	418	0.995	Both	15
	HOH99	0.00	-28	-0.039	0.95	50	0.278	0.95	23	0.257	Protein B	23
	HOH123	0.00	-220	-0.210	1.84	33	0.342	1.84	-187	-0.168	Protein B	0
	HOH132	1.16	-101	0.178	1.22	-57	0.217	2.38	-158	-0.103	Neither	1
	HOH134	1.08	34	0.288	2.07	431	0.721	3.15	465	0.819	Both	5
	HOH179	1.17	301	0.446	1.18	175	0.385	2.35	476	0.753	Both	2
	HOH198	0.90	86	0.295	0.94	-276	-0.376	1.83	-190	-0.174	Protein A	12
3A4U	HOH1	2.32	-237	-0.283	0.00	-269	-0.335	2.32	-506	-0.986	Neither	3
	HOH8	5.15	185	0.892	0.00	-236	-0.249	5.15	-51	0.602	Protein A	3
	HOH297	1.02	-38	0.223	0.00	-267	-0.329	1.02	-304	-0.445	Neither	0
	HOH309	1.14	-237	-0.283	1.02	250	0.363	2.16	12	0.353	Protein B	8
	HOH314	1.13	-27	0.247	0.85	-70	0.169	1.99	-97	0.167	Neither	3
	HOH369	2.25	95	0.462	1.02	55	0.294	3.27	150	0.656	Both	0
	HOH410	1.93	41	0.356	0.00	53	-0.038	1.93	94	0.422	Protein A	18
	HOH150	1.30	-56	0.214	2.34	390	0.706	3.64	334	0.813	Protein B	30
	HOH151	1.13	-24	0.250	2.75	-213	-0.226	3.89	-237	-0.281	Protein A	8
	HOH156	1.31	157	0.418	2.62	271	0.664	3.92	429	0.879	Both	9
	HOH157	1.13	-117	-0.010	2.54	428	0.744	3.68	311	0.807	Protein B	25
	HOH159	1.18	-22	0.252	4.00	784	0.975	5.18	762	1.055	Both	19
	HOH168	0.00	-136	-0.047	1.85	-231	-0.270	1.85	-367	-0.605	Neither	22
	HOH170	0.91	88	0.298	1.01	171	0.338	1.91	259	0.582	Both	19
	HOH187	4.04	-44	0.544	1.54	481	0.691	5.58	437	0.991	Both	39
	HOH189	0.71	-251	-0.315	0.00	-178	-0.113	0.71	-429	-0.769	Neither	2
3A8K	HOH23	2.22	-114	-0.006	0.00	-187	-0.132	2.22	-301	-0.438	Neither	10

	HOH44	3.75	-63	0.496	0.00	3	-0.039	3.75	-61	0.499	Protein A	22
	HOH56	2.41	450	0.744	0.00	-163	-0.084	2.41	287	0.650	Protein A	0
	HOH84	2.51	77	0.477	1.04	-134	-0.048	3.55	-56	0.470	Protein A	7
	HOH104	2.19	-442	-0.806	1.09	289	0.403	3.28	-153	-0.090	Protein B	29
	HOH198	1.34	13	0.291	2.23	10	0.363	3.57	23	0.550	Both	3
	HOH213	1.99	55	0.378	1.07	-13	0.251	3.06	43	0.506	Both	35
	HOH214	2.47	27	0.419	1.04	20	0.271	3.51	47	0.565	Both	31
	HOH463	0.00	-272	-0.344	0.00	-213	-0.191	0.00	-485	-0.922	Neither	2
	HOH565	2.38	-140	-0.063	1.14	87	0.339	3.52	-53	0.469	Protein B	6
	HOH608	0.91	-224	-0.252	0.00	-200	-0.162	0.91	-424	-0.757	Neither	0
	HOH690	1.01	22	0.267	0.81	62	0.263	1.82	83	0.403	Both	25
	HOH1335	0.87	21	0.244	1.20	-63	0.212	2.07	-42	0.266	Neither	16
	HOH1337	2.23	-191	-0.177	1.21	140	0.389	3.45	-51	0.461	Protein B	24
	HOH1338	3.80	-206	-0.210	1.45	310	0.555	5.25	104	0.744	Protein B	8
	HOH1349	0.00	-178	-0.113	0.85	195	0.298	0.85	17	0.236	Protein B	29
	HOH1350	0.00	-94	-0.040	2.31	53	0.424	2.31	-42	0.315	Protein B	16
	HOH1356	0.00	79	-0.038	0.90	-172	-0.134	0.90	-93	0.158	Neither	8
	HOH1358	2.12	-24	0.301	1.21	376	0.511	3.33	352	0.787	Both	11
	HOH1362	1.02	-95	0.177	2.53	-266	-0.351	3.55	-360	-0.588	Neither	32
	HOH1363	0.91	-392	-0.670	2.27	43	0.406	3.18	-350	-0.559	Protein B	44
	HOH1370	1.04	-117	-0.011	1.24	319	0.490	2.27	202	0.582	Protein B	12
	HOH1371	1.08	-80	0.199	1.23	491	0.572	2.30	411	0.716	Protein B	12
	HOH1375	1.06	-33	0.235	2.62	354	0.711	3.68	321	0.812	Protein B	12
	HOH1383	0.00	-76	-0.040	2.21	560	0.785	2.21	484	0.752	Protein B	0
	HOH1384	3.77	7	0.566	1.40	585	0.694	5.17	592	1.021	Both	12
	HOH1404	0.97	-49	0.206	1.15	-137	-0.055	2.12	-185	-0.164	Neither	0
	HOH1406	1.14	-28	0.246	2.42	356	0.693	3.57	329	0.802	Protein B	22
	HOH1419	0.99	-50	0.209	1.98	155	0.500	2.96	105	0.557	Protein B	2
	HOH1426	0.00	-42	-0.039	2.14	-152	-0.089	2.14	-193	-0.182	Neither	6
	HOH1441	1.72	77	0.388	0.00	-9	-0.039	1.72	68	0.378	Protein A	23
	HOH1444	0.89	11	0.239	1.15	454	0.510	2.04	465	0.743	Protein B	43
	HOH1445	0.00	-316	-0.459	0.00	-304	-0.428	0.00	-620	-1.327	Neither	9
3A98	HOH48	4.16	-98	0.501	1.45	113	0.409	5.61	15	0.681	Both	8
	HOH91	2.45	6	0.393	1.32	-70	0.197	3.77	-64	0.499	Protein A	0
	HOH95	2.18	11	0.355	0.91	35	0.260	3.09	46	0.513	Both	4
	HOH97	1.10	-279	-0.383	1.03	27	0.275	2.13	-252	-0.319	Protein B	49
	HOH106	2.21	43	0.398	0.00	-172	-0.101	2.21	-129	-0.039	Protein A	14
	HOH113	2.34	-129	-0.039	1.24	-264	-0.346	3.58	-393	-0.673	Neither	0
	HOH114	1.34	-151	-0.087	3.75	0	0.556	5.09	-151	-0.082	Protein B	10
	HOH117	0.85	-1	0.224	1.13	330	0.439	1.98	328	0.649	Protein B	25
	HOH119	1.01	22	0.269	2.16	29	0.373	3.17	51	0.527	Both	14
	HOH120	2.52	225	0.628	2.66	-54	0.356	5.18	171	0.868	Both	20
	HOH121	1.33	-64	0.203	2.54	223	0.630	3.87	159	0.750	Protein B	29
	HOH123	2.26	-41	0.306	1.38	163	0.436	3.64	122	0.657	Both	39
	HOH125	0.95	-88	0.172	1.32	361	0.554	2.28	272	0.625	Protein B	19
	HOH126	1.29	148	0.412	2.48	-108	0.264	3.77	40	0.595	Both	16
	HOH127	1.18	58	0.323	3.64	127	0.665	4.82	185	0.870	Both	11
	HOH128	0.00	-132	-0.044	0.82	129	0.302	0.82	-3	0.217	Protein B	7
	HOH130	2.57	110	0.518	2.95	263	0.702	5.52	373	0.965	Both	44
	HOH131	0.00	-96	-0.040	1.94	-355	-0.574	1.94	-451	-0.832	Neither	7
3BH7	HOH181	1.09	267	0.390	0.00	-12	-0.039	1.09	255	0.383	Protein A	36
	HOH185	0.00	-347	-0.541	1.73	-157	-0.102	1.73	-504	-0.981	Neither	13
	HOH188	2.70	390	0.740	2.46	-143	-0.069	5.17	247	0.917	Protein A	0
	HOH200	0.94	-85	0.172	0.00	-129	-0.041	0.94	-214	-0.230	Neither	29
	HOH203	1.01	-64	0.201	0.96	-124	-0.026	1.97	-188	-0.169	Neither	35
	HOH204	4.74	-352	-0.567	0.00	-199	-0.159	4.74	-552	-1.119	Neither	0
	HOH210	3.97	-300	-0.435	1.27	-206	-0.211	5.24	-506	-0.986	Neither	2
	HOH213	1.03	63	0.302	0.92	-376	-0.627	1.95	-312	-0.466	Protein A	10
	HOH221	1.35	147	0.423	2.52	521	0.784	3.87	668	0.945	Both	7
	HOH224	1.35	-10	0.266	2.24	-250	-0.312	3.59	-260	-0.336	Protein A	15

	HOH229	2.15	-159	-0.105	1.02	-359	-0.584	3.18	-518	-1.021	Neither	6
	HOH236	2.39	387	0.709	1.23	-337	-0.527	3.63	50	0.583	Protein A	8
	HOH242	2.33	-236	-0.281	1.34	191	0.435	3.67	-46	0.501	Protein B	9
	HOH351	1.08	278	0.393	0.95	-40	0.211	2.03	238	0.575	Protein A	38
	HOH365	0.00	71	-0.038	2.22	-108	0.212	2.22	-37	0.303	Neither	0
	HOH371	1.31	75	0.355	2.75	225	0.660	4.06	300	0.843	Both	12
	HOH379	2.92	300	0.716	2.99	533	0.831	5.91	833	1.092	Both	0
	HOH397	0.00	-227	-0.226	5.40	-6	0.654	5.40	-233	-0.272	Protein B	0
	HOH403	0.00	-285	-0.378	0.93	-302	-0.441	0.93	-588	-1.228	Neither	11
	HOH438	1.14	192	0.369	2.32	32	0.402	3.46	224	0.755	Both	24
	HOH466	0.79	29	0.235	1.04	-154	-0.093	1.83	-126	-0.033	Neither	32
	HOH475	0.00	-301	-0.418	2.16	-192	-0.179	2.16	-493	-0.949	Neither	1
	HOH480	1.31	-190	-0.174	2.69	183	0.625	4.00	-7	0.575	Protein B	4
	HOH481	0.00	-282	-0.369	2.14	103	0.457	2.14	-179	-0.149	Protein B	27
	HOH492	0.00	14	-0.039	3.63	-351	-0.563	3.63	-338	-0.529	Neither	2
	HOH504	0.95	-31	0.217	1.08	64	0.310	2.03	33	0.355	Protein B	9
3BN3	HOH1	5.51	-71	0.598	0.00	286	-0.038	5.51	215	0.941	Protein A	0
	HOH2	5.75	534	1.030	0.00	-124	-0.037	5.75	410	0.987	Protein A	4
	HOH6	1.03	-175	-0.139	0.95	-132	-0.045	1.97	-307	-0.452	Neither	13
	HOH10	2.50	-128	-0.036	0.96	-76	0.183	3.46	-204	-0.205	Neither	0
	HOH17	1.47	468	0.667	3.69	-7	0.540	5.16	461	0.980	Both	9
	HOH41	1.01	12	0.260	2.04	17	0.338	3.05	29	0.492	Both	34
	HOH47	0.73	-28	0.181	0.00	-27	-0.039	0.73	-55	0.159	Neither	1
	HOH69	1.13	-360	-0.586	1.08	57	0.306	2.22	-303	-0.442	Protein B	2
	HOH74	2.00	74	0.403	0.99	-16	0.236	2.99	58	0.513	Protein A	33
	HOH95	0.98	-51	0.207	0.88	-77	0.168	1.86	-127	-0.036	Neither	15
	HOH114	1.21	-597	-1.255	1.25	133	0.395	2.46	-464	-0.866	Protein B	21
	HOH141	1.26	-135	-0.053	4.26	-59	0.546	5.53	-194	-0.181	Protein B	27
	HOH153	1.17	-197	-0.191	2.31	263	0.622	3.48	65	0.577	Protein B	32
	HOH159	1.08	-235	-0.279	1.20	173	0.391	2.27	-62	0.283	Protein B	0
	HOH184	1.08	91	0.329	2.45	-158	-0.103	3.53	-67	0.457	Protein A	45
	HOH190	0.00	-53	-0.039	2.06	548	0.785	2.06	495	0.759	Protein B	42
	HOH191	0.00	-375	-0.617	2.20	16	0.365	2.20	-359	-0.584	Protein B	5
	HOH193	0.84	-404	-0.702	0.83	-34	0.194	1.67	-438	-0.794	Neither	23
	HOH194	0.97	-150	-0.084	0.98	151	0.335	1.95	1	0.303	Protein B	7
	HOH196	2.18	29	0.377	1.25	175	0.407	3.42	204	0.749	Both	6
	HOH204	0.00	119	-0.038	1.99	113	0.449	1.99	232	0.567	Protein B	27
	HOH212	0.87	-129	-0.039	0.88	-8	0.224	1.75	-137	-0.057	Neither	19
3BN9	HOH425	4.84	278	0.900	0.00	46	-0.038	4.84	324	0.912	Protein A	1
	HOH437	2.06	-120	-0.019	1.04	11	0.265	3.11	-109	0.351	Protein B	8
	HOH438	2.40	-13	0.364	1.13	-58	0.221	3.52	-71	0.451	Protein A	9
	HOH439	1.31	214	0.434	2.25	-237	-0.283	3.56	-23	0.505	Protein A	5
	HOH441	4.18	-95	0.505	1.41	67	0.355	5.59	-28	0.641	Both	0
	HOH450	4.04	-177	-0.142	1.14	37	0.301	5.18	-139	-0.056	Protein B	2
	HOH453	4.21	-63	0.538	1.26	-11	0.263	5.46	-74	0.593	Both	4
	HOH458	2.62	-174	-0.138	1.25	-60	0.212	3.87	-234	-0.275	Neither	0
	HOH468	0.00	-266	-0.326	0.77	-9	0.202	0.77	-274	-0.371	Neither	28
	HOH473	1.96	180	0.524	1.07	-5	0.258	3.03	175	0.662	Both	30
	HOH474	1.05	-34	0.232	0.91	32	0.258	1.96	-2	0.301	Protein B	47
	HOH475	0.95	98	0.312	0.85	15	0.236	1.80	114	0.438	Protein A	48
	HOH496	3.82	-203	-0.202	1.11	50	0.305	4.93	-152	-0.086	Protein B	0
	HOH521	1.41	254	0.498	2.46	-34	0.348	3.86	220	0.812	Both	14
	HOH523	0.00	-33	-0.039	0.72	24	0.219	0.72	-9	0.194	Neither	21
	HOH550	2.15	652	0.820	0.97	-192	-0.178	3.12	460	0.814	Protein A	30
	HOH552	1.11	172	0.361	0.86	-173	-0.135	1.97	-1	0.302	Protein A	37
	HOH591	2.50	-247	-0.307	1.24	119	0.383	3.74	-128	-0.031	Protein B	0
	HOH602	2.61	-157	-0.099	1.56	-109	0.003	4.16	-266	-0.351	Neither	2
	HOH624	0.88	-102	0.148	1.11	-17	0.254	1.99	-119	-0.018	Protein B	23
	HOH684	0.00	-103	-0.040	3.39	-10	0.494	3.39	-113	0.002	Protein B	17
	HOH722	1.25	62	0.335	1.28	232	0.441	2.53	294	0.667	Both	42

	HOH743	1.01	12	0.261	2.32	-125	-0.030	3.33	-113	0.002	Protein A	3
	HOH750	2.40	-45	0.326	2.63	236	0.648	5.02	191	0.894	Both	1
	HOH752	0.00	76	-0.038	0.80	-178	-0.146	0.80	-102	0.133	Neither	30
	HOH754	0.00	-127	-0.039	1.06	35	0.286	1.06	-92	0.187	Protein B	47
	HOH756	0.85	-26	0.204	1.34	90	0.372	2.19	64	0.418	Protein B	27
	HOH766	2.36	-25	0.343	1.27	184	0.416	3.64	159	0.717	Both	3
	HOH771	2.47	295	0.660	2.48	-211	-0.222	4.95	84	0.712	Protein A	3
	HOH773	1.96	-25	0.268	1.24	-148	-0.081	3.19	-174	-0.136	Protein A	24
	HOH784	0.80	34	0.241	0.99	14	0.258	1.79	47	0.357	Protein B	66
	HOH788	2.21	-66	0.266	1.32	274	0.489	3.53	208	0.768	Both	7
3BWU	HOH217	2.67	-9	0.406	1.29	102	0.378	3.95	93	0.657	Both	0
	HOH219	1.40	-296	-0.423	3.64	-145	-0.070	5.04	-440	-0.800	Neither	2
	HOH228	1.98	-555	-1.131	0.00	-233	-0.242	1.98	-789	-1.880	Neither	6
	HOH230	1.23	-496	-0.957	2.11	-172	-0.135	3.34	-668	-1.480	Neither	1
	HOH249	2.22	14	0.366	1.05	-43	0.224	3.27	-29	0.459	Protein A	1
	HOH250	1.18	-193	-0.181	0.90	32	0.257	2.08	-161	-0.109	Protein B	0
	HOH253	2.45	-49	0.330	1.02	-262	-0.342	3.47	-311	-0.461	Protein A	5
	HOH262	1.15	-101	0.180	0.90	-132	-0.044	2.05	-233	-0.273	Neither	6
	HOH301	3.67	268	0.792	0.00	-155	-0.072	3.67	113	0.648	Protein A	0
	HOH306	1.22	64	0.333	1.08	25	0.282	2.30	89	0.461	Both	0
	HOH331	1.17	39	0.304	1.03	129	0.341	2.20	168	0.541	Both	9
	HOH347	0.95	39	0.270	0.00	-192	-0.144	0.95	-154	-0.092	Protein A	0
	HOH382	0.00	-168	-0.094	0.00	-202	-0.166	0.00	-370	-0.603	Neither	29
	HOH447	3.87	101	0.661	1.08	-5	0.259	4.94	96	0.722	Both	18
	HOH469	1.12	98	0.342	1.06	-80	0.197	2.18	19	0.364	Protein A	4
	HOH160	2.33	-289	-0.407	0.00	-179	-0.114	2.33	-467	-0.876	Neither	1
	HOH234	1.12	-25	0.248	1.03	327	0.411	2.15	302	0.635	Protein B	30
	HOH156	1.30	-270	-0.361	2.60	-75	0.322	3.90	-345	-0.547	Protein B	29
	HOH159	2.22	-115	-0.008	1.29	-1	0.275	3.51	-116	-0.004	Protein B	0
	HOH162	1.04	-33	0.232	2.58	366	0.714	3.62	333	0.810	Protein B	0
	HOH163	1.16	-238	-0.285	2.36	8	0.381	3.52	-231	-0.267	Protein B	2
	HOH164	2.40	-224	-0.251	1.26	-130	-0.040	3.66	-353	-0.568	Neither	0
	HOH166	2.21	-488	-0.933	1.30	-14	0.261	3.50	-501	-0.973	Protein B	14
	HOH167	0.00	-240	-0.261	1.00	-168	-0.124	1.00	-409	-0.714	Neither	25
	HOH169	0.00	-188	-0.134	3.85	-80	0.497	3.85	-269	-0.357	Protein B	30
	HOH170	1.14	-347	-0.552	2.61	218	0.639	3.75	-128	-0.032	Protein B	11
	HOH171	1.23	-85	0.187	3.76	-114	0.002	4.99	-199	-0.193	Neither	22
	HOH176	0.00	-410	-0.711	3.65	-77	0.466	3.65	-486	-0.929	Protein B	25
	HOH180	1.20	-88	0.186	1.28	80	0.356	2.49	-8	0.382	Protein B	29
	HOH183	2.49	117	0.518	2.85	548	0.823	5.34	665	1.047	Both	11
	HOH198	0.98	-332	-0.515	1.11	-151	-0.086	2.09	-483	-0.920	Neither	5
	HOH204	1.21	-155	-0.097	1.89	-144	-0.073	3.10	-300	-0.434	Neither	0
	HOH215	0.00	-242	-0.265	1.05	5	0.263	1.05	-237	-0.282	Protein B	18
	HOH216	1.11	23	0.286	1.26	-30	0.244	2.38	-6	0.368	Protein A	26
	HOH231	0.00	-102	-0.040	2.30	-92	0.250	2.30	-194	-0.182	Protein B	18
	HOH232	0.00	-190	-0.138	0.00	-211	-0.186	0.00	-400	-0.686	Neither	4
	HOH238	0.00	-335	-0.509	0.00	-148	-0.062	0.00	-483	-0.918	Neither	37
	HOH242	0.00	-204	-0.171	0.00	-64	-0.039	0.00	-269	-0.334	Neither	8
	HOH248	0.90	-169	-0.127	1.05	-90	0.186	1.95	-260	-0.337	Neither	22
	HOH251	0.98	-249	-0.310	1.14	121	0.361	2.12	-128	-0.037	Protein B	29
	HOH256	0.87	-76	0.166	1.01	-150	-0.083	1.87	-226	-0.256	Neither	51
	HOH257	0.00	-302	-0.420	0.93	136	0.323	0.93	-165	-0.119	Protein B	36
	HOH263	3.64	-122	-0.017	1.28	-47	0.224	4.92	-169	-0.124	Neither	45
	HOH272	0.00	-201	-0.164	1.05	17	0.271	1.05	-184	-0.160	Protein B	50
	HOH285	0.86	-31	0.202	1.13	54	0.312	1.99	23	0.336	Protein B	14
	HOH312	0.89	63	0.278	0.99	5	0.252	1.89	68	0.388	Both	47
3BX1	HOH81	2.28	130	0.507	1.18	96	0.354	3.46	226	0.756	Both	10
	HOH94	3.86	-242	-0.294	1.36	43	0.326	5.22	-199	-0.193	Protein B	20
	HOH164	1.01	-216	-0.232	1.01	1	0.252	2.02	-214	-0.230	Protein B	1
	HOH190	2.33	21	0.392	1.08	-104	0.180	3.41	-83	0.422	Protein A	11

	HOH192	2.52	85	0.486	1.19	-524	-1.038	3.70	-439	-0.796	Protein A	29
	HOH211	3.99	-184	-0.159	1.13	-156	-0.096	5.11	-340	-0.534	Neither	0
	HOH218	2.12	218	0.571	0.00	-29	-0.039	2.12	190	0.551	Protein A	0
	HOH220	1.46	-217	-0.236	3.76	-211	-0.221	5.21	-428	-0.766	Neither	42
	HOH233	2.54	108	0.513	1.05	-25	0.239	3.59	84	0.608	Protein A	11
	HOH240	1.09	152	0.356	0.86	35	0.252	1.96	186	0.528	Both	18
	HOH279	2.08	-243	-0.296	1.02	253	0.367	3.11	10	0.479	Protein B	40
	HOH308	2.52	-10	0.384	2.47	112	0.509	4.99	101	0.729	Both	57
	HOH341	0.98	-28	0.225	1.04	89	0.322	2.03	61	0.390	Protein B	18
	HOH395	0.82	-4	0.215	0.00	-193	-0.144	0.82	-197	-0.189	Neither	18
	HOH435	1.10	0	0.267	2.25	-112	0.000	3.34	-112	0.005	Protein A	41
	HOH438	1.05	-54	0.215	2.21	52	0.407	3.25	-2	0.484	Protein B	32
	HOH441	0.00	-36	-0.039	0.89	-201	-0.199	0.89	-237	-0.283	Neither	0
	HOH449	0.00	-109	-0.040	1.10	-229	-0.264	1.10	-338	-0.530	Neither	28
	HOH451	1.22	-86	0.187	4.17	-49	0.549	5.38	-135	-0.045	Protein B	21
	HOH485	1.13	-39	0.237	2.33	24	0.395	3.46	-15	0.499	Protein B	18
	HOH491	0.00	-369	-0.600	0.96	-4	0.239	0.96	-373	-0.620	Neither	26
	HOH515	0.00	-498	-0.959	0.99	10	0.256	0.99	-488	-0.933	Protein B	30
	HOH546	1.16	97	0.351	2.46	116	0.513	3.62	213	0.777	Both	8
	HOH549	0.00	-185	-0.127	0.87	184	0.306	0.87	0	0.227	Protein B	0
	HOH551	0.00	-98	-0.040	1.93	-123	-0.027	1.93	-221	-0.245	Neither	50
	HOH552	1.22	-188	-0.170	2.56	35	0.439	3.77	-153	-0.088	Protein B	11
	HOH563	0.00	-162	-0.083	0.86	-34	0.200	0.86	-196	-0.186	Neither	32
	HOH567	1.31	-167	-0.122	3.70	-86	0.465	5.01	-253	-0.319	Protein B	3
3BX7	HOH4	0.95	147	0.328	2.41	-21	0.355	3.35	126	0.628	Both	1
	HOH8	3.58	-363	-0.595	1.14	132	0.365	4.72	-231	-0.268	Protein B	0
	HOH9	2.07	-34	0.277	1.25	-269	-0.359	3.32	-303	-0.442	Protein A	0
	HOH11	0.94	-271	-0.364	1.05	-44	0.225	1.99	-315	-0.472	Neither	4
	HOH15	1.02	-34	0.226	2.67	-10	0.407	3.69	-44	0.505	Protein B	3
	HOH16	0.85	-194	-0.183	1.08	-81	0.198	1.92	-275	-0.374	Neither	2
	HOH18	0.00	-261	-0.314	0.96	-113	-0.004	0.96	-375	-0.625	Neither	31
	HOH20	0.90	41	0.263	1.17	-284	-0.396	2.07	-243	-0.298	Protein A	18
	HOH24	0.85	-22	0.207	1.08	-220	-0.242	1.93	-242	-0.294	Neither	0
	HOH34	1.11	-114	-0.004	1.15	124	0.364	2.26	10	0.367	Protein B	9
	HOH36	1.02	-240	-0.289	2.21	-83	0.243	3.23	-323	-0.491	Neither	6
	HOH39	2.17	2	0.343	0.00	-278	-0.357	2.17	-276	-0.375	Protein A	11
	HOH40	0.00	-229	-0.232	0.86	-311	-0.461	0.86	-540	-1.084	Neither	27
	HOH41	1.39	143	0.427	4.24	-338	-0.529	5.62	-194	-0.182	Protein A	19
	HOH43	0.00	-126	-0.038	1.86	-184	-0.162	1.86	-310	-0.459	Neither	27
	HOH44	1.13	-70	0.210	2.28	-164	-0.116	3.41	-234	-0.275	Neither	11
	HOH57	1.54	-239	-0.288	3.63	370	0.826	5.17	131	0.786	Protein B	0
	HOH63	0.00	-205	-0.172	0.88	-85	0.162	0.88	-290	-0.410	Neither	2
	HOH65	1.34	214	0.442	2.23	-116	-0.010	3.56	98	0.616	Protein A	1
	HOH71	0.74	1	0.205	0.00	-436	-0.785	0.74	-436	-0.788	Neither	4
	HOH76	1.16	-48	0.228	3.77	-72	0.492	4.94	-120	-0.011	Protein B	1
	HOH100	3.45	-468	-0.878	0.00	-137	-0.048	3.45	-604	-1.279	Neither	0
	HOH108	1.10	41	0.298	0.81	-51	0.176	1.91	-10	0.288	Protein A	0
	HOH109	1.91	-133	-0.048	0.00	-338	-0.517	1.91	-471	-0.885	Neither	1
	HOH111	1.13	-247	-0.306	0.91	-89	0.164	2.05	-336	-0.526	Neither	1
	HOH116	1.27	326	0.510	1.96	-59	0.221	3.23	267	0.738	Protein A	39
	HOH131	2.27	156	0.538	1.11	-177	-0.145	3.38	-21	0.482	Protein A	8
	HOH135	0.98	4	0.249	0.00	-240	-0.258	0.98	-236	-0.279	Neither	0
	HOH151	1.99	100	0.432	0.00	-271	-0.341	1.99	-172	-0.133	Protein A	0
	HOH165	1.29	-85	0.181	2.08	-87	0.205	3.37	-172	-0.133	Neither	13
	HOH167	0.73	6	0.207	0.00	-362	-0.582	0.73	-356	-0.575	Neither	2
3CBJ	HOH264	1.22	31	0.303	1.16	-369	-0.611	2.38	-339	-0.532	Protein A	0
	HOH266	3.66	-193	-0.181	1.14	-349	-0.557	4.80	-542	-1.091	Neither	0
	HOH267	2.40	-214	-0.228	2.37	-272	-0.366	4.77	-485	-0.927	Neither	13
	HOH326	2.00	52	0.375	1.22	-47	0.227	3.22	4	0.487	Protein A	0
	HOH356	2.46	145	0.550	1.27	-153	-0.092	3.72	-8	0.544	Protein A	0

	HOH362	1.24	149	0.400	0.89	-222	-0.247	2.13	-73	0.238	Protein A	8
	HOH373	1.91	81	0.405	0.97	-48	0.207	2.88	33	0.476	Protein A	23
	HOH375	1.26	-53	0.220	2.17	-95	0.219	3.43	-148	-0.077	Neither	6
	HOH378	1.15	299	0.435	1.14	60	0.318	2.29	359	0.684	Both	12
	HOH399	2.26	-31	0.319	1.03	33	0.279	3.29	1	0.493	Both	12
	HOH414	0.00	3	-0.039	0.00	-45	-0.039	0.00	-42	-0.039	Neither	0
	HOH430	1.05	103	0.332	0.00	-99	-0.040	1.05	4	0.261	Protein A	8
	HOH442	1.29	-169	-0.127	1.09	35	0.291	2.37	-134	-0.049	Protein B	5
	HOH114	2.40	-223	-0.249	1.32	-59	0.209	3.72	-282	-0.389	Neither	10
	HOH117	0.00	-99	-0.040	2.35	55	0.432	2.35	-44	0.319	Protein B	0
	HOH120	0.00	-154	-0.070	1.02	-250	-0.312	1.02	-403	-0.700	Neither	0
	HOH121	1.45	333	0.574	4.29	216	0.853	5.74	549	1.034	Both	0
	HOH123	2.75	455	0.776	2.79	132	0.573	5.54	587	1.037	Both	5
	HOH124	0.00	-523	-1.033	1.02	-9	0.246	1.02	-532	-1.062	Neither	26
	HOH125	0.00	-305	-0.429	1.96	39	0.355	1.96	-265	-0.350	Protein B	18
	HOH133	0.98	-40	0.215	2.69	110	0.532	3.67	70	0.606	Protein B	30
	HOH136	0.00	-259	-0.309	0.00	-240	-0.261	0.00	-499	-0.964	Neither	6
	HOH137	0.00	-306	-0.431	0.87	-190	-0.173	0.87	-495	-0.955	Neither	31
	HOH138	1.34	83	0.366	2.36	-494	-0.952	3.69	-411	-0.722	Protein A	36
	HOH142	0.00	-78	-0.040	2.01	90	0.424	2.01	13	0.326	Protein B	37
	HOH143	0.00	-564	-1.156	2.25	-288	-0.404	2.25	-852	-2.102	Neither	18
	HOH160	0.00	-258	-0.307	1.19	-135	-0.051	1.19	-393	-0.674	Neither	12
3CIP	HOH417	1.14	27	0.292	0.95	15	0.252	2.09	42	0.378	Both	8
	HOH423	0.76	-8	0.202	0.00	-299	-0.414	0.76	-307	-0.453	Neither	8
	HOH429	2.56	-206	-0.211	1.10	-33	0.241	3.66	-240	-0.288	Neither	25
	HOH451	0.70	122	0.278	0.00	-181	-0.119	0.70	-59	0.151	Protein A	41
	HOH475	2.03	58	0.387	0.00	15	-0.039	2.03	72	0.405	Protein A	25
	HOH491	0.91	-1	0.234	0.80	6	0.219	1.71	5	0.299	Neither	45
	HOH507	0.00	-138	-0.049	0.76	-232	-0.270	0.76	-369	-0.610	Neither	26
	HOH541	1.11	84	0.329	0.92	-4	0.233	2.03	80	0.414	Protein A	40
	HOH542	1.17	347	0.473	0.87	-36	0.200	2.04	312	0.637	Protein A	3
	HOH563	1.17	-116	-0.010	2.21	146	0.518	3.38	29	0.532	Protein B	43
	HOH570	0.88	232	0.323	0.84	-17	0.208	1.72	216	0.518	Protein A	2
	HOH586	2.27	299	0.643	1.23	-89	0.183	3.50	210	0.761	Protein A	33
	HOH592	2.34	390	0.706	0.00	-283	-0.373	2.34	106	0.486	Protein A	42
	HOH625	1.16	21	0.289	1.16	-99	0.180	2.32	-79	0.271	Protein A	31
	HOH660	2.20	-185	-0.162	0.00	-257	-0.304	2.20	-442	-0.806	Neither	20
	HOH662	2.25	576	0.792	0.00	-249	-0.283	2.25	327	0.660	Protein A	23
	HOH665	2.66	69	0.487	1.23	-196	-0.188	3.89	-127	-0.029	Protein A	50
	HOH667	5.15	229	0.918	0.00	22	-0.039	5.15	251	0.916	Protein A	1
	HOH714	1.06	-59	0.214	0.00	-155	-0.072	1.06	-214	-0.228	Neither	28
	HOH725	0.77	-14	0.199	0.00	-95	-0.040	0.77	-109	0.122	Neither	6
	HOH208	2.37	53	0.433	1.35	-125	-0.031	3.71	-72	0.482	Protein A	17
	HOH209	2.20	-28	0.312	1.36	-30	0.243	3.57	-58	0.472	Protein A	15
	HOH230	1.03	316	0.405	1.10	188	0.356	2.13	504	0.761	Both	4
	HOH259	0.76	47	0.243	0.00	-213	-0.193	0.76	-166	-0.121	Neither	16
	HOH265	2.27	107	0.479	1.36	129	0.412	3.63	236	0.781	Both	2
	HOH306	1.07	36	0.288	1.19	194	0.388	2.25	230	0.595	Both	15
	HOH315	0.82	-169	-0.126	1.10	201	0.352	1.92	32	0.344	Protein B	39
	HOH330	0.71	9	0.206	0.00	-20	-0.039	0.71	-11	0.191	Neither	35
3CX8	HOH756	0.99	-364	-0.597	0.00	-100	-0.040	0.99	-464	-0.866	Neither	51
	HOH766	3.66	-2	0.541	0.00	40	-0.038	3.66	39	0.578	Protein A	11
	HOH778	2.58	35	0.442	1.23	6	0.280	3.82	40	0.603	Both	0
	HOH800	1.37	12	0.291	2.19	115	0.477	3.56	127	0.654	Both	0
	HOH810	0.98	-404	-0.702	0.00	-151	-0.065	0.98	-555	-1.128	Neither	7
	HOH837	2.54	186	0.607	1.21	-135	-0.053	3.75	51	0.601	Protein A	0
	HOH838	2.11	285	0.619	0.00	-125	-0.037	2.11	160	0.521	Protein A	0
	HOH856	2.88	469	0.795	2.67	30	0.448	5.54	499	1.011	Both	2
	HOH865	2.26	117	0.489	0.00	-304	-0.426	2.26	-187	-0.166	Protein A	16
	HOH903	2.25	-139	-0.060	1.11	-40	0.236	3.36	-178	-0.146	Neither	11

	HOH932	1.22	67	0.337	0.91	-95	0.159	2.14	-28	0.298	Protein A	32
	HOH957	2.65	79	0.496	0.96	-3	0.241	3.61	76	0.604	Protein A	4
	HOH959	1.29	80	0.358	2.06	-8	0.309	3.35	72	0.568	Both	2
	HOH965	2.26	-7	0.347	0.00	-198	-0.157	2.26	-205	-0.209	Protein A	16
	HOH970	3.45	263	0.764	0.00	-53	-0.039	3.45	210	0.755	Protein A	0
	HOH4	3.90	-222	-0.248	1.34	-230	-0.267	5.24	-453	-0.835	Neither	2
	HOH22	1.05	-106	0.173	2.36	216	0.601	3.41	110	0.611	Protein B	19
	HOH23	3.97	93	0.659	1.54	-430	-0.772	5.51	-337	-0.526	Protein A	5
	HOH26	1.08	-174	-0.138	2.20	-193	-0.182	3.28	-367	-0.606	Neither	11
	HOH33	0.00	-69	-0.039	3.69	63	0.602	3.69	-7	0.540	Protein B	14
	HOH77	0.84	-164	-0.116	1.13	242	0.388	1.97	77	0.403	Protein B	4
	HOH84	0.00	-242	-0.265	2.10	-274	-0.371	2.10	-516	-1.015	Neither	22
	HOH92	1.02	-72	0.196	2.45	-206	-0.210	3.46	-278	-0.380	Neither	40
	HOH115	0.00	-41	-0.039	0.81	203	0.288	0.81	162	0.300	Protein B	4
	HOH159	1.02	-75	0.193	1.22	-28	0.246	2.23	-104	0.221	Neither	6
	HOH221	0.92	-202	-0.200	2.52	157	0.573	3.44	-45	0.466	Protein B	18
	HOH222	0.00	-169	-0.096	1.04	-64	0.207	1.04	-233	-0.273	Neither	9
	HOH223	1.36	-14	0.261	2.48	433	0.741	3.84	419	0.868	Both	19
	HOH233	1.03	-118	-0.013	1.22	312	0.475	2.24	195	0.572	Protein B	29
	HOH274	1.03	83	0.314	2.14	-739	-1.712	3.17	-656	-1.442	Protein A	2
	HOH276	0.89	-88	0.161	1.16	55	0.318	2.05	-32	0.275	Protein B	9
	HOH287	2.42	-68	0.303	2.55	37	0.440	4.97	-30	0.614	Both	0
	HOH292	0.00	-180	-0.117	1.00	-11	0.241	1.00	-190	-0.175	Neither	16
3D85	HOH512	1.47	160	0.450	4.17	-115	0.001	5.64	45	0.708	Protein A	26
	HOH550	3.66	108	0.640	0.00	-215	-0.197	3.66	-108	0.435	Protein A	54
	HOH620	1.15	106	0.355	0.90	14	0.243	2.05	119	0.466	Protein A	6
	HOH582	5.27	102	0.743	0.00	-249	-0.283	5.27	-147	-0.075	Protein A	14
	HOH599	2.12	37	0.377	1.18	31	0.299	3.30	68	0.558	Both	28
	HOH836	1.26	97	0.369	3.47	-98	0.414	4.73	-1	0.627	Both	1
	HOH665	0.74	-35	0.177	0.00	-52	-0.039	0.74	-87	0.135	Neither	1
	HOH680	0.95	-53	0.199	0.92	-120	-0.018	1.87	-173	-0.136	Neither	0
	HOH686	1.07	-104	0.179	2.49	484	0.767	3.57	380	0.824	Protein B	9
	HOH687	0.88	-172	-0.133	1.06	217	0.351	1.94	45	0.362	Protein B	34
	HOH688	1.13	67	0.323	1.22	-100	0.172	2.35	-33	0.332	Protein A	27
	HOH694	2.17	-121	-0.022	1.12	-8	0.262	3.29	-129	-0.036	Protein B	15
	HOH698	0.00	-57	-0.039	2.07	-501	-0.970	2.07	-557	-1.136	Neither	0
	HOH766	0.90	26	0.252	1.13	96	0.344	2.03	122	0.466	Both	0
	HOH799	1.14	65	0.322	1.16	123	0.368	2.30	188	0.575	Both	0
	HOH994	0.00	-147	-0.060	0.00	-102	-0.040	0.00	-249	-0.282	Neither	3
	HOH1084	0.82	23	0.235	0.86	-43	0.191	1.67	-20	0.264	Neither	1
	HOH1251	0.80	84	0.276	0.97	22	0.262	1.77	106	0.427	Both	0
	HOH1262	1.11	44	0.300	2.56	-207	-0.214	3.67	-164	-0.113	Protein A	0
	HOH1269	1.12	133	0.360	1.37	18	0.298	2.49	151	0.561	Both	0
	HOH1356	0.82	-6	0.215	1.13	-47	0.230	1.95	-53	0.230	Neither	0
	HOH684	1.41	92	0.382	4.02	-445	-0.813	5.43	-353	-0.569	Protein A	22
	HOH693	2.39	17	0.396	1.23	-228	-0.261	3.62	-211	-0.221	Protein A	6
	HOH695	1.49	501	0.689	4.22	-193	-0.180	5.71	308	0.952	Protein A	37
	HOH701	2.56	455	0.759	1.23	105	0.371	3.79	561	0.911	Both	45
	HOH719	1.44	412	0.626	2.32	129	0.512	3.76	541	0.903	Both	12
	HOH741	1.44	509	0.678	2.34	-131	-0.042	3.78	378	0.846	Protein A	4
	HOH804	3.82	540	0.908	1.18	-22	0.252	5.00	518	0.990	Both	0
	HOH881	2.63	23	0.435	2.78	59	0.490	5.40	81	0.729	Both	4
	HOH900	1.13	100	0.346	0.86	-201	-0.200	1.99	-101	0.162	Protein A	58
3D9A	HOH3	4.09	49	0.631	1.49	116	0.415	5.58	165	0.876	Both	27
	HOH4	2.50	143	0.553	2.77	-103	0.313	5.27	40	0.690	Both	33
	HOH7	0.00	-175	-0.107	1.06	133	0.347	1.06	-42	0.227	Protein B	0
	HOH9	0.00	94	-0.038	3.34	-385	-0.651	3.34	-291	-0.413	Neither	1
	HOH16	1.46	15	0.298	4.32	-86	0.523	5.78	-70	0.607	Both	3
	HOH17	1.06	-177	-0.144	2.21	94	0.456	3.28	-83	0.403	Protein B	17
	HOH23	0.80	-4	0.212	1.03	-226	-0.258	1.83	-230	-0.267	Neither	18

	HOH27	1.32	442	0.598	2.52	-89	0.294	3.84	354	0.843	Both	19
	HOH31	0.89	-34	0.204	1.10	231	0.369	1.98	197	0.540	Protein B	38
	HOH41	2.01	74	0.404	1.12	71	0.323	3.13	146	0.632	Both	10
	HOH49	2.58	140	0.559	2.81	89	0.523	5.39	229	0.931	Both	17
	HOH53	1.17	142	0.377	1.50	-368	-0.608	2.67	-227	-0.258	Protein A	5
	HOH54	0.00	-68	-0.039	0.00	-13	-0.039	0.00	-81	-0.040	Neither	29
	HOH62	1.38	30	0.312	3.78	107	0.656	5.17	137	0.797	Both	17
	HOH66	2.15	228	0.580	1.25	37	0.311	3.39	265	0.757	Both	8
	HOH107	0.92	20	0.251	1.03	-218	-0.237	1.95	-197	-0.191	Protein A	0
	HOH123	1.05	16	0.271	2.26	38	0.400	3.31	54	0.546	Both	15
	HOH157	2.96	322	0.732	2.83	69	0.506	5.80	390	0.982	Both	12
	HOH165	2.50	20	0.416	1.34	3	0.281	3.85	24	0.593	Both	7
	HOH184	1.09	15	0.276	1.00	52	0.288	2.08	67	0.406	Both	17
	HOH265	1.01	63	0.299	0.00	-195	-0.151	1.01	-132	-0.045	Protein A	10
	HOH285	1.09	143	0.355	0.89	-99	0.152	1.99	45	0.363	Protein A	0
	HOH330	2.41	-53	0.318	1.22	62	0.332	3.63	9	0.546	Both	8
	HOH350	2.43	127	0.524	1.20	-3	0.270	3.63	124	0.660	Both	44
	HOH454	3.87	578	0.923	0.00	-26	-0.039	3.87	552	0.916	Protein A	7
	HOH470	1.81	366	0.656	0.00	-146	-0.059	1.81	220	0.534	Protein A	8
	HOH487	4.17	-263	-0.343	1.33	142	0.417	5.51	-120	-0.011	Protein B	48
	HOH502	5.19	723	1.050	0.00	-73	-0.040	5.19	650	1.036	Protein A	7
	HOH573	1.56	586	0.744	3.67	-75	0.471	5.23	512	1.001	Both	0
	HOH593	1.33	68	0.349	2.32	13	0.381	3.65	80	0.612	Both	18
	HOH616	0.82	-149	-0.082	0.00	64	-0.038	0.82	-85	0.150	Neither	0
	HOH639	3.40	439	0.833	0.00	82	-0.038	3.40	521	0.864	Protein A	8
	HOH675	2.93	664	0.865	2.45	60	0.452	5.38	724	1.059	Both	1
3DDC	HOH7	2.85	322	0.719	2.56	1	0.403	5.41	323	0.943	Both	4
	HOH14	0.94	-213	-0.227	0.92	-75	0.176	1.86	-288	-0.406	Neither	0
	HOH16	1.33	121	0.400	2.23	-222	-0.248	3.56	-101	0.425	Protein A	7
	HOH20	2.54	-64	0.327	1.03	-28	0.232	3.57	-93	0.436	Protein A	0
	HOH21	1.00	12	0.260	0.00	-129	-0.041	1.00	-117	-0.012	Protein A	22
	HOH22	2.60	-106	0.285	1.29	54	0.331	3.89	-52	0.525	Both	10
	HOH24	2.46	390	0.716	1.25	33	0.307	3.71	423	0.856	Both	2
	HOH26	1.98	-31	0.260	0.00	-209	-0.182	1.98	-240	-0.290	Protein A	3
	HOH34	1.00	-127	-0.033	0.00	-245	-0.272	1.00	-372	-0.617	Neither	0
	HOH36	2.28	100	0.471	0.00	-239	-0.257	2.28	-139	-0.060	Protein A	15
	HOH37	1.98	177	0.523	0.00	-194	-0.148	1.98	-17	0.279	Protein A	19
	HOH57	1.01	140	0.339	0.00	-250	-0.284	1.01	-110	0.005	Protein A	17
	HOH73	0.87	-15	0.215	1.05	-80	0.195	1.92	-95	0.169	Neither	19
	HOH74	1.32	-198	-0.192	2.38	-122	-0.021	3.70	-319	-0.483	Neither	0
	HOH79	1.15	-163	-0.113	2.36	39	0.416	3.51	-124	-0.023	Protein B	9
	HOH80	0.00	-108	-0.040	0.98	-12	0.237	0.98	-119	-0.017	Neither	0
	HOH106	1.10	-145	-0.074	1.15	-409	-0.716	2.26	-554	-1.127	Neither	16
	HOH110	0.92	-108	0.148	1.25	-233	-0.273	2.17	-341	-0.537	Neither	31
3DLQ	HOH181	2.90	-13	0.431	2.45	171	0.580	5.35	158	0.850	Both	0
	HOH182	2.22	-249	-0.311	1.14	139	0.368	3.36	-110	0.009	Protein B	1
	HOH185	1.05	-214	-0.228	0.97	-83	0.178	2.01	-296	-0.426	Neither	2
	HOH186	0.00	-73	-0.040	0.74	25	0.222	0.74	-48	0.166	Neither	4
	HOH198	0.00	-237	-0.252	0.71	-102	0.118	0.71	-339	-0.531	Neither	21
	HOH220	2.73	-322	-0.488	1.37	16	0.296	4.10	-306	-0.449	Protein B	0
	HOH243	1.14	-45	0.231	0.00	-203	-0.168	1.14	-248	-0.309	Neither	2
	HOH248	1.09	56	0.307	2.03	76	0.409	3.12	131	0.610	Both	0
	HOH249	1.07	-15	0.250	0.99	-172	-0.133	2.05	-186	-0.166	Protein A	0
	HOH258	0.99	15	0.259	0.89	-460	-0.855	1.88	-445	-0.813	Protein A	33
	HOH236	1.11	275	0.401	1.15	-168	-0.125	2.26	106	0.476	Protein A	31
	HOH237	1.39	92	0.381	4.49	198	0.864	5.88	290	0.954	Both	17
	HOH240	2.44	141	0.543	1.41	-79	0.181	3.85	63	0.627	Protein A	2
	HOH252	2.07	-171	-0.132	1.25	65	0.339	3.32	-106	0.384	Protein B	9
	HOH267	0.94	320	0.389	1.10	-147	-0.078	2.04	172	0.526	Protein A	6
	HOH268	1.06	48	0.296	1.09	-80	0.202	2.15	-32	0.297	Protein A	42

	HOH271	1.32	224	0.446	2.25	-117	-0.011	3.57	107	0.628	Protein A	3
	HOH279	0.00	-101	-0.040	2.40	232	0.615	2.40	131	0.524	Protein B	1
	HOH281	1.12	30	0.291	1.18	20	0.290	2.30	50	0.419	Both	29
	HOH340	2.06	-147	-0.079	1.06	95	0.327	3.12	-52	0.416	Protein B	18
	HOH351	0.00	-350	-0.549	0.88	194	0.305	0.88	-156	-0.097	Protein B	15
	HOH352	3.97	-63	0.521	1.40	-100	0.153	5.37	-164	-0.112	Protein A	1
	HOH354	1.00	-58	0.205	1.02	193	0.338	2.02	135	0.482	Protein B	9
	HOH372	0.00	-175	-0.107	0.76	-4	0.205	0.76	-179	-0.149	Neither	27
	HOH401	0.00	-293	-0.397	1.07	526	0.490	1.07	233	0.365	Protein B	41
	HOH403	0.85	-160	-0.107	0.85	1	0.224	1.69	-159	-0.105	Neither	22
3EGG	HOH408	3.01	479	0.812	2.52	11	0.408	5.52	490	1.007	Both	7
	HOH411	1.04	-42	0.225	0.91	-87	0.166	1.96	-128	-0.038	Neither	15
	HOH435	1.07	-144	-0.071	0.00	-56	-0.039	1.07	-200	-0.196	Neither	0
	HOH438	0.00	-164	-0.087	0.71	16	0.211	0.71	-148	-0.080	Neither	35
	HOH454	2.66	532	0.800	1.28	-25	0.249	3.94	507	0.909	Protein A	27
	HOH459	1.23	95	0.361	3.42	-59	0.449	4.64	36	0.655	Both	4
	HOH470	1.15	-84	0.196	1.10	-23	0.250	2.25	-107	0.220	Protein B	9
	HOH479	1.19	92	0.354	0.89	33	0.255	2.08	125	0.477	Both	8
	HOH482	1.98	388	0.695	0.00	-114	-0.031	1.98	275	0.603	Protein A	11
	HOH488	1.23	-198	-0.192	0.85	1	0.225	2.09	-196	-0.189	Neither	47
	HOH497	2.18	-38	0.294	1.02	-108	0.167	3.20	-146	-0.073	Protein A	46
	HOH517	1.00	324	0.404	1.00	-367	-0.604	2.00	-43	0.249	Protein A	39
	HOH519	2.34	-129	-0.037	1.21	-9	0.265	3.55	-137	-0.054	Protein B	0
	HOH520	0.00	-113	-0.030	3.63	181	0.750	3.63	69	0.600	Protein B	27
	HOH534	4.03	29	0.609	1.21	-100	0.173	5.24	-71	0.587	Protein A	9
	HOH535	1.13	202	0.365	1.18	-115	-0.009	2.30	86	0.459	Protein A	3
	HOH536	2.63	351	0.711	1.32	-429	-0.771	3.95	-78	0.504	Protein A	0
	HOH550	1.29	193	0.422	2.27	-378	-0.634	3.56	-185	-0.162	Protein A	15
	HOH555	1.91	90	0.416	0.00	5	-0.039	1.91	94	0.422	Protein A	3
	HOH557	1.21	105	0.367	1.09	-7	0.260	2.30	98	0.472	Both	5
	HOH430	3.51	-39	0.482	0.00	-57	-0.039	3.51	-96	0.424	Protein A	1
	HOH468	1.34	14	0.292	2.34	-109	0.005	3.68	-96	0.452	Protein A	4
	HOH496	0.97	-14	0.235	0.00	-406	-0.701	0.97	-420	-0.744	Neither	0
	HOH498	0.90	-194	-0.183	1.28	122	0.394	2.19	-73	0.251	Protein B	32
	HOH502	2.46	116	0.512	0.87	-56	0.183	3.32	60	0.553	Protein A	4
	HOH504	0.91	-174	-0.137	1.09	52	0.303	2.00	-122	-0.025	Protein B	10
	HOH547	2.04	427	0.720	0.00	-94	-0.040	2.04	334	0.654	Protein A	29
	HOH554	0.88	-23	0.212	1.16	-119	-0.015	2.04	-141	-0.066	Neither	16
	HOH594	1.08	221	0.360	1.14	-31	0.244	2.22	190	0.566	Protein A	9
	HOH595	1.11	162	0.361	1.13	-204	-0.206	2.24	-42	0.301	Protein A	1
	HOH688	2.37	-63	0.300	1.22	146	0.393	3.59	83	0.607	Both	1
	HOH689	2.58	116	0.527	2.62	-7	0.403	5.21	108	0.748	Both	23
	HOH597	1.07	356	0.432	2.33	-74	0.278	3.39	282	0.763	Both	32
	HOH614	1.06	98	0.331	0.00	-531	-1.057	1.06	-434	-0.782	Protein A	10
	HOH615	1.12	-19	0.253	2.18	39	0.388	3.29	20	0.511	Both	36
	HOH628	1.18	57	0.322	0.97	-262	-0.342	2.15	-204	-0.207	Protein A	10
	HOH629	0.88	-22	0.212	0.87	-23	0.210	1.75	-45	0.234	Neither	43
	HOH640	1.00	-50	0.211	0.00	-484	-0.920	1.00	-534	-1.069	Neither	12
	HOH641	0.96	-221	-0.245	1.03	-114	-0.005	1.99	-335	-0.522	Neither	0
3EGV	HOH1	1.92	192	0.529	0.00	-272	-0.342	1.92	-80	0.190	Protein A	25
	HOH2	2.07	-70	0.228	0.00	-167	-0.092	2.07	-237	-0.282	Neither	14
	HOH23	0.00	-247	-0.277	0.00	-254	-0.296	0.00	-501	-0.968	Neither	2
	HOH45	1.28	109	0.383	2.20	-114	-0.007	3.48	-6	0.511	Protein A	33
	HOH51	3.30	-194	-0.182	0.00	80	-0.038	3.30	-114	-0.001	Neither	10
	HOH71	1.22	411	0.533	1.05	52	0.296	2.27	462	0.742	Both	50
	HOH74	1.07	-272	-0.367	0.93	113	0.317	2.00	-160	-0.107	Protein B	9
	HOH82	1.20	524	0.562	1.04	-21	0.240	2.23	502	0.760	Protein A	36
	HOH89	1.99	-182	-0.156	0.00	-310	-0.442	1.99	-491	-0.943	Neither	0
	HOH98	1.93	106	0.437	0.00	-280	-0.363	1.93	-174	-0.139	Protein A	6
	HOH100	1.06	355	0.431	0.00	-152	-0.068	1.06	203	0.346	Protein A	44

	HOH115	3.32	27	0.522	0.00	-221	-0.211	3.32	-194	-0.183	Protein A	0
	HOH122	1.10	211	0.356	0.99	-79	0.185	2.09	132	0.486	Protein A	9
	HOH130	0.00	-167	-0.093	0.83	-283	-0.392	0.83	-450	-0.827	Neither	3
	HOH144	1.11	320	0.426	1.05	57	0.301	2.16	377	0.688	Both	22
	HOH149	2.28	-36	0.316	1.23	-256	-0.328	3.50	-292	-0.414	Protein A	0
	HOH174	1.18	178	0.387	1.11	148	0.361	2.30	326	0.663	Both	6
	HOH178	2.13	192	0.554	0.00	-255	-0.299	2.13	-63	0.250	Protein A	9
	HOH242	0.96	-23	0.226	0.80	-247	-0.306	1.77	-270	-0.360	Neither	18
	HOH270	1.07	185	0.349	0.00	-284	-0.375	1.07	-100	0.181	Protein A	57
	HOH280	2.44	35	0.424	1.29	138	0.407	3.74	173	0.754	Both	12
	HOH292	0.00	-221	-0.211	0.79	200	0.282	0.79	-21	0.197	Protein B	0
	HOH322	4.23	-351	-0.564	1.45	250	0.503	5.67	-101	0.574	Protein B	6
	HOH332	0.97	-295	-0.421	0.00	-55	-0.039	0.97	-350	-0.560	Neither	24
	HOH338	1.23	212	0.408	1.18	117	0.368	2.41	329	0.675	Both	3
	HOH343	1.69	38	0.339	0.00	-245	-0.271	1.69	-206	-0.211	Protein A	1
	HOH348	0.00	-221	-0.212	0.00	-152	-0.067	0.00	-373	-0.611	Neither	6
	HOH362	0.91	-70	0.180	1.21	-6	0.268	2.12	-76	0.233	Protein B	3
	HOH363	1.11	-50	0.228	2.35	-144	-0.071	3.45	-194	-0.182	Neither	59
	HOH364	0.00	-141	-0.053	2.21	-106	0.213	2.21	-248	-0.308	Neither	23
	HOH370	1.08	-67	0.210	1.08	149	0.354	2.16	82	0.435	Protein B	23
	HOH373	0.00	-197	-0.154	1.93	-152	-0.090	1.93	-349	-0.558	Neither	30
	HOH374	0.00	5	-0.039	2.41	-76	0.291	2.41	-72	0.297	Protein B	27
	HOH377	1.08	-177	-0.143	2.51	89	0.488	3.59	-88	0.445	Protein B	21
	HOH382	1.11	62	0.315	1.11	-438	-0.793	2.22	-375	-0.626	Protein A	1
	HOH386	0.85	104	0.297	1.15	128	0.367	2.00	231	0.567	Both	14
	HOH398	0.89	-391	-0.668	1.04	252	0.369	1.93	-139	-0.061	Protein B	21
	HOH399	0.95	-145	-0.073	1.04	-109	0.169	1.99	-254	-0.323	Neither	41
	HOH402	0.92	30	0.258	1.12	-108	0.178	2.04	-78	0.208	Protein A	1
	HOH406	0.00	-329	-0.494	0.83	-87	0.151	0.83	-417	-0.737	Neither	33
	HOH411	0.98	29	0.269	1.05	100	0.331	2.03	130	0.476	Both	6
	HOH413	1.28	-83	0.185	4.16	-5	0.589	5.44	-88	0.579	Protein B	13
3EVS	HOH13	1.15	-15	0.258	3.95	-86	0.497	5.10	-101	0.552	Both	3
	HOH27	0.93	26	0.257	1.10	194	0.354	2.02	220	0.560	Both	16
	HOH37	0.81	11	0.225	0.85	-121	-0.021	1.66	-111	0.000	Neither	26
	HOH39	0.95	11	0.249	0.95	198	0.321	1.90	209	0.536	Protein B	20
3F62	HOH5	4.47	277	0.873	1.41	71	0.359	5.88	348	0.969	Both	0
	HOH134	2.73	-123	-0.023	1.04	-165	-0.117	3.76	-288	-0.404	Neither	10
	HOH142	1.14	333	0.448	1.08	-86	0.195	2.22	247	0.601	Protein A	19
	HOH144	2.38	614	0.809	0.97	-56	0.200	3.34	558	0.871	Protein A	2
	HOH145	1.36	-78	0.184	2.53	15	0.413	3.89	-63	0.514	Protein B	15
	HOH167	1.99	63	0.387	0.00	-277	-0.357	1.99	-214	-0.230	Protein A	23
	HOH175	2.25	89	0.456	1.07	-43	0.227	3.32	46	0.539	Protein A	11
	HOH176	2.21	356	0.676	1.23	-25	0.249	3.44	330	0.789	Protein A	12
	HOH179	0.00	-76	-0.040	1.86	-76	0.196	1.86	-151	-0.089	Neither	1
	HOH181	0.00	-7	-0.039	3.45	-264	-0.346	3.45	-270	-0.362	Neither	57
	HOH185	0.00	-240	-0.260	0.81	35	0.243	0.81	-205	-0.208	Neither	29
	HOH198	0.00	219	-0.039	0.91	-197	-0.189	0.91	22	0.252	Neither	0
	HOH201	0.00	-478	-0.903	5.13	-47	0.605	5.13	-525	-1.042	Protein B	0
3F75	HOH229	2.43	118	0.512	2.75	56	0.485	5.18	174	0.875	Both	7
	HOH237	3.43	75	0.579	1.12	-1	0.268	4.55	73	0.681	Both	31
	HOH238	3.84	145	0.723	0.00	-54	-0.039	3.84	91	0.648	Protein A	38
	HOH239	1.34	483	0.629	2.33	-34	0.328	3.68	449	0.864	Both	26
	HOH248	3.50	-41	0.479	1.24	-38	0.236	4.74	-79	0.555	Protein A	18
	HOH253	3.86	80	0.643	1.19	49	0.316	5.05	129	0.776	Both	30
	HOH270	2.23	390	0.700	0.00	-47	-0.039	2.23	343	0.669	Protein A	33
	HOH273	2.31	-123	-0.025	2.62	93	0.506	4.93	-30	0.612	Protein B	0
	HOH275	2.11	487	0.753	0.00	-35	-0.039	2.11	452	0.733	Protein A	1
	HOH282	0.96	421	0.438	2.18	96	0.452	3.14	516	0.839	Both	0
	HOH288	2.25	425	0.721	0.98	39	0.276	3.23	464	0.827	Both	20
	HOH292	1.27	91	0.365	2.39	-136	-0.054	3.65	-45	0.499	Protein A	17

	HOH298	3.75	321	0.820	1.24	-65	0.208	4.99	256	0.907	Protein A	0
	HOH302	0.00	50	-0.038	1.87	74	0.395	1.87	124	0.456	Protein B	11
	HOH303	3.34	121	0.620	0.00	-84	-0.040	3.34	37	0.534	Protein A	11
	HOH308	2.01	621	0.820	0.00	2	-0.039	2.01	623	0.821	Protein A	0
	HOH312	0.94	-66	0.187	0.89	-56	0.187	1.83	-123	-0.026	Neither	16
	HOH1	1.20	-35	0.240	3.67	11	0.554	4.87	-24	0.614	Protein B	0
	HOH24	0.00	-187	-0.132	2.16	46	0.395	2.16	-141	-0.065	Protein B	36
	HOH69	0.86	-153	-0.090	1.17	36	0.302	2.02	-117	-0.014	Protein B	7
	HOH86	0.99	-17	0.234	1.19	148	0.385	2.17	131	0.496	Protein B	6
	HOH91	1.13	-86	0.197	2.50	169	0.584	3.62	83	0.611	Protein B	15
	HOH201	2.51	374	0.712	1.40	155	0.436	3.91	529	0.913	Both	7
	HOH205	1.03	72	0.307	1.11	105	0.343	2.14	176	0.541	Both	45
	HOH207	0.00	-173	-0.102	2.12	171	0.534	2.12	-2	0.328	Protein B	20
	HOH213	0.00	7	-0.039	1.01	-86	0.183	1.01	-79	0.189	Neither	26
	HOH218	0.00	-97	-0.040	0.00	-13	-0.039	0.00	-110	-0.029	Neither	24
	HOH219	0.00	-122	-0.036	1.05	-25	0.239	1.05	-147	-0.078	Neither	9
3FFD	HOH225	2.34	220	0.601	1.04	-153	-0.090	3.39	67	0.567	Protein A	0
	HOH258	1.24	137	0.394	0.99	118	0.328	2.22	255	0.607	Both	1
	HOH276	0.92	26	0.255	0.00	-128	-0.040	0.92	-102	0.154	Protein A	26
	HOH223	4.27	302	0.863	1.12	-5	0.264	5.39	297	0.935	Both	7
	HOH239	1.29	83	0.360	2.29	-166	-0.120	3.58	-83	0.448	Protein A	30
	HOH266	0.83	-77	0.159	0.00	18	-0.039	0.83	-59	0.174	Neither	0
	HOH109	1.31	27	0.305	3.73	45	0.593	5.04	71	0.706	Both	0
	HOH110	2.08	138	0.492	1.17	-115	-0.008	3.25	23	0.509	Protein A	4
	HOH111	1.05	-9	0.251	1.15	18	0.285	2.19	9	0.355	Both	34
	HOH126	0.00	-165	-0.088	3.59	493	0.872	3.59	328	0.804	Protein B	17
	HOH188	0.84	-20	0.207	0.98	-27	0.225	1.82	-47	0.234	Neither	31
3FHI	HOH369	1.34	240	0.466	2.24	-82	0.249	3.57	157	0.706	Protein A	1
	HOH381	5.19	351	0.941	0.00	-242	-0.265	5.19	109	0.749	Protein A	0
	HOH404	3.25	21	0.507	0.00	-262	-0.318	3.25	-242	-0.293	Protein A	11
	HOH417	1.28	-167	-0.123	2.48	-285	-0.398	3.76	-452	-0.834	Neither	0
	HOH419	2.35	41	0.417	1.07	-209	-0.216	3.42	-168	-0.123	Protein A	0
	HOH426	2.01	244	0.579	0.00	-69	-0.039	2.01	176	0.525	Protein A	5
	HOH431	1.12	16	0.281	3.72	-152	-0.085	4.84	-136	-0.048	Protein A	2
	HOH451	4.04	477	0.907	0.00	-19	-0.039	4.04	457	0.900	Protein A	2
	HOH455	2.34	124	0.509	1.24	-99	0.170	3.59	25	0.555	Protein A	13
	HOH456	2.50	394	0.722	1.31	-156	-0.098	3.81	238	0.805	Protein A	12
	HOH458	0.00	-313	-0.451	1.04	21	0.273	1.04	-292	-0.414	Protein B	29
	HOH459	0.00	-206	-0.175	1.08	-476	-0.899	1.08	-682	-1.523	Neither	19
	HOH460	1.17	21	0.289	2.33	-338	-0.530	3.50	-318	-0.478	Protein A	5
	HOH495	3.95	10	0.587	1.51	-138	-0.058	5.46	-128	-0.028	Protein A	0
	HOH496	0.83	35	0.246	0.00	-156	-0.074	0.83	-121	-0.022	Neither	5
	HOH497	1.99	-122	-0.025	1.08	-61	0.215	3.07	-183	-0.157	Neither	18
	HOH499	0.00	-120	-0.034	1.78	21	0.323	1.78	-99	0.159	Protein B	25
	HOH500	1.43	-68	0.194	2.43	31	0.418	3.86	-37	0.540	Protein B	24
3G5O	HOH117	2.19	-47	0.284	0.00	-61	-0.039	2.19	-109	0.203	Protein A	12
	HOH103	1.17	-39	0.237	1.22	-65	0.209	2.38	-104	0.252	Neither	44
	HOH108	0.87	-14	0.217	1.04	-49	0.218	1.92	-64	0.214	Neither	26
	HOH113	0.00	-142	-0.055	0.76	-26	0.188	0.76	-169	-0.126	Neither	13
	HOH126	0.87	-301	-0.437	0.87	-119	-0.016	1.74	-419	-0.744	Neither	9
	HOH129	0.00	-139	-0.050	1.05	-11	0.249	1.05	-150	-0.083	Neither	16
	HOH132	0.91	-145	-0.074	1.14	395	0.479	2.04	250	0.586	Protein B	15
	HOH136	0.00	-201	-0.163	1.75	-30	0.254	1.75	-231	-0.269	Protein B	28
	HOH139	0.00	-19	-0.039	2.42	332	0.678	2.42	313	0.667	Protein B	16
	HOH149	0.88	-69	0.175	1.18	212	0.390	2.07	143	0.496	Protein B	14
	HOH118	2.46	22	0.412	1.04	-192	-0.179	3.50	-170	-0.127	Protein A	20
	HOH123	3.35	690	0.907	0.00	-204	-0.170	3.35	486	0.847	Protein A	25
	HOH131	3.51	233	0.764	0.00	-32	-0.039	3.51	201	0.757	Protein A	35
	HOH145	0.97	31	0.268	0.00	-83	-0.040	0.97	-51	0.204	Protein A	66
	HOH147	0.92	359	0.403	0.00	-37	-0.039	0.92	322	0.385	Protein A	12

	HOH148	2.18	-89	0.228	1.20	197	0.392	3.38	108	0.607	Protein B	22
	HOH166	1.12	308	0.424	1.03	83	0.315	2.15	391	0.697	Both	40
3GEW	HOH234	1.23	14	0.288	2.32	-385	-0.652	3.55	-372	-0.617	Protein A	8
	HOH235	1.11	-136	-0.053	3.69	39	0.583	4.80	-97	0.541	Protein B	1
	HOH243	1.17	-329	-0.506	2.46	-508	-0.990	3.63	-836	-2.047	Neither	3
	HOH248	2.32	-77	0.274	1.39	-240	-0.291	3.72	-317	-0.477	Protein A	0
	HOH249	0.00	-52	-0.039	0.00	-450	-0.822	0.00	-501	-0.970	Neither	1
	HOH254	0.75	-90	0.135	0.98	-90	0.175	1.74	-180	-0.151	Neither	13
	HOH259	1.21	-249	-0.311	1.22	-299	-0.433	2.43	-549	-1.111	Neither	0
	HOH285	1.54	85	0.385	4.11	-2	0.588	5.65	83	0.740	Both	41
	HOH521	1.14	-154	-0.092	4.14	324	0.859	5.28	170	0.872	Protein B	27
	HOH522	0.82	-24	0.201	0.99	-266	-0.350	1.81	-289	-0.408	Neither	39
	HOH527	2.01	-7	0.299	1.17	45	0.311	3.18	38	0.516	Both	0
	HOH579	1.17	87	0.346	1.19	-488	-0.933	2.37	-401	-0.693	Protein A	0
	HOH580	0.94	-146	-0.075	1.21	-194	-0.182	2.15	-339	-0.533	Neither	7
	HOH584	0.92	-35	0.208	0.95	-42	0.209	1.87	-78	0.193	Neither	25
	HOH636	0.93	-242	-0.294	0.91	-17	0.221	1.84	-258	-0.334	Neither	12
	HOH686	1.14	-151	-0.087	1.25	513	0.590	2.38	362	0.693	Protein B	59
	HOH268	2.33	-5	0.363	1.07	39	0.290	3.40	35	0.539	Both	21
	HOH271	1.27	167	0.412	1.18	171	0.387	2.45	338	0.685	Both	0
	HOH284	1.26	187	0.412	0.91	-197	-0.190	2.17	-11	0.328	Protein A	6
	HOH547	0.85	98	0.295	0.93	-270	-0.360	1.78	-172	-0.135	Protein A	4
	HOH565	0.97	-202	-0.202	0.84	151	0.307	1.81	-51	0.228	Protein B	14
	HOH582	2.34	-26	0.340	1.03	-239	-0.286	3.38	-264	-0.347	Protein A	12
	HOH650	1.27	71	0.345	2.41	102	0.490	3.67	173	0.745	Both	9
	HOH562	1.12	191	0.363	0.81	49	0.253	1.93	240	0.567	Both	19
3GMW	HOH1	4.50	267	0.873	1.39	-45	0.224	5.89	222	0.955	Protein A	0
	HOH15	1.19	74	0.337	0.87	4	0.231	2.06	78	0.416	Protein A	41
	HOH35	2.18	-103	0.210	1.23	371	0.517	3.41	268	0.760	Protein B	45
	HOH44	1.07	78	0.318	1.89	1	0.302	2.96	78	0.529	Both	21
	HOH54	1.26	418	0.557	0.87	-171	-0.131	2.13	247	0.592	Protein A	41
	HOH104	1.07	62	0.308	2.16	44	0.391	3.23	105	0.586	Both	8
	HOH130	0.91	127	0.318	0.97	73	0.297	1.87	200	0.528	Both	10
	HOH161	0.00	-19	-0.039	0.93	52	0.276	0.93	33	0.262	Protein B	13
	HOH164	1.35	-231	-0.269	2.51	74	0.473	3.86	-157	-0.098	Protein B	0
	HOH165	0.00	-197	-0.153	2.25	173	0.554	2.25	-23	0.327	Protein B	11
	HOH166	0.93	224	0.328	0.99	-135	-0.050	1.92	90	0.416	Protein A	4
	HOH174	2.47	-480	-0.912	2.62	-127	-0.032	5.08	-607	-1.287	Neither	26
	HOH177	1.09	55	0.306	3.52	-1	0.521	4.61	54	0.669	Both	20
	HOH180	0.00	-423	-0.749	2.08	-405	-0.706	2.08	-829	-2.020	Neither	28
3GRW	HOH2	4.12	259	0.839	1.13	-27	0.247	5.25	232	0.923	Protein A	20
	HOH5	3.88	-818	-1.981	1.38	160	0.434	5.26	-658	-1.446	Protein B	12
	HOH6	2.47	-75	0.303	2.41	-86	0.278	4.88	-161	-0.106	Both	14
	HOH9	3.35	117	0.614	0.00	-168	-0.093	3.35	-51	0.447	Protein A	29
	HOH10	2.58	-26	0.376	1.06	-66	0.207	3.64	-93	0.448	Protein A	8
	HOH14	2.47	572	0.799	2.61	-68	0.332	5.08	504	0.990	Both	42
	HOH15	1.08	-98	0.185	0.83	-36	0.192	1.91	-134	-0.051	Neither	0
	HOH28	2.45	115	0.510	1.32	58	0.338	3.77	173	0.757	Both	2
	HOH36	2.13	-68	0.243	0.99	-23	0.231	3.12	-91	0.373	Neither	0
	HOH37	0.94	-110	0.004	0.00	-138	-0.050	0.94	-248	-0.309	Neither	0
	HOH38	0.86	19	0.240	0.00	-223	-0.217	0.86	-204	-0.207	Neither	5
	HOH41	2.46	-28	0.356	1.20	47	0.315	3.66	19	0.560	Both	14
	HOH42	1.08	16	0.275	0.00	-260	-0.312	1.08	-245	-0.300	Protein A	3
	HOH43	0.94	-164	-0.116	0.00	-194	-0.148	0.94	-359	-0.583	Neither	7
	HOH46	1.12	235	0.382	1.12	-29	0.245	2.24	206	0.579	Protein A	26
	HOH78	1.15	87	0.340	1.16	-373	-0.621	2.30	-286	-0.401	Protein A	46
	HOH87	0.00	-26	-0.039	1.88	-79	0.192	1.88	-105	0.154	Neither	8
	HOH102	1.33	178	0.430	4.14	-466	-0.873	5.47	-288	-0.406	Protein A	10
	HOH116	1.11	87	0.332	2.31	-268	-0.357	3.43	-181	-0.154	Protein A	11
	HOH126	0.92	-62	0.186	1.09	-40	0.235	2.01	-102	0.165	Neither	2

	HOH160	2.16	-168	-0.125	1.16	-54	0.223	3.32	-222	-0.247	Neither	6
	HOH166	2.65	-103	0.296	2.89	35	0.479	5.54	-68	0.601	Both	31
	HOH176	2.31	-136	-0.055	2.80	154	0.605	5.11	17	0.663	Protein B	22
	HOH180	3.57	33	0.560	1.34	263	0.487	4.92	296	0.909	Both	24
	HOH204	1.28	98	0.373	2.47	134	0.538	3.75	233	0.796	Both	0
	HOH205	0.00	-197	-0.153	4.74	176	0.849	4.74	-21	0.610	Protein B	0
	HOH218	1.20	73	0.338	3.54	-53	0.473	4.74	20	0.647	Both	3
	HOH241	0.85	19	0.238	1.02	-46	0.218	1.88	-28	0.263	Neither	26
	HOH253	0.00	-117	-0.032	1.02	-6	0.248	1.02	-123	-0.024	Neither	0
3HCT	HOH16	3.90	-237	-0.282	1.24	68	0.339	5.13	-170	-0.126	Protein B	25
	HOH25	2.12	14	0.347	0.00	-267	-0.329	2.12	-253	-0.321	Protein A	1
	HOH39	2.15	123	0.482	0.00	-85	-0.040	2.15	38	0.382	Protein A	16
	HOH40	0.00	-150	-0.065	0.73	35	0.228	0.73	-115	-0.009	Neither	5
	HOH63	0.00	-177	-0.110	0.00	-318	-0.465	0.00	-495	-0.951	Neither	22
	HOH64	0.75	-318	-0.480	0.00	-159	-0.078	0.75	-477	-0.903	Neither	21
	HOH74	0.99	338	0.407	0.80	-23	0.197	1.78	315	0.613	Protein A	30
	HOH102	0.86	-179	-0.149	1.10	46	0.301	1.97	-133	-0.049	Protein B	6
	HOH123	1.19	-418	-0.739	1.22	161	0.397	2.41	-256	-0.328	Protein B	35
	HOH124	1.42	222	0.472	2.64	275	0.669	4.06	496	0.916	Both	27
	HOH169	1.11	131	0.356	1.14	77	0.331	2.25	207	0.581	Both	1
	HOH192	1.07	89	0.326	1.07	-162	-0.110	2.14	-73	0.242	Protein A	37
3HEI	HOH438	1.01	-8	0.246	0.00	-180	-0.118	1.01	-188	-0.169	Neither	4
	HOH450	2.35	258	0.623	0.95	-23	0.223	3.29	235	0.736	Protein A	26
	HOH456	0.78	-384	-0.649	0.00	-190	-0.139	0.78	-574	-1.186	Neither	15
	HOH457	3.91	-84	0.496	0.00	-214	-0.195	3.91	-299	-0.431	Protein A	0
	HOH464	2.09	-253	-0.321	0.00	-74	-0.040	2.09	-327	-0.502	Neither	1
	HOH466	3.79	-228	-0.261	1.46	-2	0.277	5.25	-231	-0.267	Protein B	31
	HOH478	1.34	-391	-0.669	2.42	17	0.401	3.76	-374	-0.624	Protein B	27
	HOH485	1.00	-11	0.241	0.96	212	0.326	1.96	202	0.540	Protein B	4
	HOH495	3.70	369	0.834	1.18	20	0.289	4.88	388	0.937	Both	19
	HOH502	1.22	331	0.488	0.88	-227	-0.259	2.10	104	0.452	Protein A	0
	HOH526	2.44	50	0.439	1.16	65	0.326	3.60	115	0.643	Both	2
	HOH550	2.69	-50	0.364	1.12	-52	0.226	3.81	-102	0.468	Protein A	1
	HOH567	1.17	-28	0.246	0.97	-40	0.214	2.14	-68	0.248	Neither	1
	HOH586	1.04	74	0.310	0.00	-261	-0.314	1.04	-187	-0.168	Protein A	23
	HOH593	0.92	5	0.240	0.97	-75	0.185	1.89	-70	0.205	Neither	11
	HOH597	2.08	128	0.479	0.00	-48	-0.039	2.08	80	0.420	Protein A	6
	HOH654	0.00	-143	-0.055	0.88	-27	0.208	0.88	-169	-0.127	Neither	12
	HOH661	1.00	-491	-0.942	3.30	-60	0.432	4.30	-550	-1.116	Protein B	9
	HOH663	1.26	-36	0.237	2.57	94	0.501	3.83	58	0.619	Protein B	19
	HOH668	0.00	-245	-0.272	3.14	25	0.498	3.14	-220	-0.241	Protein B	34
	HOH684	0.95	-13	0.232	2.14	21	0.360	3.09	8	0.475	Protein B	22
	HOH694	1.06	-72	0.203	1.12	-2	0.267	2.17	-73	0.247	Protein B	8
	HOH695	1.04	76	0.312	1.98	-121	-0.023	3.02	-45	0.411	Protein A	2
	HOH718	0.00	-164	-0.086	2.04	297	0.624	2.04	133	0.481	Protein B	0
	HOH721	1.10	-219	-0.239	2.22	81	0.442	3.31	-137	-0.054	Protein B	0
	HOH725	0.00	-155	-0.073	1.83	-63	0.213	1.83	-218	-0.239	Neither	7
	HOH730	1.13	-23	0.251	1.04	-146	-0.074	2.17	-168	-0.125	Protein A	12
	HOH770	0.00	-145	-0.058	0.74	74	0.259	0.74	-71	0.149	Protein B	7
	HOH774	0.00	-154	-0.070	0.84	14	0.233	0.84	-140	-0.061	Neither	16
	HOH798	1.01	19	0.266	2.16	-12	0.325	3.17	8	0.485	Both	21
	HOH807	0.00	-34	-0.039	0.78	13	0.221	0.78	-20	0.195	Neither	4
	HOH2515	1.11	-21	0.251	1.05	-142	-0.065	2.16	-163	-0.114	Protein A	2
	HOH2529	1.02	199	0.336	0.00	-120	-0.034	1.02	80	0.311	Protein A	21
	HOH2568	3.55	650	0.915	0.00	-288	-0.385	3.55	362	0.815	Protein A	34
	HOH2570	2.42	454	0.746	1.31	-247	-0.307	3.73	206	0.794	Protein A	40
	HOH2572	2.49	123	0.526	2.19	-233	-0.274	4.68	-110	0.012	Protein A	32
	HOH2641	0.00	-168	-0.094	0.77	18	0.223	0.77	-150	-0.086	Neither	41
3HG0	HOH38	1.11	-211	-0.222	0.94	38	0.268	2.05	-173	-0.137	Protein B	34
	HOH51	0.93	-49	0.198	0.00	-244	-0.270	0.93	-294	-0.419	Neither	17

	HOH56	0.82	-9	0.211	0.00	-264	-0.321	0.82	-273	-0.367	Neither	3
	HOH101	2.18	-126	-0.033	1.05	18	0.272	3.23	-108	0.369	Protein B	31
	HOH180	2.03	135	0.482	0.00	-87	-0.040	2.03	48	0.374	Protein A	46
	HOH186	2.47	204	0.612	1.01	-208	-0.214	3.47	-3	0.512	Protein A	0
	HOH326	3.23	32	0.516	0.00	18	-0.039	3.23	51	0.534	Protein A	45
	HOH361	1.01	11	0.260	2.01	68	0.397	3.02	80	0.538	Both	14
	HOH364	2.61	-151	-0.087	2.93	-2	0.445	5.53	-153	-0.088	Protein B	9
	HOH366	0.80	15	0.226	0.98	34	0.272	1.78	48	0.358	Protein B	17
	HOH370	1.05	-358	-0.582	1.09	62	0.311	2.15	-297	-0.426	Protein B	34
	HOH376	3.98	228	0.823	1.22	94	0.359	5.20	322	0.933	Both	12
	HOH381	1.01	34	0.277	1.03	228	0.353	2.04	262	0.596	Both	13
	HOH385	0.00	-137	-0.049	0.90	-201	-0.199	0.90	-338	-0.530	Neither	17
	HOH389	0.00	-61	-0.039	2.25	104	0.472	2.25	43	0.404	Protein B	48
3HH2	HOH8	1.25	-182	-0.156	1.19	148	0.384	2.44	-33	0.347	Protein B	9
	HOH35	2.66	117	0.538	2.67	49	0.467	5.33	166	0.865	Both	19
	HOH61	1.31	-54	0.216	2.20	170	0.544	3.51	117	0.634	Protein B	17
	HOH63	1.39	87	0.375	2.64	-450	-0.827	4.03	-363	-0.595	Protein A	18
	HOH72	1.03	154	0.345	1.98	-125	-0.031	3.01	29	0.487	Protein A	33
	HOH75	2.50	44	0.441	1.20	-68	0.207	3.70	-24	0.526	Protein A	28
	HOH77	0.97	-234	-0.276	0.00	-146	-0.059	0.97	-381	-0.640	Neither	26
	HOH83	2.31	306	0.651	1.05	12	0.266	3.36	317	0.774	Both	4
	HOH88	0.91	240	0.334	0.00	-521	-1.026	0.91	-281	-0.388	Protein A	23
	HOH92	5.00	-256	-0.326	0.00	-48	-0.039	5.00	-303	-0.442	Neither	27
	HOH94	1.08	-145	-0.073	0.98	-41	0.213	2.05	-187	-0.167	Neither	7
	HOH98	3.11	-67	0.398	0.00	-51	-0.039	3.11	-118	-0.012	Protein A	0
	HOH100	4.10	553	0.937	1.24	-16	0.259	5.33	537	1.014	Both	16
	HOH136	1.17	-18	0.255	3.67	-14	0.531	4.84	-32	0.605	Both	1
	HOH142	0.00	-73	-0.040	3.68	358	0.828	3.68	285	0.799	Protein B	9
	HOH146	0.00	-68	-0.039	2.03	-180	-0.152	2.03	-248	-0.310	Neither	11
	HOH158	0.00	-368	-0.597	1.04	-1	0.257	1.04	-369	-0.609	Protein B	9
	HOH177	1.09	19	0.280	2.49	-10	0.380	3.58	10	0.539	Both	32
	HOH178	2.23	-105	0.218	1.28	149	0.408	3.51	44	0.561	Protein B	37
	HOH188	1.18	-343	-0.544	1.24	318	0.488	2.42	-26	0.352	Protein B	31
	HOH208	0.91	-128	-0.037	1.18	8	0.279	2.09	-121	-0.021	Protein B	2
	HOH209	0.86	-10	0.218	0.90	-113	-0.002	1.76	-123	-0.026	Neither	6
	HOH223	0.00	-407	-0.702	0.00	-257	-0.304	0.00	-664	-1.465	Neither	15
3HY2	HOH14	0.96	-243	-0.296	0.00	-298	-0.412	0.96	-541	-1.088	Neither	0
	HOH24	2.20	227	0.587	1.06	-233	-0.273	3.26	-6	0.482	Protein A	18
	HOH41	1.16	175	0.377	1.10	112	0.345	2.25	287	0.633	Both	20
	HOH48	2.26	85	0.451	1.04	-114	-0.004	3.29	-29	0.462	Protein A	29
	HOH93	2.32	404	0.713	0.00	-70	-0.040	2.32	333	0.670	Protein A	18
	HOH101	1.20	-120	-0.019	1.15	-485	-0.925	2.36	-605	-1.281	Neither	18
	HOH109	1.23	253	0.438	1.20	-129	-0.038	2.43	124	0.519	Protein A	1
	HOH134	2.15	-164	-0.115	0.00	-238	-0.255	2.15	-402	-0.697	Neither	18
	HOH139	1.14	-89	0.193	0.89	8	0.236	2.03	-81	0.200	Neither	17
	HOH152	1.84	-54	0.225	0.00	-277	-0.357	1.84	-332	-0.514	Neither	0
	HOH162	3.65	-223	-0.249	0.00	-288	-0.384	3.65	-511	-1.000	Neither	0
	HOH210	0.83	-38	0.190	1.16	66	0.327	1.99	28	0.342	Protein B	0
	HOH211	1.01	516	0.475	0.99	2	0.249	1.99	518	0.775	Protein A	1
	HOH212	2.41	-181	-0.153	1.35	-370	-0.612	3.75	-550	-1.116	Neither	26
	HOH213	0.90	-170	-0.130	1.11	-249	-0.312	2.01	-420	-0.745	Neither	30
	HOH214	0.00	-238	-0.256	2.37	-245	-0.302	2.37	-484	-0.922	Neither	3
	HOH215	0.00	-41	-0.039	3.52	-79	0.442	3.52	-120	-0.014	Protein B	28
	HOH216	1.13	20	0.285	1.24	-10	0.264	2.38	10	0.386	Both	13
	HOH221	0.92	-14	0.224	1.26	406	0.553	2.18	392	0.698	Protein B	24
	HOH225	1.14	-220	-0.243	1.17	13	0.283	2.31	-207	-0.213	Protein B	22
	HOH226	1.03	7	0.261	2.16	-54	0.271	3.20	-47	0.431	Both	9
	HOH227	1.16	-311	-0.462	2.12	61	0.405	3.28	-250	-0.313	Protein B	29
	HOH228	0.92	-80	0.173	1.18	-87	0.190	2.10	-167	-0.123	Neither	14
	HOH238	0.95	-55	0.199	2.50	-53	0.334	3.46	-108	0.402	Protein B	5

	HOH240	0.00	45	-0.038	2.00	123	0.464	2.00	168	0.517	Protein B	34
	HOH244	0.91	-5	0.231	2.11	-238	-0.285	3.02	-243	-0.297	Neither	27
	HOH252	2.34	-100	0.247	1.26	337	0.512	3.60	237	0.776	Protein B	37
	HOH254	0.95	-350	-0.562	0.94	-17	0.227	1.89	-367	-0.605	Neither	22
	HOH258	1.87	-54	0.227	0.00	-214	-0.194	1.87	-268	-0.355	Neither	6
	HOH259	0.00	-192	-0.143	0.92	-283	-0.393	0.92	-475	-0.898	Neither	7
3HZH	HOH9	1.15	-165	-0.119	1.03	85	0.316	2.17	-81	0.238	Protein B	26
	HOH11	2.58	208	0.632	1.24	274	0.459	3.82	482	0.890	Both	2
	HOH12	2.40	590	0.802	0.89	-246	-0.303	3.29	344	0.779	Protein A	8
	HOH30	2.14	-169	-0.128	0.00	-234	-0.244	2.14	-403	-0.700	Neither	23
	HOH32	1.20	-421	-0.747	0.89	-63	0.181	2.10	-484	-0.923	Neither	0
	HOH41	1.20	-460	-0.856	1.08	-155	-0.095	2.29	-615	-1.313	Neither	2
	HOH43	1.04	97	0.327	0.88	-132	-0.045	1.92	-35	0.255	Protein A	19
	HOH50	1.33	79	0.360	0.78	49	0.248	2.11	128	0.483	Protein A	52
	HOH87	1.18	-602	-1.271	1.21	-204	-0.206	2.38	-806	-1.939	Neither	10
	HOH90	0.00	-247	-0.277	1.92	-18	0.278	1.92	-265	-0.349	Protein B	18
	HOH91	2.62	-386	-0.655	2.53	-329	-0.507	5.15	-715	-1.633	Neither	18
	HOH92	0.00	-85	-0.040	1.02	-166	-0.120	1.02	-251	-0.316	Neither	9
	HOH97	0.88	-491	-0.942	1.08	67	0.313	1.96	-424	-0.755	Protein B	27
	HOH107	0.00	-314	-0.453	1.17	-60	0.216	1.17	-374	-0.623	Neither	31
	HOH109	1.07	-93	0.186	1.17	-131	-0.043	2.23	-225	-0.254	Neither	3
	HOH110	2.00	22	0.337	1.24	-31	0.243	3.25	-9	0.477	Protein A	29
	HOH117	0.00	-347	-0.541	3.48	-399	-0.688	3.48	-745	-1.733	Neither	20
	HOH133	0.00	-189	-0.136	1.91	107	0.437	1.91	-82	0.188	Protein B	0
	HOH142	0.96	13	0.252	1.12	392	0.466	2.08	405	0.705	Both	28
	HOH155	2.23	-176	-0.143	2.60	131	0.549	4.83	-45	0.593	Protein B	3
3JZA	HOH1	0.97	263	0.362	0.00	-274	-0.349	0.97	-12	0.236	Protein A	19
	HOH5	2.84	65	0.503	2.30	-98	0.242	5.14	-34	0.618	Protein A	30
	HOH8	2.41	177	0.581	0.95	-110	0.005	3.36	67	0.564	Protein A	5
	HOH9	2.54	332	0.691	2.38	-280	-0.386	4.92	52	0.684	Protein A	5
	HOH12	2.53	-128	-0.036	2.61	361	0.715	5.14	233	0.916	Protein B	5
	HOH15	2.42	-112	0.001	1.39	511	0.659	3.80	400	0.857	Protein B	0
	HOH21	4.19	162	0.780	1.36	6	0.284	5.54	167	0.879	Both	26
	HOH23	1.01	92	0.318	0.93	98	0.309	1.95	190	0.530	Both	7
	HOH24	1.45	-400	-0.691	3.73	207	0.794	5.18	-193	-0.179	Protein B	27
	HOH27	2.62	58	0.470	2.62	-103	0.292	5.24	-46	0.611	Both	0
	HOH31	0.98	5	0.250	0.93	28	0.259	1.91	33	0.345	Both	15
	HOH39	2.21	159	0.533	0.00	-220	-0.210	2.21	-62	0.270	Protein A	39
	HOH48	2.64	47	0.461	0.97	-76	0.185	3.61	-29	0.507	Protein A	8
	HOH51	0.90	-92	0.159	0.00	-80	-0.040	0.90	-172	-0.132	Neither	6
	HOH65	3.25	380	0.791	0.00	29	-0.038	3.25	409	0.805	Protein A	5
	HOH75	2.62	-338	-0.530	1.11	9	0.274	3.72	-329	-0.507	Protein B	52
	HOH83	4.92	28	0.663	0.00	0	-0.039	4.92	28	0.663	Protein A	1
	HOH85	0.00	-210	-0.184	0.00	-92	-0.040	0.00	-302	-0.421	Neither	57
	HOH86	0.83	-40	0.189	0.89	-253	-0.320	1.72	-293	-0.417	Neither	0
	HOH88	3.75	10	0.566	1.27	-16	0.259	5.03	-6	0.638	Both	0
	HOH97	2.67	-365	-0.599	2.72	50	0.474	5.39	-315	-0.471	Protein B	0
	HOH103	0.84	-140	-0.062	1.01	-79	0.188	1.85	-219	-0.242	Neither	4
	HOH104	2.12	-145	-0.075	1.36	122	0.407	3.48	-23	0.494	Protein B	0
	HOH105	1.04	-180	-0.150	2.72	273	0.679	3.76	94	0.639	Protein B	39
	HOH106	1.33	-48	0.222	3.98	3	0.583	5.31	-45	0.615	Protein B	38
	HOH107	1.22	-29	0.246	2.49	230	0.627	3.72	201	0.787	Protein B	20
	HOH112	1.15	76	0.331	1.25	-243	-0.296	2.39	-167	-0.121	Protein A	10
	HOH123	0.00	-222	-0.213	0.00	-185	-0.128	0.00	-407	-0.702	Neither	30
	HOH126	1.91	-410	-0.720	1.03	22	0.271	2.94	-389	-0.661	Protein B	17
	HOH129	1.13	186	0.367	1.15	270	0.417	2.28	455	0.739	Both	1
	HOH136	1.30	26	0.303	2.42	465	0.752	3.72	490	0.883	Both	12
	HOH194	0.83	-176	-0.143	1.03	0	0.255	1.85	-176	-0.143	Protein B	0
	HOH201	1.02	-139	-0.059	2.22	-81	0.248	3.24	-219	-0.241	Neither	29
	HOH202	0.00	-129	-0.041	0.00	-50	-0.039	0.00	-179	-0.115	Neither	2

	HOH240	1.32	-101	0.159	3.47	-65	0.450	4.79	-166	-0.117	Protein B	0
	HOH241	0.00	-283	-0.371	2.35	-1453	-4.614	2.35	-1736	-6.045	Neither	0
	HOH245	2.20	99	0.458	1.24	254	0.443	3.43	353	0.798	Both	0
3K2M	HOH4	4.85	13	0.646	0.00	26	-0.038	4.85	38	0.669	Protein A	41
	HOH9	2.45	-342	-0.540	0.88	-202	-0.202	3.33	-544	-1.098	Neither	11
	HOH16	1.06	-11	0.251	0.96	-257	-0.330	2.02	-268	-0.356	Protein A	15
	HOH25	3.54	-215	-0.230	1.22	25	0.298	4.76	-190	-0.172	Protein B	17
	HOH38	1.21	19	0.292	1.04	28	0.277	2.25	47	0.409	Both	2
	HOH41	1.35	403	0.588	2.31	-13	0.349	3.66	390	0.838	Both	20
	HOH49	1.15	-52	0.225	1.13	25	0.289	2.28	-27	0.328	Protein B	24
	HOH63	4.02	106	0.675	1.29	5	0.281	5.30	111	0.757	Both	0
	HOH67	0.00	-215	-0.196	0.00	-14	-0.039	0.00	-229	-0.230	Neither	0
	HOH108	1.92	-27	0.266	1.22	-127	-0.034	3.14	-154	-0.091	Protein A	0
	HOH138	1.04	-7	0.251	2.54	-196	-0.188	3.57	-203	-0.202	Protein A	10
	HOH152	1.31	-17	0.257	2.40	535	0.781	3.71	518	0.892	Both	29
	HOH163	1.38	128	0.415	3.83	-248	-0.308	5.21	-120	-0.010	Protein A	12
	HOH190	0.00	-210	-0.186	0.00	-56	-0.039	0.00	-266	-0.327	Neither	24
	HOH195	0.00	-251	-0.288	2.36	224	0.606	2.36	-27	0.341	Protein B	5
	HOH197	0.00	-268	-0.332	2.03	22	0.341	2.03	-246	-0.304	Protein B	18
	HOH199	1.37	-11	0.265	4.11	422	0.893	5.48	411	0.977	Both	37
	HOH208	0.00	-116	-0.032	5.07	-4	0.642	5.07	-120	-0.010	Protein B	0
	HOH228	2.33	-57	0.300	1.37	113	0.400	3.71	55	0.599	Both	8
	HOH245	0.88	-13	0.219	1.19	-99	0.177	2.07	-112	-0.003	Neither	25
	HOH246	1.27	398	0.550	2.35	-155	-0.096	3.62	242	0.780	Protein A	1
	HOH248	1.06	48	0.295	1.11	309	0.420	2.17	357	0.675	Both	45
	HOH286	1.05	20	0.273	2.50	375	0.711	3.55	395	0.829	Both	0
3KDF	HOH93	1.34	-106	0.152	2.61	43	0.454	3.94	-63	0.519	Protein B	3
	HOH94	1.45	41	0.328	3.75	285	0.808	5.20	326	0.934	Both	18
	HOH100	2.10	1	0.328	1.25	182	0.408	3.34	183	0.714	Both	49
	HOH102	2.19	-210	-0.219	0.00	-44	-0.039	2.19	-253	-0.321	Neither	0
	HOH119	2.32	425	0.725	0.96	3	0.246	3.28	428	0.816	Protein A	19
	HOH143	2.78	17	0.447	2.27	-207	-0.213	5.05	-190	-0.172	Protein A	7
	HOH146	1.12	-38	0.237	1.14	-5	0.265	2.26	-44	0.303	Protein B	2
	HOH149	0.00	-66	-0.039	2.39	128	0.520	2.39	62	0.446	Protein B	0
	HOH154	0.99	-64	0.197	2.31	-44	0.313	3.30	-108	0.379	Protein B	5
	HOH166	1.09	124	0.350	1.11	-198	-0.193	2.20	-74	0.252	Protein A	3
	HOH168	0.00	-235	-0.246	1.95	-248	-0.308	1.95	-482	-0.918	Neither	5
	HOH176	0.86	-246	-0.303	1.07	108	0.337	1.93	-138	-0.059	Protein B	44
	HOH183	0.00	-173	-0.104	1.76	-80	0.185	1.76	-253	-0.321	Neither	35
	HOH202	0.00	-179	-0.115	1.75	48	0.356	1.75	-131	-0.043	Protein B	4
	HOH258	0.87	8	0.233	0.89	-258	-0.332	1.76	-250	-0.313	Neither	0
3KDJ	HOH6	2.62	-192	-0.177	1.26	31	0.306	3.87	-160	-0.105	Protein B	0
	HOH25	1.14	12	0.280	1.04	99	0.328	2.18	111	0.471	Both	15
	HOH53	2.18	51	0.402	1.17	23	0.292	3.35	74	0.569	Both	32
	HOH76	0.91	-96	0.158	0.00	-81	-0.040	0.91	-177	-0.144	Neither	25
	HOH88	0.88	-490	-0.940	0.96	20	0.259	1.84	-470	-0.883	Protein B	27
	HOH106	1.29	-125	-0.031	1.99	390	0.696	3.28	265	0.743	Protein B	24
	HOH131	0.95	-402	-0.696	2.54	-178	-0.146	3.49	-579	-1.203	Neither	2
	HOH159	1.06	336	0.420	1.09	-103	0.181	2.14	232	0.583	Protein A	12
	HOH161	0.00	-253	-0.293	0.82	35	0.245	0.82	-218	-0.238	Neither	4
3KF6	HOH5	1.28	104	0.379	2.14	-57	0.262	3.43	47	0.554	Both	26
	HOH28	1.06	52	0.298	0.00	-50	-0.039	1.06	2	0.261	Protein A	22
	HOH32	3.59	53	0.581	0.00	-252	-0.291	3.59	-199	-0.193	Protein A	28
	HOH51	0.95	-114	-0.006	0.00	-46	-0.039	0.95	-161	-0.108	Neither	29
	HOH62	2.14	161	0.526	1.11	6	0.272	3.25	166	0.679	Both	42
	HOH65	1.01	-123	-0.024	0.00	-116	-0.032	1.01	-239	-0.286	Neither	6
	HOH84	0.88	29	0.251	0.84	49	0.258	1.72	78	0.390	Both	46
	HOH86	1.23	-288	-0.404	2.05	-124	-0.029	3.29	-412	-0.723	Neither	1
	HOH89	0.82	130	0.303	0.84	-17	0.209	1.66	113	0.427	Protein A	0
	HOH120	0.94	21	0.256	1.15	-72	0.208	2.09	-50	0.259	Protein A	18

	HOH125	1.13	-310	-0.458	2.23	41	0.399	3.36	-269	-0.357	Protein B	2
	HOH131	1.90	56	0.374	0.00	-19	-0.039	1.90	37	0.350	Protein A	9
	HOH135	0.93	-75	0.179	1.15	-104	0.178	2.08	-179	-0.149	Neither	1
	HOH151	0.00	-30	-0.039	0.76	-32	0.183	0.76	-61	0.160	Neither	15
	HOH152	2.08	-48	0.261	1.02	7	0.259	3.10	-41	0.426	Both	1
	HOH159	0.99	-45	0.213	1.12	47	0.305	2.11	3	0.333	Protein B	28
	HOH173	3.85	138	0.712	1.41	50	0.336	5.26	188	0.904	Both	7
	HOH181	1.18	-224	-0.253	1.20	-412	-0.723	2.38	-636	-1.377	Neither	28
	HOH185	0.00	-148	-0.061	0.86	124	0.307	0.86	-24	0.206	Protein B	18
	HOH186	0.97	-38	0.215	1.04	-270	-0.362	2.01	-309	-0.456	Neither	60
	HOH187	1.27	-22	0.252	3.35	19	0.517	4.62	-3	0.620	Both	21
	HOH188	0.00	-634	-1.370	2.19	145	0.514	2.19	-489	-0.937	Protein B	55
3KLD	HOH9	1.13	10	0.277	1.99	40	0.357	3.12	49	0.520	Both	30
	HOH53	0.96	-42	0.210	0.00	-288	-0.385	0.96	-330	-0.511	Neither	1
	HOH110	2.14	375	0.686	1.10	-83	0.201	3.24	292	0.749	Protein A	18
	HOH113	1.52	487	0.689	4.26	-193	-0.179	5.78	294	0.951	Protein A	24
	HOH114	2.14	319	0.646	0.00	-251	-0.287	2.14	68	0.415	Protein A	18
	HOH161	2.44	119	0.514	1.25	79	0.351	3.69	198	0.780	Both	29
	HOH170	1.95	135	0.474	0.00	-64	-0.039	1.95	71	0.394	Protein A	31
	HOH180	1.13	-175	-0.140	1.13	86	0.335	2.25	-89	0.244	Protein B	3
	HOH192	2.45	468	0.756	1.11	-49	0.229	3.57	419	0.841	Protein A	11
	HOH213	3.60	241	0.776	0.00	4	-0.039	3.60	244	0.777	Protein A	18
	HOH253	2.30	104	0.479	1.10	320	0.422	3.40	424	0.827	Both	0
	HOH256	3.91	102	0.662	1.27	458	0.582	5.18	559	1.013	Both	2
	HOH264	0.00	-41	-0.039	5.25	-384	-0.649	5.25	-424	-0.757	Neither	0
	HOH273	1.41	9	0.289	4.16	155	0.766	5.57	164	0.872	Both	22
	HOH322	2.59	-363	-0.594	1.39	32	0.315	3.98	-331	-0.512	Protein B	25
	HOH337	0.00	-163	-0.084	1.02	-51	0.213	1.02	-214	-0.228	Neither	2
	HOH345	0.96	-29	0.220	2.24	24	0.381	3.19	-5	0.475	Protein B	31
	HOH346	1.42	-74	0.186	3.90	429	0.877	5.32	355	0.949	Protein B	10
	HOH368	0.84	122	0.303	1.16	-192	-0.178	2.00	-70	0.210	Protein A	0
	HOH388	0.00	-265	-0.324	2.11	-52	0.260	2.11	-317	-0.477	Protein B	17
	HOH397	3.92	-608	-1.291	1.45	-163	-0.115	5.37	-772	-1.822	Neither	30
3KMU	HOH106	1.95	-70	0.205	0.00	-37	-0.039	1.95	-107	0.150	Neither	27
	HOH154	1.45	83	0.376	2.43	338	0.683	3.88	421	0.873	Both	9
	HOH158	1.06	65	0.309	0.00	-110	-0.029	1.06	-45	0.225	Protein A	43
	HOH159	1.27	82	0.356	0.80	-75	0.156	2.07	7	0.330	Protein A	41
	HOH189	1.24	385	0.532	1.03	12	0.263	2.27	397	0.705	Both	33
	HOH201	0.00	-14	-0.039	0.00	-60	-0.039	0.00	-75	-0.040	Neither	52
	HOH208	1.02	120	0.335	0.00	40	-0.038	1.02	160	0.342	Protein A	47
	HOH230	2.06	-82	0.210	1.08	414	0.458	3.14	333	0.757	Protein B	26
	HOH251	1.15	-95	0.186	2.11	-31	0.290	3.26	-126	-0.028	Protein B	2
	HOH254	0.85	-20	0.209	1.14	99	0.348	1.99	79	0.408	Protein B	42
	HOH270	0.00	-107	-0.040	1.97	-51	0.233	1.97	-158	-0.103	Neither	23
	HOH287	1.20	49	0.318	3.61	55	0.584	4.81	104	0.723	Both	2
	HOH290	2.28	-69	0.276	2.62	-154	-0.092	4.90	-223	-0.248	Protein A	19
	HOH294	0.00	-147	-0.060	0.89	303	0.369	0.89	156	0.316	Protein B	25
	HOH295	0.00	-26	-0.039	1.96	290	0.615	1.96	264	0.592	Protein B	25
3KYJ	HOH25	2.58	379	0.722	1.27	-282	-0.391	3.85	97	0.655	Protein A	20
	HOH27	1.97	9	0.315	0.00	-286	-0.379	1.97	-277	-0.378	Protein A	13
	HOH50	1.00	-241	-0.291	0.00	-301	-0.419	1.00	-542	-1.090	Neither	42
	HOH55	1.19	413	0.518	2.21	-268	-0.356	3.40	145	0.663	Protein A	9
	HOH77	4.33	476	0.931	1.51	-13	0.265	5.84	463	1.009	Both	60
	HOH95	2.14	208	0.567	0.00	-163	-0.085	2.14	45	0.390	Protein A	12
	HOH118	0.70	-24	0.179	0.00	-99	-0.040	0.70	-123	-0.025	Neither	20
	HOH127	3.57	233	0.772	1.11	-178	-0.147	4.67	55	0.673	Protein A	0
	HOH135	1.31	47	0.326	2.03	14	0.332	3.34	60	0.556	Both	7
	HOH145	1.18	-14	0.260	0.91	-63	0.185	2.09	-76	0.223	Protein A	14
	HOH149	0.00	-182	-0.120	0.00	-148	-0.061	0.00	-329	-0.494	Neither	0
	HOH176	0.97	-16	0.232	1.03	-239	-0.287	2.00	-255	-0.326	Neither	6

	HOH184	0.86	-473	-0.891	0.89	-47	0.194	1.75	-519	-1.025	Neither	5
	HOH189	0.00	-186	-0.131	1.00	-326	-0.499	1.00	-512	-1.004	Neither	26
	HOH202	0.00	-724	-1.662	0.98	-82	0.182	0.98	-806	-1.940	Neither	0
	HOH229	0.00	-25	-0.039	0.86	-61	0.178	0.86	-85	0.158	Neither	36
3L9J	HOH4	1.07	29	0.284	1.03	253	0.370	2.11	282	0.617	Both	4
	HOH13	1.35	62	0.345	2.59	175	0.603	3.94	237	0.818	Both	0
	HOH14	2.59	15	0.422	1.05	96	0.328	3.64	111	0.642	Both	0
	HOH17	1.03	76	0.309	0.00	-147	-0.060	1.03	-71	0.198	Protein A	8
	HOH76	1.21	166	0.396	2.22	59	0.417	3.43	225	0.752	Both	32
	HOH124	0.00	-90	-0.040	1.93	187	0.526	1.93	97	0.426	Protein B	33
	HOH137	1.40	193	0.449	3.68	38	0.579	5.07	231	0.913	Both	43
	HOH142	1.13	163	0.368	1.13	56	0.314	2.26	219	0.589	Both	22
	HOH159	0.92	-165	-0.118	1.05	68	0.307	1.97	-98	0.164	Protein B	0
	HOH176	0.99	-10	0.241	2.52	-16	0.378	3.51	-26	0.495	Protein B	4
	HOH195	4.19	58	0.645	1.51	135	0.436	5.70	193	0.934	Both	21
	HOH219	1.08	32	0.287	3.90	51	0.618	4.98	82	0.712	Both	4
	HOH236	1.06	-132	-0.044	1.22	220	0.408	2.28	88	0.458	Protein B	44
	HOH239	1.04	24	0.275	2.42	141	0.540	3.47	165	0.704	Both	38
	HOH240	0.85	-3	0.220	1.02	292	0.391	1.87	289	0.602	Protein B	33
	HOH244	0.95	23	0.258	1.00	-95	0.174	1.95	-72	0.202	Protein A	24
3LIZ	HOH2	5.59	469	1.002	0.00	-94	-0.040	5.59	375	0.968	Protein A	47
	HOH4	1.33	375	0.565	2.12	10	0.344	3.45	386	0.815	Both	32
	HOH22	2.46	-33	0.350	1.28	-411	-0.722	3.74	-444	-0.812	Protein A	0
	HOH37	1.19	-233	-0.273	1.14	-439	-0.798	2.33	-672	-1.492	Neither	3
	HOH38	3.08	148	0.630	2.50	-222	-0.248	5.58	-74	0.597	Protein A	0
	HOH48	2.76	-139	-0.060	2.40	47	0.431	5.16	-92	0.563	Protein B	0
	HOH58	1.18	-257	-0.330	1.13	-56	0.222	2.31	-313	-0.467	Neither	8
	HOH75	0.97	-85	0.177	0.00	-168	-0.094	0.97	-253	-0.321	Neither	27
	HOH78	1.44	295	0.541	3.99	186	0.802	5.43	481	1.000	Both	2
	HOH80	1.14	184	0.370	3.80	-36	0.531	4.94	148	0.808	Both	15
	HOH96	0.89	-50	0.192	0.00	-299	-0.414	0.89	-349	-0.559	Neither	0
	HOH109	1.01	92	0.319	0.96	356	0.411	1.98	448	0.736	Both	0
	HOH136	0.94	-263	-0.344	0.00	-197	-0.155	0.94	-460	-0.856	Neither	1
	HOH140	3.59	-83	0.450	1.18	66	0.329	4.77	-17	0.616	Both	31
	HOH148	4.43	593	0.974	1.03	-83	0.189	5.46	509	1.011	Protein A	12
	HOH162	1.37	-371	-0.616	2.32	-82	0.267	3.69	-453	-0.836	Protein B	0
	HOH202	1.08	-142	-0.065	0.87	-74	0.168	1.95	-216	-0.234	Neither	0
	HOH209	1.10	394	0.453	0.81	-34	0.191	1.91	360	0.665	Protein A	0
	HOH220	1.25	52	0.325	0.92	-201	-0.198	2.17	-149	-0.082	Protein A	20
	HOH238	2.42	-120	-0.018	1.06	-145	-0.073	3.48	-265	-0.349	Neither	0
	HOH247	1.18	-71	0.206	2.45	-12	0.373	3.62	-82	0.456	Protein B	0
	HOH253	3.40	-79	0.425	0.00	-259	-0.310	3.40	-338	-0.530	Protein A	22
	HOH272	3.54	445	0.849	0.00	-64	-0.039	3.54	381	0.822	Protein A	12
	HOH345	2.17	-281	-0.388	0.90	0	0.234	3.08	-281	-0.387	Neither	0
	HOH394	1.22	-5	0.269	1.14	-635	-1.373	2.36	-639	-1.387	Protein A	0
	HOH418	0.00	-135	-0.047	0.94	16	0.252	0.94	-119	-0.017	Protein B	26
	HOH434	1.20	-195	-0.184	2.29	329	0.665	3.50	135	0.659	Protein B	17
	HOH446	0.86	-123	-0.024	0.83	-205	-0.207	1.69	-327	-0.502	Neither	0
	HOH488	0.93	-179	-0.148	0.98	-283	-0.392	1.91	-461	-0.859	Neither	2
	HOH500	0.00	-119	-0.033	1.09	152	0.355	1.09	34	0.290	Protein B	4
	HOH549	0.00	-24	-0.039	3.34	466	0.838	3.34	442	0.828	Protein B	0
	HOH550	0.98	-83	0.180	2.68	477	0.780	3.66	394	0.840	Protein B	0
	HOH631	0.95	-124	-0.027	2.23	-163	-0.113	3.17	-287	-0.402	Neither	14
	HOH684	0.00	-159	-0.078	3.24	63	0.547	3.24	-96	0.384	Protein B	2
	HOH687	0.00	-98	-0.040	3.29	-78	0.411	3.29	-175	-0.140	Protein B	32
	HOH706	1.27	286	0.480	2.52	-288	-0.405	3.79	-1	0.560	Protein A	0
	HOH715	0.00	-149	-0.064	0.00	-320	-0.468	0.00	-469	-0.877	Neither	24
	HOH725	0.87	-310	-0.460	1.05	3	0.260	1.92	-307	-0.452	Protein B	4
	HOH727	2.08	63	0.400	1.23	-231	-0.268	3.31	-168	-0.124	Protein A	0
	HOH731	0.00	-63	-0.039	2.35	136	0.525	2.35	73	0.451	Protein B	12

	HOH787	0.00	-87	-0.040	3.75	32	0.584	3.75	-55	0.504	Protein B	48
	HOH820	0.00	-152	-0.068	1.08	-57	0.219	1.08	-209	-0.217	Neither	49
	HOH866	0.00	-319	-0.466	0.82	40	0.248	0.82	-278	-0.381	Neither	0
3LXR	HOH2	0.75	111	0.283	0.00	-62	-0.039	0.75	49	0.242	Protein A	18
	HOH5	4.35	-83	0.527	1.40	-105	0.147	5.75	-188	-0.168	Protein A	9
	HOH17	2.14	96	0.447	0.00	-114	-0.031	2.14	-19	0.310	Protein A	0
	HOH19	4.27	-3	0.598	1.16	-195	-0.185	5.43	-198	-0.191	Protein A	3
	HOH25	2.01	-82	0.195	0.00	-64	-0.039	2.01	-146	-0.076	Neither	2
	HOH28	0.74	24	0.222	0.00	-25	-0.039	0.74	-1	0.203	Neither	9
	HOH39	2.42	-84	0.283	2.65	236	0.651	5.07	152	0.823	Both	1
	HOH59	0.82	-74	0.160	0.00	-16	-0.039	0.82	-90	0.146	Neither	0
	HOH68	0.00	-147	-0.060	0.75	-90	0.135	0.75	-237	-0.282	Neither	57
	HOH70	5.37	119	0.773	0.00	26	-0.038	5.37	145	0.823	Protein A	23
	HOH88	1.98	36	0.351	0.00	-110	-0.029	1.98	-74	0.200	Protein A	41
	HOH96	1.03	145	0.343	1.02	-35	0.225	2.04	110	0.452	Protein A	4
	HOH114	1.10	-172	-0.134	1.08	-255	-0.324	2.17	-427	-0.764	Neither	23
	HOH128	1.19	2	0.274	2.27	-52	0.295	3.46	-50	0.463	Both	9
	HOH141	1.22	93	0.359	1.16	-117	-0.011	2.37	-24	0.347	Protein A	5
	HOH146	0.91	-12	0.225	0.93	-374	-0.623	1.84	-387	-0.656	Neither	13
	HOH169	1.13	-23	0.251	1.08	-172	-0.132	2.21	-194	-0.183	Protein A	10
	HOH173	0.00	-149	-0.063	1.80	55	0.367	1.80	-94	0.167	Protein B	0
	HOH233	0.95	-146	-0.074	0.87	164	0.311	1.82	19	0.322	Protein B	11
	HOH234	1.12	320	0.432	0.90	-88	0.163	2.02	233	0.570	Protein A	0
	HOH241	1.39	-24	0.249	2.30	65	0.435	3.69	41	0.583	Protein B	21
	HOH263	2.18	155	0.526	1.08	-31	0.240	3.26	125	0.616	Protein A	29
	HOH264	0.00	-314	-0.454	0.00	-79	-0.040	0.00	-393	-0.666	Neither	9
	HOH265	0.92	-94	0.160	1.16	96	0.350	2.08	2	0.325	Protein B	0
	HOH269	2.24	-71	0.266	1.36	498	0.643	3.60	427	0.848	Both	51
	HOH270	1.28	-118	-0.015	2.66	-310	-0.459	3.94	-428	-0.768	Neither	24
	HOH271	0.00	-65	-0.039	2.01	-268	-0.356	2.01	-333	-0.516	Neither	16
	HOH273	0.00	-224	-0.218	3.54	171	0.724	3.54	-52	0.474	Protein B	29
	HOH278	0.90	-236	-0.281	1.19	552	0.565	2.09	316	0.642	Protein B	6
	HOH282	0.84	-268	-0.356	1.12	28	0.290	1.96	-240	-0.291	Protein B	5
	HOH285	1.41	-197	-0.191	4.27	497	0.933	5.67	300	0.948	Protein B	40
	HOH289	0.00	-217	-0.202	1.06	111	0.339	1.06	-107	0.175	Protein B	3
	HOH294	0.00	-186	-0.129	0.89	-110	0.004	0.89	-295	-0.423	Neither	1
	HOH299	0.83	-4	0.218	0.99	286	0.381	1.83	282	0.591	Protein B	18
	HOH318	0.88	-33	0.204	1.17	396	0.495	2.05	363	0.676	Protein B	10
	HOH341	0.88	-31	0.205	1.08	339	0.426	1.96	308	0.629	Protein B	38
	HOH364	1.32	-17	0.257	2.56	-57	0.338	3.89	-74	0.504	Both	10
	HOH369	0.00	-149	-0.064	0.72	46	0.234	0.72	-103	0.117	Neither	1
	HOH388	0.87	-154	-0.093	1.14	48	0.308	2.01	-105	0.160	Protein B	22
	HOH428	0.00	-295	-0.402	0.75	-68	0.152	0.75	-363	-0.594	Neither	31
	HOH435	0.00	-279	-0.361	0.73	-102	0.121	0.73	-381	-0.642	Neither	17
	HOH442	0.00	-205	-0.172	0.00	-70	-0.040	0.00	-275	-0.350	Neither	32
	HOH446	0.86	-250	-0.312	0.95	-17	0.228	1.81	-266	-0.353	Neither	0
	HOH449	1.38	-299	-0.433	1.40	358	0.579	2.79	59	0.491	Protein B	14
	HOH450	0.00	-64	-0.039	3.46	172	0.714	3.46	108	0.616	Protein B	31
	HOH457	0.00	-26	-0.039	0.00	-291	-0.392	0.00	-316	-0.460	Neither	0
	HOH460	0.00	-30	-0.039	2.25	273	0.622	2.25	243	0.602	Protein B	0
	HOH474	0.00	-57	-0.039	0.94	90	0.305	0.94	33	0.264	Protein B	18
3M18	HOH8	3.69	-38	0.511	1.39	44	0.328	5.08	6	0.651	Both	7
	HOH13	4.24	-376	-0.628	1.25	-4	0.271	5.49	-379	-0.637	Protein B	26
	HOH15	1.27	278	0.473	1.97	-313	-0.468	3.23	-36	0.448	Protein A	33
	HOH21	3.64	-67	0.474	0.00	-25	-0.039	3.64	-92	0.449	Protein A	0
	HOH23	1.22	41	0.313	1.13	76	0.328	2.35	117	0.501	Both	26
	HOH32	1.39	34	0.317	2.53	-1	0.396	3.92	33	0.604	Both	2
	HOH37	2.69	511	0.794	1.25	-226	-0.256	3.93	285	0.827	Protein A	13
	HOH58	1.30	34	0.312	1.88	19	0.326	3.18	53	0.530	Both	29
	HOH63	2.54	110	0.515	1.31	-303	-0.441	3.85	-192	-0.178	Protein A	0

	HOH65	5.53	-24	0.643	0.00	-63	-0.039	5.53	-87	0.583	Protein A	17
	HOH68	3.88	45	0.611	1.34	-70	0.196	5.22	-25	0.630	Protein A	10
	HOH79	2.81	83	0.517	2.79	268	0.684	5.60	351	0.960	Both	13
	HOH80	1.23	-34	0.240	1.09	39	0.295	2.32	5	0.373	Protein B	1
	HOH81	1.28	269	0.472	2.27	-84	0.255	3.55	186	0.746	Both	10
	HOH85	2.41	-52	0.319	0.96	-18	0.229	3.37	-70	0.429	Protein A	15
	HOH91	2.31	-161	-0.110	1.23	135	0.392	3.54	-26	0.499	Protein B	21
	HOH92	1.80	-189	-0.171	0.00	-216	-0.200	1.80	-405	-0.704	Neither	25
	HOH94	0.90	-260	-0.336	0.94	71	0.291	1.84	-189	-0.171	Protein B	25
	HOH103	2.30	-110	0.003	1.18	-31	0.244	3.47	-141	-0.063	Neither	31
	HOH108	2.05	71	0.406	0.00	-160	-0.080	2.05	-89	0.196	Protein A	23
	HOH120	0.99	145	0.336	0.85	-26	0.204	1.84	119	0.448	Protein A	8
	HOH138	1.16	9	0.278	0.87	7	0.232	2.02	16	0.333	Protein A	0
	HOH164	3.93	588	0.932	0.00	-63	-0.039	3.93	526	0.914	Protein A	22
	HOH179	0.94	-14	0.229	2.26	156	0.536	3.20	142	0.635	Protein B	7
	HOH183	2.07	-570	-1.173	1.22	10	0.284	3.30	-559	-1.143	Protein B	9
	HOH187	0.96	40	0.271	2.04	147	0.499	3.00	187	0.672	Both	9
	HOH197	1.16	82	0.339	2.46	252	0.634	3.61	334	0.810	Both	1
	HOH203	1.03	-188	-0.168	2.31	-122	-0.022	3.33	-309	-0.458	Neither	9
	HOH214	1.30	-66	0.202	2.37	48	0.427	3.67	-18	0.527	Protein B	26
	HOH216	1.13	162	0.370	1.21	-137	-0.055	2.34	25	0.398	Protein A	1
	HOH223	0.92	27	0.256	1.15	23	0.290	2.08	50	0.384	Both	19
	HOH234	1.20	33	0.303	2.39	266	0.634	3.60	299	0.793	Both	9
	HOH247	0.86	5	0.229	1.12	135	0.361	1.98	139	0.482	Protein B	25
	HOH258	1.02	-185	-0.162	1.22	211	0.402	2.24	26	0.383	Protein B	6
3M7F	HOH4	2.49	114	0.514	1.25	-155	-0.095	3.74	-40	0.516	Protein A	5
	HOH5	2.45	-131	-0.041	1.21	109	0.371	3.66	-22	0.522	Protein B	24
	HOH6	3.36	-70	0.429	0.00	-415	-0.726	3.36	-485	-0.926	Protein A	2
	HOH12	0.98	-126	-0.031	0.00	-202	-0.165	0.98	-327	-0.502	Neither	55
	HOH24	2.41	193	0.596	1.26	-92	0.175	3.67	101	0.634	Protein A	15
	HOH46	1.31	184	0.425	0.93	129	0.322	2.24	313	0.649	Both	5
	HOH49	2.19	-398	-0.685	0.00	-242	-0.264	2.19	-639	-1.388	Neither	0
	HOH62	0.92	-91	0.164	0.00	-219	-0.207	0.92	-310	-0.458	Neither	46
	HOH75	1.30	30	0.307	2.58	-65	0.331	3.88	-36	0.540	Both	11
	HOH89	0.00	-16	-0.039	1.01	-168	-0.125	1.01	-184	-0.161	Neither	31
	HOH92	0.97	-155	-0.096	1.13	-44	0.233	2.10	-199	-0.195	Neither	0
3MA2	HOH21	2.56	459	0.760	2.54	-131	-0.041	5.10	329	0.929	Protein A	13
	HOH33	2.39	533	0.780	0.96	-302	-0.438	3.34	231	0.742	Protein A	2
	HOH62	0.97	78	0.302	0.92	10	0.244	1.89	87	0.412	Protein A	23
	HOH65	4.07	43	0.624	1.45	-291	-0.413	5.51	-248	-0.308	Protein A	21
	HOH68	2.31	-622	-1.333	1.31	-421	-0.748	3.62	-1043	-2.821	Neither	17
	HOH86	2.38	-23	0.348	2.58	-307	-0.453	4.96	-331	-0.511	Protein A	8
	HOH93	1.18	367	0.488	1.02	-75	0.193	2.19	292	0.631	Protein A	17
	HOH94	0.76	-8	0.203	0.00	-248	-0.279	0.76	-255	-0.326	Neither	0
	HOH102	2.23	195	0.569	2.40	-18	0.358	4.63	176	0.842	Both	3
	HOH114	1.25	42	0.316	4.18	163	0.782	5.43	205	0.938	Both	7
	HOH115	0.00	-89	-0.040	0.87	-94	0.152	0.87	-183	-0.158	Neither	12
	HOH117	0.00	-75	-0.040	0.76	-275	-0.373	0.76	-350	-0.562	Neither	18
3MA9	HOH9	2.03	-275	-0.373	0.99	21	0.265	3.02	-254	-0.321	Protein B	24
	HOH19	3.97	363	0.858	1.11	-98	0.188	5.08	266	0.913	Protein A	21
	HOH32	2.62	82	0.494	1.27	-150	-0.085	3.89	-68	0.510	Protein A	15
	HOH42	1.08	239	0.371	0.94	-143	-0.068	2.02	96	0.432	Protein A	12
	HOH55	2.36	-121	-0.020	1.21	-54	0.221	3.57	-175	-0.138	Neither	0
	HOH74	1.09	-214	-0.229	0.00	-222	-0.214	1.09	-436	-0.789	Neither	29
	HOH106	1.05	379	0.438	1.02	64	0.300	2.07	443	0.729	Both	15
	HOH126	1.13	28	0.291	1.22	61	0.330	2.34	88	0.467	Both	4
	HOH144	1.33	90	0.371	3.61	-12	0.524	4.94	78	0.706	Both	19
	HOH163	2.22	-295	-0.421	1.03	-5	0.251	3.26	-300	-0.433	Protein B	14
	HOH197	1.12	-198	-0.192	1.15	91	0.343	2.27	-107	0.224	Protein B	31
	HOH214	1.00	-22	0.232	2.18	-70	0.254	3.18	-92	0.380	Protein B	45

	HOH217	2.38	-87	0.272	1.32	27	0.305	3.70	-60	0.490	Both	12
	HOH242	2.05	-127	-0.035	1.49	79	0.374	3.54	-48	0.477	Protein B	1
	HOH258	0.00	-376	-0.619	2.05	-160	-0.107	2.05	-536	-1.073	Neither	1
	HOH259	0.00	-17	-0.039	2.42	-35	0.341	2.42	-53	0.321	Protein B	7
	HOH269	0.91	46	0.268	0.94	-30	0.215	1.85	15	0.319	Protein A	56
	HOH276	0.00	-243	-0.267	2.27	511	0.765	2.27	269	0.622	Protein B	49
	HOH334	0.95	-279	-0.383	2.33	66	0.441	3.28	-213	-0.227	Protein B	3
	HOH339	1.20	-204	-0.207	1.25	-98	0.171	2.44	-303	-0.441	Neither	22
	HOH372	1.07	-97	0.183	1.10	361	0.441	2.17	264	0.608	Protein B	0
	HOH389	0.00	-194	-0.148	0.00	-93	-0.040	0.00	-288	-0.384	Neither	22
	HOH411	2.12	-277	-0.378	1.32	284	0.499	3.45	8	0.519	Protein B	0
3MC0	HOH4	2.33	-258	-0.332	1.08	-6	0.259	3.41	-263	-0.344	Protein B	0
	HOH6	1.35	540	0.654	3.57	-130	-0.036	4.91	410	0.947	Protein A	19
	HOH8	2.98	420	0.783	3.00	82	0.538	5.98	503	1.027	Both	0
	HOH9	0.81	33	0.241	0.00	-379	-0.626	0.81	-346	-0.550	Neither	11
	HOH12	1.45	512	0.681	4.28	-250	-0.312	5.72	262	0.945	Protein A	0
	HOH14	1.30	67	0.345	2.15	-189	-0.173	3.45	-123	-0.020	Protein A	10
	HOH15	1.28	56	0.333	2.29	-50	0.301	3.57	6	0.535	Both	1
	HOH21	1.28	-94	0.171	2.40	135	0.530	3.69	40	0.583	Protein B	15
	HOH24	1.24	-76	0.195	2.22	-119	-0.018	3.47	-196	-0.186	Neither	0
	HOH29	2.06	-141	-0.066	1.05	-51	0.219	3.11	-192	-0.178	Neither	11
	HOH35	1.35	-187	-0.167	3.67	-251	-0.314	5.02	-437	-0.793	Neither	18
	HOH43	0.81	7	0.222	0.00	-211	-0.188	0.81	-205	-0.208	Neither	0
	HOH47	2.09	38	0.372	0.00	-44	-0.039	2.09	-5	0.317	Protein A	0
	HOH59	1.90	-1	0.300	0.00	-96	-0.040	1.90	-96	0.167	Protein A	35
	HOH68	1.23	227	0.418	1.02	153	0.343	2.25	380	0.694	Both	20
	HOH83	1.11	61	0.314	1.01	-6	0.247	2.12	55	0.398	Protein A	7
	HOH96	4.12	11	0.600	1.50	-213	-0.226	5.61	-202	-0.200	Protein A	0
	HOH129	1.07	23	0.279	2.17	-116	-0.011	3.24	-93	0.387	Protein A	6
	HOH131	0.93	12	0.247	0.95	-122	-0.022	1.88	-110	0.001	Neither	3
	HOH136	2.10	-106	0.186	1.26	159	0.409	3.37	54	0.552	Protein B	30
	HOH143	0.00	-67	-0.039	0.79	-259	-0.335	0.79	-327	-0.501	Neither	0
	HOH161	1.09	16	0.277	2.43	-51	0.325	3.52	-36	0.487	Both	0
3MDY	HOH3	2.27	-321	-0.486	1.26	-138	-0.059	3.54	-459	-0.852	Neither	0
	HOH43	1.16	100	0.353	0.85	-12	0.215	2.01	88	0.422	Protein A	11
	HOH80	1.33	624	0.674	1.14	-58	0.220	2.46	566	0.797	Protein A	6
	HOH98	1.09	15	0.276	1.07	79	0.319	2.16	94	0.448	Both	4
	HOH300	1.03	64	0.303	0.89	-127	-0.034	1.92	-63	0.215	Protein A	27
	HOH567	2.30	-195	-0.186	2.44	61	0.451	4.74	-134	-0.043	Protein B	11
	HOH569	0.00	-328	-0.491	0.98	-121	-0.021	0.98	-449	-0.825	Neither	17
	HOH572	1.14	-250	-0.314	2.14	100	0.452	3.28	-150	-0.083	Protein B	2
	HOH573	1.21	-138	-0.057	2.30	101	0.475	3.52	-36	0.486	Protein B	1
	HOH574	1.26	-24	0.250	2.37	-54	0.311	3.63	-78	0.461	Both	16
	HOH575	1.23	-350	-0.560	2.60	202	0.631	3.83	-148	-0.077	Protein B	20
	HOH579	0.00	-170	-0.097	2.32	238	0.608	2.32	68	0.442	Protein B	25
	HOH586	2.45	239	0.626	2.64	198	0.633	5.09	437	0.967	Both	51
	HOH587	1.16	315	0.450	1.17	140	0.376	2.33	455	0.741	Both	9
	HOH593	1.01	25	0.270	1.07	23	0.279	2.08	48	0.383	Both	16
	HOH597	1.11	-3	0.265	2.21	116	0.481	3.31	113	0.605	Both	3
	HOH617	0.00	-155	-0.072	0.83	-19	0.206	0.83	-174	-0.139	Neither	0
	HOH618	1.09	-127	-0.034	2.35	39	0.415	3.44	-88	0.420	Protein B	50
	HOH620	0.00	-309	-0.439	2.28	-192	-0.178	2.28	-500	-0.969	Neither	14
	HOH624	0.86	56	0.267	0.86	80	0.284	1.73	135	0.457	Both	32
	HOH628	1.13	-93	0.190	2.25	29	0.389	3.38	-63	0.438	Protein B	11
3N3A	HOH163	1.04	-239	-0.288	0.00	-179	-0.114	1.04	-418	-0.739	Neither	13
	HOH164	1.06	-28	0.238	0.00	-59	-0.039	1.06	-87	0.191	Neither	26
	HOH178	2.39	439	0.737	1.36	172	0.435	3.75	611	0.921	Both	17
	HOH183	2.44	87	0.478	1.13	-204	-0.206	3.58	-117	-0.007	Protein A	0
	HOH200	1.87	-243	-0.296	0.00	-126	-0.038	1.87	-368	-0.607	Neither	0
	HOH210	2.58	131	0.546	1.11	-5	0.264	3.69	126	0.669	Both	1

	HOH253	2.38	193	0.590	0.00	-96	-0.040	2.38	97	0.480	Protein A	25
	HOH255	2.57	-31	0.369	1.27	-135	-0.052	3.84	-166	-0.118	Protein A	2
	HOH256	5.06	62	0.699	0.00	34	-0.038	5.06	96	0.727	Protein A	5
	HOH262	0.87	-198	-0.192	1.12	214	0.366	1.99	17	0.327	Protein B	12
	HOH267	0.93	113	0.316	0.00	-91	-0.040	0.93	22	0.254	Protein A	21
	HOH270	4.02	-28	0.557	1.41	31	0.314	5.43	3	0.663	Both	0
	HOH272	1.05	-38	0.230	2.27	-1	0.355	3.32	-39	0.455	Protein B	1
	HOH274	0.87	-103	0.144	0.89	-174	-0.139	1.76	-278	-0.380	Neither	17
	HOH277	0.00	3	-0.039	3.63	-104	0.435	3.63	-101	0.438	Protein B	10
3NCE	HOH15	4.85	170	0.845	0.00	89	-0.038	4.85	258	0.897	Protein A	4
	HOH17	2.35	207	0.597	0.96	-295	-0.422	3.31	-89	0.402	Protein A	52
	HOH39	2.18	36	0.386	1.10	-45	0.232	3.28	-9	0.482	Protein A	41
	HOH52	2.23	718	0.836	0.00	-101	-0.040	2.23	617	0.807	Protein A	34
	HOH86	5.67	378	0.973	0.00	-12	-0.039	5.67	366	0.969	Protein A	24
	HOH87	0.97	-197	-0.189	0.00	-268	-0.333	0.97	-465	-0.869	Neither	0
	HOH98	2.11	156	0.517	0.00	-58	-0.039	2.11	97	0.445	Protein A	25
	HOH102	4.20	-5	0.591	1.33	246	0.468	5.53	240	0.937	Both	10
	HOH105	1.27	129	0.397	1.10	20	0.281	2.37	148	0.543	Both	1
	HOH112	0.93	115	0.317	0.79	-47	0.176	1.72	68	0.377	Protein A	0
	HOH122	0.75	23	0.224	0.00	-176	-0.109	0.75	-153	-0.090	Neither	35
	HOH142	1.01	125	0.335	0.00	-210	-0.185	1.01	-85	0.183	Protein A	1
	HOH221	0.00	-640	-1.388	1.91	218	0.546	1.91	-422	-0.750	Protein B	18
	HOH223	1.31	92	0.372	2.69	196	0.638	4.01	287	0.835	Both	21
	HOH236	0.00	-122	-0.036	5.10	330	0.929	5.10	208	0.922	Protein B	1
	HOH265	0.00	-305	-0.429	0.79	-138	-0.058	0.79	-443	-0.807	Neither	13
	HOH270	1.11	331	0.431	1.19	122	0.374	2.30	453	0.738	Both	21
	HOH271	2.49	400	0.724	1.28	129	0.398	3.77	529	0.900	Both	0
	HOH296	0.00	-313	-0.449	1.02	-38	0.224	1.02	-350	-0.561	Neither	36
	HOH298	0.00	-16	-0.039	2.29	-516	-1.015	2.29	-532	-1.062	Neither	0
	HOH304	0.00	-105	-0.040	1.01	-139	-0.059	1.01	-244	-0.298	Neither	0
	HOH307	0.83	-324	-0.493	1.38	-435	-0.787	2.21	-759	-1.778	Neither	3
	HOH328	0.98	-28	0.225	2.55	544	0.795	3.53	517	0.875	Protein B	24
	HOH329	0.91	8	0.240	1.12	93	0.338	2.03	101	0.439	Protein B	3
	HOH336	0.00	-64	-0.039	1.93	-122	-0.024	1.93	-186	-0.165	Neither	26
	HOH342	3.64	-24	0.517	1.42	362	0.586	5.06	338	0.930	Both	26
	HOH346	1.18	-114	-0.006	3.77	-187	-0.167	4.94	-302	-0.438	Neither	0
	HOH349	0.00	133	-0.038	2.09	43	0.378	2.09	176	0.535	Protein B	0
	HOH350	2.52	-278	-0.380	1.54	-36	0.236	4.06	-314	-0.469	Neither	19
	HOH356	0.00	-16	-0.039	3.55	310	0.793	3.55	294	0.787	Protein B	17
	HOH361	0.95	-9	0.234	1.03	-85	0.187	1.98	-94	0.169	Neither	3
	HOH382	2.16	-447	-0.819	1.13	70	0.324	3.29	-377	-0.631	Protein B	18
	HOH427	0.00	-43	-0.039	2.08	217	0.565	2.08	174	0.532	Protein B	2
3OG6	HOH4	3.86	210	0.813	1.35	-11	0.265	5.21	199	0.919	Both	44
	HOH7	4.04	114	0.688	1.38	109	0.397	5.42	223	0.934	Both	18
	HOH14	1.98	-41	0.247	0.00	-168	-0.095	1.98	-209	-0.218	Neither	32
	HOH18	1.16	10	0.280	0.76	-23	0.191	1.92	-12	0.286	Protein A	12
	HOH68	0.00	2	-0.039	0.00	-11	-0.039	0.00	-8	-0.039	Neither	3
	HOH70	2.30	-100	0.240	1.08	69	0.315	3.38	-31	0.472	Protein B	0
	HOH104	0.00	-124	-0.037	0.00	-128	-0.040	0.00	-252	-0.290	Neither	0
	HOH120	0.00	5	-0.039	2.11	171	0.533	2.11	176	0.537	Protein B	6
	HOH133	0.85	30	0.246	1.17	24	0.292	2.02	54	0.380	Protein B	20
	HOH140	1.12	227	0.376	2.50	-77	0.305	3.62	150	0.700	Both	53
	HOH150	2.51	273	0.652	2.90	8	0.453	5.41	282	0.933	Both	34
	HOH154	1.29	95	0.372	2.64	431	0.754	3.93	526	0.914	Both	15
	HOH162	1.14	-402	-0.696	1.19	0	0.272	2.33	-401	-0.695	Protein B	23
	HOH163	0.76	-73	0.151	1.02	14	0.264	1.78	-59	0.217	Protein B	36
	HOH171	0.00	-83	-0.040	1.07	276	0.391	1.07	193	0.349	Protein B	15
	HOH175	0.00	-215	-0.197	0.96	-100	0.163	0.96	-315	-0.471	Neither	17
	HOH177	1.05	-19	0.243	1.27	378	0.541	2.32	359	0.686	Protein B	32
	HOH195	0.00	-172	-0.101	0.00	-223	-0.217	0.00	-395	-0.672	Neither	45

	HOH212	0.00	-168	-0.094	2.12	230	0.578	2.12	62	0.406	Protein B	41
	HOH217	0.88	53	0.268	1.10	91	0.332	1.98	144	0.488	Both	2
	HOH218	1.11	73	0.322	2.17	313	0.644	3.28	386	0.797	Both	47
	HOH222	0.00	-109	-0.040	0.00	-177	-0.111	0.00	-285	-0.377	Neither	0
	HOH266	0.83	-262	-0.342	1.05	-48	0.221	1.88	-310	-0.459	Neither	17
3OKY	HOH11	1.17	133	0.374	1.05	-343	-0.543	2.22	-211	-0.221	Protein A	41
	HOH18	1.74	-119	-0.018	0.00	-387	-0.649	1.74	-506	-0.985	Neither	0
	HOH20	1.90	-48	0.236	0.00	-65	-0.039	1.90	-113	-0.005	Neither	4
	HOH32	5.31	-62	0.598	0.00	-386	-0.646	5.31	-448	-0.823	Protein A	28
	HOH33	1.27	-257	-0.329	2.01	-232	-0.271	3.27	-489	-0.936	Neither	17
	HOH42	1.09	64	0.312	0.94	55	0.279	2.03	119	0.461	Both	14
	HOH54	2.13	134	0.493	1.16	26	0.294	3.29	160	0.673	Both	32
	HOH62	3.71	796	0.952	0.00	-369	-0.599	3.71	428	0.859	Protein A	22
	HOH109	0.94	-90	0.168	0.86	-3	0.222	1.79	-93	0.168	Neither	3
	HOH235	0.97	173	0.331	1.17	-53	0.223	2.15	120	0.478	Protein A	2
	HOH243	1.21	-16	0.258	2.50	222	0.624	3.71	206	0.791	Both	14
	HOH248	0.00	-70	-0.039	1.11	322	0.425	1.11	252	0.386	Protein B	24
	HOH253	0.00	-265	-0.324	0.74	-205	-0.207	0.74	-470	-0.882	Neither	0
	HOH264	1.28	73	0.350	2.38	-95	0.263	3.66	-21	0.523	Both	9
	HOH271	1.22	-9	0.265	2.35	129	0.515	3.57	120	0.645	Both	1
	HOH282	1.03	30	0.278	3.77	6	0.564	4.80	36	0.664	Both	29
	HOH300	0.98	184	0.331	1.15	-172	-0.133	2.13	12	0.347	Protein A	18
	HOH303	2.20	-25	0.315	1.33	106	0.387	3.52	81	0.597	Both	13
	HOH317	0.93	-37	0.209	1.17	-145	-0.074	2.10	-182	-0.156	Neither	7
	HOH324	0.94	-22	0.222	0.87	-59	0.181	1.81	-81	0.187	Neither	9
	HOH339	0.96	-3	0.241	2.32	-262	-0.341	3.28	-265	-0.349	Neither	17
	HOH347	1.02	27	0.273	1.14	212	0.373	2.16	239	0.589	Both	31
	HOH357	2.25	1	0.355	1.30	-13	0.262	3.55	-12	0.515	Both	27
	HOH364	1.07	60	0.306	1.21	-160	-0.107	2.28	-101	0.234	Protein A	39
	HOH376	0.73	-31	0.177	0.00	-40	-0.039	0.73	-71	0.145	Neither	20
	HOH382	1.09	4	0.268	2.56	-90	0.299	3.65	-86	0.457	Both	0
3ORV	HOH50	1.17	7	0.278	1.00	-267	-0.353	2.17	-259	-0.336	Protein A	0
	HOH72	1.10	-20	0.252	1.04	-41	0.225	2.14	-60	0.258	Protein A	0
	HOH77	0.71	-278	-0.379	0.00	-158	-0.076	0.71	-435	-0.787	Neither	3
	HOH84	1.39	-56	0.210	2.54	-283	-0.392	3.92	-339	-0.531	Neither	10
	HOH105	1.26	-127	-0.035	2.27	-319	-0.482	3.53	-446	-0.817	Neither	37
	HOH113	1.06	175	0.349	0.97	-24	0.226	2.03	151	0.501	Protein A	30
	HOH129	0.00	9	-0.039	0.74	-91	0.133	0.74	-82	0.140	Neither	26
	HOH146	0.00	-164	-0.087	0.00	-3	-0.039	0.00	-167	-0.092	Neither	27
	HOH186	2.09	61	0.400	0.00	-42	-0.039	2.09	18	0.349	Protein A	37
	HOH203	1.06	35	0.286	0.00	-140	-0.052	1.06	-105	0.176	Protein A	25
	HOH204	0.00	-78	-0.040	0.80	-90	0.143	0.80	-168	-0.124	Neither	31
	HOH206	2.30	120	0.498	0.96	-139	-0.060	3.26	-19	0.468	Protein A	1
	HOH217	0.93	153	0.324	0.00	-159	-0.078	0.93	-6	0.233	Protein A	0
	HOH526	1.49	156	0.451	4.26	262	0.852	5.75	417	0.990	Both	0
	HOH537	4.13	54	0.638	1.50	-246	-0.304	5.63	-192	-0.177	Protein A	0
	HOH538	1.10	-281	-0.387	2.26	3	0.359	3.35	-278	-0.380	Protein B	3
	HOH562	0.91	-64	0.183	2.25	309	0.648	3.16	245	0.721	Protein B	5
	HOH572	0.80	28	0.236	1.09	-258	-0.332	1.89	-230	-0.267	Neither	26
	HOH579	0.83	-92	0.146	1.06	-28	0.239	1.89	-119	-0.018	Neither	2
	HOH585	0.95	-103	0.158	2.52	4	0.401	3.47	-99	0.414	Protein B	1
	HOH589	1.04	-111	0.001	1.85	143	0.476	2.89	32	0.475	Protein B	1
	HOH590	1.09	100	0.336	2.60	-543	-1.094	3.68	-443	-0.810	Protein A	17
	HOH600	0.00	-313	-0.450	0.99	-90	0.176	0.99	-403	-0.700	Neither	5
	HOH705	0.00	-37	-0.039	1.83	-240	-0.290	1.83	-277	-0.378	Neither	1
	HOH712	1.01	24	0.269	2.33	133	0.517	3.34	157	0.674	Both	12
	HOH733	1.19	248	0.420	1.23	332	0.496	2.42	580	0.800	Both	18
	HOH739	2.11	-15	0.309	1.03	-76	0.195	3.14	-91	0.376	Protein A	2
	HOH827	1.15	-122	-0.023	1.26	-62	0.210	2.40	-184	-0.161	Neither	1
	HOH832	1.19	-19	0.255	2.32	-334	-0.520	3.51	-354	-0.570	Protein A	2

	HOH1024	1.26	131	0.396	4.22	163	0.785	5.48	293	0.939	Both	13
	HOH1236	1.09	-52	0.224	2.32	6	0.372	3.40	-47	0.459	Protein B	9
3Q3J	HOH10	5.643	222.8	0.618	0	-373.1	-0.273	5.643	-150.3	-0.07	Protein A	7
	HOH15	2.673	58.9	0.681	2.54	137.7	0.55	5.213	196.6	0.553	Both	15
	HOH29	0	-248.6	-0.146	3.639	-51.1	0.851	3.639	-299.8	0.131	Protein B	16
