# Supplementary material for: Bound Water at Protein-Protein Interfaces: Partners, Roles and Hydrophobic Bubbles as a Conserved Motif
Source: PLoS One. 2011 Sep 22;6(9):e24712. doi: 10.1371/journal.pone.0024712 (PMC3178540; doi:10.1371/journal.pone.0024712)
Supplement: Table S3 — Water Rank, HINT score, Relevance and solvent accessible surface area for low-resolution data set. (PDF) [file pone.0024712.s004.pdf]

Table S3: Water Rank, HINT score, Relevance and solvent accessible surface area for low-resolution data set.

PDB ID	Water Name	Rank A	Score A	Relev A	Rank B	Score B	Relev B	Total Rank	Total Score	Overall Relev	Water Relev to:	SASA (A^2)
1HE8	HOH1	1.09	139	0.355	0.00	-442	-0.802	1.09	-303	-0.442	Protein A	10
1MA9	HOH1	1.29	-89	0.176	1.98	165	0.511	3.27	76	0.562	Protein B	5
	HOH7	3.74	-253	-0.319	1.41	396	0.607	5.15	144	0.810	Protein B	2
	HOH14	2.67	-197	-0.191	2.70	-95	0.313	5.37	-292	-0.416	Protein B	0
	HOH15	2.17	-78	0.240	0.00	-115	-0.032	2.17	-194	-0.182	Neither	6
	HOH18	3.32	-35	0.460	1.19	-51	0.225	4.50	-85	0.536	Protein A	1
	HOH23	1.19	-114	-0.005	1.12	-121	-0.020	2.30	-235	-0.278	Neither	0
	HOH50	1.19	247	0.419	1.91	-88	0.179	3.10	159	0.649	Protein A	10
	HOH57	0.00	-172	-0.101	0.73	-12	0.194	0.73	-183	-0.159	Neither	30
	HOH59	0.93	114	0.318	0.79	-9	0.206	1.72	105	0.422	Protein A	50
	HOH61	2.31	-113	-0.003	0.00	189	-0.039	2.31	76	0.450	Neither	8
	HOH82	1.06	-119	-0.016	0.81	-94	0.141	1.87	-214	-0.228	Neither	16
	HOH89	3.73	50	0.597	0.00	61	-0.038	3.73	110	0.653	Protein A	16
	HOH91	2.13	-108	0.190	1.07	-48	0.223	3.20	-157	-0.098	Neither	14
	HOH106	1.23	-322	-0.490	1.02	76	0.309	2.25	-246	-0.304	Protein B	18
	HOH108	1.21	-160	-0.107	1.09	301	0.410	2.30	141	0.524	Protein B	8
	HOH131	1.15	36	0.301	0.89	193	0.308	2.04	229	0.569	Both	28
	HOH144	2.57	-241	-0.291	2.77	589	0.830	5.34	349	0.948	Protein B	8
	HOH145	1.22	-106	0.166	2.70	716	0.857	3.91	610	0.936	Protein B	2
	HOH152	1.21	-226	-0.256	3.71	268	0.798	4.92	42	0.676	Protein B	0
	HOH154	1.31	-155	-0.096	2.35	116	0.499	3.67	-39	0.506	Protein B	0
	HOH157	0.00	-156	-0.073	3.43	-179	-0.148	3.43	-335	-0.521	Neither	12
	HOH165	0.93	2	0.239	1.26	-383	-0.645	2.19	-380	-0.639	Neither	1
	HOH168	2.23	133	0.505	1.31	117	0.394	3.53	250	0.770	Both	12
	HOH175	0.00	-231	-0.237	2.02	-90	0.186	2.02	-321	-0.487	Neither	4
	HOH176	0.00	-210	-0.184	3.21	-256	-0.328	3.21	-466	-0.871	Neither	0
	HOH180	1.11	-115	-0.007	1.14	-188	-0.168	2.25	-303	-0.441	Neither	12
	HOH181	2.09	-212	-0.226	1.36	515	0.649	3.44	302	0.777	Protein B	0
	HOH183	1.18	67	0.330	3.37	-102	0.396	4.55	-35	0.586	Both	3
	HOH189	0.00	-206	-0.174	2.17	-337	-0.527	2.17	-542	-1.091	Neither	13
	HOH191	0.00	-174	-0.104	2.27	174	0.557	2.27	0	0.358	Protein B	10
	HOH213	2.47	-57	0.324	2.57	-81	0.311	5.04	-138	-0.053	Both	3
	HOH217	1.20	-319	-0.483	2.55	214	0.628	3.75	-105	0.454	Protein B	3
	HOH225	2.16	-13	0.322	1.17	-5	0.267	3.33	-18	0.479	Both	24
	HOH242	0.88	-217	-0.237	1.20	331	0.477	2.07	114	0.461	Protein B	0
	HOH245	0.00	-170	-0.097	1.10	229	0.370	1.10	59	0.311	Protein B	37
1RE0	HOH3	1.41	21	0.303	2.51	433	0.744	3.92	455	0.889	Both	22
	HOH5	1.26	-24	0.250	2.26	-529	-1.054	3.52	-553	-1.124	Protein A	3
	HOH7	2.94	-58	0.388	2.70	-140	-0.061	5.64	-197	-0.189	Protein A	1
	HOH8	1.13	-84	0.198	1.12	-23	0.250	2.26	-107	0.221	Protein B	5
	HOH9	3.99	-253	-0.320	1.38	47	0.331	5.37	-206	-0.209	Protein B	12
	HOH11	2.21	-68	0.263	1.17	98	0.354	3.38	30	0.532	Both	7
	HOH15	3.50	-21	0.499	1.09	-27	0.244	4.59	-48	0.577	Protein A	8
	HOH23	3.20	201	0.714	0.00	-111	-0.029	3.20	91	0.567	Protein A	10
	HOH30	1.89	-229	-0.265	0.00	-153	-0.069	1.89	-382	-0.645	Neither	4
	HOH34	1.03	-12	0.246	0.00	-205	-0.172	1.03	-217	-0.235	Neither	38
	HOH44	2.04	-148	-0.082	1.18	33	0.301	3.22	-116	-0.006	Protein B	31
	HOH51	1.44	-341	-0.538	4.12	212	0.838	5.56	-129	-0.032	Protein B	0
	HOH56	1.20	-141	-0.065	2.29	-147	-0.077	3.49	-288	-0.404	Neither	2
	HOH64	0.00	-100	-0.040	2.06	54	0.387	2.06	-46	0.259	Protein B	13
	HOH66	0.88	37	0.257	1.01	37	0.278	1.89	74	0.395	Both	26
	HOH93	0.00	-38	-0.039	1.99	160	0.507	1.99	122	0.461	Protein B	22
	HOH98	0.00	-60	-0.039	3.67	435	0.858	3.67	375	0.833	Protein B	3
	HOH107	0.00	-345	-0.537	5.38	295	0.935	5.38	-50	0.613	Protein B	2
2CH4	none										N/A	
2JGZ	HOH3	1.79	-66	0.206	0.00	-32	-0.039	1.79	-98	0.161	Neither	36
	HOH4	0.98	-27	0.226	2.31	97	0.472	3.29	70	0.559	Protein B	33
	HOH5	2.53	-501	-0.972	1.30	212	0.430	3.83	-290	-0.409	Protein B	2
3DI3	none										N/A	
3DPL	HOH16	3.95	294	0.832	1.36	71	0.356	5.31	366	0.953	Both	21

	HOH26	2.15	107	0.463	1.28	24	0.300	3.42	132	0.645	Both	10
	HOH36	2.63	505	0.786	0.96	-168	-0.125	3.59	337	0.808	Protein A	20
	HOH62	0.00	-180	-0.118	2.33	342	0.676	2.33	162	0.553	Protein B	22
	HOH63	1.16	480	0.527	1.10	-23	0.249	2.26	457	0.739	Protein A	15
	HOH66	0.00	-250	-0.286	5.18	-4	0.647	5.18	-255	-0.324	Protein B	0
	HOH71	1.03	-61	0.207	2.53	202	0.619	3.55	141	0.677	Protein B	14
	HOH72	2.34	411	0.718	0.00	-165	-0.088	2.34	247	0.616	Protein A	6
3GJ6	HOH20	2.47	125	0.525	1.02	-153	-0.090	3.48	-28	0.490	Protein A	4
	HOH34	2.54	-162	-0.110	2.57	-83	0.308	5.10	-245	-0.300	Protein B	2
3H5C	HOH16	0.00	-82	-0.040	0.81	196	0.290	0.81	115	0.296	Protein B	48
3LVL	none										N/A	
3NCC	HOH11	4.01	163	0.767	1.20	147	0.387	5.20	310	0.929	Both	0
	HOH13	2.59	88	0.497	1.10	-163	-0.112	3.69	-74	0.475	Protein A	23
	HOH24	2.57	89	0.495	2.56	-21	0.380	5.13	68	0.708	Both	0
	HOH34	1.20	-53	0.222	2.00	-116	-0.012	3.20	-169	-0.126	Neither	0
	HOH42	1.40	397	0.604	2.40	106	0.494	3.80	503	0.895	Both	12
	HOH43	1.18	-66	0.210	1.19	45	0.313	2.37	-21	0.351	Protein B	10
	HOH48	2.45	731	0.843	0.00	-157	-0.076	2.45	574	0.799	Protein A	8
	HOH61	0.84	-193	-0.181	0.96	-146	-0.075	1.79	-339	-0.532	Neither	11
	HOH68	0.95	150	0.329	0.00	-364	-0.586	0.95	-213	-0.227	Protein A	16
	HOH77	3.91	635	0.941	0.00	-97	-0.040	3.91	538	0.915	Protein A	31
	HOH95	2.41	-59	0.311	1.31	453	0.598	3.71	394	0.845	Both	12
	HOH102	1.17	188	0.382	1.16	35	0.300	2.33	222	0.600	Both	52
	HOH107	2.04	-2	0.312	1.25	-7	0.268	3.29	-9	0.482	Both	4
	HOH108	1.29	98	0.374	2.75	206	0.655	4.03	304	0.842	Both	1
	HOH121	1.29	-60	0.210	4.36	542	0.955	5.65	482	1.009	Protein B	0
	HOH130	1.24	445	0.558	1.19	-175	-0.139	2.43	270	0.641	Protein A	0
	HOH131	2.73	520	0.801	1.35	84	0.368	4.08	604	0.949	Both	18
	HOH148	1.31	-166	-0.121	2.47	-325	-0.497	3.78	-491	-0.943	Neither	1
	HOH154	1.17	-18	0.255	1.12	-369	-0.609	2.29	-387	-0.656	Protein A	9
	HOH165	4.35	450	0.923	1.26	1	0.276	5.61	451	0.997	Both	0
	HOH167	0.96	-69	0.189	2.51	515	0.780	3.47	446	0.843	Protein B	0
	HOH170	0.00	-82	-0.040	3.78	-136	-0.048	3.78	-217	-0.236	Neither	0
	HOH175	5.23	-71	0.587	0.00	466	-0.037	5.23	395	0.959	Protein A	0
	HOH191	2.15	342	0.664	1.15	-146	-0.076	3.30	196	0.724	Protein A	26
	HOH194	3.77	-538	-1.079	1.43	90	0.381	5.19	-448	-0.823	Protein B	0
	HOH207	1.11	68	0.317	1.18	64	0.327	2.28	132	0.510	Both	24
	HOH209	0.00	37	-0.038	2.15	-32	0.297	2.15	5	0.343	Protein B	0
	HOH226	0.00	-129	-0.041	5.48	328	0.948	5.48	198	0.933	Protein B	0
3NW0	HOH4	0.81	160	0.299	0.87	227	0.316	1.68	387	0.655	Both	62
	HOH10	1.06	-175	-0.139	0.81	-368	-0.606	1.87	-542	-1.092	Neither	3
	HOH16	1.08	356	0.434	1.05	-224	-0.252	2.13	132	0.490	Protein A	33
3P71	HOH6	1.22	407	0.531	3.46	201	0.751	4.68	608	0.996	Both	0
	HOH21	0.00	509	-0.037	4.95	42	0.677	4.95	552	0.998	Protein B	0
	HOH45	2.66	-279	-0.383	1.05	30	0.280	3.71	-250	-0.312	Protein B	0
	HOH54	1.27	232	0.436	2.37	275	0.637	3.63	507	0.881	Both	0
	HOH69	3.56	305	0.792	1.14	-209	-0.217	4.70	96	0.709	Protein A	0
	HOH110	0.00	-102	-0.040	3.87	813	0.965	3.87	712	0.952	Protein B	0
	HOH116	1.08	132	0.349	2.28	140	0.521	3.36	272	0.755	Both	29
3PCR	HOH4	2.39	-222	-0.247	1.32	-210	-0.221	3.70	-432	-0.778	Neither	0
	HOH6	2.17	-181	-0.155	1.24	-63	0.210	3.41	-244	-0.298	Neither	2
	HOH134	1.01	-323	-0.491	1.19	125	0.376	2.20	-198	-0.192	Protein B	32
3Q35	none										N/A	
