# Supplementary material for: Bound Water at Protein-Protein Interfaces: Partners, Roles and Hydrophobic Bubbles as a Conserved Motif
Source: PLoS One. 2011 Sep 22;6(9):e24712. doi: 10.1371/journal.pone.0024712 (PMC3178540; doi:10.1371/journal.pone.0024712)
Supplement: Table S4 — Water Rank, HINT score, Relevance and solvent accessible surface area for homo-dimer data set. (PDF) [file pone.0024712.s005.pdf]

Table S4: Water Rank, HINT score, Relevance and solvent accessible surface area for homo-dimer data set.

PDB ID	Water Name	Rank A	Score A	Relev A	Rank B	Score B	Relev B	Total Rank	Total Score	Overall Relev	Water Relev to:	SASA (A^2)
2X3J	HOH4	0.93	-231	-0.258	0.94	-98	0.161	1.87	-328	0.088	Neither	7
	HOH6	0.93	-106	0.153	0.00	-95	-0.040	0.93	-201	-0.191	Neither	11
	HOH15	0.74	138	0.450	0.00	-112	-0.027	0.74	26	0.224	Protein A	5
	HOH28	3.48	-65	0.825	1.47	122	0.414	4.95	57	0.690	Both	27
	HOH29	0.00	-97	-0.040	1.76	-40	0.476	1.76	-137	0.265	Protein B	17
	HOH50	3.31	182	0.657	0.00	-40	-0.039	3.31	143	0.623	Protein A	20
	HOH58	1.05	-130	-0.037	0.00	-275	-0.172	1.05	-405	-0.687	Neither	44
	HOH64	0.82	37	0.246	0.84	-163	-0.108	1.66	-126	0.259	Neither	3
	HOH65	0.88	-43	0.195	0.00	-255	-0.152	0.88	-298	-0.417	Neither	3
	HOH227	2.23	64	0.585	1.03	-330	-0.494	3.25	-266	0.154	Protein A	49
	HOH307	0.97	-115	-0.007	0.90	-237	-0.274	1.87	-353	0.060	Neither	18
	HOH308	0.00	-105	-0.040	1.03	-84	0.189	1.03	-189	-0.163	Neither	14
	HOH319	0.95	39	0.269	2.29	17	0.598	3.24	56	0.787	Both	23
	HOH324	0.88	27	0.249	0.93	-69	0.184	1.81	-42	0.488	Neither	13
	HOH325	1.22	168	0.398	2.21	-48	0.579	3.43	120	0.613	Both	8
	HOH328	0.90	25	0.252	1.22	109	0.363	2.12	134	0.504	Both	24
	HOH345	0.00	-179	-0.083	1.05	449	0.802	1.05	270	0.615	Protein B	10
	HOH346	0.00	-224	-0.123	0.94	-112	-0.001	0.94	-336	-0.510	Neither	4
	HOH350	0.98	-167	-0.117	2.00	81	0.534	2.98	-87	0.736	Protein B	10
	HOH488	1.00	-117	-0.010	2.38	37	0.620	3.39	-80	0.809	Protein B	21
	HOH566	2.12	8	0.559	1.10	-53	0.299	3.21	-45	0.780	Both	19
	HOH567	2.17	-16	0.569	0.00	-234	-0.132	2.17	-250	0.082	Protein A	26
2XFN	HOH127	2.12	233	0.571	1.02	40	0.283	3.13	272	0.752	Both	39
	HOH133	1.05	-30	0.234	0.98	43	0.279	2.03	13	0.538	Protein B	30
	HOH136	1.09	122	0.427	1.00	-15	0.238	2.08	107	0.476	Protein A	35
	HOH137	2.09	352	0.597	0.00	-45	-0.039	2.09	307	0.586	Protein A	8
	HOH165	2.54	129	0.542	0.97	-27	0.223	3.50	101	0.602	Protein A	28
	HOH166	1.15	-156	0.151	1.14	-348	-0.042	2.29	-504	-0.308	Neither	0
	HOH167	2.33	360	0.643	1.33	-384	-0.055	3.66	-25	0.855	Protein A	2
	HOH168	4.02	432	0.843	1.38	6	0.377	5.39	438	0.859	Both	0
	HOH198	1.24	-247	0.081	1.15	106	0.348	2.39	-141	0.246	Protein B	4
	HOH206	1.26	40	0.346	2.41	-261	0.090	3.67	-221	0.247	Protein A	0
	HOH207	2.08	45	0.551	0.00	-7	-0.038	2.08	38	0.550	Protein A	32
	HOH211	2.30	-9	0.599	1.25	-21	0.341	3.54	-30	0.837	Both	17
	HOH212	2.41	-291	0.047	1.13	-43	0.307	3.54	-334	0.072	Protein B	3
	HOH219	1.47	57	0.403	4.15	-300	-0.415	5.62	-243	-0.278	Protein A	1
	HOH220	0.98	-327	-0.487	0.00	-223	-0.122	0.98	-550	-1.090	Neither	0
	HOH221	1.40	3	0.383	3.97	392	0.804	5.37	395	0.817	Both	0
	HOH260	1.07	-35	0.235	1.06	148	0.468	2.13	114	0.487	Protein B	23
	HOH263	4.26	-37	0.566	1.28	84	0.353	5.54	47	0.706	Both	0
	HOH264	2.18	70	0.574	1.07	-122	-0.021	3.25	-53	0.786	Protein A	12
	HOH294	0.92	85	0.298	0.00	-174	-0.078	0.92	-88	0.165	Protein A	26
	HOH297	2.38	-226	0.136	1.01	-304	-0.430	3.39	-530	-0.271	Neither	2
	HOH298	1.93	46	0.517	0.00	-198	-0.100	1.93	-152	0.275	Protein A	20
	HOH299	1.97	135	0.499	0.00	-198	-0.099	1.97	-63	0.526	Protein A	12
	HOH302	2.97	-259	0.141	2.79	-197	0.212	5.76	-456	-0.818	Neither	0
	HOH305	1.06	-133	-0.043	0.00	-459	-0.371	1.06	-591	-1.212	Neither	0
	HOH539	2.65	493	0.724	1.33	-98	0.362	3.98	395	0.807	Both	0
	HOH541	1.14	135	0.364	1.17	355	0.372	2.31	491	0.664	Both	27
	HOH542	0.00	-60	-0.039	2.02	365	0.585	2.02	305	0.571	Protein B	7
	HOH544	1.30	-148	0.183	2.32	192	0.573	3.62	44	0.851	Protein B	0
	HOH548	0.90	100	0.390	1.17	172	0.392	2.07	272	0.574	Both	30
	HOH569	1.01	-28	0.231	2.38	71	0.621	3.40	43	0.815	Protein B	29
	HOH570	1.26	75	0.347	4.24	22	0.618	5.50	97	0.398	Both	0
	HOH571	1.33	-381	-0.051	2.34	391	0.651	3.67	10	0.858	Protein B	2
	HOH572	1.13	-334	-0.028	1.16	-148	0.160	2.29	-482	-0.270	Neither	0
	HOH608	1.17	101	0.349	1.20	-259	0.063	2.37	-158	0.224	Protein A	3
	HOH615	0.00	-10	-0.038	2.12	35	0.560	2.12	25	0.560	Protein B	30
	HOH619	1.11	-62	0.303	2.40	-275	0.070	3.52	-337	0.067	Protein A	0
	HOH620	1.13	-39	0.308	2.26	-4	0.591	3.39	-43	0.811	Both	21

	HOH621	2.32	68	0.607	2.52	-219	0.159	4.84	-150	-0.070	Protein A	0
	HOH626	0.87	-36	0.200	1.16	-293	0.021	2.04	-329	-0.045	Neither	0
	HOH627	2.55	-83	0.652	2.56	-91	0.653	5.12	-174	-0.121	Both	0
	HOH628	4.12	-296	-0.405	1.45	47	0.396	5.57	-249	-0.292	Protein B	1
	HOH629	0.00	-219	-0.119	0.97	-333	-0.502	0.97	-552	-1.097	Neither	0
	HOH667	1.18	161	0.387	2.19	-18	0.575	3.37	143	0.629	Both	20
	HOH669	1.10	-127	0.168	2.19	79	0.577	3.29	-48	0.793	Protein B	8
	HOH705	2.51	-218	0.159	2.36	79	0.615	4.87	-139	-0.047	Protein B	0
	HOH706	0.00	-872	-0.998	2.17	234	0.581	2.17	-638	-0.578	Protein B	26
	HOH707	0.00	-179	-0.083	0.90	140	0.454	0.90	-39	0.202	Protein B	26
	HOH710	0.00	-193	-0.095	2.00	133	0.490	2.00	-60	0.530	Protein B	10
	HOH711	0.00	-199	-0.101	1.89	40	0.507	1.89	-159	0.263	Protein B	21
	HOH714	2.80	-141	0.285	2.98	-312	0.065	5.78	-453	-0.809	Protein A	0
	HOH717	0.00	-463	-0.377	1.06	-119	-0.013	1.06	-582	-1.185	Neither	0
2XIQ	HOH301	1.16	351	0.369	1.16	-152	0.157	2.32	199	0.578	Protein A	33
	HOH303	2.25	142	0.524	1.02	-53	0.212	3.27	89	0.794	Protein A	15
	HOH373	1.06	-481	-0.894	0.97	37	0.272	2.03	-444	-0.229	Protein B	0
	HOH374	1.20	-79	0.328	2.35	-301	0.027	3.55	-380	0.000	Protein A	0
	HOH375	1.17	-325	-0.012	2.21	255	0.594	3.38	-70	0.808	Protein B	14
	HOH376	2.88	-10	0.719	2.60	-61	0.662	5.47	-70	0.597	Both	22
	HOH377	2.22	-93	0.578	0.98	-73	0.189	3.20	-166	0.285	Protein A	24
	HOH378	1.27	-174	0.154	2.12	26	0.560	3.39	-148	0.322	Protein B	7
	HOH379	1.96	154	0.509	0.00	-163	-0.069	1.96	-10	0.525	Protein A	23
	HOH380	1.23	167	0.399	1.09	-30	0.243	2.32	137	0.528	Protein A	9
	HOH382	2.42	371	0.661	1.31	264	0.393	3.73	635	0.880	Both	6
	HOH398	1.33	255	0.396	2.47	358	0.666	3.80	612	0.889	Both	3
	HOH403	0.79	110	0.406	0.00	-58	-0.039	0.79	52	0.252	Protein A	0
	HOH414	1.05	17	0.270	0.89	15	0.242	1.94	32	0.519	Protein A	0
	HOH415	1.10	228	0.336	2.07	-253	0.068	3.17	-24	0.773	Protein A	0
	HOH417	1.15	409	0.373	1.07	49	0.299	2.22	458	0.643	Both	3
	HOH418	2.50	-350	-0.033	1.21	-46	0.331	3.71	-395	-0.015	Protein B	28
	HOH420	1.04	116	0.416	0.88	-294	-0.408	1.92	-179	0.249	Protein A	23
	HOH421	2.56	369	0.684	1.05	-185	-0.156	3.61	184	0.681	Protein A	24
	HOH423	2.26	-214	0.140	1.26	-14	0.343	3.51	-228	0.226	Protein B	11
	HOH434	1.16	-382	-0.079	1.04	-114	-0.003	2.20	-496	-0.303	Neither	0
	HOH436	2.25	47	0.590	1.24	-83	0.338	3.49	-36	0.827	Both	0
	HOH864	3.03	501	0.760	2.79	55	0.704	5.82	556	1.039	Both	3
	HOH876	1.89	153	0.499	1.03	-151	-0.081	2.93	3	0.729	Protein A	5
	HOH877	1.13	-150	0.154	1.22	395	0.388	2.35	244	0.617	Protein B	11
	HOH880	2.35	-10	0.611	1.32	190	0.428	3.67	180	0.683	Both	8
	HOH881	1.00	30	0.272	1.03	39	0.284	2.03	69	0.541	Both	4
	HOH991	0.94	-49	0.202	1.14	-445	-0.157	2.08	-494	-0.310	Neither	2
	HOH992	2.40	179	0.570	2.58	-334	-0.001	4.98	-155	-0.081	Protein A	27
	HOH993	3.93	-2	0.575	1.43	130	0.411	5.36	128	0.449	Both	10
	HOH994	0.99	-75	0.189	2.22	-58	0.581	3.21	-132	0.329	Protein B	3
	HOH995	2.11	2	0.556	1.25	-171	0.155	3.36	-169	0.294	Protein A	1
	HOH996	0.00	-189	-0.091	1.92	155	0.503	1.92	-34	0.513	Protein B	16
	HOH997	1.14	9	0.313	1.21	141	0.380	2.35	150	0.542	Both	23
	HOH1011	0.00	-36	-0.039	0.85	47	0.259	0.85	10	0.232	Protein B	8
	HOH1013	1.32	197	0.431	2.51	-34	0.644	3.83	163	0.680	Both	2
	HOH1027	0.89	-11	0.221	1.03	28	0.276	1.92	17	0.515	Protein B	0
	HOH1028	1.29	17	0.355	3.82	122	0.643	5.11	139	0.466	Both	0
	HOH1031	0.94	-209	-0.208	1.10	148	0.366	2.04	-61	0.539	Protein B	0
	HOH1047	0.00	-239	-0.137	1.89	79	0.508	1.89	-160	0.262	Protein B	11
	HOH1048	1.27	-23	0.348	2.19	27	0.577	3.47	3	0.825	Both	31
3AN1	HOH3	1.10	86	0.329	0.82	-618	-1.294	1.92	-531	-0.162	Protein A	0
	HOH6	1.07	-260	-0.325	0.82	2	0.220	1.89	-258	0.167	Neither	24
	HOH11	0.98	-92	0.173	0.85	-108	0.137	1.83	-200	0.216	Neither	1
	HOH14	2.09	-79	0.550	0.00	-269	-0.166	2.09	-348	-0.069	Protein A	11
	HOH19	1.19	16	0.326	2.17	-379	-0.109	3.36	-363	0.014	Protein A	16
	HOH20	0.00	24	-0.038	1.80	-73	0.484	1.80	-49	0.485	Protein B	26
	HOH26	1.09	-32	0.240	0.86	3	0.227	1.95	-29	0.521	Neither	45
	HOH29	1.18	78	0.324	1.04	58	0.299	2.22	136	0.516	Both	2
	HOH33	2.26	33	0.592	0.00	-130	-0.041	2.26	-97	0.587	Protein A	4
	HOH34	2.44	-166	0.220	1.10	16	0.278	3.54	-150	0.331	Protein B	6
	HOH46	0.98	-150	-0.079	0.00	-64	-0.039	0.98	-214	-0.220	Neither	5

HOH54	3.91	-122	-0.012	1.41	-137	0.211	5.32	-259	-0.314	Neither	1
HOH103	1.44	-175	0.181	3.97	139	0.465	5.41	-36	0.628	Protein B	0
HOH124	1.21	-110	0.202	1.17	-602	-0.365	2.39	-712	-0.715	Neither	3
HOH129	1.27	-207	0.124	2.13	-12	0.561	3.40	-219	0.230	Protein B	12
HOH174	1.18	-121	0.187	1.16	174	0.391	2.34	54	0.610	Protein B	1
HOH184	2.69	-29	0.681	2.56	-66	0.654	5.25	-95	0.564	Both	1
HOH199	2.69	687	0.694	1.02	-268	-0.345	3.71	419	0.865	Protein A	26
HOH223	1.85	-415	-0.018	0.00	-57	-0.039	1.85	-473	-0.091	Neither	23
HOH226	1.14	-20	0.312	0.86	-447	-0.800	2.00	-468	-0.272	Protein A	16
HOH249	1.53	176	0.455	4.15	-131	-0.030	5.68	46	0.710	Protein A	22
HOH251	1.18	186	0.401	1.16	-123	0.182	2.34	63	0.610	Protein A	1
HOH261	0.00	-54	-0.039	0.00	-463	-0.377	0.00	-517	-0.445	Neither	3
HOH298	2.24	-80	0.583	0.98	-97	0.169	3.22	-176	0.273	Protein A	25
HOH318	2.10	2	0.555	1.01	7	0.257	3.11	9	0.763	Both	1
HOH345	1.40	120	0.401	2.22	-10	0.582	3.63	111	0.619	Both	2
HOH360	1.05	-257	-0.319	0.88	-380	-0.623	1.93	-637	-0.318	Neither	14
HOH392	0.00	-71	-0.039	0.72	33	0.226	0.72	-39	0.171	Neither	2
HOH395	1.24	314	0.383	1.17	40	0.321	2.41	354	0.655	Both	13
HOH433	2.70	656	0.696	0.99	-3	0.246	3.69	652	0.873	Protein A	21
HOH452	0.73	30	0.224	0.00	-75	-0.039	0.73	-45	0.167	Neither	0
HOH462	1.04	-230	-0.257	0.00	-157	-0.064	1.04	-388	-0.641	Neither	44
HOH471	1.06	279	0.626	0.90	31	0.254	1.96	309	0.548	Both	55
HOH479	2.56	-85	0.653	2.65	-39	0.674	5.22	-124	-0.015	Both	1
HOH506	1.10	381	0.358	0.00	-17	-0.038	1.10	364	0.356	Protein A	19
HOH550	1.17	236	0.354	1.06	-66	0.207	2.23	170	0.545	Protein A	0
HOH577	1.21	88	0.331	2.13	-125	0.240	3.34	-37	0.803	Protein A	3
HOH611	2.58	113	0.533	2.60	21	0.666	5.18	134	0.458	Both	6
HOH655	1.07	16	0.273	0.81	-458	-0.830	1.88	-442	-0.047	Protein A	1
HOH673	2.44	52	0.632	1.11	37	0.303	3.55	89	0.841	Both	6
HOH683	1.21	-120	0.193	2.14	-279	0.038	3.35	-399	-0.044	Neither	35
HOH688	1.01	-283	-0.381	0.00	-215	-0.114	1.01	-498	-0.941	Neither	7
HOH749	1.13	82	0.310	0.90	-54	0.190	2.03	28	0.538	Protein A	2
HOH763	0.00	-216	-0.115	0.75	29	0.228	0.75	-187	-0.160	Neither	4
HOH831	0.83	-75	0.160	0.00	-201	-0.102	0.83	-276	-0.363	Neither	6
HOH838	1.21	-223	0.100	1.00	9	0.256	2.21	-214	0.135	Protein B	32
HOH840	0.00	-4	-0.038	0.00	-393	-0.295	0.00	-397	-0.300	Neither	12
HOH974	1.13	380	0.364	0.00	-210	-0.110	1.13	170	0.383	Protein A	2
HOH991	2.32	281	0.621	0.00	-436	-0.345	2.32	-155	0.222	Protein A	31
HOH1009	3.38	242	0.779	0.00	2	-0.038	3.38	244	0.779	Protein A	0
HOH1013	0.82	-47	0.182	0.00	-221	-0.120	0.82	-268	-0.346	Neither	4
HOH1039	2.81	360	0.723	2.21	-28	0.579	5.02	332	0.748	Both	9
HOH1076	2.23	17	0.586	1.02	143	0.459	3.25	160	0.633	Both	27
HOH1121	0.82	47	0.253	0.00	-238	-0.136	0.82	-191	-0.170	Protein A	10
HOH1175	3.55	152	0.651	1.33	18	0.364	4.88	170	0.514	Both	32
HOH1313	0.80	-635	-1.346	1.08	89	0.328	1.88	-545	-0.186	Protein B	0
HOH1365	4.14	-150	-0.071	1.52	175	0.453	5.66	25	0.691	Protein B	0
HOH1378	1.39	-135	0.209	3.91	-122	-0.012	5.29	-256	-0.309	Neither	1
HOH1405	0.00	-136	-0.046	2.22	13	0.583	2.22	-123	0.252	Protein B	9
HOH1412	1.08	-101	0.183	3.58	-73	0.840	4.66	-174	-0.122	Protein B	3
HOH1422	1.08	17	0.276	2.44	-158	0.230	3.52	-140	0.341	Protein A	19
HOH1433	0.00	-53	-0.039	1.00	-139	-0.057	1.00	-192	-0.171	Neither	43
HOH1464	0.84	0	0.222	1.08	-279	-0.370	1.92	-279	0.149	Neither	0
HOH1482	2.01	16	0.534	1.27	-21	0.347	3.28	-5	0.793	Both	0
HOH1497	1.21	34	0.331	3.76	73	0.874	4.97	107	0.414	Both	16
HOH1505	0.98	-65	0.195	2.44	257	0.637	3.43	192	0.674	Protein B	2
HOH1539	2.14	-12	0.563	1.23	-203	0.122	3.37	-215	0.234	Protein A	36
HOH1557	0.00	-213	-0.113	0.81	-56	0.173	0.81	-269	-0.349	Neither	2
HOH1607	0.00	-159	-0.066	1.01	-239	-0.277	1.01	-398	-0.670	Neither	5
HOH1611	3.98	125	0.442	1.45	-140	0.215	5.43	-14	0.648	Protein A	4
HOH1615	1.01	-274	-0.359	2.60	658	0.676	3.61	384	0.845	Protein B	14
HOH1622	0.93	-192	-0.171	1.15	-107	0.313	2.08	-299	0.004	Protein B	1
HOH1642	0.00	79	-0.037	2.03	32	0.540	2.03	110	0.473	Protein B	6
HOH1669	0.00	-196	-0.098	0.84	-77	0.162	0.84	-273	-0.357	Neither	6
HOH1716	0.00	-324	-0.221	0.83	-365	-0.584	0.83	-689	-1.517	Neither	38
HOH1729	0.91	-47	0.198	1.12	-38	0.305	2.03	-84	0.536	Protein B	36
HOH1733	0.86	-67	0.173	1.12	81	0.306	1.97	15	0.528	Protein B	21

	HOH1766	1.04	-7	0.252	2.70	673	0.696	3.74	666	0.882	Both	38
	HOH1779	1.20	-603	-0.364	1.20	-123	0.188	2.39	-726	-0.746	Neither	26
	HOH1790	0.84	-409	-0.698	1.13	-15	0.309	1.97	-424	-0.013	Protein B	3
	HOH1802	0.85	-416	-0.716	1.05	-243	-0.285	1.90	-658	-0.355	Neither	29
	HOH1867	0.00	-468	-0.383	0.00	-52	-0.039	0.00	-520	-0.447	Neither	1
	HOH1885	1.20	48	0.328	1.21	287	0.371	2.40	335	0.649	Both	34
	HOH1888	1.00	102	0.394	2.24	-5	0.587	3.24	97	0.578	Both	0
	HOH1908	0.00	-277	-0.174	2.11	-91	0.555	2.11	-368	-0.097	Protein B	7
	HOH1909	2.11	-135	0.226	1.26	103	0.366	3.37	-32	0.808	Protein B	5
	HOH1947	1.04	65	0.304	1.22	89	0.337	2.26	154	0.536	Both	21
	HOH2001	0.00	-37	-0.039	0.00	-21	-0.038	0.00	-58	-0.039	Neither	0
	HOH2077	0.92	27	0.256	2.43	178	0.573	3.35	205	0.678	Both	17
	HOH2079	1.00	-3	0.248	2.10	-220	0.117	3.11	-223	0.203	Neither	21
	HOH2105	0.00	40	-0.037	3.44	115	0.609	3.44	155	0.644	Protein B	26
	HOH2106	2.16	-262	0.065	1.20	-114	0.197	3.36	-375	-0.005	Neither	23
	HOH2144	0.81	-455	-0.822	1.10	17	0.301	1.91	-438	-0.039	Protein B	54
	HOH2184	0.86	27	0.246	1.04	255	0.597	1.90	283	0.531	Protein B	39
	HOH2245	2.66	21	0.677	2.58	106	0.527	5.24	127	0.447	Both	24
	HOH2325	0.93	-81	0.173	1.11	579	0.307	2.04	498	0.615	Protein B	20
	HOH2338	0.00	-441	-0.350	2.34	336	0.638	2.34	-105	0.605	Protein B	6
	HOH2387	2.33	-20	0.605	1.39	138	0.410	3.72	118	0.633	Both	6
	HOH2482	0.72	-40	0.169	0.00	-37	-0.039	0.72	-77	0.139	Neither	0
3NA5	HOH14	5.05	-141	-0.051	0.00	-242	-0.140	5.05	-384	-0.624	Neither	20
	HOH84	1.05	37	0.286	1.02	41	0.284	2.08	78	0.551	Both	25
	HOH89	2.34	50	0.610	0.00	-130	-0.042	2.34	-80	0.605	Protein A	7
	HOH94	0.82	-198	-0.185	0.00	-45	-0.039	0.82	-242	-0.286	Neither	5
	HOH109	1.24	-211	0.115	2.34	4	0.608	3.57	-207	0.259	Protein B	0
	HOH117	3.57	-35	0.840	0.00	-155	-0.062	3.57	-190	0.282	Protein A	9
	HOH165	1.32	-129	0.203	2.50	77	0.645	3.82	-53	0.878	Protein B	34
	HOH198	1.17	446	0.383	1.15	-209	0.103	2.32	237	0.609	Protein A	26
	HOH226	0.00	-60	-0.039	0.89	22	0.247	0.89	-38	0.201	Neither	13
	HOH241	1.41	552	0.392	2.37	-256	0.093	3.78	296	0.845	Protein A	6
	HOH283	1.08	-116	-0.008	2.22	-151	0.217	3.29	-267	0.156	Neither	11
	HOH305	0.95	-38	0.212	0.90	-237	-0.274	1.85	-275	0.144	Neither	10
	HOH332	2.16	-208	0.139	0.00	-102	-0.040	2.16	-310	-0.004	Neither	6
	HOH334	2.37	273	0.628	0.96	-52	0.202	3.33	221	0.765	Protein A	16
	HOH351	1.97	-130	0.301	0.00	-401	-0.304	1.97	-531	-0.155	Protein A	0
	HOH366	2.22	-138	0.233	1.01	-347	-0.538	3.22	-485	-0.201	Neither	0
	HOH367	2.40	-371	-0.075	1.04	-88	0.186	3.43	-459	-0.140	Neither	27
	HOH389	0.90	102	0.393	0.00	-140	-0.050	0.90	-38	0.202	Protein A	53
	HOH409	0.94	98	0.387	0.00	-62	-0.039	0.94	36	0.267	Protein A	35
	HOH415	1.05	175	0.508	0.00	-56	-0.039	1.05	119	0.421	Protein A	32
	HOH469	0.83	-150	-0.080	0.85	27	0.244	1.67	-123	0.264	Neither	19
	HOH494	2.41	504	0.634	1.11	-97	0.301	3.52	408	0.837	Both	6
	HOH504	0.97	-63	0.195	2.39	275	0.633	3.36	212	0.768	Protein B	0
	HOH512	0.00	-127	-0.039	2.33	-10	0.606	2.33	-136	0.246	Protein B	11
	HOH551	2.21	-287	0.035	1.39	570	0.384	3.60	283	0.819	Protein B	0
	HOH564	0.00	-256	-0.153	3.75	-164	0.328	3.75	-419	-0.052	Protein B	27
	HOH567	1.01	38	0.280	1.06	33	0.284	2.07	70	0.550	Both	8
	HOH582	3.79	39	0.877	1.45	-194	0.165	5.24	-155	-0.080	Protein A	2
	HOH585	1.15	58	0.316	2.45	-161	0.227	3.60	-104	0.843	Protein A	32
	HOH646	1.13	-325	-0.019	1.20	591	0.334	2.33	266	0.619	Protein B	10
	HOH746	0.95	-161	-0.103	1.00	-22	0.232	1.95	-183	0.249	Neither	3
	HOH763	0.00	-109	-0.040	3.51	-154	0.324	3.51	-263	0.177	Protein B	18
	HOH841	2.44	88	0.632	1.35	-158	0.183	3.79	-70	0.874	Protein A	15
	HOH862	0.00	-272	-0.168	2.58	468	0.708	2.58	196	0.602	Protein B	17
	HOH895	1.05	-71	0.201	2.42	-363	-0.060	3.46	-434	-0.095	Neither	0
	HOH901	2.52	130	0.542	2.55	115	0.531	5.06	245	0.644	Both	0
	HOH906	0.95	-337	-0.512	1.16	-84	0.317	2.12	-420	-0.181	Protein B	0
	HOH930	0.86	-23	0.208	0.90	-189	-0.164	1.76	-211	0.195	Neither	0
	HOH943	0.00	-417	-0.322	1.96	-117	0.311	1.96	-533	-0.159	Protein B	9
	HOH947	0.00	-70	-0.039	0.94	98	0.387	0.94	28	0.260	Protein B	8
	HOH952	1.13	515	0.313	1.15	-168	0.140	2.27	347	0.629	Protein A	17
	HOH1001	1.12	-150	0.152	1.16	503	0.322	2.29	353	0.632	Protein B	56
	HOH1009	1.03	-67	0.203	2.38	602	0.628	3.41	536	0.827	Protein B	22
3NT1	HOH35	2.16	-374	-0.102	0.99	-5	0.244	3.14	-379	-0.027	Neither	24

HOH48	1.17	290	0.363	1.11	-11	0.304	2.28	279	0.614	Both	29
HOH60	1.61	-91	0.436	0.00	-87	-0.039	1.61	-178	0.205	Protein A	33
HOH66	3.75	313	0.845	1.23	24	0.336	4.97	338	0.754	Both	4
HOH70	4.02	-309	-0.438	1.45	-5	0.396	5.48	-314	-0.448	Protein B	2
HOH71	1.35	-155	0.185	2.26	-183	0.181	3.61	-338	0.072	Neither	0
HOH72	1.23	-24	0.337	0.93	-215	-0.223	2.16	-240	0.096	Protein A	0
HOH73	1.20	124	0.369	2.33	22	0.607	3.53	147	0.645	Both	0
HOH74	2.21	-128	0.244	1.08	71	0.315	3.29	-57	0.793	Protein B	5
HOH77	1.10	-100	0.300	0.83	-36	0.192	1.93	-136	0.290	Protein A	22
HOH78	1.14	-128	0.174	2.05	77	0.546	3.19	-51	0.776	Protein B	21
HOH79	1.83	-21	0.491	0.00	49	-0.037	1.83	28	0.492	Protein A	52
HOH90	2.56	-268	0.095	1.26	180	0.412	3.82	-88	0.878	Protein B	37
HOH91	3.50	211	0.786	0.00	-42	-0.039	3.50	170	0.662	Protein A	0
HOH92	3.86	315	0.860	1.18	-62	0.321	5.04	253	0.653	Both	26
HOH100	0.72	-16	0.188	0.00	-179	-0.083	0.72	-195	-0.179	Neither	4
HOH135	2.04	-134	0.219	0.00	-109	-0.040	2.04	-243	0.079	Neither	14
HOH162	0.89	138	0.451	0.00	-139	-0.049	0.89	-1	0.230	Protein A	0
HOH163	3.33	-49	0.801	0.00	-94	-0.040	3.33	-143	0.325	Protein A	21
HOH164	3.48	124	0.621	0.00	-52	-0.039	3.48	73	0.829	Protein A	16
HOH165	1.26	85	0.346	2.55	-61	0.651	3.81	24	0.879	Both	2
HOH166	1.10	-86	0.300	0.92	-204	-0.198	2.02	-290	0.011	Protein A	13
HOH170	1.24	75	0.341	0.96	-17	0.229	2.20	57	0.579	Protein A	32
HOH175	2.20	5	0.579	0.00	-3	-0.038	2.20	2	0.579	Protein A	23
HOH190	1.18	74	0.325	0.88	-29	0.206	2.06	45	0.547	Protein A	5
HOH207	0.97	65	0.293	0.85	-159	-0.100	1.82	-94	0.489	Protein A	36
HOH211	2.65	-33	0.673	1.28	-136	0.190	3.93	-169	-0.113	Protein A	4
HOH231	1.06	510	0.485	0.00	-263	-0.160	1.06	247	0.587	Protein A	18
HOH235	2.47	237	0.635	1.18	-21	0.322	3.65	216	0.806	Both	36
HOH257	2.31	-106	0.599	0.95	-33	0.216	3.26	-139	0.324	Protein A	30
HOH279	2.43	-56	0.627	2.38	-39	0.615	4.81	-94	0.544	Both	17
HOH280	1.88	-45	0.504	0.00	-100	-0.040	1.88	-145	0.275	Protein A	0
HOH284	1.14	118	0.353	0.81	-32	0.193	1.95	86	0.523	Protein A	16
HOH388	2.16	169	0.537	1.04	-106	0.172	3.20	63	0.782	Protein A	19
HOH395	1.21	116	0.366	1.16	-9	0.318	2.37	107	0.507	Both	12
HOH396	0.00	55	-0.037	1.78	-26	0.479	1.78	29	0.480	Protein B	0
HOH408	1.29	266	0.387	2.02	-121	0.233	3.31	145	0.626	Protein A	0
HOH427	1.83	-122	0.288	0.00	-111	-0.026	1.83	-232	0.185	Protein A	0
HOH441	1.33	-59	0.362	2.53	132	0.544	3.85	74	0.887	Both	2
HOH498	0.95	7	0.245	0.00	-100	-0.040	0.95	-94	0.166	Neither	1
HOH525	0.93	29	0.259	0.89	11	0.239	1.81	41	0.489	Protein A	0
HOH528	0.85	-21	0.208	1.13	108	0.346	1.98	87	0.530	Protein B	0
HOH531	3.65	-135	0.357	1.39	90	0.381	5.03	-45	0.603	Both	2
HOH545	0.00	-92	-0.040	0.74	-85	0.136	0.74	-177	-0.140	Neither	5
HOH548	1.12	-4	0.307	1.18	307	0.368	2.30	303	0.624	Both	24
HOH557	0.00	-43	-0.039	3.50	210	0.785	3.50	167	0.660	Protein B	16
HOH558	1.21	128	0.373	2.53	-242	0.128	3.73	-114	0.390	Protein A	13
HOH560	1.19	-64	0.324	3.85	313	0.858	5.04	249	0.649	Both	3
HOH561	1.29	-138	0.191	2.67	-17	0.677	3.96	-155	-0.083	Protein B	0
HOH564	1.01	6	0.255	2.10	-370	-0.102	3.11	-364	-0.005	Protein A	1
HOH565	1.08	33	0.287	2.20	-242	0.097	3.28	-209	0.235	Protein A	11
HOH566	0.00	-64	-0.039	3.52	63	0.836	3.52	-1	0.834	Protein B	14
HOH567	1.04	-130	-0.038	2.19	-45	0.573	3.23	-175	0.276	Protein B	37
HOH568	0.82	-137	-0.054	0.97	109	0.404	1.79	-29	0.483	Protein B	20
HOH569	1.06	-115	-0.005	2.09	155	0.517	3.15	40	0.772	Protein B	5
HOH578	0.00	-180	-0.084	0.89	-230	-0.258	0.89	-410	-0.702	Neither	23
HOH603	0.94	-49	0.202	2.29	-114	0.269	3.23	-163	0.292	Protein B	9
HOH605	2.08	88	0.553	1.14	-125	0.176	3.22	-37	0.782	Protein A	0
HOH606	1.18	-253	0.065	1.20	28	0.328	2.37	-225	0.137	Protein B	51
HOH607	0.93	-41	0.206	2.47	304	0.653	3.40	263	0.786	Protein B	0
HOH608	1.06	-51	0.219	2.26	6	0.590	3.31	-46	0.798	Protein B	38
HOH609	2.26	12	0.592	1.17	100	0.349	3.44	111	0.606	Both	0
HOH610	0.92	156	0.478	1.14	70	0.313	2.06	225	0.558	Both	0
HOH644	0.00	-4	-0.038	2.21	13	0.581	2.21	9	0.581	Protein B	7
HOH646	0.92	-228	-0.252	1.12	-87	0.306	2.04	-314	-0.022	Protein B	9
HOH650	0.96	-18	0.229	1.19	69	0.327	2.15	51	0.568	Protein B	4
HOH702	0.90	22	0.249	1.21	192	0.410	2.11	214	0.565	Protein B	24

	HOH715	0.85	-155	-0.091	0.98	64	0.293	1.83	-91	0.490	Protein B	0
	HOH727	1.44	-26	0.392	4.02	-297	-0.408	5.46	-323	-0.470	Protein A	0
	HOH739	1.21	10	0.330	3.73	343	0.851	4.94	353	0.771	Both	41
	HOH741	0.00	-114	-0.029	1.90	-21	0.510	1.90	-135	0.287	Protein B	26
	HOH742	3.59	-188	0.285	1.42	-162	0.190	5.02	-350	-0.539	Protein A	0
	HOH743	2.69	163	0.586	2.69	172	0.593	5.38	334	0.753	Both	3
	HOH746	0.00	-141	-0.050	1.85	-97	0.496	1.85	-238	0.182	Protein B	22
	HOH751	0.00	-280	-0.177	1.01	469	0.819	1.01	189	0.528	Protein B	29
	HOH793	0.00	6	-0.038	0.00	-166	-0.072	0.00	-160	-0.066	Neither	21
	HOH797	0.00	-5	-0.038	1.06	42	0.290	1.06	36	0.287	Protein B	42
	HOH839	0.00	-182	-0.085	0.00	-263	-0.160	0.00	-445	-0.355	Neither	11
	HOH880	0.91	-222	-0.237	1.23	-16	0.336	2.14	-238	0.097	Protein B	10
	HOH883	1.13	-35	0.308	2.50	229	0.638	3.62	194	0.691	Both	0
	HOH907	1.08	6	0.268	1.24	322	0.385	2.32	329	0.633	Both	12
	HOH934	0.00	-292	-0.188	1.91	125	0.483	1.91	-167	0.258	Protein B	12
	HOH953	2.34	57	0.611	1.36	4	0.371	3.70	60	0.864	Both	0
	HOH1001	0.00	16	-0.038	1.05	203	0.548	1.05	219	0.551	Protein B	21
	HOH1014	0.00	-97	-0.040	0.98	-44	0.212	0.98	-141	-0.061	Neither	6
3NT6	HOH25	1.17	-375	-0.070	1.12	-80	0.304	2.28	-455	-0.224	Protein B	15
	HOH26	1.19	-608	-0.372	1.18	296	0.367	2.38	-312	0.014	Protein B	2
	HOH28	2.14	-58	0.562	0.00	-63	-0.039	2.14	-121	0.246	Protein A	22
	HOH30	3.90	-73	0.506	1.51	304	0.446	5.40	231	0.628	Both	0
	HOH49	2.62	-42	0.667	1.34	381	0.418	3.97	339	0.748	Both	20
	HOH53	2.42	-105	0.623	1.18	-380	-0.073	3.60	-485	-0.175	Protein A	10
	HOH54	1.01	55	0.292	0.00	-63	-0.039	1.01	-8	0.246	Protein A	0
	HOH56	4.37	234	0.625	1.48	108	0.405	5.85	342	0.764	Both	11
	HOH58	2.21	31	0.581	2.59	20	0.663	4.80	51	0.677	Both	0
	HOH59	1.52	317	0.451	3.89	-14	0.561	5.42	303	0.717	Both	23
	HOH74	0.99	-74	0.188	0.00	-41	-0.039	0.99	-115	-0.006	Neither	14
	HOH76	1.23	33	0.336	1.07	-467	-0.853	2.30	-434	-0.187	Protein A	0
	HOH105	1.27	55	0.350	2.37	111	0.510	3.64	166	0.669	Both	1
	HOH108	1.13	-422	-0.131	0.89	-58	0.185	2.02	-480	-0.292	Neither	1
	HOH118	2.14	-99	0.562	0.98	-61	0.199	3.13	-160	0.288	Protein A	24
	HOH120	2.93	464	0.764	2.59	-127	0.282	5.52	337	0.757	Both	9
	HOH122	1.35	-196	0.147	2.13	68	0.564	3.48	-128	0.354	Protein B	36
	HOH123	5.54	581	1.036	0.00	-39	-0.039	5.54	541	1.024	Protein A	1
	HOH128	1.34	382	0.416	1.09	-618	-1.294	2.43	-236	0.127	Protein A	0
	HOH131	2.36	566	0.624	0.00	-185	-0.088	2.36	381	0.653	Protein A	3
	HOH132	2.34	443	0.662	0.00	-311	-0.207	2.34	132	0.525	Protein A	5
	HOH134	1.12	-284	0.023	0.00	-68	-0.039	1.12	-352	-0.050	Neither	0
	HOH136	2.35	-19	0.609	1.17	150	0.379	3.52	131	0.629	Both	8
	HOH157	0.97	-213	-0.218	0.00	2	-0.038	0.97	-212	-0.215	Neither	28
	HOH161	4.35	453	0.865	1.49	218	0.427	5.84	670	1.067	Both	4
	HOH164	5.44	110	0.420	0.00	-46	-0.039	5.44	63	0.716	Protein A	0
	HOH167	2.10	131	0.498	0.00	-338	-0.235	2.10	-207	0.133	Protein A	0
	HOH189	2.05	38	0.544	0.00	-285	-0.182	2.05	-248	0.073	Protein A	1
	HOH229	1.26	452	0.405	0.88	-11	0.221	2.14	440	0.623	Protein A	1
	HOH261	2.25	-99	0.586	1.34	-12	0.366	3.59	-111	0.383	Both	0
	HOH263	1.11	-569	-0.327	1.13	19	0.310	2.24	-551	-0.399	Protein B	0
	HOH270	0.00	16	-0.038	0.96	-249	-0.300	0.96	-233	-0.263	Neither	1
	HOH273	0.00	-33	-0.038	5.66	536	1.028	5.66	503	1.017	Protein B	2
	HOH274	0.00	-77	-0.039	1.09	-402	-0.680	1.09	-479	-0.887	Neither	0
	HOH281	1.38	-355	-0.013	3.45	-185	0.280	4.83	-540	-1.056	Protein B	0
	HOH283	2.47	12	0.638	2.68	-241	0.142	5.16	-229	-0.246	Protein A	1
	HOH287	0.00	-275	-0.171	0.98	51	0.284	0.98	-224	-0.242	Protein B	18
	HOH288	1.34	54	0.369	4.39	216	0.600	5.73	270	0.679	Both	3
	HOH289	3.52	20	0.835	1.37	116	0.392	4.89	136	0.461	Both	18
	HOH292	1.29	106	0.372	3.56	14	0.840	4.84	119	0.435	Both	0
	HOH293	1.44	85	0.393	4.18	45	0.634	5.62	130	0.453	Both	10
	HOH295	0.85	-58	0.179	1.09	-422	-0.731	1.95	-479	-0.087	Neither	1
	HOH308	0.94	-536	-1.049	1.21	405	0.389	2.16	-131	0.235	Protein B	47
	HOH310	1.32	388	0.414	2.54	-164	0.232	3.87	224	0.835	Protein A	18
	HOH315	0.00	-51	-0.039	0.93	-18	0.225	0.93	-68	0.184	Neither	0
	HOH318	1.23	45	0.337	2.38	-2	0.617	3.61	43	0.849	Both	9
	HOH320	2.24	102	0.488	1.20	20	0.329	3.44	122	0.616	Both	20
	HOH322	1.06	-78	0.198	1.22	-369	-0.053	2.28	-447	-0.211	Neither	5

	HOH323	0.00	-203	-0.104	2.35	517	0.622	2.35	313	0.636	Protein B	30
	HOH324	0.00	-310	-0.206	3.82	338	0.860	3.82	28	0.882	Protein B	19
	HOH329	0.00	-18	-0.038	1.74	226	0.487	1.74	207	0.507	Protein B	0
	HOH331	0.00	-402	-0.305	1.97	94	0.473	1.97	-309	0.124	Protein B	13
	HOH332	1.08	-72	0.206	2.56	314	0.672	3.64	242	0.813	Protein B	0
	HOH334	2.52	5	0.647	2.26	45	0.592	4.77	50	0.674	Both	1
	HOH338	0.93	-13	0.227	1.21	-197	0.124	2.14	-210	0.133	Neither	31
	HOH354	0.96	-57	0.199	2.16	-20	0.568	3.12	-77	0.763	Protein B	22
	HOH394	0.00	-449	-0.360	1.16	84	0.318	1.16	-365	-0.059	Protein B	28
	HOH418	1.05	-20	0.243	2.24	100	0.486	3.29	80	0.797	Protein B	1
	HOH424	2.41	-233	0.128	1.31	33	0.359	3.72	-200	0.278	Protein B	24
	HOH446	0.00	-71	-0.039	2.06	45	0.546	2.06	-26	0.543	Protein B	37
	HOH462	1.19	217	0.356	2.28	-352	-0.056	3.46	-135	0.345	Protein A	0
	HOH463	1.57	189	0.469	4.43	563	0.966	6.00	751	1.086	Both	25
	HOH467	0.00	-43	-0.039	2.46	588	0.645	2.46	545	0.644	Protein B	24
	HOH481	0.00	-478	-0.395	1.73	-17	0.467	1.73	-495	-0.137	Protein B	13
	HOH484	1.07	74	0.316	1.29	303	0.394	2.36	377	0.651	Both	32
	HOH509	0.00	-315	-0.212	2.03	-12	0.537	2.03	-327	-0.043	Protein B	2
	HOH524	0.00	-32	-0.038	0.86	243	0.577	0.86	211	0.536	Protein B	8
	HOH533	0.98	-29	0.225	2.11	8	0.556	3.09	-21	0.759	Protein B	0
	HOH539	1.17	287	0.362	1.18	-50	0.322	2.35	237	0.614	Both	14
	HOH541	0.00	-160	-0.066	2.21	-93	0.577	2.21	-253	0.082	Protein B	10
3NZ4	HOH8	1.08	146	0.465	0.00	-114	-0.029	1.08	32	0.287	Protein A	0
	HOH26	2.22	28	0.583	0.00	-147	-0.056	2.22	-119	0.256	Protein A	2
	HOH38	3.55	-185	0.287	0.00	-197	-0.099	3.55	-382	-0.003	Protein A	12
	HOH59	1.39	-334	0.011	2.54	121	0.536	3.93	-214	-0.212	Protein B	20
	HOH85	1.17	22	0.320	1.12	-25	0.306	2.29	-3	0.598	Both	5
	HOH137	4.17	-19	0.577	1.22	-245	0.079	5.39	-264	-0.326	Protein A	1
	HOH139	1.18	-555	-0.298	3.48	-191	0.274	4.66	-746	-1.702	Protein B	25
	HOH142	3.57	-232	0.225	0.00	72	-0.037	3.57	-160	0.320	Neither	8
	HOH159	0.00	-180	-0.084	2.17	17	0.572	2.17	-163	0.198	Protein B	2
	HOH166	0.89	68	0.281	0.98	164	0.491	1.88	232	0.517	Both	0
	HOH191	1.09	-140	-0.058	3.70	-169	0.318	4.79	-309	-0.435	Protein B	1
	HOH192	0.91	-155	-0.091	1.04	37	0.284	1.95	-118	0.308	Protein B	2
	HOH200	1.01	-461	-0.838	2.49	47	0.643	3.50	-414	-0.059	Protein B	39
	HOH201	1.16	16	0.318	2.37	-96	0.613	3.54	-79	0.834	Both	24
	HOH207	1.15	-235	0.078	4.25	34	0.629	5.40	-201	-0.182	Protein B	31
	HOH219	2.50	-156	0.238	2.83	126	0.568	5.33	-30	0.629	Protein B	1
	HOH226	3.62	-194	0.279	1.12	-473	-0.195	4.74	-667	-1.445	Protein A	6
	HOH229	0.00	-114	-0.028	1.02	149	0.468	1.02	35	0.280	Protein B	38
	HOH268	1.19	-23	0.326	1.34	9	0.368	2.54	-14	0.650	Both	32
3NZP	HOH31	0.93	-598	-1.232	0.93	-296	-0.411	1.86	-893	-0.783	Neither	2
	HOH35	2.64	464	0.717	1.09	17	0.278	3.73	481	0.880	Both	34
	HOH51	0.77	136	0.447	0.00	-30	-0.038	0.77	106	0.399	Protein A	1
	HOH54	1.15	274	0.356	1.96	-91	0.523	3.11	183	0.641	Both	4
	HOH56	1.15	46	0.315	0.98	31	0.270	2.13	77	0.564	Both	3
	HOH64	2.52	-186	0.202	1.32	17	0.361	3.83	-169	0.327	Protein B	12
	HOH76	2.75	595	0.706	2.32	-252	0.095	5.08	344	0.762	Protein A	16
	HOH78	1.80	104	0.454	0.00	-499	-0.421	1.80	-395	0.000	Protein A	0
	HOH79	1.05	131	0.440	1.16	-63	0.316	2.21	68	0.581	Both	11
	HOH80	2.53	109	0.524	1.08	43	0.295	3.61	152	0.655	Both	42
	HOH83	2.15	-89	0.564	1.16	97	0.345	3.31	8	0.800	Both	27
	HOH94	1.98	-60	0.526	1.20	270	0.366	3.18	210	0.741	Both	43
	HOH96	0.94	174	0.506	0.98	49	0.282	1.92	223	0.525	Both	10
	HOH97	1.08	33	0.288	2.26	-90	0.588	3.34	-57	0.801	Both	5
	HOH130	3.63	-17	0.851	1.18	95	0.346	4.81	78	0.700	Both	19
	HOH132	1.05	78	0.316	2.06	17	0.545	3.11	95	0.565	Both	0
	HOH133	1.08	-59	0.216	2.13	110	0.484	3.21	51	0.782	Protein B	19
	HOH135	1.14	-146	0.159	2.17	-486	-0.287	3.31	-632	-0.478	Neither	7
	HOH143	0.88	-343	-0.528	1.95	36	0.521	2.83	-307	0.060	Protein B	23
	HOH150	0.00	40	-0.037	3.31	-120	0.351	3.31	-80	0.796	Protein B	64
	HOH162	1.10	35	0.300	1.22	-239	0.085	2.32	-204	0.159	Protein A	20
3ODU	HOH22	0.98	90	0.312	0.89	-47	0.194	1.87	44	0.503	Protein A	42
	HOH25	1.26	-14	0.343	2.30	90	0.603	3.55	76	0.842	Both	9
	HOH44	0.97	27	0.265	0.81	-40	0.187	1.79	-12	0.481	Protein A	44
	HOH73	1.28	291	0.389	1.10	140	0.360	2.37	430	0.665	Both	44

	HOH86	2.33	116	0.511	1.28	1	0.351	3.61	118	0.625	Both	7
3OOY	HOH1	5.20	352	0.772	0.00	-11	-0.038	5.20	342	0.761	Protein A	0
	HOH4	4.02	103	0.407	1.22	-105	0.331	5.24	-2	0.652	Both	0
	HOH11	2.68	-162	0.247	2.40	-388	-0.102	5.08	-550	-1.087	Neither	41
	HOH12	1.07	1	0.263	0.00	-295	-0.191	1.07	-294	-0.406	Protein A	0
	HOH31	4.12	164	0.502	1.41	368	0.432	5.53	531	1.021	Both	22
	HOH32	4.06	-81	0.511	1.31	-113	0.216	5.38	-194	-0.164	Protein A	5
	HOH35	1.37	-85	0.374	2.20	-94	0.574	3.57	-179	0.296	Both	1
	HOH40	1.08	81	0.321	0.00	-287	-0.183	1.08	-206	-0.202	Protein A	0
	HOH43	2.13	-155	0.203	0.00	-403	-0.306	2.13	-558	-0.424	Neither	6
	HOH45	1.01	20	0.267	0.00	-482	-0.400	1.01	-461	-0.839	Protein A	1
	HOH49	0.98	-216	-0.224	0.00	-339	-0.237	0.98	-555	-1.106	Neither	39
	HOH54	5.04	260	0.662	0.00	-151	-0.059	5.04	109	0.418	Protein A	0
	HOH56	1.00	211	0.539	0.00	-339	-0.237	1.00	-128	-0.034	Protein A	15
	HOH57	0.70	-46	0.162	0.00	-54	-0.039	0.70	-100	0.117	Neither	48
	HOH64	0.90	-251	-0.305	0.00	-243	-0.141	0.90	-494	-0.930	Neither	8
	HOH73	0.99	-170	-0.122	2.23	218	0.588	3.22	48	0.784	Protein B	18
	HOH78	2.44	-25	0.629	1.29	-302	0.031	3.72	-327	0.096	Protein A	7
	HOH81	4.18	42	0.631	1.25	245	0.375	5.43	287	0.698	Both	6
	HOH93	1.04	283	0.631	0.00	-253	-0.150	1.04	30	0.279	Protein A	38
	HOH96	2.72	351	0.707	1.36	26	0.373	4.08	378	0.790	Both	21
	HOH100	1.29	355	0.402	1.00	-291	-0.400	2.29	64	0.600	Protein A	0
	HOH106	2.37	110	0.509	1.02	-6	0.248	3.39	103	0.595	Protein A	0
	HOH110	0.00	-246	-0.144	2.06	104	0.471	2.06	-143	0.211	Protein B	0
	HOH114	1.05	76	0.313	0.93	-150	-0.080	1.97	-74	0.526	Protein A	3
	HOH119	3.55	-92	0.835	0.00	-290	-0.186	3.55	-382	-0.003	Protein A	2
	HOH132	1.07	21	0.277	1.01	-466	-0.850	2.08	-445	-0.226	Protein A	2
	HOH160	1.07	-192	-0.170	0.00	-260	-0.157	1.07	-452	-0.812	Neither	0
	HOH163	1.17	674	0.324	1.09	47	0.300	2.26	721	0.601	Both	0
	HOH180	1.04	-259	-0.324	0.00	-199	-0.100	1.04	-458	-0.830	Neither	0
	HOH186	1.38	420	0.432	2.18	-79	0.571	3.56	341	0.829	Both	12
	HOH200	0.91	-68	0.181	0.87	159	0.482	1.78	91	0.481	Protein B	9
	HOH229	2.05	388	0.597	1.35	-340	-0.001	3.40	48	0.815	Protein A	0
	HOH239	0.88	-29	0.206	0.88	121	0.423	1.76	91	0.477	Protein B	0
	HOH255	1.07	-323	-0.478	0.00	-239	-0.137	1.07	-562	-1.126	Neither	0
	HOH257	0.90	-289	-0.395	0.00	-419	-0.325	0.90	-708	-1.578	Neither	0
	HOH259	0.00	-37	-0.039	0.71	-164	-0.111	0.71	-201	-0.193	Neither	7
	HOH263	1.10	431	0.364	2.18	-112	0.260	3.28	319	0.784	Both	3
	HOH278	0.88	-277	-0.367	1.27	16	0.348	2.15	-262	0.064	Protein B	0
	HOH281	1.45	412	0.448	4.14	209	0.589	5.60	621	1.048	Both	13
	HOH284	0.00	-13	-0.038	5.33	311	0.727	5.33	298	0.711	Protein B	10
	HOH286	0.00	-332	-0.229	1.04	90	0.322	1.04	-242	-0.283	Protein B	11
	HOH288	2.29	-117	0.266	2.54	313	0.667	4.82	197	0.553	Both	30
	HOH289	1.10	-297	0.007	1.18	271	0.363	2.29	-27	0.596	Protein B	1
	HOH293	0.00	-158	-0.065	5.03	258	0.660	5.03	100	0.403	Protein B	36
	HOH294	2.41	-348	-0.038	2.74	-171	0.241	5.15	-519	-0.996	Neither	4
	HOH298	1.27	230	0.378	4.11	-58	0.536	5.38	172	0.518	Both	11
	HOH306	0.00	-224	-0.123	1.01	245	0.583	1.01	20	0.266	Protein B	0
	HOH307	1.17	-258	0.057	1.22	390	0.389	2.39	132	0.530	Protein B	2
	HOH308	1.12	-95	0.305	3.93	82	0.646	5.05	-14	0.633	Both	5
	HOH309	2.56	274	0.661	2.29	-114	0.269	4.85	160	0.499	Both	20
	HOH310	0.00	-285	-0.181	3.43	-198	0.261	3.43	-483	-0.183	Protein B	0
	HOH324	0.00	-227	-0.126	2.01	-194	0.141	2.01	-421	-0.192	Neither	5
	HOH325	0.97	-199	-0.185	2.24	-303	0.014	3.22	-502	-0.231	Neither	0
	HOH338	1.27	-323	0.006	2.47	-105	0.633	3.74	-428	-0.068	Protein B	7
	HOH341	0.00	-256	-0.153	0.95	-308	-0.440	0.95	-563	-1.130	Neither	7
	HOH362	2.12	-70	0.558	1.30	544	0.360	3.42	475	0.838	Both	19
	HOH364	1.05	173	0.505	2.08	-175	0.173	3.13	-2	0.767	Protein A	30
	HOH383	0.95	-26	0.220	1.20	714	0.334	2.15	688	0.577	Protein B	0
	HOH387	1.27	-27	0.347	2.73	382	0.715	4.00	355	0.766	Both	32
	HOH395	0.99	-20	0.233	2.03	60	0.540	3.02	40	0.749	Protein B	17
	HOH402	0.91	-162	-0.105	1.02	69	0.304	1.93	-92	0.515	Protein B	9
	HOH407	2.27	-81	0.591	1.20	-188	0.130	3.47	-269	0.165	Protein A	5
	HOH410	0.00	-343	-0.240	1.06	222	0.555	1.06	-121	-0.019	Protein B	57
	HOH412	0.00	-252	-0.149	1.02	-219	-0.230	1.02	-471	-0.865	Neither	8
	HOH450	1.06	-236	-0.268	1.10	-233	0.071	2.16	-469	-0.259	Neither	8

	HOH461	1.09	-162	-0.106	1.15	-180	0.130	2.24	-342	-0.046	Neither	0
	HOH464	1.07	42	0.293	2.43	369	0.661	3.50	411	0.836	Both	0
	HOH471	2.65	-43	0.673	2.75	-43	0.692	5.40	-86	0.579	Both	30
	HOH474	0.76	-154	-0.088	0.00	-33	-0.038	0.76	-186	-0.159	Neither	11
	HOH483	1.09	-189	-0.163	1.14	-191	0.118	2.23	-380	-0.105	Neither	1
	HOH484	0.00	-379	-0.280	0.91	-316	-0.461	0.91	-696	-1.539	Neither	10
	HOH490	0.00	-184	-0.087	1.02	360	0.715	1.02	176	0.509	Protein B	0
	HOH496	0.00	-463	-0.377	0.98	-16	0.234	0.98	-479	-0.887	Neither	17
	HOH518	0.00	-230	-0.128	1.07	-240	-0.278	1.07	-469	-0.860	Neither	0
